# Hydrogenation of Carbamates, Ureas, and Polyurethanes Using Heterogeneous Catalysts

**DOI:** 10.1021/acssuschemeng.5c04473

**Published:** 2025-10-03

**Authors:** Benjamin Sole, Julian S. Kolb, Raymundo Marcial-Hernandez, James Luk, Tai Williams, Oxana V. Magdysyuk, Daylan Sheppard, Gary Walker, Amit Kumar

**Affiliations:** † EaStCHEM, School of Chemistry, 150654 University of St. Andrews, North Haugh, St. Andrews KY16 9ST, U.K.; ‡ 114599 Lubrizol Advanced Materials, Inc., 9911 Brecksville Road, Cleveland, Ohio 44141-3247, United States; § Lubrizol Ltd., Hazelwood, Derby DE56 4AN, U.K.

**Keywords:** Hydrogenation, Palladium, Heterogeneous, Carbamates, Isocyanates, Polyureaurethanes

## Abstract

We report here the hydrogenation of carbamates, ureas, and polyurethanes using heterogeneous catalysts. Under our catalytic conditions, carbamates and urea derivatives can be selectively hydrogenated to formamides and alcohols and amines, whereas polyurethanes were hydrogenatively depolymerized to make diamines and polyols. Recycling of catalysts for the hydrogenative depolymerization of a polyurethane has also been demonstrated 10 times.

## Graphical abstract

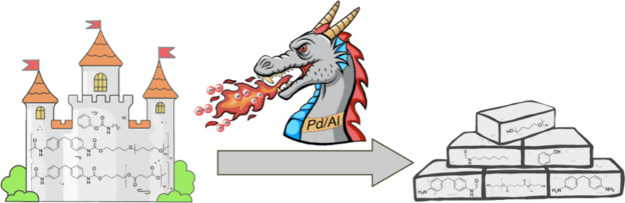

## Introduction

Selective catalytic hydrogenation of carbonyl compounds is an atom-economical and clean approach to carry out functional group transformations and make valuable organic compounds. The hydrogenation of several carbonyl compounds such as aldehydes, ketones, esters, amides, carboxylic acids, carbamates, and ureas have been studied to make alcohols and amines.^1–4^ Among all carbonyl compounds, the hydrogenation of carbamates and urea derivatives is the most challenging due to the low polarizability of carbonyl bonds. The hydrogenation of these substrates was first accomplished by Milstein using ruthenium pincer catalysts to make alcohols, and amines in 2011 (Figure 1A).^5,6^ These reactivities picked up significant attention in the past five years due to their potential to be utilized for the hydrogenative depolymerization of polyurethanes and polyureas.^7–14^ Indeed, a few catalysts for the hydrogenative depolymerization of polyurethanes to diols and diamines have been reported in the past few years.^15^ These are based on pincer complexes of ruthenium, iridium, and manganese (Figure 1A).^16–19^ Recently, we and Baráth have independently reported on the hydrogenative depolymerization of polyureas to diamines and methanol using transition-metal pincer catalysts.^20,21^ Recently, Li^22–24^ and Nozaki^25–27^ have independently reported another mode of hydrogenation of urea derivatives and carbamates, where these substrates can be selectively hydrogenated to make formamides and amines/alcohols (Figure 1B). Although large-scale chemical recycling processes for polyurethanes, particularly glycolysis, are already in operation, they are primarily used for polyol recovery.^28^ This is due to the lack of selectivity for the formation of diamines, as the process also generates significant amounts of byproducts such as carbamates, ureas, and their oligomers. In this context, the approach of catalytic hydrogenation can improve the selectivity toward the formation of amines.

**Figure 1:**
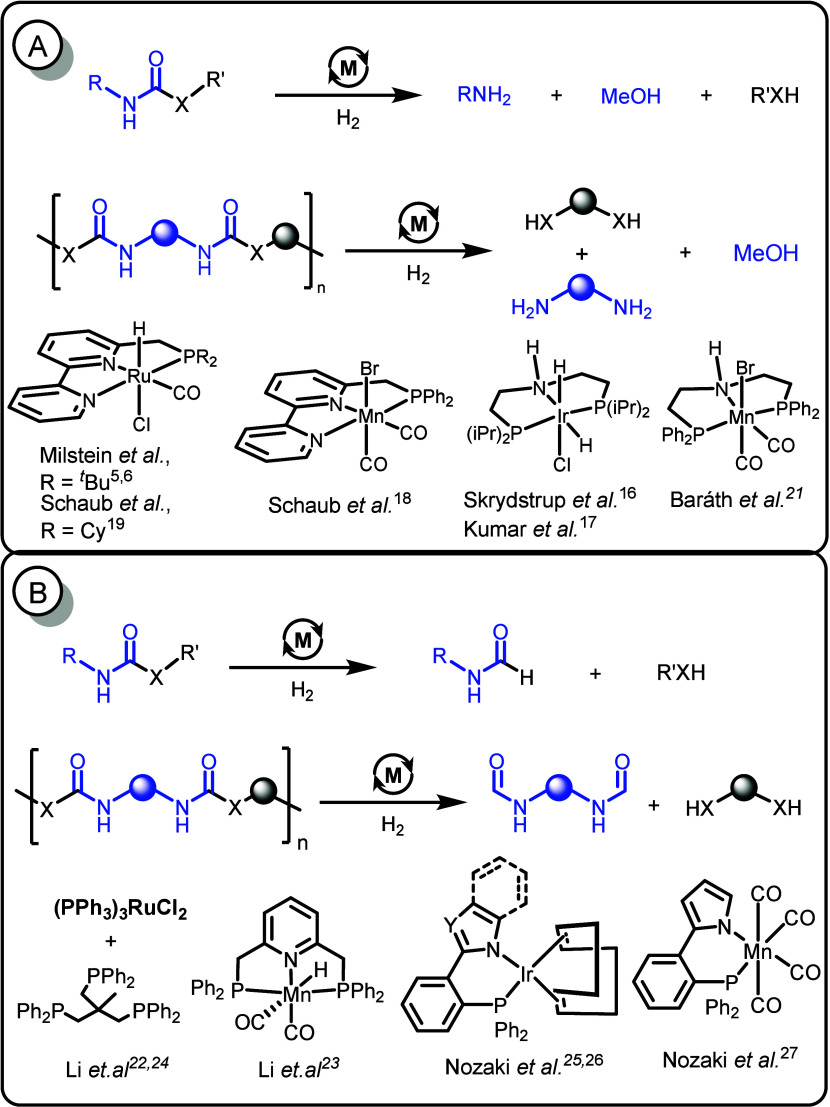
Previous examples of the hydrogenation of carbamates, urea, polyurethanes, and polyureas (X = NH, O); Y = C, N.

Although the reported homogeneously catalyzed hydrogenation processes are cleaner and atom-economic in comparison to using stoichiometric reductants, their large-scale application is challenging due to the use of expensive organometallic catalysts that are difficult to recycle. In this context, the use of a heterogeneous catalyst that can be easily separated and recycled will move the process ahead on the road to commercialization. The hydrogenation of aldehydes, ketones, esters, and amides has been studied using heterogeneous catalysts.^3,29,30^ However, to the best of our awareness, there is no report on the direct hydrogenation of carbamates or urea derivatives using heterogeneous catalysts, although a report on the hydrogenative depolymerization of polyurethanes using NiMo/Al_2_O_3_ has been published during the preparation of this manuscript.^31^ Another related paper describes the depolymerization of polyurethanes using methanolysis combined with hydrogenation in the presence of CO_2_/H_2_ using an inverse ZnO-ZrO_2_/Cu catalyst to make useful feedstock such as aromatic diamines, polyols, and lactones.^32^ Transfer hydrogenation of urea derivatives using methanol as a hydrogen donor reagent in the presence of Ni-doped carbon nanomaterial and Pd/C catalyst has also been reported recently.^12,33^ Herein, we present our studies of the direct hydrogenation of carbamates, urea derivatives, and polyurethanes using commercially available heterogeneous catalysts.

## Results and Discussion

We hypothesized that the hydrogenation of carbamates could be achieved in two steps—first by the dissociation of carbamates to isocyanates and alcohols followed by the hydrogenation of isocyanates to formamides. The dissociation of carbamates to isocyanates and alcohols via thermal cracking has been reported by a few catalysts such as ZnO, Al_2_O_3_, and Bi_2_O_3_ at temperatures in the range of 175–300 °C.^34,35^ In the absence of a catalyst, the dissociation equilibrium is very slow and yields <10% isocyanate at 200 °C for methyl *N*-phenyl carbamate.^34,35^ We envisioned that the dissociation equilibrium of isocyanate could be pushed forward by the continuous hydrogenation of isocyanate to formamides. The reduction of isocyanates to formamides has been reported using silanes^36^ and boranes^37^ in the past. However, to the best of our awareness, the hydrogenation of isocyanates to formamides using a heterogeneous catalyst has been reported only once for a single example using Pd/C catalyst in the presence of NEt_3_.^38^ Inspired by this, we started our investigation by studying the hydrogenation of phenyl isocyanate (1 mmol) in the presence of Pd/C (2.4 mol %) at 30 bar H_2_ pressure and 50 °C for 24 h in 1,4-dioxane solvent. However, the reaction did not lead to the formation of any formanilide (Table 1, entry 1). We then conducted further optimization to hydrogenate isocyanate and understand the effects of catalytic conditions on the reaction outcome. Performing the reaction in the presence of Et_3_N (10 mol %), similar to the previous report on the hydrogenation of isocyanate,^38^ led to the formation of formanilide in 83% yield (entry 2). Using KO^t^Bu instead of Et_3_N led to a similar yield of formanilide (81%, entry 3). Using Pd/Al_2_O_3_ under identical conditions without using any base led to the formation of formanilide in 64% yield (entry 4). Similar to Pd/C when the reaction was performed using Pd/Al_2_O_3_ but in the presence of Et_3_N (10 mol %), the formanilide yield was enhanced, this time to 75% (entry 5). Using K_2_CO_3_ as a base in the case of Pd/Al_2_O_3_ also led to an excellent yield of formanilide (88%, entry 6). Doing a control experiment without using any metal catalyst and/or base did not lead to the formation of any formamide (entries 7, 8).

**Table 1:**
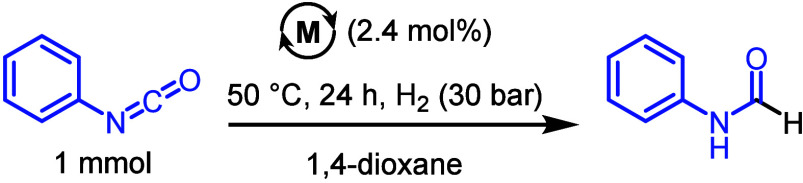
Optimization of the Catalytic Conditions for the Hydrogenation of Phenyl Isocyanate^a^

Entry	Catalyst (2.4 mol %)	Base (10 mol %)	Yield
1.	Pd/C	-	0%
2.	Pd/C	Et_3_N	83%
3.	Pd/C	KO^t^Bu	81%
4.	Pd/Al_2_O_3_	-	64%
5.	Pd/Al_2_O_3_	Et_3_N	75%
6.	Pd/Al_2_O_3_	K_2_CO_3_	88%
7.	-	-	0%
8.	-	Et_3_N	0%

a Reaction conditions: phenyl isocyanate (1 mmol), catalyst (2.4 mol %), base (10 mol %), 1,4-dioxane (1 mL), 50 °C, 24 h. Products were identified by GC-MS, and yield was determined by ^1^H NMR spectroscopy using cyclohexene as an internal standard. See ESI for more details.

With the knowledge of the hydrogenation of isocyanates in hand, we studied the catalytic hydrogenation of carbamates. Using Pd/Al_2_O_3_ (2.4 mol %) and K_2_CO_3_ (10 mol %), only 2% hydrogenation of ethyl phenylcarbamate to formanilide was obtained at 150 °C (50 bar, 24 h, Figure 2A). Notably, the hydrogenation of ethyl phenylcarbamate using Pd/C (2.4 mol %) and Et_3_N (10 mol %) in 1,4-dioxane (50 bar H_2_, 24 h, 150 °C) showed 40% conversion of ethyl phenylcarbamate to a mixture of ethyl *N*-cyclohexylcarbamate (30%) and formanilide (10%) (Figure 2B). Increasing the temperature to 180 °C did not change the above reaction outcome. However, the use of Pd/Al_2_O_3_ (instead of Pd/C) in combination with Et_3_N (50 bar H_2_, 24 h, 150 °C) did increase the conversion of ethyl phenylcarbamate to 70%, but formanilide was obtained in only 5% yield (Figure 2C). The remaining products were obtained as a mixture of ethyl *N*-cyclohexylcarbamate, cyclohexylformamide, and diphenylurea, where diphenyl urea was observed as the major product (60% yield). Interestingly, using Pd/C (5 mol %), K_2_CO_3_ (10 mol %) in toluene at 150 °C, 24 h, and 50 bar H_2_ led to the selective formation of ethyl *N*-cyclohexylcarbamate in 76% yield (Figure 2D).

**Figure 2:**
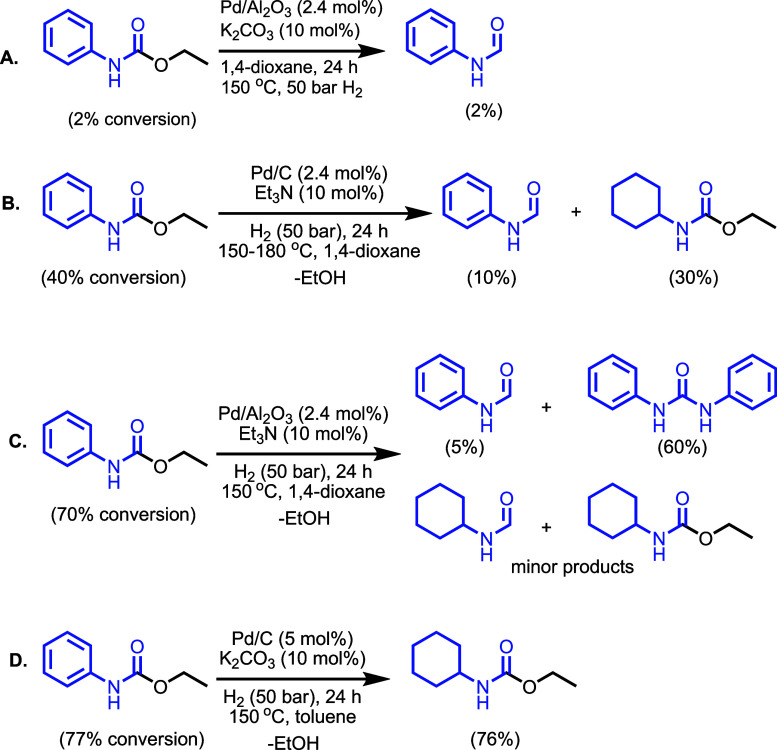
Hydrogenation of ethyl phenylcarbamate.

We hypothesized that the observed reactivity could be due to the lack of dissociation of ethyl phenylcarbamate to phenyl isocyanate and ethanol, leading to the occurrence of the competing reaction, i.e., hydrogenation of the aromatic ring to produce ethyl *N*-cyclohexylcarbamate. We further hypothesized that the dissociation of a carbamate bond to isocyanate will be more favorable in a carbamate made from an aliphatic amine and aromatic alcohol, such as phenyl *N*-octylcarbamate. To test this hypothesis, we performed the hydrogenation of phenyl *N*-octylcarbamate under a range of catalytic conditions as described in Table 2. We started optimizing the reaction conditions using Pd/Al_2_O_3_ (2.4 mol %) and K_2_CO_3_ (10 mol %), in 1,4-dioxane solvent, as it proved to be the most effective for the hydrogenation of isocyanate (Table 1). Indeed, the hydrogenation of phenyl *N*-octylcarbamate using Pd/Al_2_O_3_ (2.4 mol %) and K_2_CO_3_ (10 mol %) in 1,4-dioxane at 150 °C and 50 bar H_2_ pressure led to 100% conversion of the starting material. However, although phenol was obtained in 92% yield, *N*-octyl formamide was obtained in only 43% yield (entry 1). The remaining amine component was obtained as *N*,*N′*-dioctylurea. We speculate that the formation of a urea derivative occurs via decarbonylation of *N*-octylformamide to form octylamine that reacts with isocyanate formed from the thermal dissociation of carbamate. Alternatively, isocyanate could also react with a trace amount of water present in the reaction mixture to form a carbamic acid that can be decarboxylated to form amine. To improve the selectivity toward formamide, we performed further optimization by variation of base, solvent, and catalyst. Performing the reaction in the presence of Et_3_N instead of K_2_CO_3_ increased the yield of formamide only slightly (51%, entry 2). We then tested this reaction in different solvents (toluene, anisole, and THF) using two bases—K_2_CO_3_ and Et_3_N (entries 3–8). Although the conversions of phenyl *N*-octylcarbamate in these cases were found to be high, the selectivity of *N*-octyl formamide remained poor (40–54%). Interestingly, a higher selectivity was obtained when *t*-amyl alcohol (*t*AmOH) was used as a solvent, leading to the formation of formamide in 71% yield in the case of the K_2_CO_3_ base (entry 9). Use of Et_3_N (entry 10) and Cs_2_CO_3_ (entry 11) as a base, keeping the remaining conditions the same, lowered the selectivity of *N*-octylformamide, whereas remarkably, when KBH_4_ was used as a base, the selectivity of *N*-octylformamide was found to be 83% (entry 12, TON for formamide: 35). These studies suggest *t*AmOH to be an optimum solvent and KBH_4_ to be an optimum base. To assess whether KBH_4_ can also function as a reductant, we carried out the reduction of phenyl *N*-octylcarbamate under identical conditions but without applying H_2_ pressure (entry 13). Analysis of the reaction mixture revealed complete conversion of the starting material; however, *N*-octylformamide was obtained in only 14% yield, with the remaining products being dioctylurea (34%) and *t*-amyl octylcarbamate (44%). To further probe into the role of base, we performed this reaction using Pd/Al_2_O_3_ (2.4 mol %) and K_2_CO_3_ (10 mol %) in *t*AmOH in the absence of H_2_ (entry 14). This reaction did not lead to the formation of any formamide or amine. In this case, phenyl *N*-octylcarbamate was fully converted to phenol, dioctyl urea, and *t*-amyl octylcarbamate. Performing the reaction in the presence of KBH_4_ with (entry 15) or without (entry 16) H_2_, but in the absence of Pd/Al_2_O_3_, showed similar results, producing *N*-octyl formamide in yields of 15%, and 19%, respectively whereas the rest of the amine-containing products were observed as *t*-amyl carbamate and dioctyl urea. These experiments confirm that H_2_ is needed for the formation of formamides in high yields and that base is able to catalyze the transcarbamoylation process, which is a competing reaction, forming *t*-amyl octylcarbamate from the reaction of phenyl *N*-octylcarbamate with *t*AmOH.

**Table 2:**
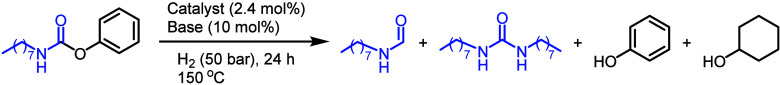
Optimization of the Catalytic Conditions for the Hydrogenation of Phenyl *N*-Octylcarbamate^a^

Entry	Catalyst	Solvent	Base	Conversion (%)	Formamide Yield (%)	Urea Yield (%)	Phenol Yield (%)	Cyclohexanol Yield (%)
1	Pd/Al_2_O_3_	1,4-dioxane	K_2_CO_3_	100	43	57	92	0
2	Pd/Al_2_O_3_	1,4-dioxane	Et_3_N	98	51	26	69	6
3	Pd/Al_2_O_3_	toluene	K_2_CO_3_	100	51	41	76	14
4	Pd/Al_2_O_3_	toluene	Et_3_N	100	40	21	64	0
5	Pd/Al_2_O_3_	anisole	K_2_CO_3_	100	42	37	76	0
6	Pd/Al_2_O_3_	anisole	Et_3_N	100	43	11	64	0
7	Pd/Al_2_O_3_	THF	K_2_CO_3_	100	54	17	58	4
8	Pd/Al_2_O_3_	THF	Et_3_N	100	50	18	54	0
9	Pd/Al_2_O_3_	*t*-amyl OH	K_2_CO_3_	100	71	17	87	4
10	Pd/Al_2_O_3_	*t*-amyl OH	Et_3_N	100	60	14	68	0
11	Pd/Al_2_O_3_	*t*-amyl OH	Cs_2_CO_3_	100	45	30	57	0
12	Pd/Al_2_O_3_	*t*-amyl OH	KBH_4_	100	83	10	85	14
13^b^	Pd/Al_2_O_3_	*t*-amyl OH	KBH_4_	100	14	32	99	0
14^b^	Pd/Al_2_O_3_	*t*-amyl OH	K_2_CO_3_	100	0	60	93	0
15	-	*t*-amyl OH	KBH_4_	100	15	26	99	0
16^b^	-	*t*-amyl OH	KBH_4_	100	19	23	99	0
17	Pd/C	*t*-amyl OH	KBH_4_	100	75	7	65	34
18	Pt/C	*t*-amyl OH	KBH_4_	100	16	51	90	0
19	Ru/Al_2_O_3_	*t*-amyl OH	KBH_4_	100	18	39	93	0
20	Ni/Si–Al_2_O_3_	*t*-amyl OH	KBH_4_	100	18	35	93	0
21	-	*t*-amyl OH	-	0	0	0	0	0

a Reaction conditions: phenyl *N*-octylcarbamate (1 mmol), catalyst (2.4 mol %), base (10 mol %), solvent 1 mL, H_2_ (50 bar), 24 h, 150 °C. The products were identified by GC-MS, and the conversion and yields were estimated by ^1^H NMR spectroscopy using 1,1′-diphenylethylene as an internal standard (see ESI, Section 3.4). Two other uncharacterized side products were obtained in each case with the *m*/*z* values in GC-MS of 174 and 211 Da.

b No hydrogen pressure.

Using the *t*AmOH solvent and KBH_4_ base, we studied other metal catalysts for the hydrogenation of phenyl *N*-octylcarbamate, keeping the remaining conditions the same. Although a high yield of *N*-octylformamide (75%) was obtained in the case of Pd/C (entry 17), significantly poorer yields were obtained in the case of Pt/C, Ru/Al_2_O_3_, and Ni/Si–Al_2_O_3_ (entries 18–20). Based on these studies, our optimum conditions for the hydrogenation of phenyl *N*-octylcarbamate are Pd/Al_2_O_3_ (2.4 mol %), KBH_4_ (10 mol %), *t*AmOH, 50 bar H_2_ pressure, 150 °C, and 24 h reaction time (entry 12). In a control experiment, when the hydrogenation of phenyl *N*-octylcarbamate was performed without any metal catalyst or base in *t*AmOH (entry 21), no conversion of phenyl *N*-octylcarbamate was obtained, suggesting the crucial role of catalyst in the hydrogenation process.

To verify our hypothesis that the hydrogenation of carbamates occurs through an isocyanate intermediate, we studied the thermal cracking of phenyl *N*-octyl carbamate using TGA-MS (thermogravimetric analysis–mass spectrometry). In this study, phenyl *N*-octyl carbamate (∼65 mg) in the presence of Pd/Al_2_O_3_ (13 mg) or Pd/C (5 mg), as well as without any metal catalyst, was heated in an N_2_ atmosphere at 150 °C for 1 h (see ESI, Section 7 for more details). Mass loss was monitored over time, along with the release of molecular species by mass spectrometry. Interestingly, in all cases, the formation of *N*-octyl isocyanate was observed by mass spectrometry, suggesting that under the reaction conditions, thermal dissociation of carbamates to isocyanates and alcohols is possible. The results also showed that the rate of dissociation gets enhanced in the presence of Pd/Al_2_O_3_.

Using the optimized conditions for the hydrogenation of phenyl *N*-octylcarbamate, we studied the hydrogenation of other carbamates and urea derivatives (Table 3). The hydrogenation of phenyl *N*-cyclohexylcarbamate showed complete conversion, forming a high yield of cyclohexyl formamide (81%) and phenol (81%, entry 2). *N*,*N′*-dicylcohexylurea was observed as a minor product. Under the same reaction conditions, diphenyl carbamate also showed complete conversion, and the formation of phenol was obtained in 86% yield. However, relatively lower selectivity (57%) toward formanilide was obtained. Aniline was observed as a byproduct in 22% yield, likely via the decarbonylation of formanilide. Similarly, methyl *N*-phenyl carbamate and ethyl *N*-phenyl carbamate led to lower conversion of carbamate and lower selectivity of formamides (entries 4, 5). These experiments suggest that carbamates prepared from electron-rich isocyanates and electron-deficient alcohols are more suitable candidates for their selective hydrogenation to formamides under these conditions in comparison to carbamates prepared from electron-deficient isocyanates and electron-rich alcohols. Indeed, phenyl 4-methoxy carbamate showed a higher yield/selectivity of formamide (78%, entry 6) in comparison to diphenyl carbamate (57%, entry 3). Phenyl (6-hydroxyhexyl)­carbamate (entry 7) and phenyl benzylcarbamate (entry 8) also showed very good yields of corresponding formamides and alcohols. In comparison, diphenyl 1,4-phenylenedicarbamate showed only 10% conversion to phenol (10%) and N-(4-aminophenyl) formamide (5%, entry 9).

**Table 3:**
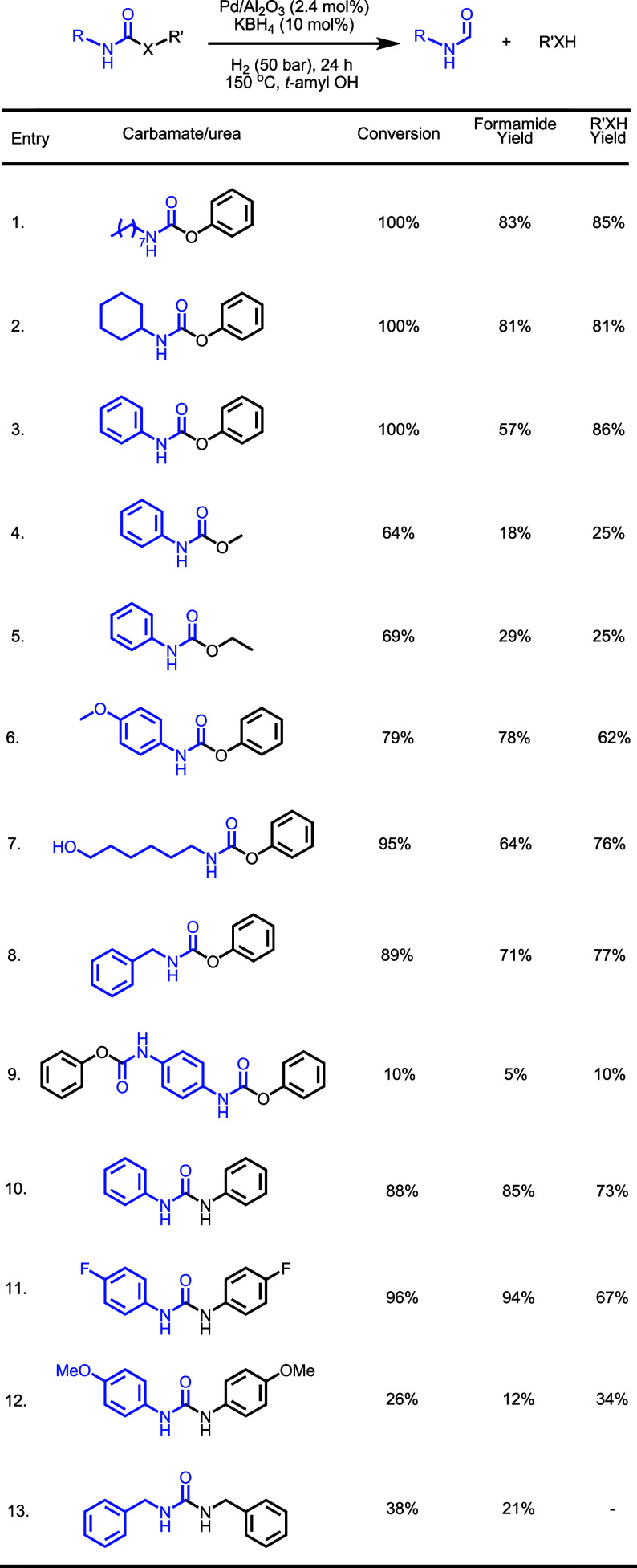
Hydrogenation of Carbamates and Urea Derivatives^a^

a Reaction conditions: Carbamate (1 mmol), Pd/Al_2_O_3_ (2.4 mol %), KBH_4_ (10 mol %), *t*-amyl alcohol (1 mL), H_2_ (50 bar), 24 h, 150 °C. The products were identified by GC-MS and the conversion and yields were estimated by ^1^H NMR spectroscopy using 1,1′-diphenylethylene or 1,3,5-trimethoxybenzene as internal standards. X = O, NH.

In addition to carbamate derivatives, we also studied the hydrogenation of a few urea derivatives under this catalytic protocol. The hydrogenation of diphenyl urea and 1,3-bis­(4-fluorophenyl)­urea led to excellent conversion of urea derivative to the corresponding formamides (entries 10, 11). However, lower conversions were obtained in the cases of 1,3-bis­(4-methoxyphenyl)­urea (entry 12) and dibenzylurea (entry 13).

Having studied the hydrogenation of carbamates and urea derivatives, we applied this approach to the hydrogenative depolymerization of flexible polyurethane samples supplied by Lubrizol (colorless pellets of 2–4 mm diameter)—polyurethane made from polyether polyols (**PU1**) and polyurethane made from polyesterol (**PU2**). We performed the hydrogenation of the polyurethane sample (**PU1**) at 180 °C as 150 °C did not show full conversion in the case of ethyl phenylcarbamate (Figure 2), which could be considered a model carbamate for commercial polyurethanes. Remarkably, using Pd/Al_2_O_3_ (10 wt %) and KBH_4_ (1 wt %) in *t*AmOH at 180 °C (24 h and 50 bar H_2_), almost quantitative conversion of **PU1** (*M*_n_ = 66 337 Da; PDI = 2.7, 3.3 g) was obtained. The depolymerization products were separated using column chromatography (weight recovery of 90%), which confirmed the formation of polyetherol (1.97 g, *M*_n_ = 2258 Da; PDI = 1.5), aromatic diamine (0.54 g), and an aromatic formamide–amine (0.35 g) as mentioned in Figure 3A. Furthermore, we studied the hydrogenative depolymerization of another commercial flexible polyurethane (**PU2**, *M*_n_ = 169, 204 Da, PDI = 2.5, 3.3 g) made from polyester polyol under the same reaction conditions. An 83% conversion of **PU2** was observed and 4,4′-methylenedianiline (0.45 g) and polyesterol (1.8 g, *M*_n_ = 1249 Da; PDI = 1.8) were isolated as major products from the reaction (weight recovery: 71%). Interestingly, the formation of 1,6-hexanediol or 1,4-butanediol was not observed at all; however, a small amount (<50 mg) of transesterification product from the reaction of polyesterol with *t*AmOH was obtained. To further confirm the catalyst’s selectivity for ester hydrogenation, we performed the hydrogenation of pentyl valerate under identical conditions. In this case, no pentanol formation was observed. Instead, only ∼5% of pentyl valerate was converted to *t*-Amyl pentanoate via transesterification with *t*AmOH. These results confirm that the catalyst and reaction conditions are highly selective for the hydrogenation of carbamates over esters. Motivated by these results, we performed hydrogenation of a polyurethane sample obtained from a kitchen sponge. Satisfyingly, this also led to the complete conversion of polyurethane with phenylenediamine and a polyol (*M*_w_ = 4110 Da, PDI = 1.1) isolated as the two major products. The three reactions were also conducted in the absence of hydrogen gas, as it was reported by Skrydstrup that *t*-amyl alcohol can also mediate the deconstruction of polyurethanes although at higher temperatures (225 °C) under basic conditions.^39^ In the case of **PU1** and **PU2**, 26% and 34% conversions were obtained, respectively, whereas in the case of the kitchen sponge, no conversion was obtained.

**Figure 3:**
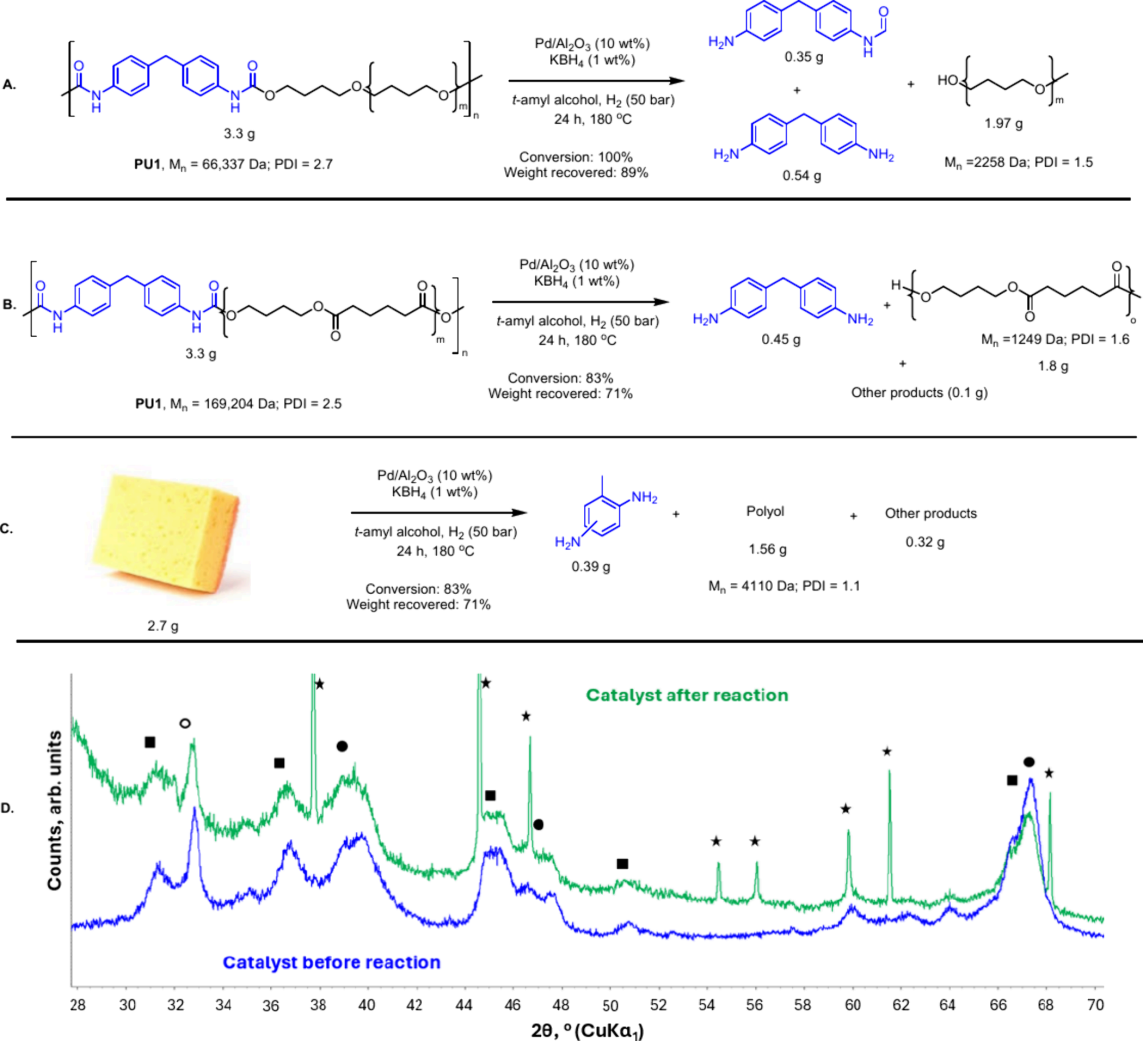
Hydrogenative depolymerization of a polyurethane sample containing polyetherol (A) and polyesterol (B). Hydrogenative depolymerization of a kitchen sponge (C) and powder XRD pattern of the Pd/Al_2_O_3_ sample (blue) as well as the catalyst recovered after the hydrogenation of isocyanate, Table 1, entry 6 (green). * represents signals from KBH_4_; ● – Pd; ○ – PdO; ■ – γ-Al_2_O_3_.

Since one of the motivations for using heterogeneous catalysts was catalyst recycling, we studied the nature of catalysts by powder X-ray diffraction (XRD) before and after the reaction. Powder XRD of the commercial sample of Pd/Al_2_O_3_ showed the presence of Pd (3%), PdO (5%), and γ-Al_2_O_3_ (92%). The average crystallite size of both palladium phases was estimated to be ∼12 nm, whereas that of γ-Al_2_O_3_ was estimated to be ∼6 nm. A powder XRD of the catalyst sample recovered after the hydrogenation of isocyanate (from Table 1, entry 6) showed that signals from the Pd/Al_2_O_3_ catalyst sample are still present (Figure 3D). Additionally, strong sharp peaks corresponding to KBH_4_ (crystallite size: ∼85 nm) were observed. The corresponding weight percentage of KBH_4_ was found to be ∼7%, slightly lower than what was started. A powder XRD of the catalyst recovered from the hydrogenation of phenyl *N*-octyl carbamate (Table 2, entry 12) did not show the presence of any KBH_4_—only Pd, PdO, and γ-Al_2_O_3_ were detected. We speculate that KBH_4_ got consumed by its reaction with *t*AmOH or with moisture during catalyst separation (see ESI, Section 8). These studies suggested that although Pd/Al_2_O_3_ was stable under the reaction conditions, it is likely that a fresh batch of KBH_4_ would need to be added during catalyst recycling studies.

With this insight in hand, we studied catalyst recycling for the hydrogenative depolymerization of **PU1** (1.65 g, *M*_n_ = 66 337 Da; PDI **=** 2.7) using Pd/Al_2_O_3_ (10 wt %) and KBH_4_ (1 wt %) in *t*AmOH at 180 °C for 24 h under 50 bar H_2_ pressure. At the end of the reaction, the catalyst was separated by centrifugation and washed with methanol, followed by vacuum drying for subsequent recycling. Analysis of the separated reaction mixture by GPC showed *M*_n_ to be 1748 Da and PDI to be 1.84, confirming complete depolymerization, as also shown in Figure 3A. The amounts of the formed aromatic diamine and formamide were found to be 0.23 g and 0.2 g, respectively, as estimated by ^1^H NMR spectroscopy using diphenyl ethylene as an internal standard. The separated catalyst was successfully recycled ten times using this procedure, with 1 wt % fresh KBH_4_ added in each cycle. Complete depolymerization of **PU1** was observed in every run, as confirmed by the molecular weight (*M*_n_) of the reaction mixture, which fell in the range of 1613–1866 Da—matching that of polyetherol—along with the expected diamine fraction (0.23–0.28 g) (Figure 4, see ESI, Section 9).

**Figure 4:**
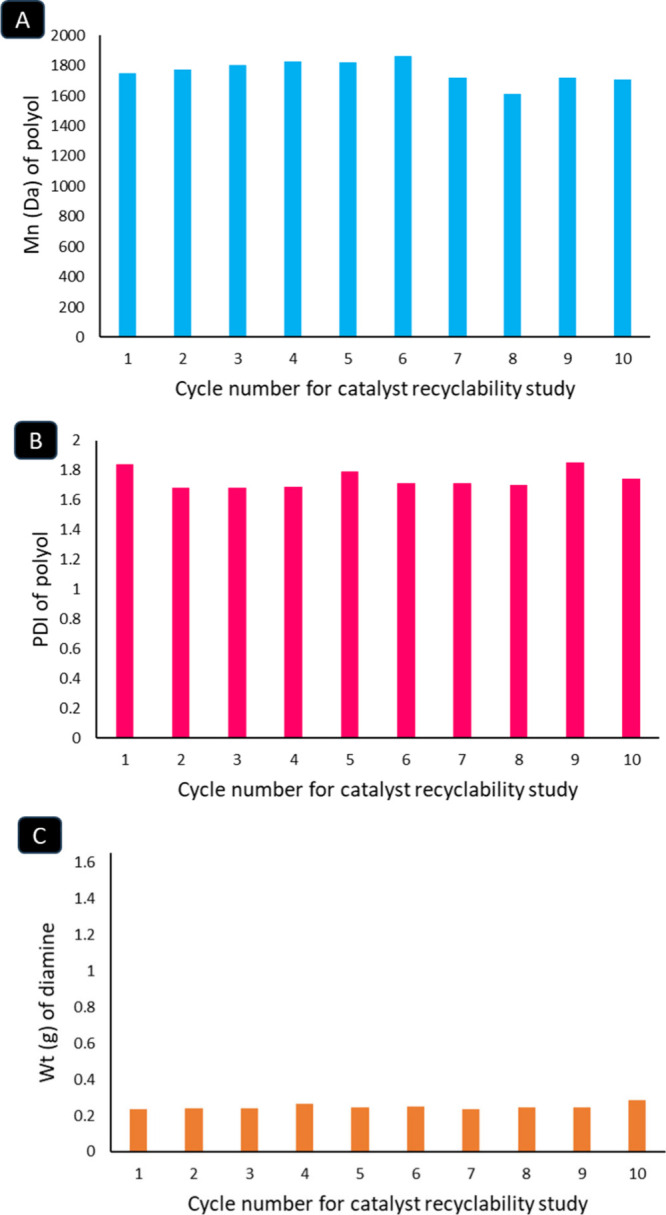
*M*_n_ (Da) (A) and PDI (B) of polyol as well as the weight (g) of aromatic diamine (C) obtained from the hydrogenative depolymerization of **PU1** over 10 runs of recycling catalyst. Reaction conditions: (**PU1**, 1.65 g, *M*_n_ = 66 337 Da; PDI = 2.7), Pd/Al_2_O_3_ (10 wt %), KBH_4_ (1 wt %), *t*AmOH, 180 °C, 24 h, 50 bar H_2_.

The reaction mixtures obtained after catalyst separation from the first and second runs were also analyzed by microwave plasma atomic emission spectroscopy (MP-AES). No palladium was detected in the analysis range, confirming that the depolymerization products are free of palladium and that the catalyst remains stable (ESI, Section 6).

## Conclusions

In conclusion, we have demonstrated that carbamates, urea derivatives, and polyurethanes can be hydrogenated to form formamides, amines, and alcohols using commercially available heterogeneous catalysts—most notably, Pd/Al_2_O_3_ in the presence of a base. Three polyurethane samples (including both technical-grade materials and real-life waste) were successfully hydrogenatively depolymerized to yield diamines and polyols in excellent yields. The reaction conditions were found to be highly selective for carbamate hydrogenation over ester reduction, enabling the selective production of polyesterol from the hydrogenative depolymerization of **PU2**. Furthermore, catalyst recyclability was demonstrated over ten cycles in the hydrogenative depolymerization of **PU1**.

## Supplementary Material



## Data Availability

The research data supporting this publication can be accessed at https://doi.org/10.17630/87bc5db4-ad8f-4bd7-a54c-b979bb219a53.

## Notes

Additional experimental procedure, characterization, and hydrogenation information and analytical data (PDF)

## References

[1] Filonenko G. A., Van Putten R., Hensen E. J. M., Pidko E. A. (2018). Catalytic (de)­Hydrogenation
Promoted by Non-Precious Metals-Co, Fe and Mn: Recent Advances in
an Emerging Field. Chem. Soc. Rev..

[2] Clarke M. L. (2012). Recent
Developments in the Homogeneous Hydrogenation of Carboxylic Acid Esters. Catal. Sci. Technol..

[3] Cabrero-Antonino J. R., Adam R., Papa V., Beller M. (2020). Homogeneous and Heterogeneous
Catalytic Reduction of Amides and Related Compounds Using Molecular
Hydrogen. Nat. Commun..

[4] Werkmeister S., Junge K., Beller M. (2014). Catalytic
Hydrogenation of Carboxylic
Acid Esters, Amides, and Nitriles with Homogeneous Catalysts. Org. Process Res. Dev..

[5] Balaraman E., Gunanathan C., Zhang J., Shimon L. J. W., Milstein D. (2011). Efficient
Hydrogenation of Organic Carbonates, Carbamates and Formates Indicates
Alternative Routes to Methanol Based on CO 2 and CO. Nat. Chem..

[6] Balaraman E., Ben-David Y., Milstein D. (2011). Unprecedented Catalytic Hydrogenation
of Urea Derivatives to Amines and Methanol. Angew. Chem., Int. Ed..

[7] Miura T., Naruto M., Toda K., Shimomura T., Saito S. (2017). Multifaceted Catalytic Hydrogenation
of Amides via Diverse Activation
of a Sterically Confined Bipyridine-Ruthenium Framework. Sci. Rep..

[8] Liu X., Werner T. (2021). Indirect Reduction of CO2 and Recycling of Polymers
by Manganese-Catalyzed Transfer Hydrogenation of Amides, Carbamates,
Urea Derivatives, and Polyurethanes. Chem. Sci..

[9] Vom
Stein T., Meuresch M., Limper D., Schmitz M., Hölscher M., Coetzee J., Cole-Hamilton D. J., Klankermayer J., Leitner W. (2014). Highly Versatile Catalytic Hydrogenation
of Carboxylic and Carbonic Acid Derivatives Using a Ru-Triphos Complex:
Molecular Control over Selectivity and Substrate Scope. J. Am. Chem. Soc..

[10] Kothandaraman J., Kar S., Sen R., Goeppert A., Olah G. A., Prakash G. K. S. (2017). Efficient
Reversible Hydrogen Carrier System Based on Amine Reforming of Methanol
Scheme 1. Aqueous Reforming of Methanol. J.
Am. Chem. Soc..

[11] Das U. K., Kumar A., Ben-David Y., Iron M. A., Milstein D. (2019). Manganese
Catalyzed Hydrogenation of Carbamates and Urea Derivatives. J. Am. Chem. Soc..

[12] Kamaraj K. H., Dixneuf P., Sundaram G. B., Reek J. N. H., Beromeo
Bheeter C. (2024). Pd/C-Catalyzed Selective N-Monomethylation by Transfer
Hydrogenation of Urea Derivatives Using Methanol as H2 and C1 Sources. Chem. - A Eur. J..

[13] Wei Z., Li H., Wang Y., Liu Q. (2023). A Tailored Versatile and Efficient
NHC-Based NNC-Pincer Manganese Catalyst for Hydrogenation of Polar
Unsaturated Compounds. Angew. Chem., Int. Ed..

[14] Wang Z., Yan X., Ma N., Liu S., Han P., Li H., Mahmood Q., Li L., Liu Q. (2023). Highly Efficient Hydrogenation
of Carbamates Catalyzed by Pincer Ruthenium Complexes. J. Catal..

[15] Kumar A., Gao C. (2021). Homogeneous (De)­Hydrogenative Catalysis for Circular Chemistry -
Using Waste as a Resource. ChemCatChem..

[16] Gausas L., Kristensen S. K., Sun H., Ahrens A., Donslund B. S., Lindhardt A. T., Skrydstrup T. (2021). Catalytic Hydrogenation of Polyurethanes
to Base Chemicals: From Model Systems to Commercial and End-of-Life
Polyurethane Materials. JACS Au.

[17] Kumar A., Von Wolff N., Rauch M., Zou Y. Q., Shmul G., Ben-David Y., Leitus G., Avram L., Milstein D. (2020). Hydrogenative
Depolymerization of Nylons. J. Am. Chem. Soc..

[18] Zubar V., Haedler A. T., Schütte M., Hashmi A. S. K., Schaub T. (2022). Hydrogenative
Depolymerization of Polyurethanes Catalyzed by a Manganese Pincer
Complex. ChemSusChem.

[19] Zhou W., Neumann P., Al Batal M., Rominger F., Hashmi A. S. K., Schaub T. (2021). Depolymerization of
Technical-Grade Polyamide 66 and
Polyurethane Materials through Hydrogenation. ChemSusChem.

[20] Kumar A., Luk J. (2021). Catalytic Hydrogenation
of Urea Derivatives and Polyureas. J. Org. Chem..

[21] Liu X., Zuo Y., Kallmeier F., Mejía E., Tin S., de Vries J. G., Baráth E. (2022). Hydrogenative Depolymerization of Silicon-Modified
Polyureas. Chem. Commun..

[22] Zhu J., Zhang Y., Wen Z., Ma Q., Wang Y., Yao J., Li H. (2023). Highly Efficient Ruthenium-Catalyzed
Semi-Hydrogenation
of Urea Derivatives to Formamides. Chem. - A
Eur. J..

[23] Zhu J., Zhang S., Wang Y., Yao J., Hong X., Li H. (2025). Noninnocent Spectator Ligands Facilitate CO Ligand-Stabilized Mn­(I)
Metal-Catalyzed Hydrogenation of Urea Derivatives or Carbamates to
the More Reactive Formamides. ACS Catal..

[24] Zhu J., Wang Y., Yao J., Li H. (2024). Switching the Hydrogenation
Selectivity of Urea Derivatives via Subtly Tuning the Amount and Type
of Additive in the Catalyst System. Chem. Sci..

[25] Iwasaki T., Tsuge K., Naito N., Nozaki K. (2023). Chemoselectivity Change
in Catalytic Hydrogenolysis Enabling Urea-Reduction to Formamide/Amine
over More Reactive Carbonyl Compounds. Nat.
Commun..

[26] Iwasaki T., Yamada Y., Naito N., Nozaki K. (2024). Chemoselective Hydrogenolysis
of Urethanes to Formamides and Alcohols in the Presence of More Electrophilic
Carbonyl Compounds. J. Am. Chem. Soc..

[27] Iwasaki T., Saito N., Yamada Y., Ajiro S., Nozaki K. (2024). Hydrogenolysis
of Urethanes and Ureas Catalyzed by Manganese Complex Supported by
Bidentate PN Ligand. Organometallics.

[28] Rossignolo G., Malucelli G., Lorenzetti A. (2024). Recycling of Polyurethanes: Where
We Are and Where We Are Going. Green Chem..

[29] Mäki-Arvela P., Hájek J., Salmi T., Murzin D. Y. (2005). Chemoselective Hydrogenation
of Carbonyl Compounds over Heterogeneous Catalysts. Appl. Catal. A Gen..

[30] Luneau M., Lim J. S., Patel D. A., Sykes E. C. H., Friend C. M., Sautet P. (2020). Guidelines to Achieving High Selectivity
for the Hydrogenation
of α,β-Unsaturated Aldehydes with Bimetallic and Dilute
Alloy Catalysts: A Review. Chem. Rev..

[31] An W., Liu X., Zhang X., Gao R., Chen Z., Hou Y., Du H. (2025). Catalytic Deconstruction of Commercial and End-Of-Life Polyurethane
with Heterogeneous Hydrogenation Catalyst. ChemSusChem.

[32] Sun B., Zou J., Qiu W., Tian S., Wang M., Tang H., Wang B., Luan S., Tang X., Wang M., Ma D. (2024). Chemical Transformation
of Polyurethane into Valuable Polymers. Natl.
Sci. Rev..

[33] Kamaraj K., Dixneuf P. H., Sundaram G. B., Bheeter C. B. (2025). Nickel-Doped Carbon
Nanomaterial Catalyzed Transfer Hydrogenation of Unsymmetrical Urea
Using Methanol as H2 Source. ChemCatChem..

[34] Wang P., Liu S., Deng Y. (2017). Important Green Chemistry and Catalysis: Non-Phosgene
Syntheses of Isocyanates - Thermal Cracking Way. Chin. J. Chem..

[35] Zamani S., Lange J.-P., Kersten S. R. A., Ruiz M. P. (2022). Polyurethane Recycling:
Conversion of Carbamates—Catalysis, Side-Reactions and Mole
Balance. Polymers..

[36] Ojima I., Inaba S., Nagai Y. (1973). A Novel Route
to Formamides and Their
Derivatives. Reduction of Isocyanates via Hydrosilylation Catalyzed
by Palladium. Tetrahedron Lett..

[37] Kumar R., Sharma V., Banerjee S., Vanka K., Sen S. S. (2023). Controlled
Reduction of Isocyanates to Formamides Using Monomeric Magnesium. Chem. Commun..

[38] Howell H. G. (1983). The Hydrogenation
of Isocyanates. Synth. Commun..

[39] Johansen M. B., Donslund B. S., Kristensen S. K., Lindhardt A. T., Skrydstrup T. (2022). Tert-Amyl Alcohol-Mediated Deconstruction
of Polyurethane
for Polyol and Aniline Recovery. ACS Sustain.
Chem. Eng..

