# Supplementary material for: Hydrogenation of Carbamates, Ureas, and Polyurethanes Using Heterogeneous Catalysts
Source: ACS Sustain Chem Eng. 2025 Oct 3;13(41):17173–81. doi: 10.1021/acssuschemeng.5c04473 (PMC12541806; doi:10.1021/acssuschemeng.5c04473)
Supplement: Supplementary file 1 [file sc5c04473_si_001.pdf]

Hydrogenation of Carbamates, Ureas, and Polyurethanes using Heterogeneous Catalysts

Benjamin Sole,[a] Julian S. Kolb,[a] Raymundo Marcial Hernandez,[a] James Luk,[a] Tai Williams,[a] Oxana V. Magdysyuk,[a] Daylan Sheppard,[b] Gary Walker,[c] and Amit Kumar[a]*

[a]*EaStCHEM, School of Chemistry, University of St. Andrews, North Haugh, St. Andrews, KY16 9ST, UK.*
[b] *Lubrizol Advanced Materials, Inc., 9911 Brecksville Road, Cleveland, Ohio, 44141-3247.*
[c]*Lubrizol Ltd., Hazelwood, Derby DE56 4AN, U.K.*

Table of Contents

1. General information *1*

2. Hydrogenation of isocyanates *2*

2.1 Procedure for the hydrogenation of isocyanates 2

2.2 Analytical data for the hydrogenation of isocyanates 4

3. Phenyl N-octylcarbamate hydrogenation optimization *17*

3.1 Synthesis of phenyl *N*-octylcarbamate 17

3.2 Characterization of phenyl *N*-octylcarbamate 17

3.3 Characterization of individual products from phenyl *N*-octylformamide hydrogenation 19

3.4 Procedure for the hydrogenation of phenyl *N*-octylcarbamate 21

3.5 Analytical data for the hydrogenation of phenyl *N*-octylcarbamate 22

4. Hydrogenation of carbamates and ureas *65*

4.1 Procedure for the hydrogenation of carbamates and ureas 65

4.2 Analytical data for the hydrogenation of carbamates and ureas 66

5. Hydrogenative depolymerization of polyurethanes *86*

5.1 Procedure for the hydrogenation of polyurethanes 86

5.2 Analytical data for the starting polyurethanes 87

5.3 Analytical data for the hydrogenation of polyurethanes 93

6. MP-AES Study 109

7. TGA-MS study for the thermal cracking of phenyl N-octyl carbamate *111*

8. Powder XRD *113*

9. Polyurethane recycling *114*

10. References: *131*

1. General information

All experiments were carried out under an inert atmosphere of argon using standard Schlenk techniques or inside a glovebox with an argon atmosphere unless specified. The catalytic reactions were carried out in a vessel equipped with a bursting disk set well below the pressure vessel threshold. Reproduction of these experiments or similar ones should only be carried out using appropriate equipment. A blast shield should be used when manipulations are carried out using appropriate risk assessment. Thermoplastic polyurethanes were supplied by The Lubrizol Corporation and used as received. All other chemicals were purchased from Sigma-Aldrich, TCI Chemicals, Strem, or Alfa-Aesar and used as received. Metal catalysts used were Pd/C (10 wt%) (Thermo-Fisher), Pd/Al_2O_3 (5 wt%) (Sigma-Aldrich), Ru/Al_2O_3 (5 wt %) (Thermo-Scientific), Pt/C (10 wt %) (Alfa-Aesar), and $Ni/(SiAl_2O_3)$ (66 wt %) (Thermo Fisher). 1,4-dioxane, tert-amyl alcohol, and THF were degassed through sparging with a needle using argon and dried using 4 Å molecular sieves purchased from Sigma-Aldrich. The sieves were activated by heating them under vacuum to 120 °C for 24 hours. Consequently, they were added to the solvents in question and left for 24 hours to dry. MeOD and $CDCl_3$ were purchased from Sigma-Aldrich and used as received.

^{1}H NMR experiments were carried out at 298 K using a Bruker Avance II 400 (400 MHz) or Bruker Avance III 500 (500 MHz) and reported in ppm (δ). ^{13}C{1H} NMR experiments were carried out at 298 K using Bruker Avance III 500 (125 MHz ^{13}C) or a Bruker Avance II 400 (125 MHz ^{13}C) and reported in ppm (δ).

GC-MS samples were prepared in HPLC grade DCM and run on an Agilent 8860 GC system coupled to an Agilent 5977B EI instrument. Helium was used as carrier gas with a column flow of 1.87 mL/min. Ion source temperature of 200 ^{o}C, and injection temperature of 330 ^{o}C were used. The oven temperature range was from 50 ^{o}C to 250 ^{o}C (start with 50 ^{o}C, hold for 4.2 minutes, followed by a temperature ramp of 10°C/minute for 15 minutes and stay at 250 ^{o}C until 39 minutes).

Powder X-ray diffraction (PXRD) measurements were performed with Empyrean diffractometer (Malvern PAnalyticalal) in reflection geometry using monochromatic Cu-radiation. For sample preparation, the circular hole with a diameter: 10 mm, and depth: 0.2 mm) present in the silicon substrate was filled with sample and pressed to level the sample with

the substrate. The substrate was then mounted on the powder diffractometer (Malvern PAnalyticalal). The silicon substrate is a single crystal which cut out in a special way so that it does not provide diffraction signals.The crystalline size was determined from peak broadening using the common software Topas Academic (A. A. Coelho, *Journal of Applied Crystallography*, 2018, **51**, 210–218).

Number-average (Mn) and weight-average (Mw) molecular weights were determined by gel permeation chromatography (GPC) using an Agilent 1260 InfinityLab II GPC fitted with a refractive index (RI) detector (35 °C). The single (plus guard column) Agilent PolarGel column setup was contained within an oven (35 °C). DMF and THF were used as eluents at a flow rate of 1.0 mL min^{-1}. Samples were dissolved in the eluent (2.0 mg mL^{-1}), filtered (0.2 µm pore size) and run immediately. The calibration was conducted using a series of monodisperse poly(ethylene glycol) (Mn = 194 – 20,000 g mol^{-1}) and poly(ethylene oxide) (Mn = 30,000 – 50,000 g mol^{-1}) standards obtained from Agilent Technologies.

2. Hydrogenation of isocyanates

2.1 Procedure for the hydrogenation of isocyanates

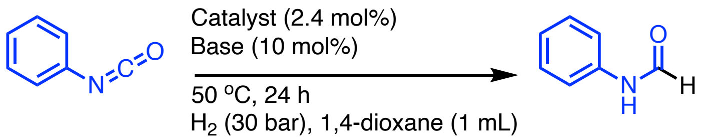

Figure 1: General scheme for the hydrogenation of isocyanates.

The reactor (Figure 2) and all necessary chemicals were transferred to the glovebox. Phenyl isocyanate (1 mmol), catalyst (2.4 mol%), base (10 mol%) and 1,4-dioxane (1 mL) were added to 2 mL HPLC vials, object A, alongside a magnetic stirrer bar. The vials were added to the reactor body, object B, then the head of the reactor (object C) was placed on top and sealed tightly. The reactor was purged with H_2 three times and then pressurized with 30 bar H_2. The autoclave was left in a preheated oil bath at 50°C for 24 hours. Then, the autoclave was removed and left to cool to room temperature before carefully venting off H_2 gas. 1 mmol cyclohexene was added as the internal standard. The characterization data for formanilide matches well with the literature.[1]

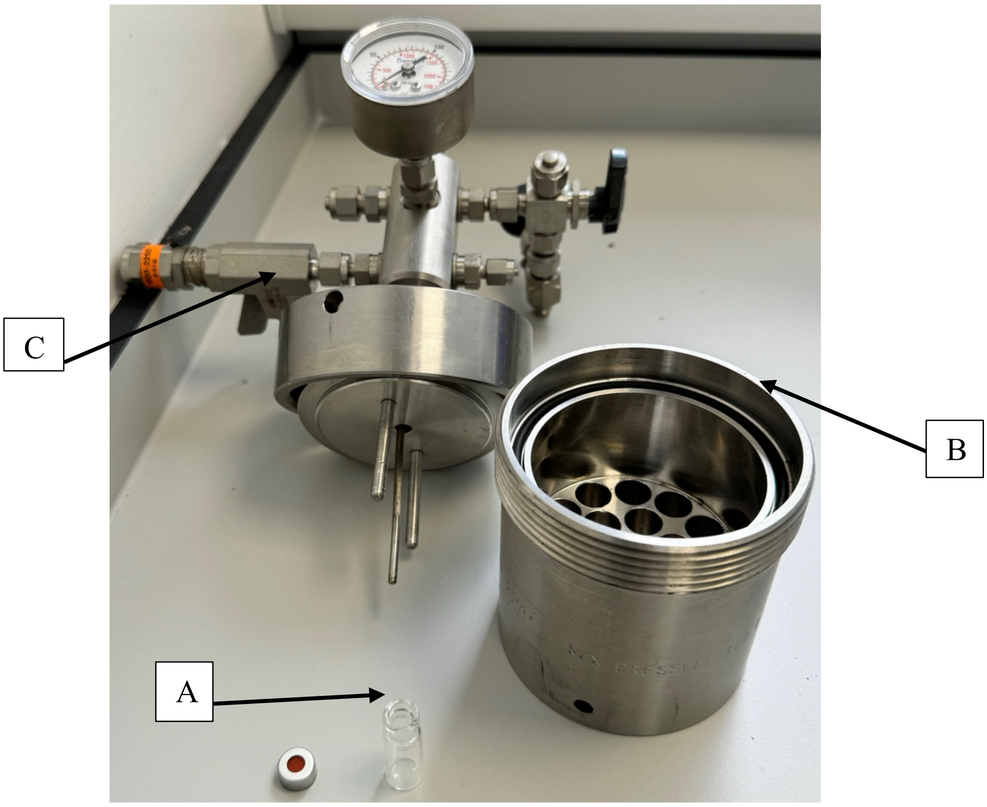

Figure 2: Image of the 16-vial autoclave used in the hydrogenation of isocyanates.

2.2 Analytical data for the hydrogenation of isocyanates

Table S1: Optimization of the catalytic conditions for the hydrogenation of phenyl isocyanate

Entry	Catalyst (2.4 mol%)	Base (10 mol%)	Yield
**1.**	Pd/C	-	0%
**2.**	Pd/C	NEt_3	83%
**3.**	Pd/C	KO^tBu	81%
**4.**	Pd/Al_2O_3	-	64%
**5.**	Pd/Al_2O_3	NEt_3	75%
**6.**	Pd/Al_2O_3	K_2CO_3	88%
**7.**	-	-	0%

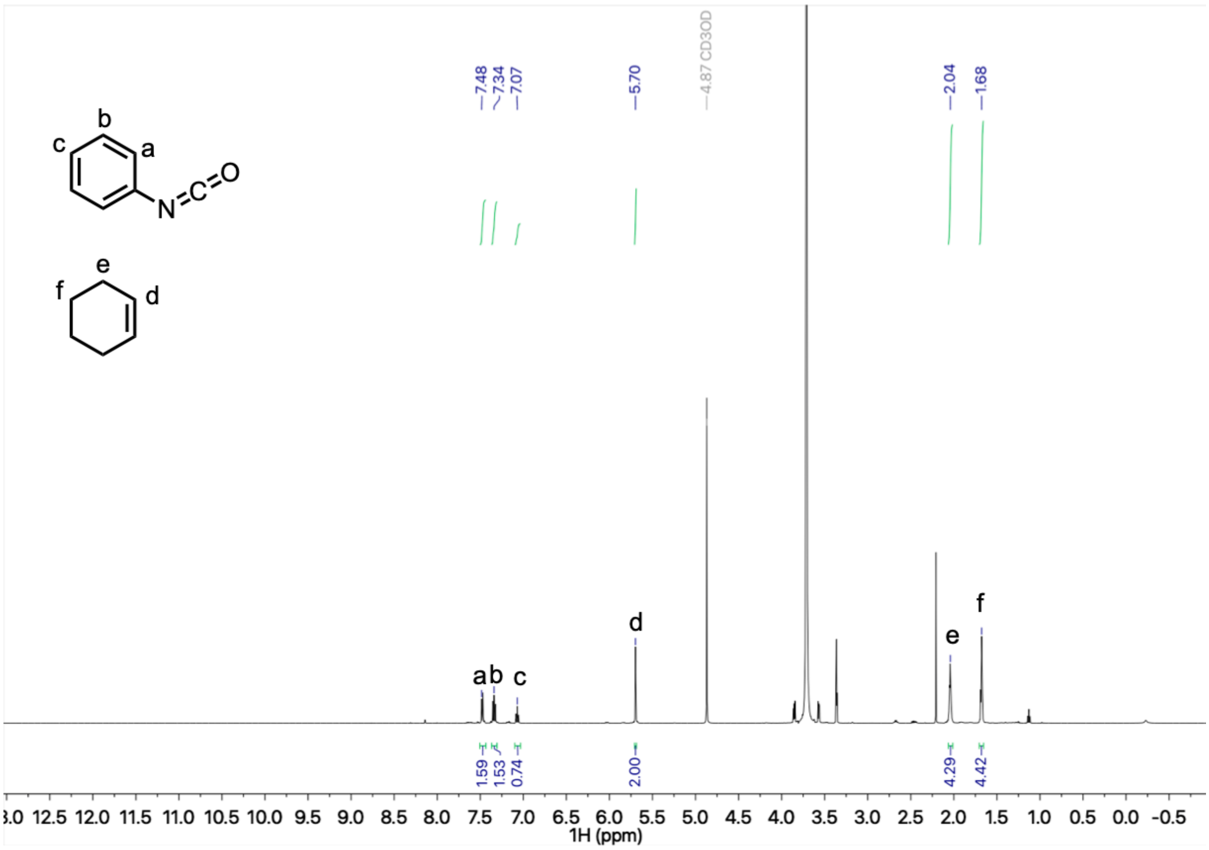

Figure 3: ^{1}H NMR (500 MHz, MeOD) spectrum of entry 1.

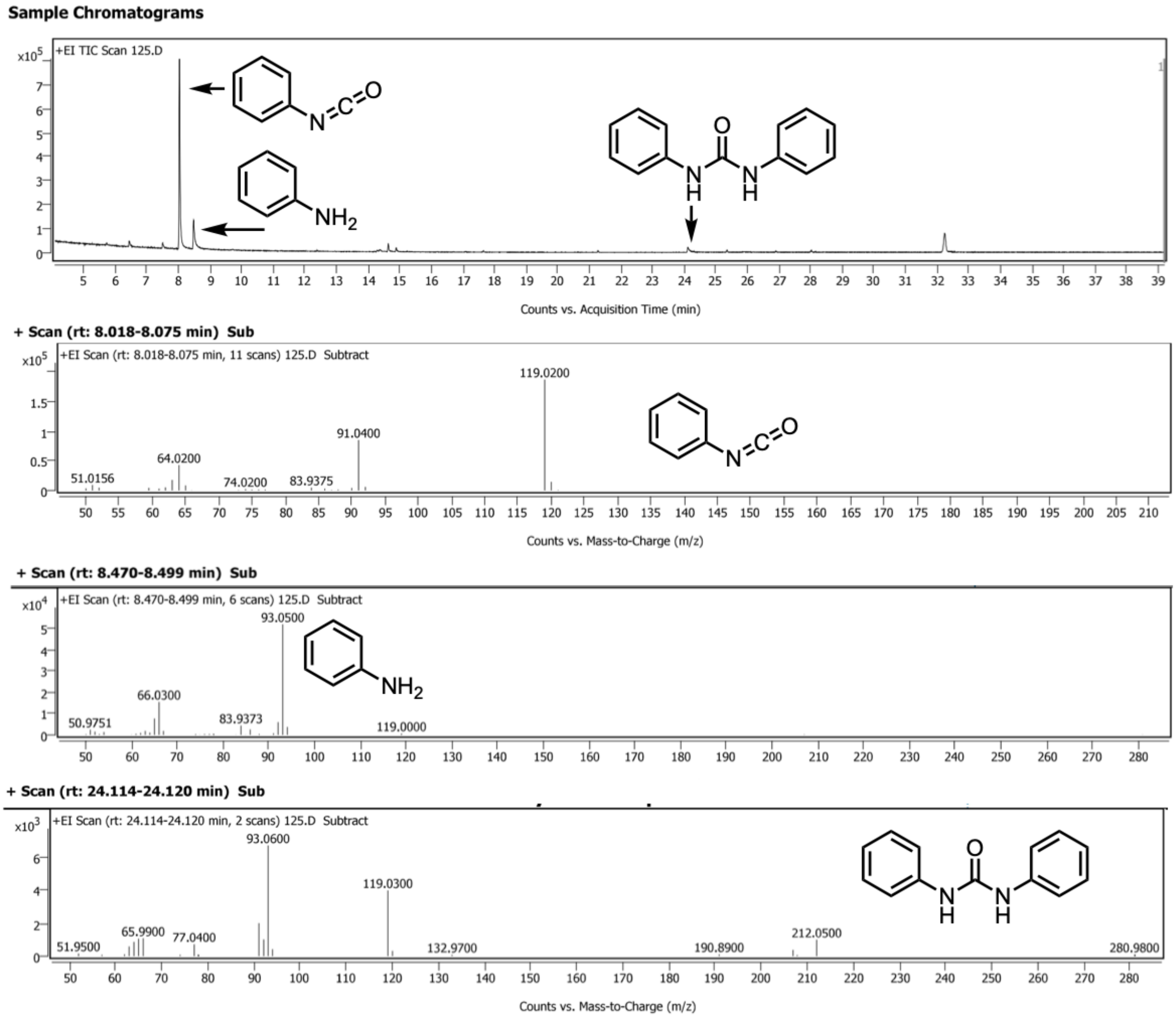

Figure 4: GC-MS data of entry 1 (Table S1). Trace aniline and diphenylurea observed. Other peaks in the chromatogram are column or column impurities.

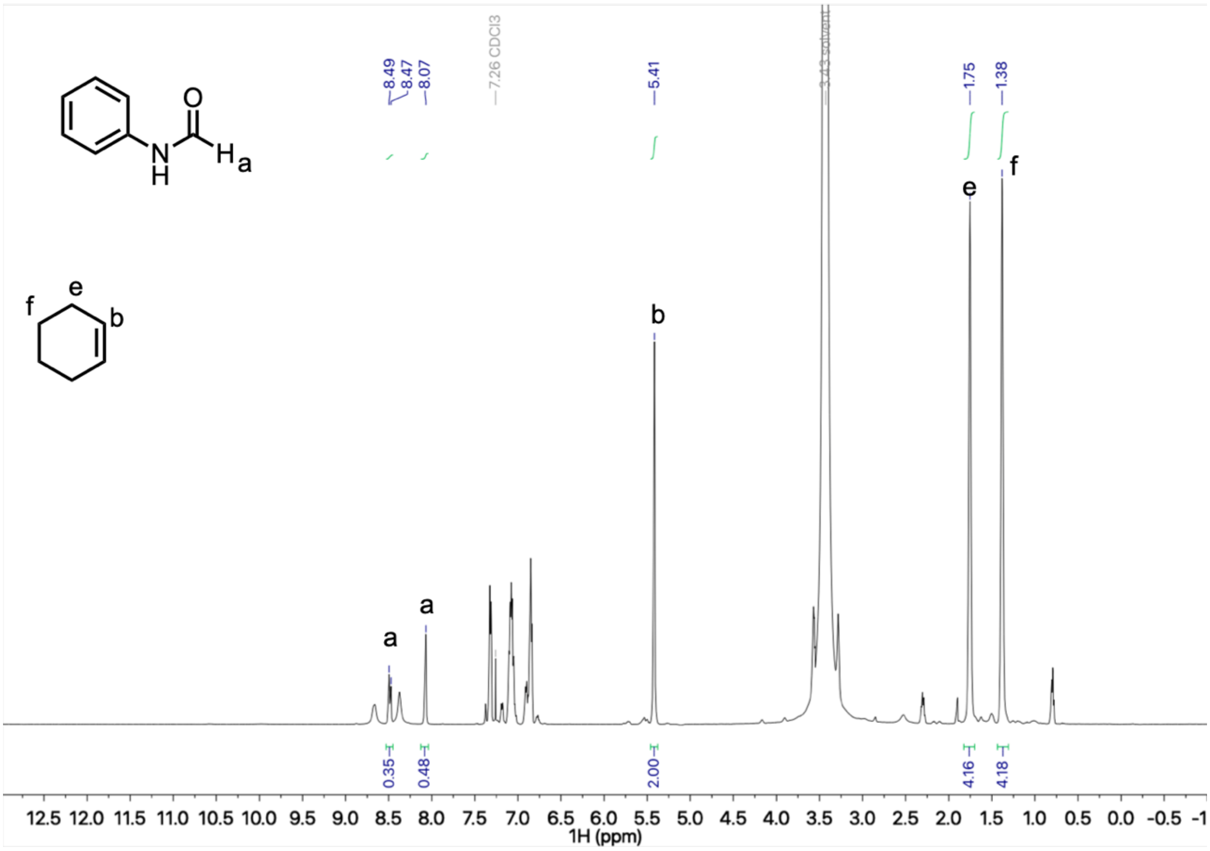

Figure 5: ^{1}H NMR ($CDCl_3$, 500 MHz) spectrum of entry 2 (Table S1).

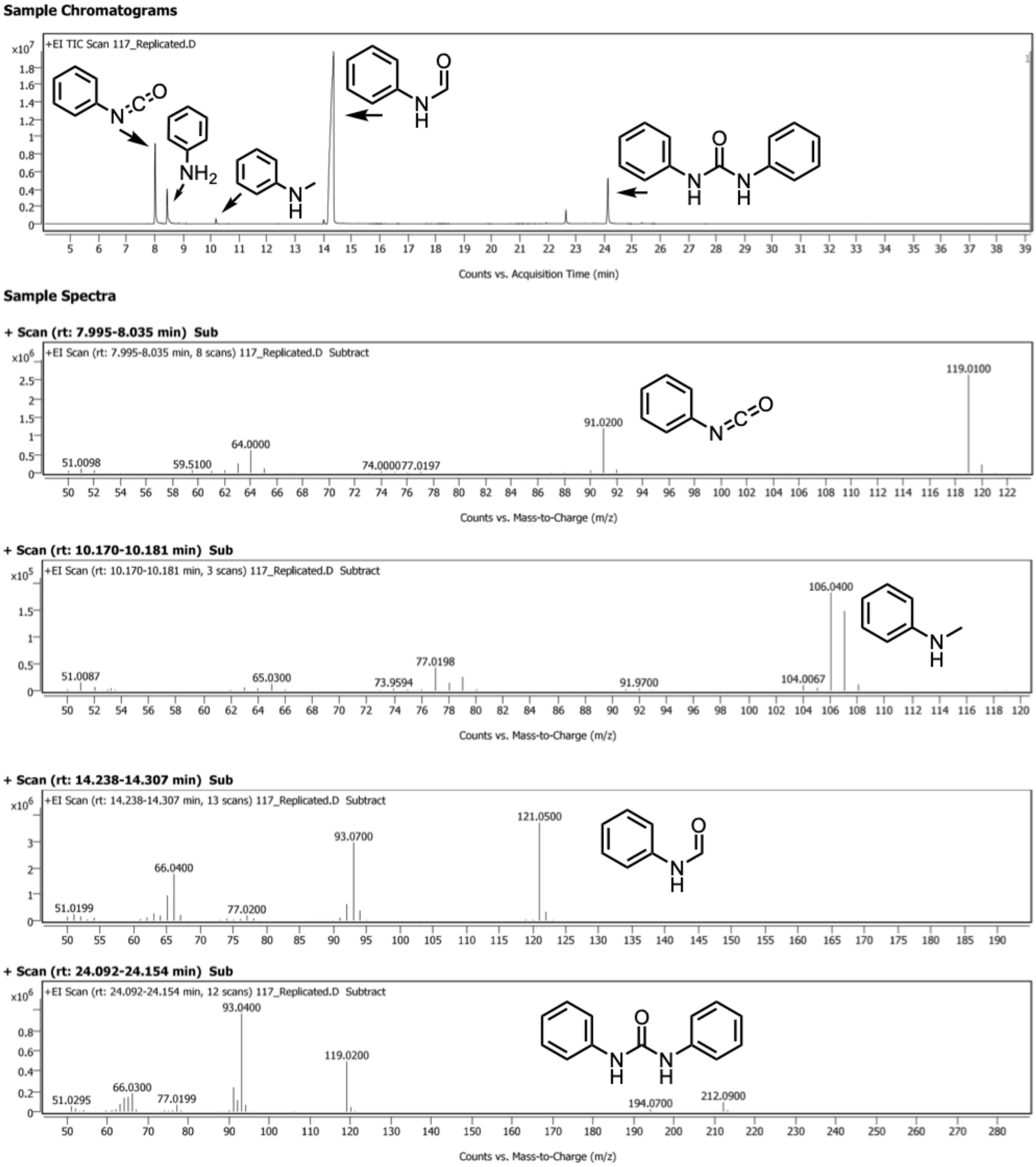

Figure 6: GC-MS data of entry 2 (Table S1).

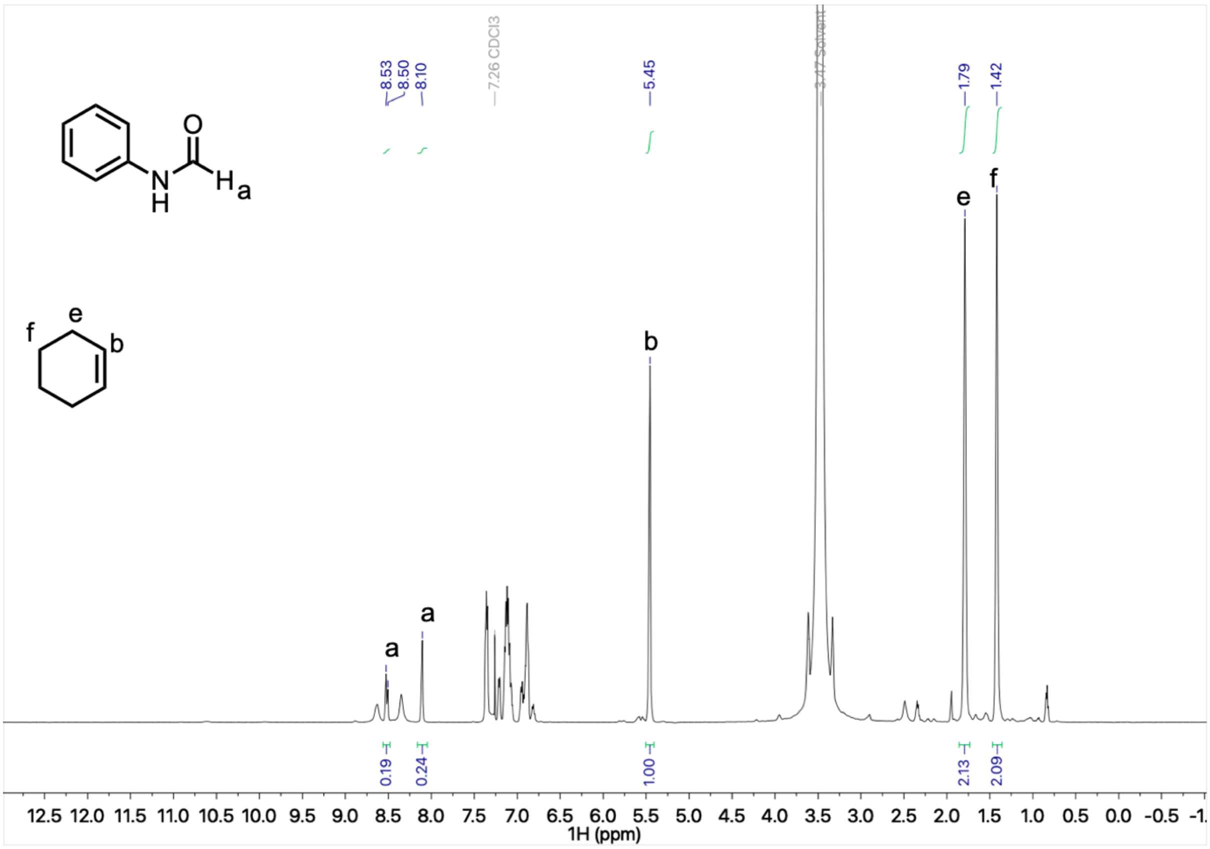

Figure 7: ^{1}H NMR ($CDCl_3$, 500 MHz) spectrum of entry 3 (Table S1).

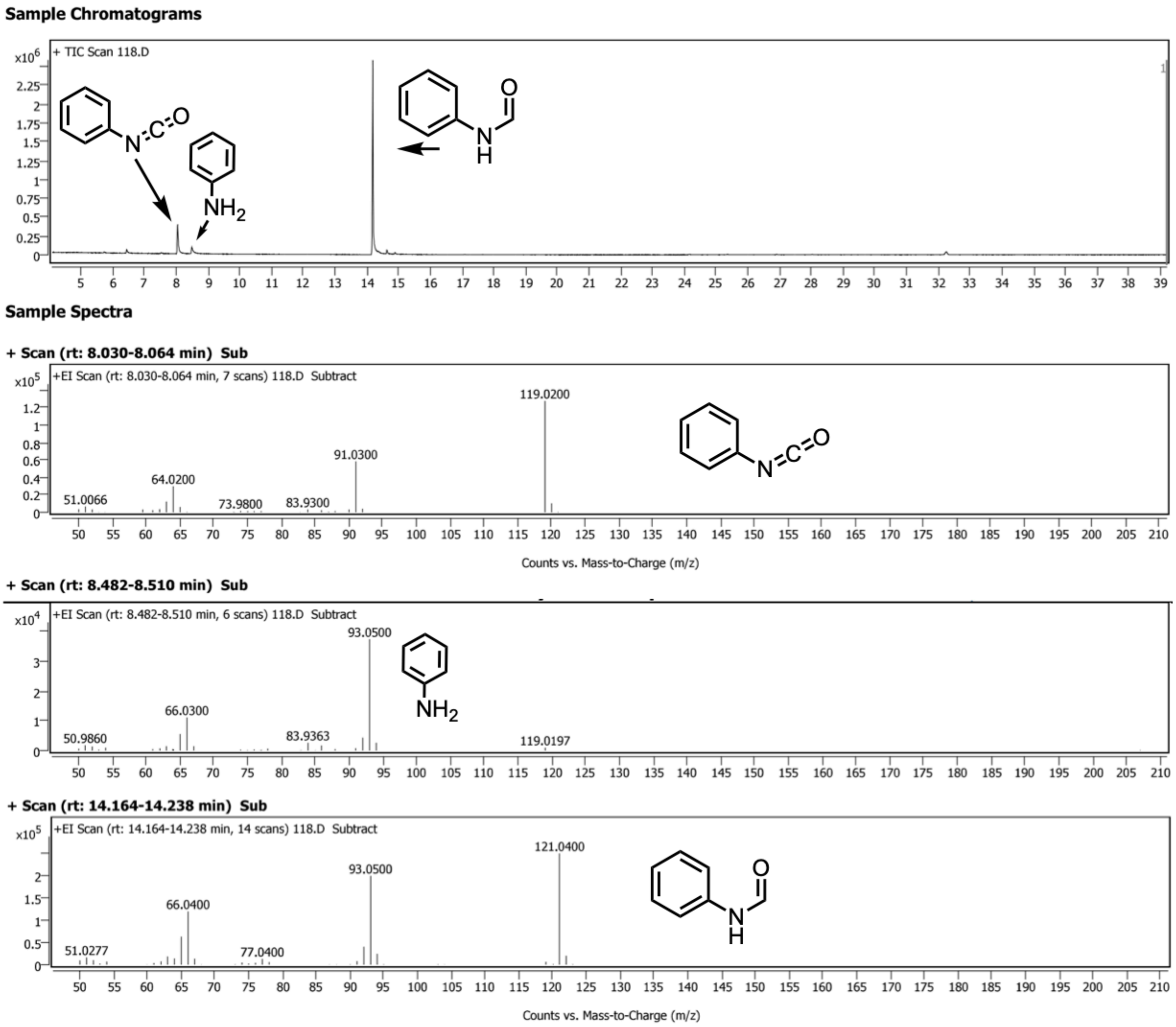

Figure 8: GC-MS data of entry 3 (Table S1).

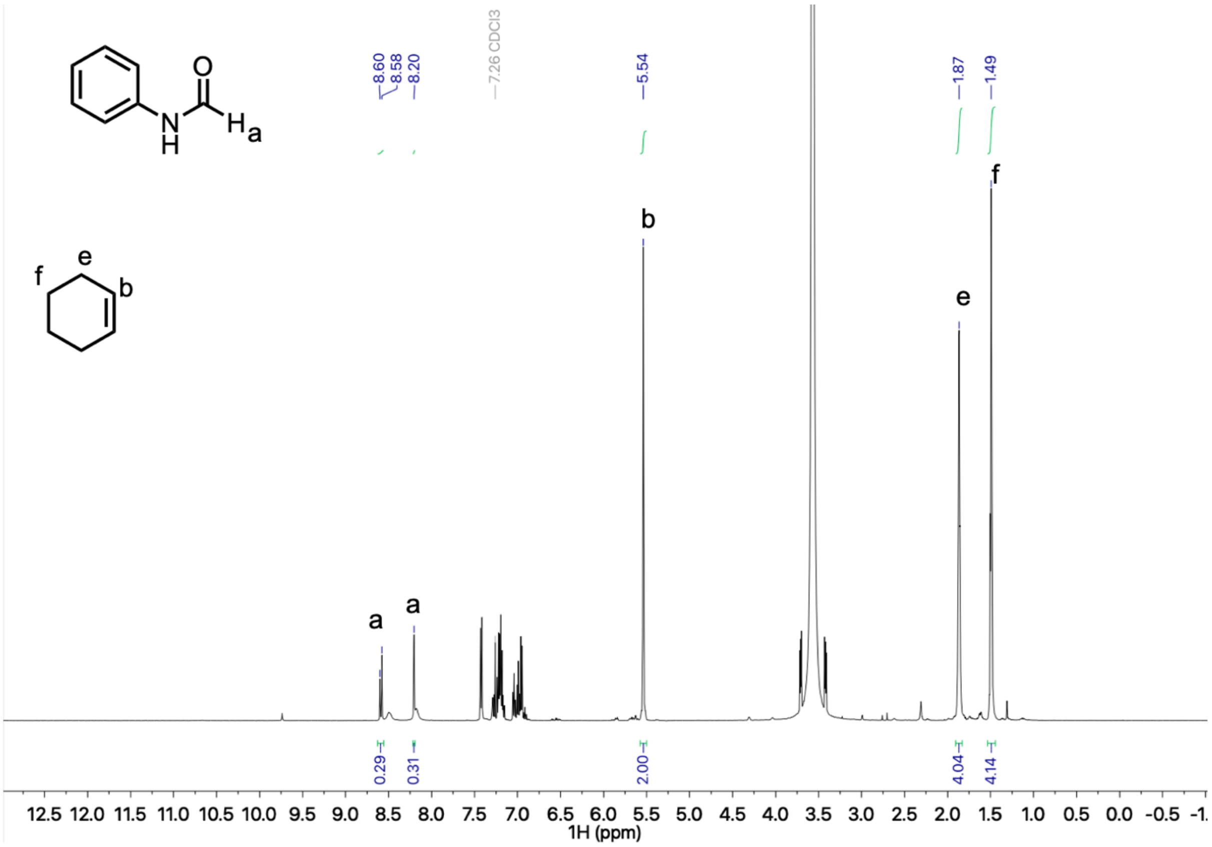

Figure 9: ^{1}H NMR ($CDCl_3$, 500 MHz) spectrum of entry 4 (Table S1).

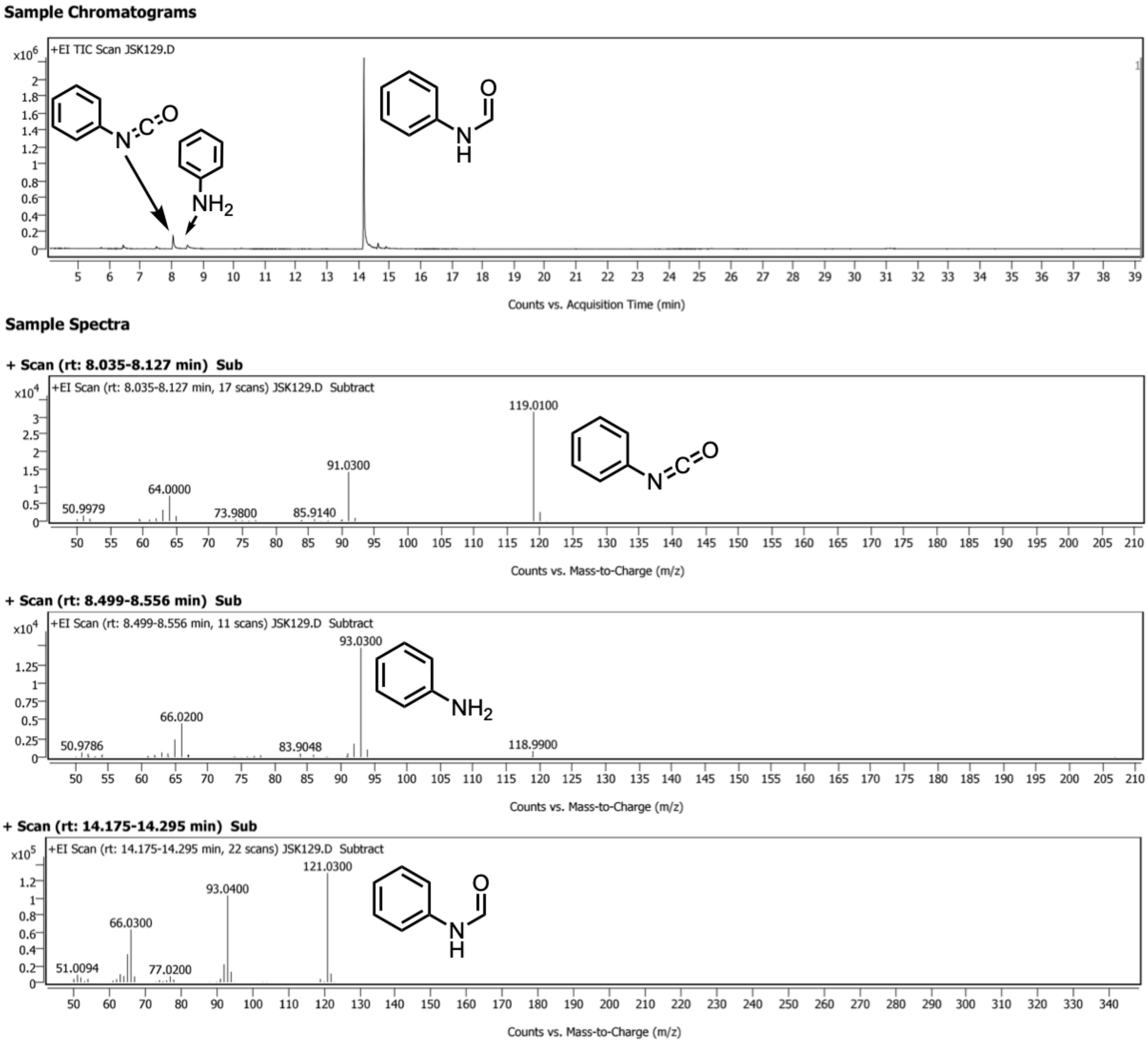

Figure 10: GC-MS data of entry 4 (Table S1).

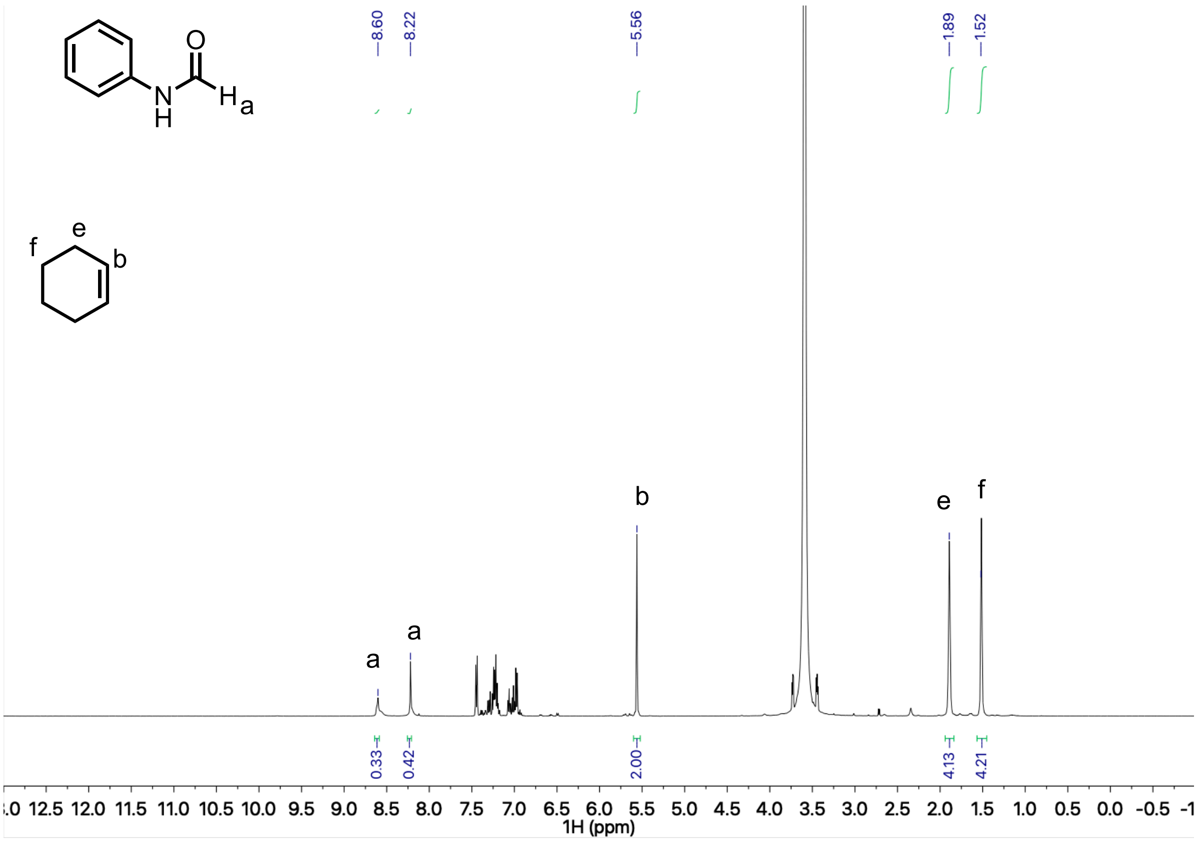

Figure 11: ^{1}H NMR ($CDCl_3$, 500 MHz) spectrum of entry 5 (Table S1).

Sample Chromatograms

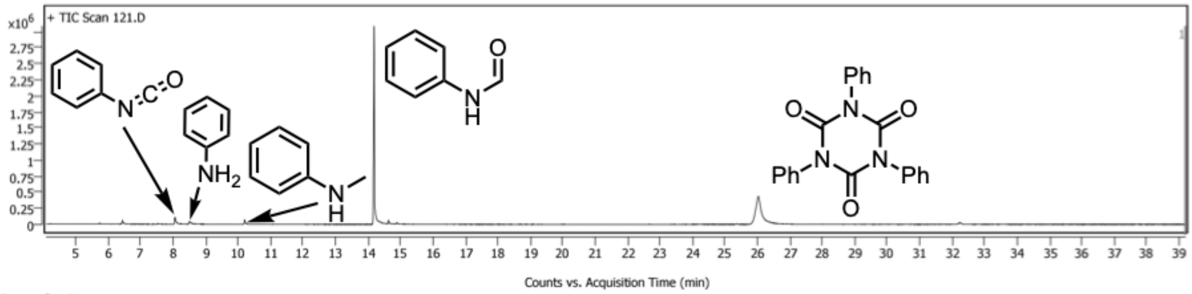

Sample Spectra

+ Scan (rt: 8.041-8.070 min) Sub

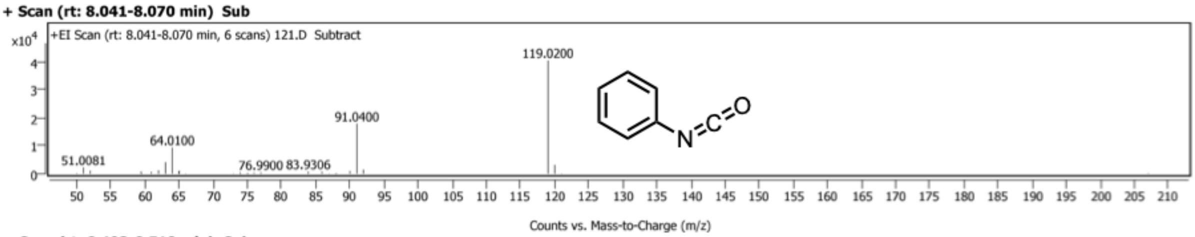

+ Scan (rt: 8.493-8.510 min) Sub

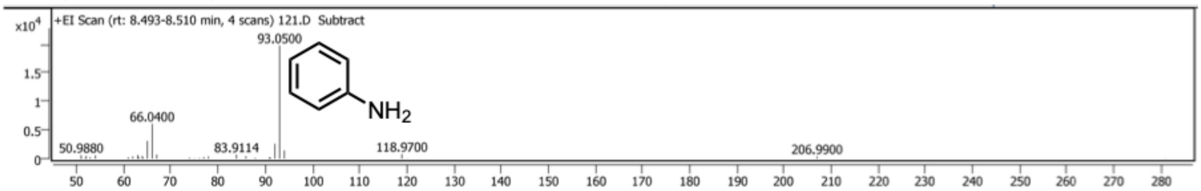

+ Scan (rt: 10.193-10.210 min) Sub

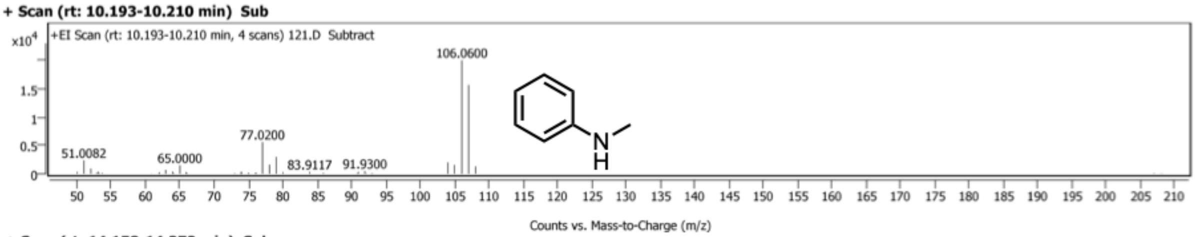

+ Scan (rt: 14.158-14.272 min) Sub

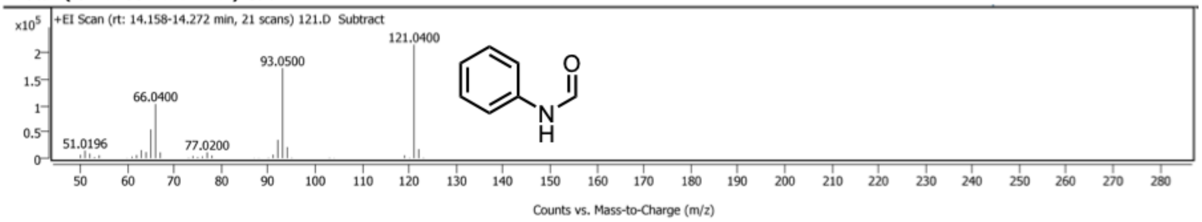

+ Scan (rt: 25.963-26.071 min) Sub

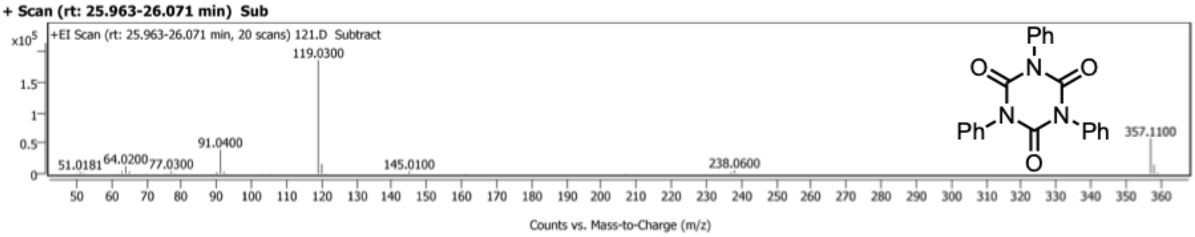

Figure 12: GC-MS data corresponding to entry 5 (Table S1).

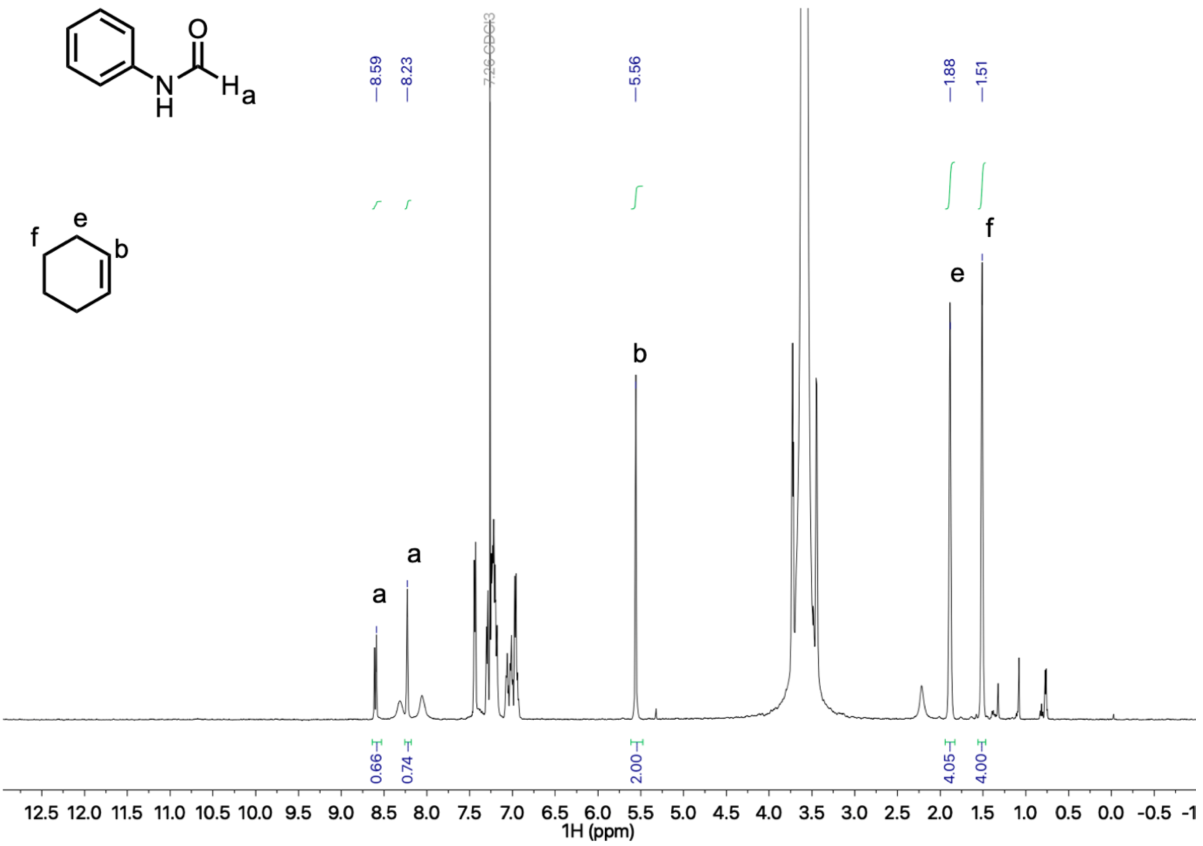

Figure 13: ^{1}H NMR ($CDCl_3$, 500 MHz) spectrum of entry 6 (Table S1).

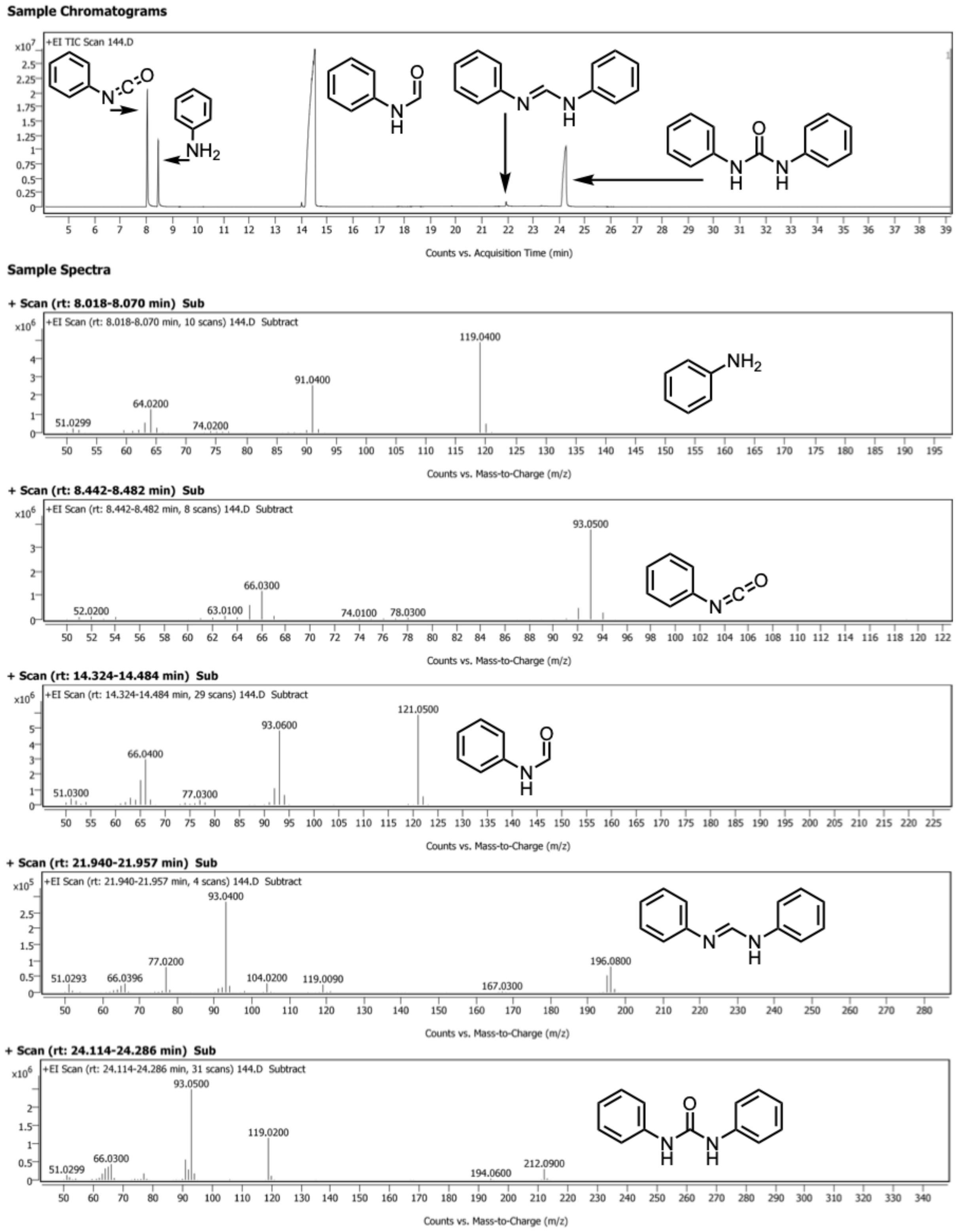

Figure 14: GC-MS data of entry 6 (Table S1).

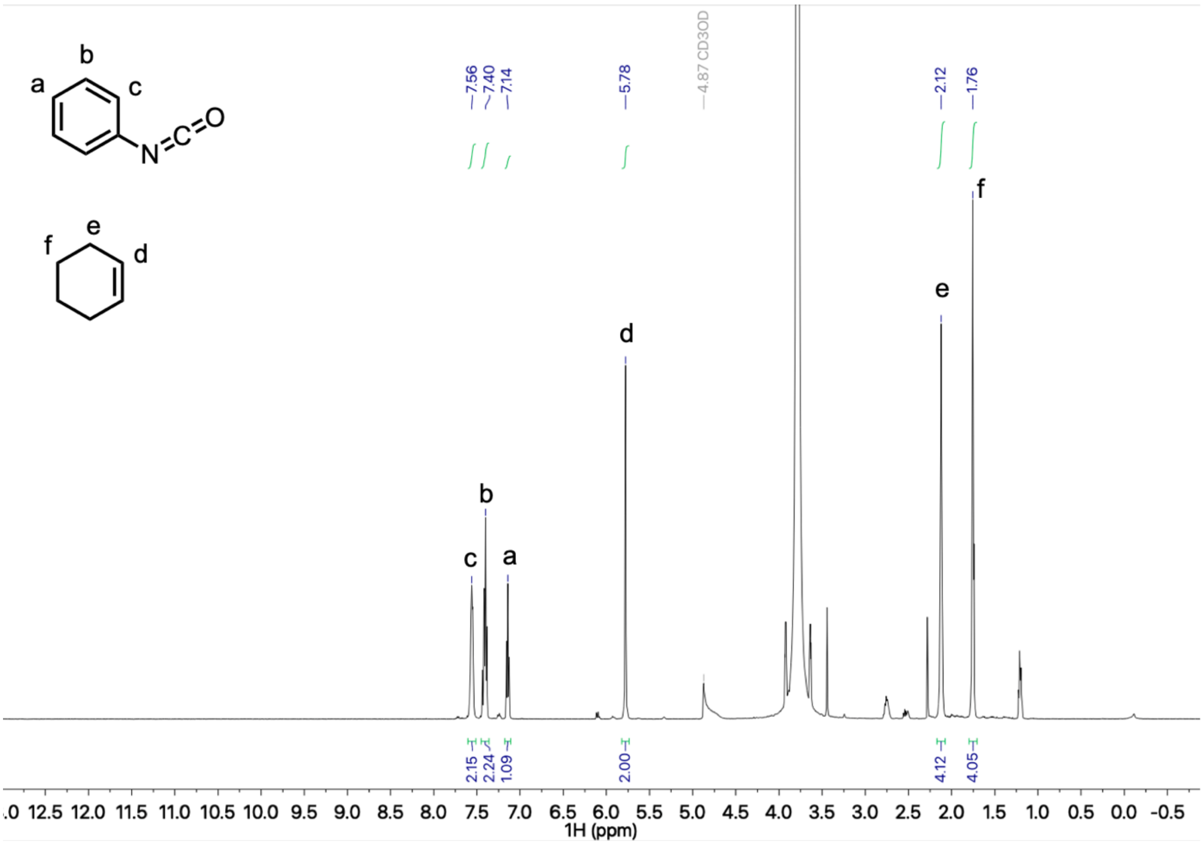

Figure 15: ^{1}H NMR (MeOD, 500 MHz) spectrum of entry 7 (Table S1).

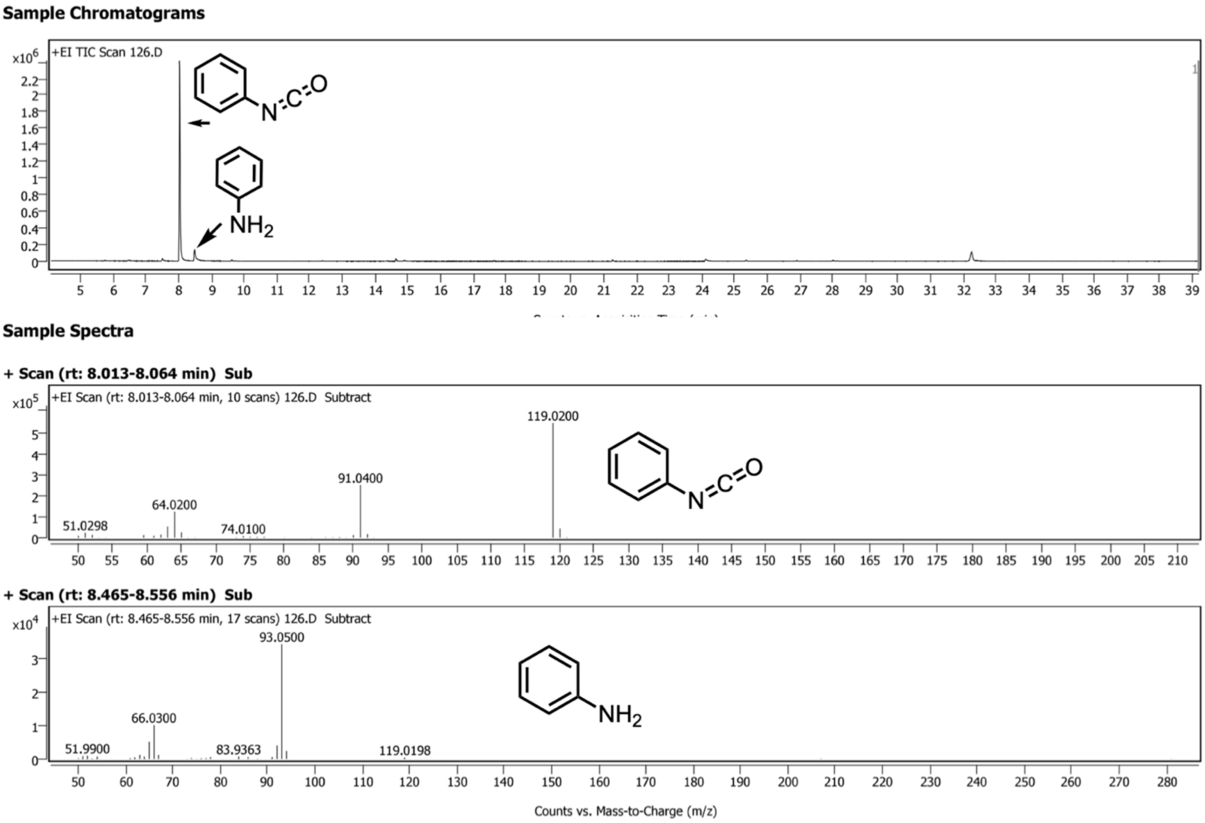

Figure 16: GC-MS data of entry 7.

3. Phenyl *N*-octylcarbamate hydrogenation optimization

3.1 Synthesis of phenyl *N*-octylcarbamate

$NaHCO_3$ (2 g) and *N*-octylamine (3.3 mL, 20.0 mmol) were added to distilled water (40 mL) in an ice bath. Phenyl chloroformate (3.233 g, 20.6 mmol) in THF (30 mL) was added slowly to the aqueous solution. The reaction was allowed to mix for 8 minutes and then ethyl acetate (220 mL) was added to the reaction mixture. The aqueous layer was separated, and the organic layer was washed with distilled water (3 x 50 ml), aq. HCl 5 wt% (3 x 50 mL) and then distilled water again (3 x 80 mL). The organic layer was dried with $MgSO_4$ and solvent was removed under reduced pressure to obtain the crude product. The product was recrystallized in minimal hexane to afford phenyl *N*-octylcarbamate (4.7280 g, 94.7%). The characterization data for phenyl *N*-octylcarbamate matches well with the literature.[2]

3.2 Characterization of phenyl *N*-octylcarbamate

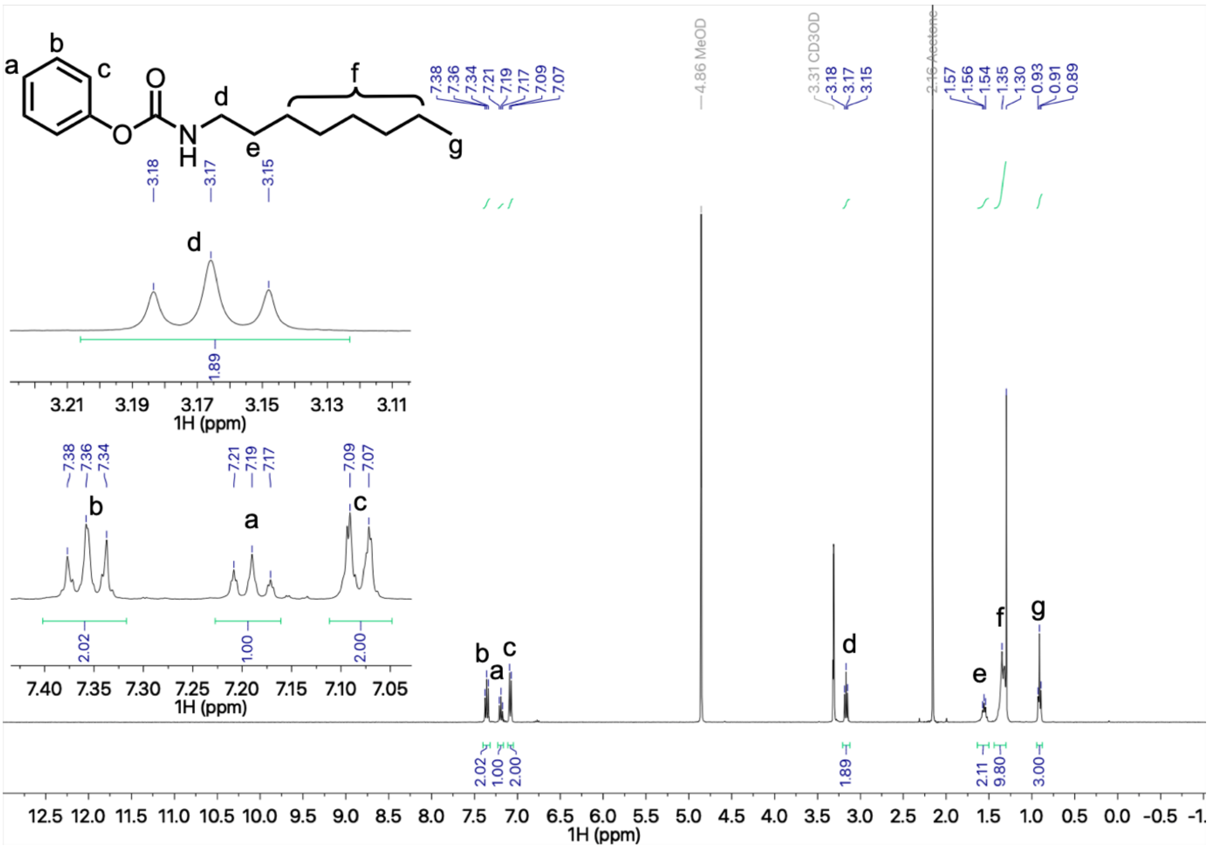

Figure 17: ¹H NMR (d⁴-MeOD, 500 MHz) spectrum of phenyl N-octylcarbamate.

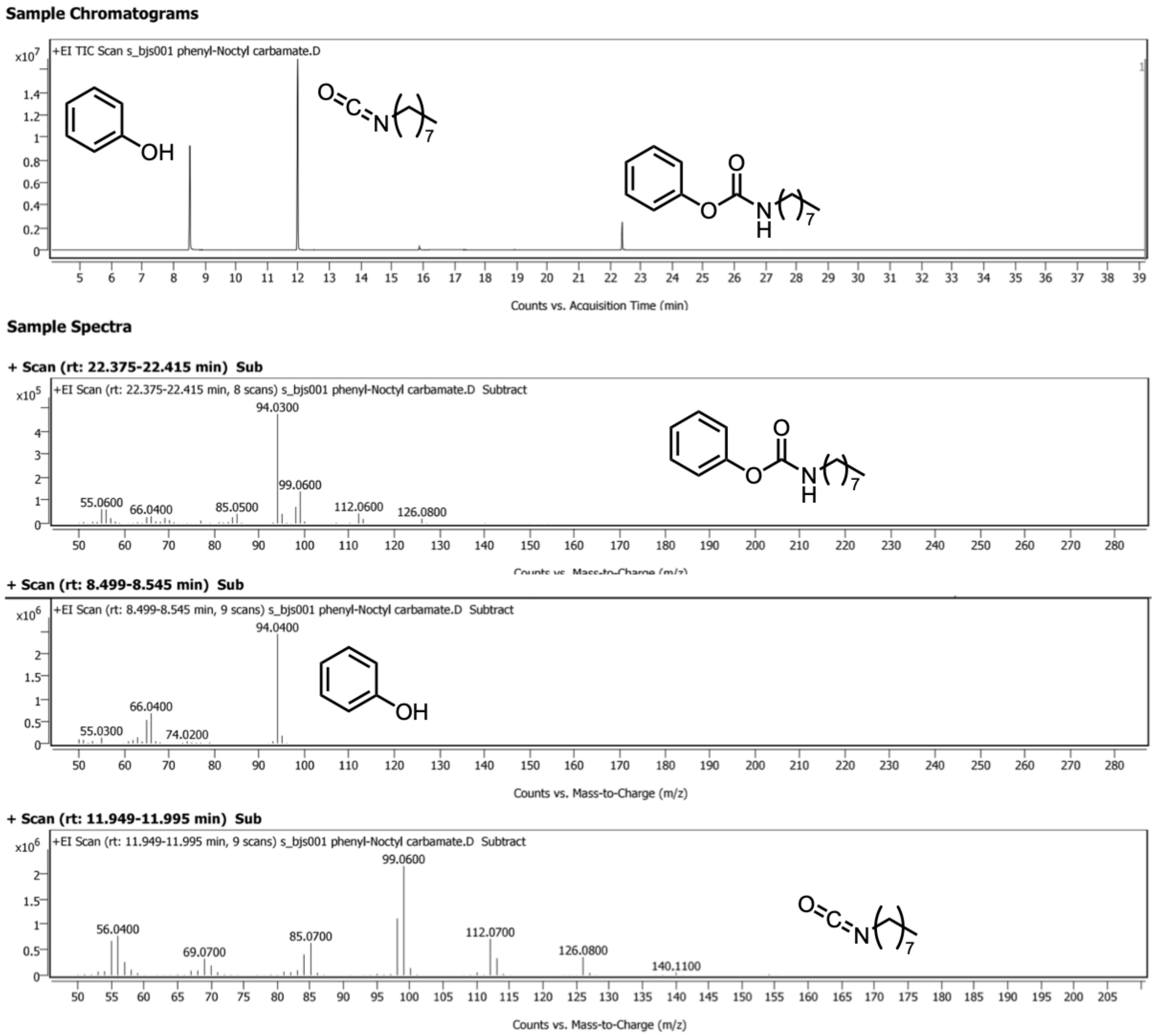

Figure 18: GC-MS of phenyl N-octylcarbamate. It dissociates into phenol and N-octylisocyanate in the GC.

3.3 Characterization of individual products from phenyl *N*-octylformamide hydrogenation

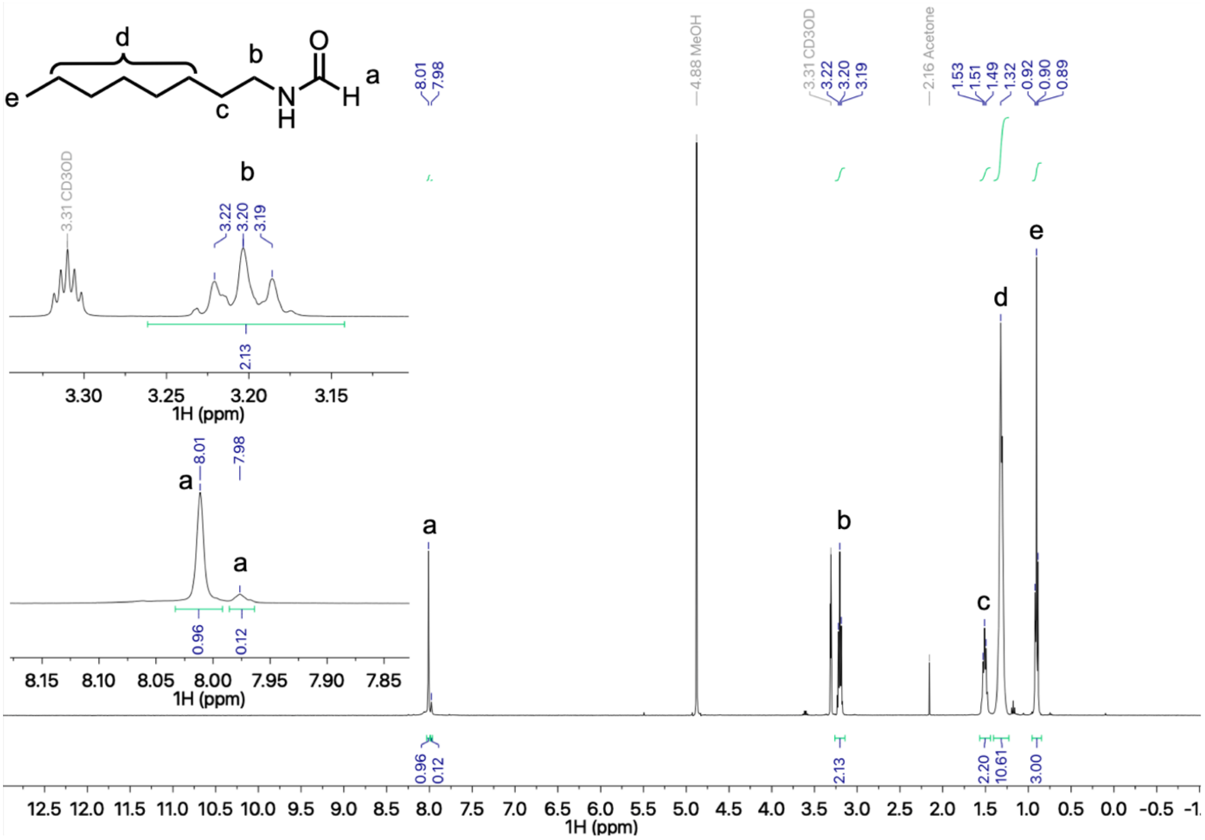

Figure 19: ^{1}H NMR (d^4-MeOD, 500 MHz) spectrum of octylformamide.

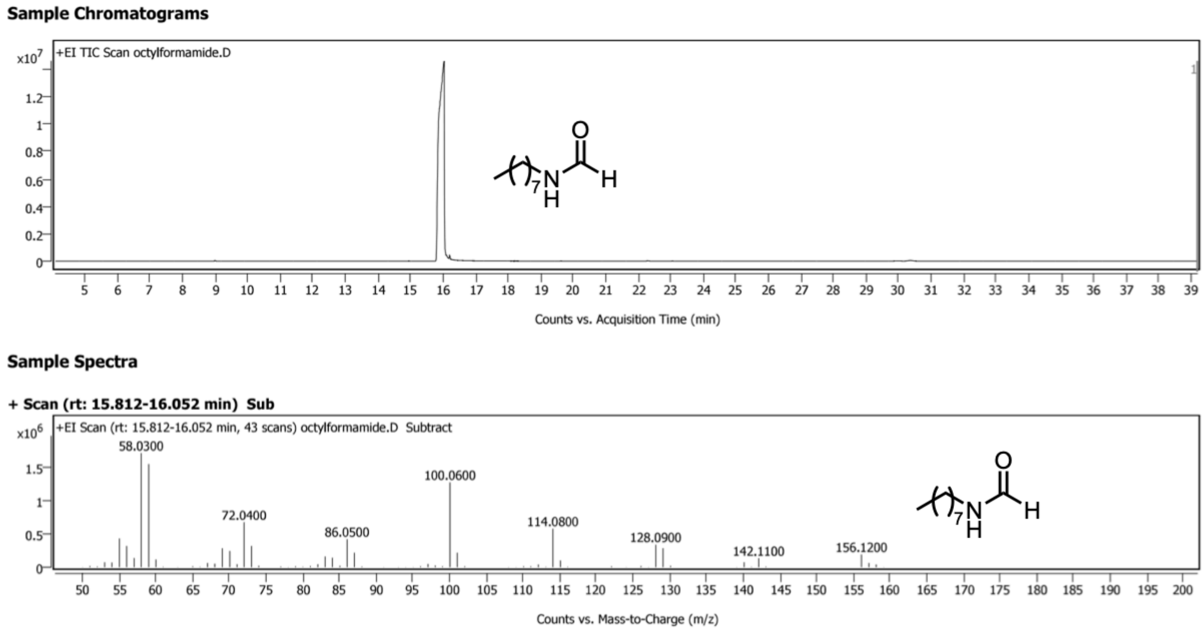

Figure 20: GC-MS of octylformamide.

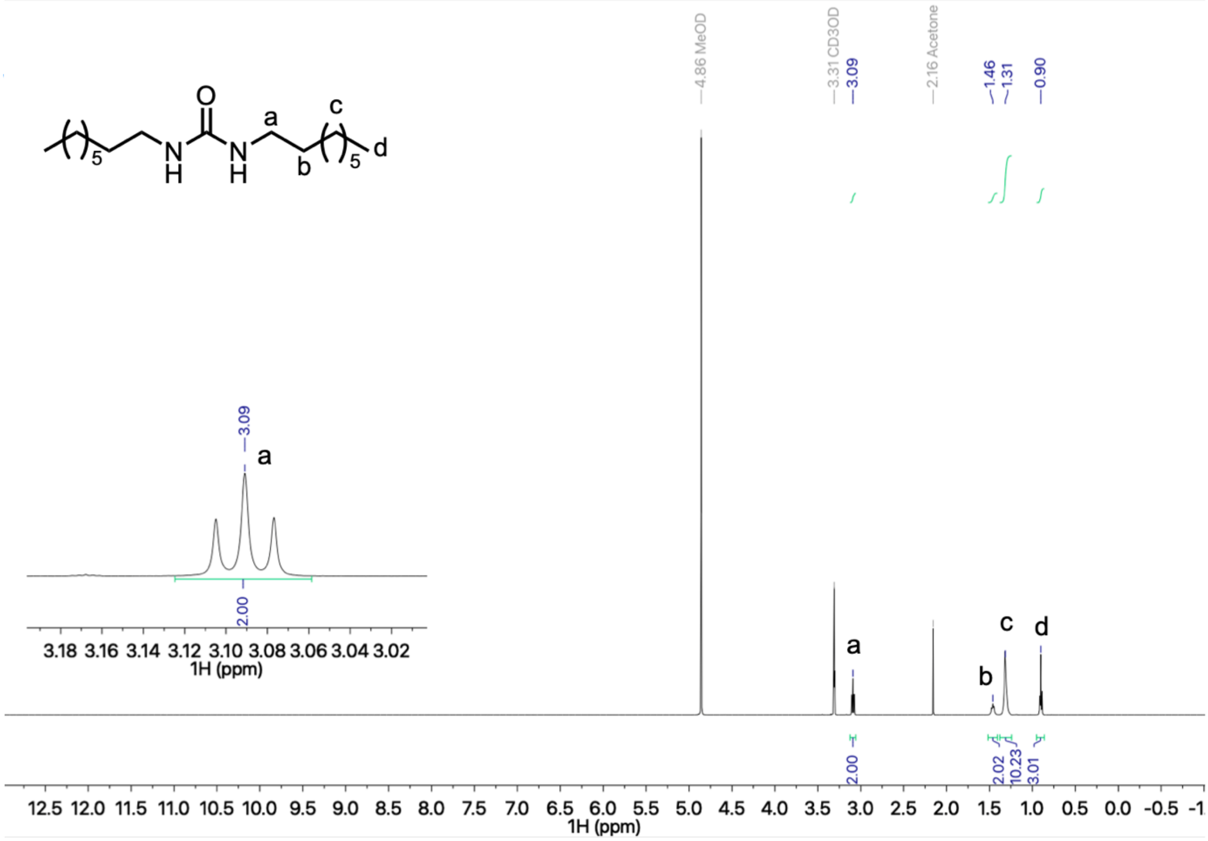

Figure 21: ^{1}H NMR (d[4]-MeOD, 500 MHz) spectrum of dioctylurea

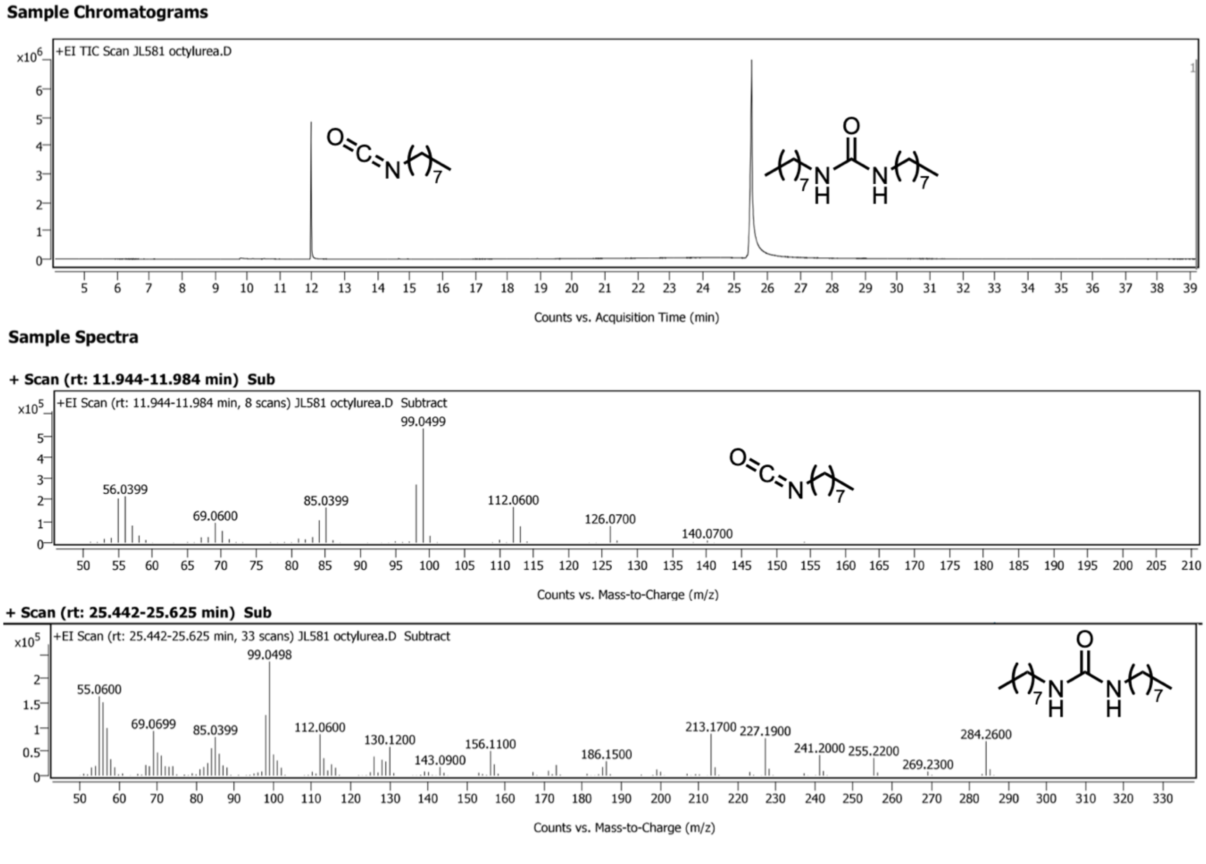

Figure 22: GC-MS of dioctylurea. Dissociates into isocyanate in the GC

3.4 Procedure for the hydrogenation of phenyl *N*-octylcarbamate

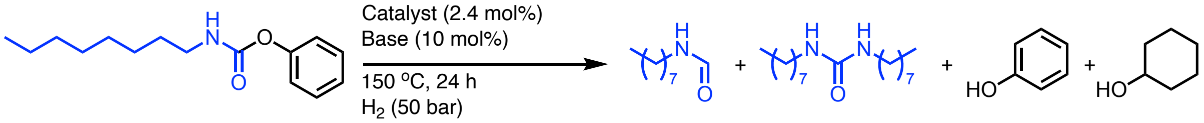

Figure 23: General scheme for the hydrogenation of phenyl N-octylcarbamate

Phenyl *N*-octylcarbamate (1 mmol), catalyst (2.4 mol%), and base (10 mol%) were weighed under air and transferred to a microwave vial (5 mL) containing a stirrer bar. The vial was sealed using a septum and degassed under argon by placing a needle through the septum. A dry solvent (1 mL) was transferred through a syringe to the vial. The septum was pierced with two needles and placed in a stainless-steel autoclave under an argon atmosphere. The autoclave was sealed and degassed three times using H_2 gas. Upon degassing, the autoclave was pressurized with 50 bar of H_2 gas. The autoclave was placed in a preheated oil bath at 150 ℃ and left for 24 hours. After completion of the reaction time, the autoclave was cooled in cold water for 30 minutes, and the hydrogen gas was slowly vented off. 0.25 mmol 1,1'-diphenylethylene was added as the internal standard. Yields of octylformamide are determined by ^{1}H NMR from formamide peaks at 7.98 and 8.01 ppm, being the different isomers. Yields of dioctylurea are determined by ^{1}H NMR spectroscopy by integrating the signal at δ 3.09 ppm relative to the internal standard (1,1'-diphenylethylene). Dioctyl urea has a theoretical yield of 0.5 mmol. Yields of dioctylurea are determined by ^{1}H NMR at 3.09 ppm and has a theoretical yield of 0.5 mmol. Yields of phenol are determined by integrating the two aromatic signals at δ 6.77 and 7.08 ppm. Conversion of phenyl *N*-octylcarbamate is determined by the absence of its corresponding peaks (see figure 17) by ^{1}H NMR and the absence of it by GC-MS. The characterisation data for octyl formamide,[3] and dioctylurea,[4] matches well with the literature.

3.5 Analytical data for the hydrogenation of phenyl *N*-octylcarbamate

Table S2: Optimization for catalytic hydrogenation of phenyl N-octylcarbamate.

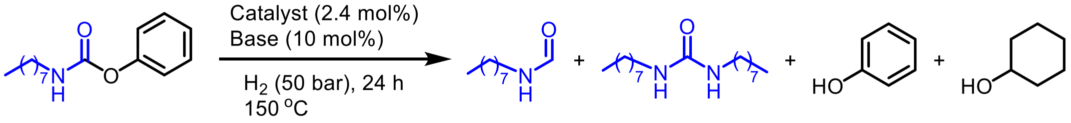

Entry	Catalyst	Solvent	Base	Conversion (%)	Formamide Yield (%)	Urea Yield (%)	Phenol Yield (%)	Cyclohexanol Yield (%)
1	Pd/Al_2O_3	1,4-dioxane	K_2CO_3	100	43	57	92	0
2	Pd/Al_2O_3	1,4-dioxane	Et_3N	98	51	26	69	6
3	Pd/Al_2O_3	toluene	K_2CO_3	100	51	41	76	14
4	Pd/Al_2O_3	toluene	Et_3N	100	40	21	64	0
5	Pd/Al_2O_3	anisole	K_2CO_3	100	42	37	76	0
6	Pd/Al_2O_3	anisole	Et_3N	100	43	11	64	0
7	Pd/Al_2O_3	THF	K_2CO_3	100	54	17	58	4
8	Pd/Al_2O_3	THF	Et_3N	100	50	18	54	0
9	Pd/Al_2O_3	*t*-amyl OH	K_2CO_3	100	71	17	87	4
10	Pd/Al_2O_3	*t*-amyl OH	Et_3N	100	60	14	68	0
11	Pd/Al_2O_3	*t*-amyl OH	Cs_2CO_3	100	45	30	57	0
12	Pd/Al_2O_3	*t*-amyl OH	KBH_4	100	83	10	85	14
13[b]	Pd/Al_2O_3	*t*-amyl OH	KBH_4	100	14	32	99	0
14[b]	Pd/Al_2O_3	*t*-amyl OH	K_2CO_3	100	0	60	93	0
15	-	*t*-amyl OH	KBH_4	100	15	26	99	0
16[b]	-	*t*-amyl OH	KBH_4	100	19	23	99	0
17	Pd/C	*t*-amyl OH	KBH_4	100	75	7	65	34
18	Pt/C	*t*-amyl OH	KBH_4	100	16	51	90	0
19	Ru/Al_2O_3	*t*-amyl OH	KBH_4	100	18	39	93	0
20	$Ni/Si\text{-}Al_2O_3$	*t*-amyl OH	KBH_4	100	18	35	93	0
21	-	*t*-amyl OH	-	0	0	0	0	0

[a]Reaction conditions: phenyl *N*-octylcarbamate (1 mmol), catalyst (2.4 mol%), base (10 mol%), solvent 1 mL, H_2 (50 bar), 24 h, 150 °C. The products were identified by GC-MS, and the conversion and yields were estimated by ^1H NMR spectroscopy using 1,1'-diphenylethylene as an internal standard. Two other uncharacterised side products were obtained in each case with the m/z values in GC-MS of 174 and 211 Da. [b]No hydrogen pressure.

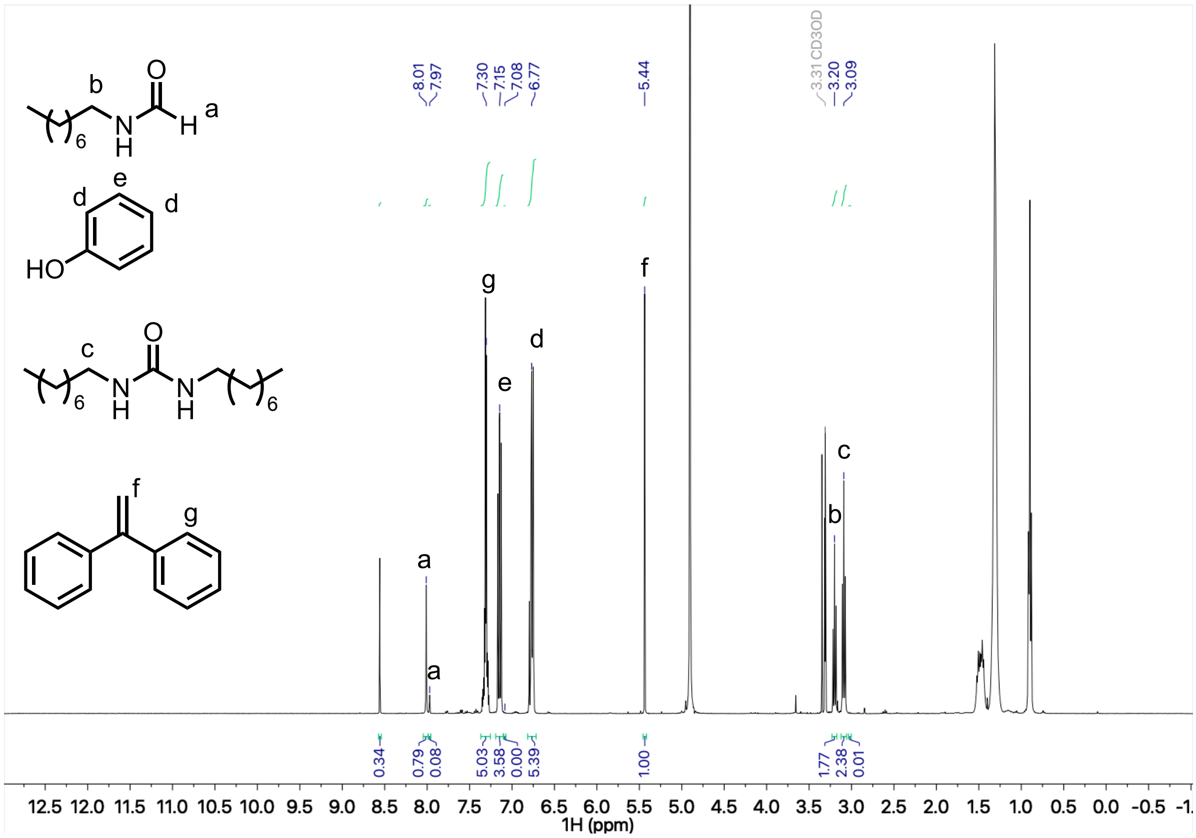

Figure 23: ^{1}H NMR (MeOD, 500 MHz) spectrum of entry 1 (Table S2).

Sample Chromatograms

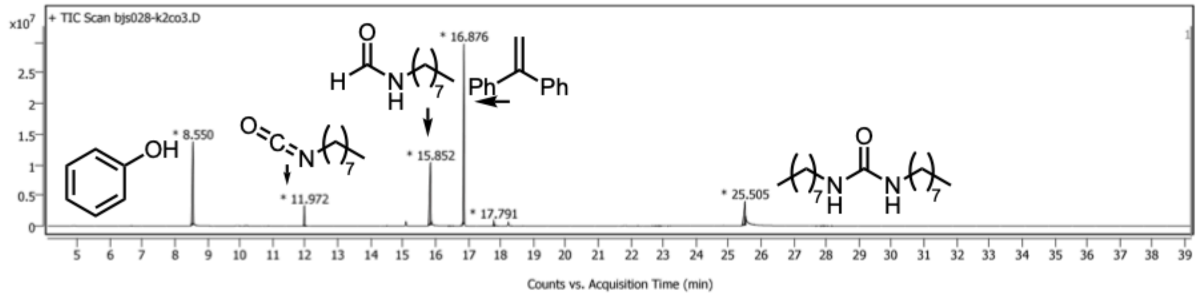

Sample Spectra

+ Scan (rt: 8.510-8.556 min) Sub

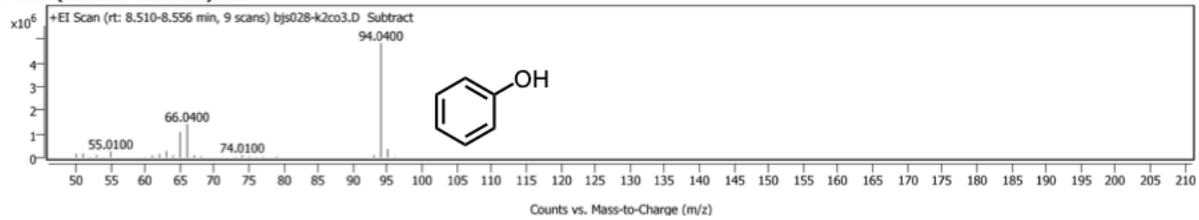

+ Scan (rt: 11.955-11.983 min) Sub

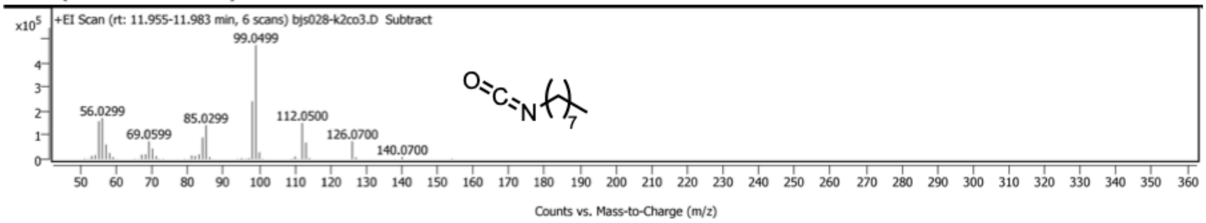

+ Scan (rt: 15.812-15.857 min) Sub

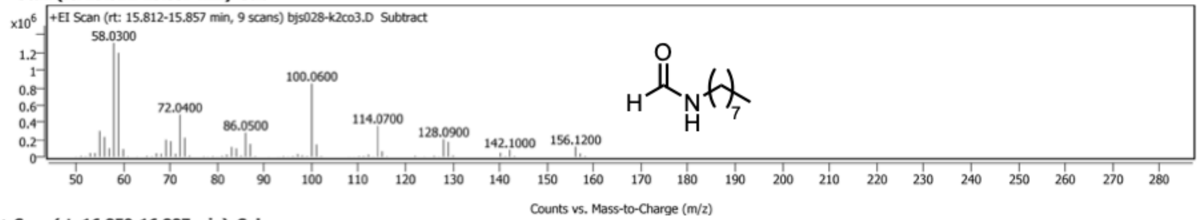

+ Scan (rt: 16.859-16.887 min) Sub

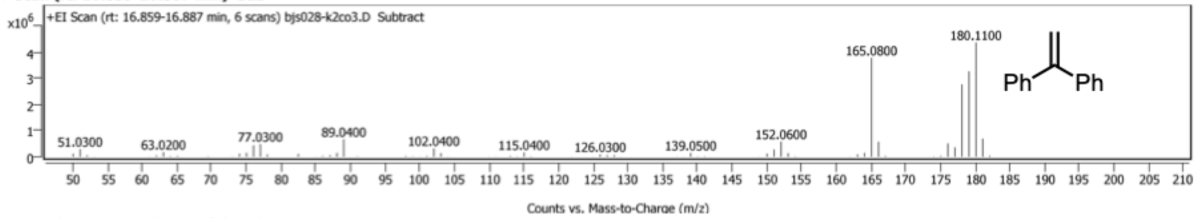

+ Scan (rt: 25.453-25.510 min) Sub

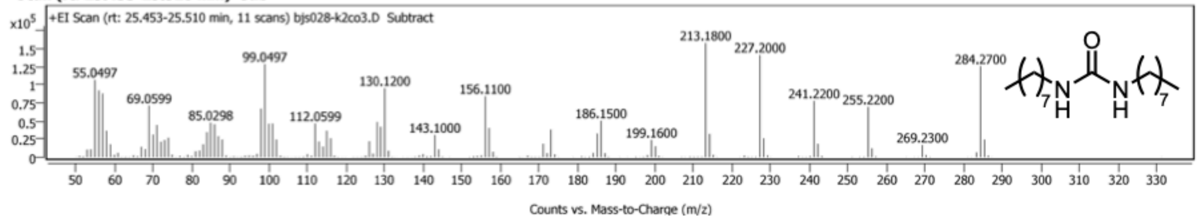

Figure 24: GC-MS data of entry 1 (Table S2).

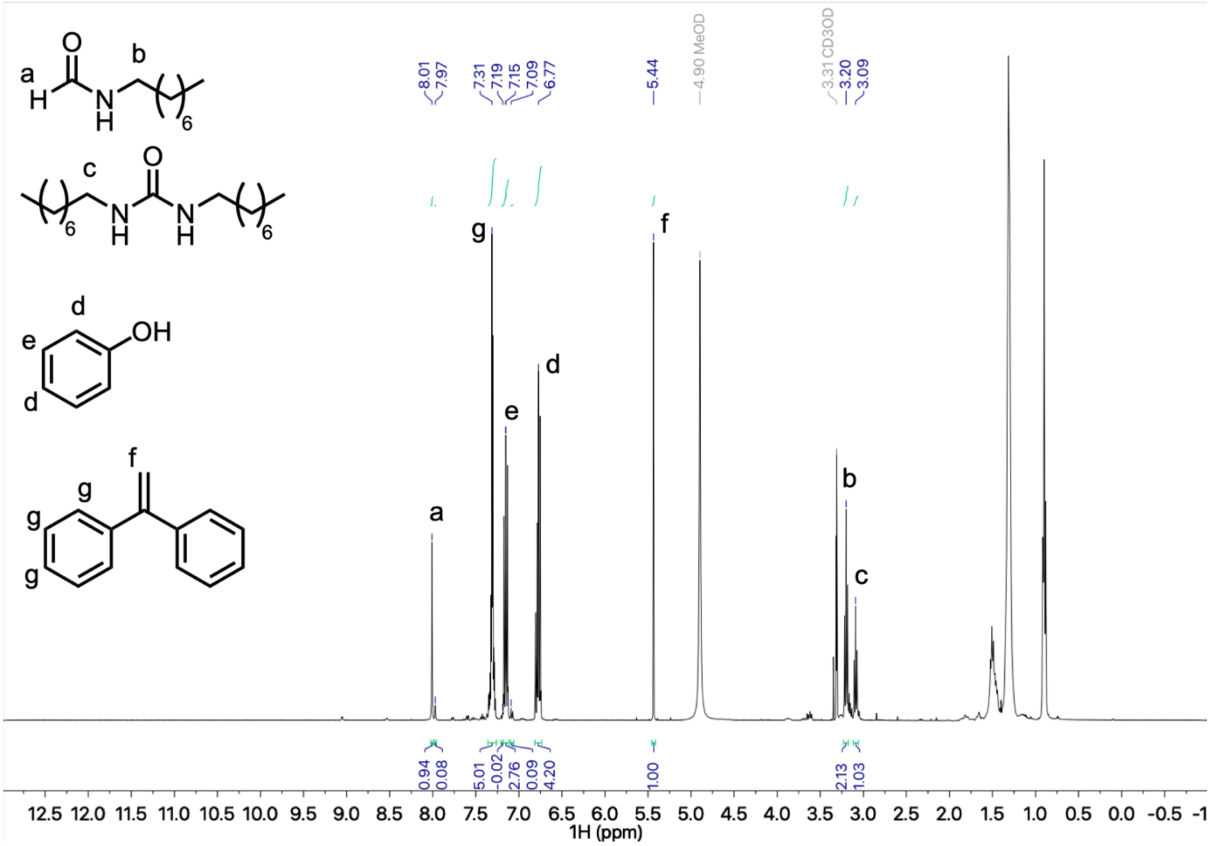

Figure 25: ^{1}H NMR (d[4]-MeOD, 500 MHz) spectrum of entry 2 (Table S2).

Sample Chromatograms

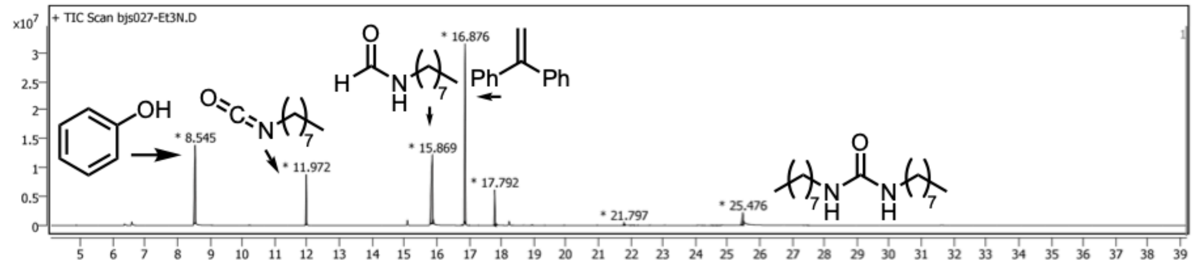

Sample Spectra

+ Scan (rt: 8.510-8.556 min) Sub

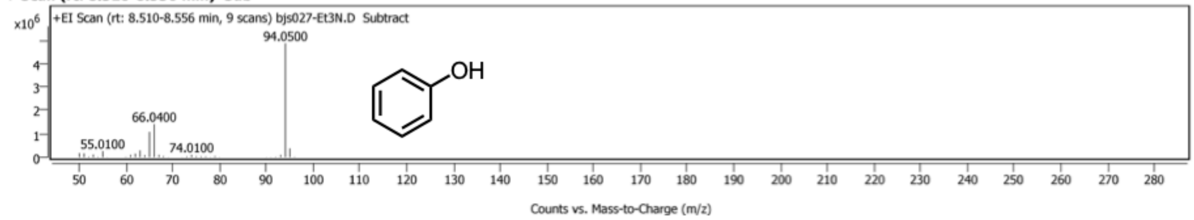

+ Scan (rt: 11.955-11.984 min) Sub

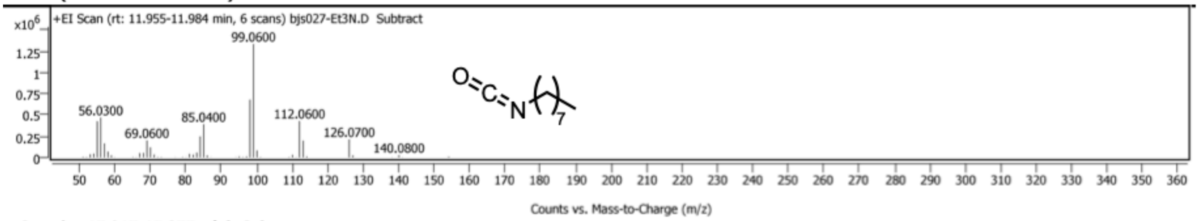

+ Scan (rt: 15.817-15.875 min) Sub

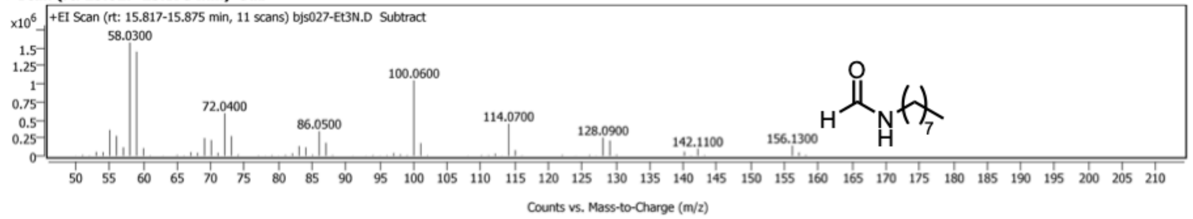

+ Scan (rt: 16.859-16.888 min) Sub

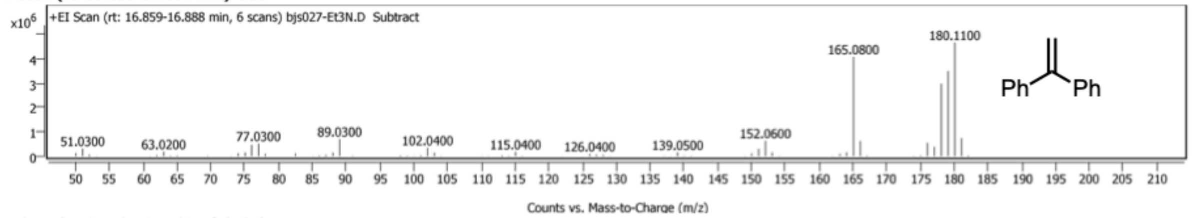

+ Scan (rt: 25.431-25.482 min) Sub

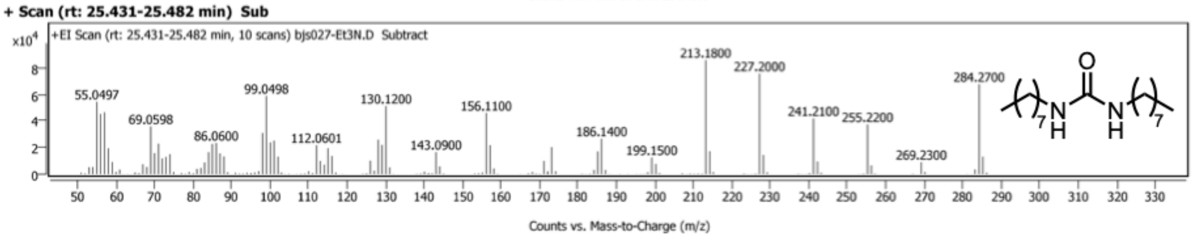

Figure 26: GC-MS of entry 2 (Table S2).

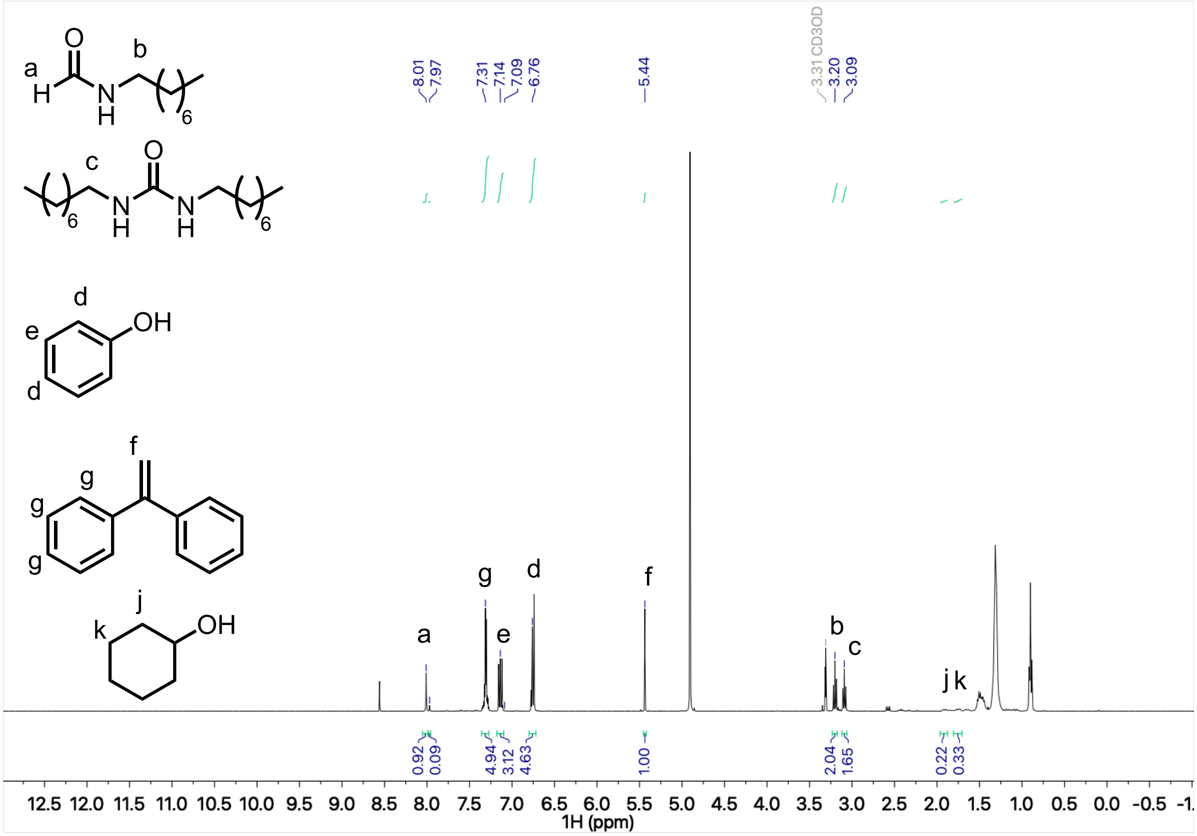

Figure 27: ^{1}H NMR (d^{4}-MeOD, 500 MHz) spectrum of entry 3 (Table S2).

Sample Chromatograms

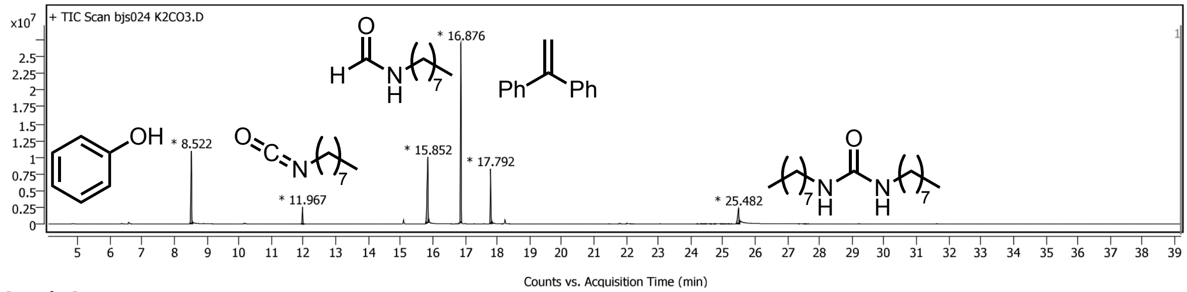

Sample Spectra

+ Scan (rt: 8.493-8.539 min) Sub

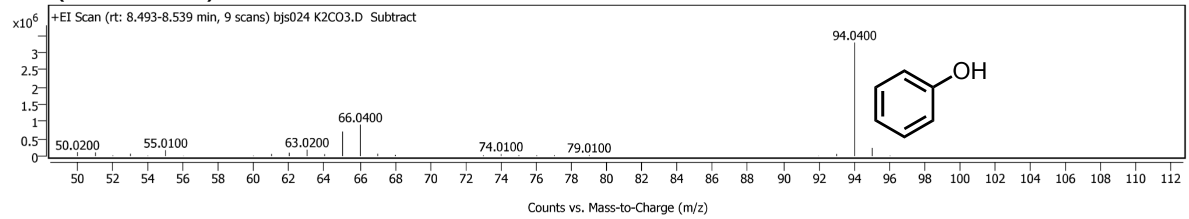

+ Scan (rt: 11.949-11.984 min) Sub

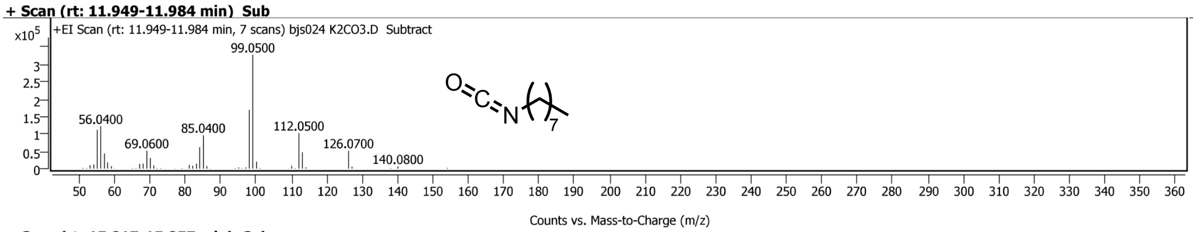

+ Scan (rt: 15.817-15.857 min) Sub

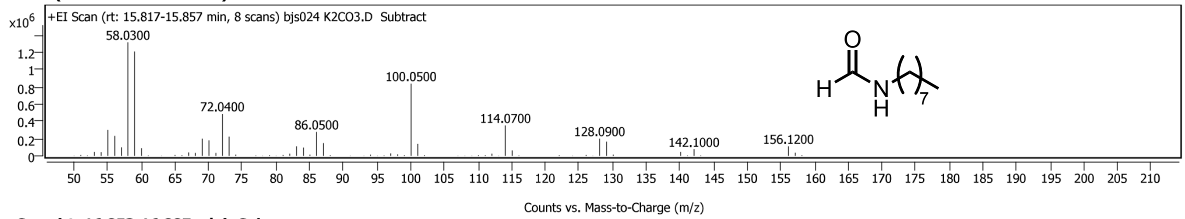

+ Scan (rt: 16.853-16.887 min) Sub

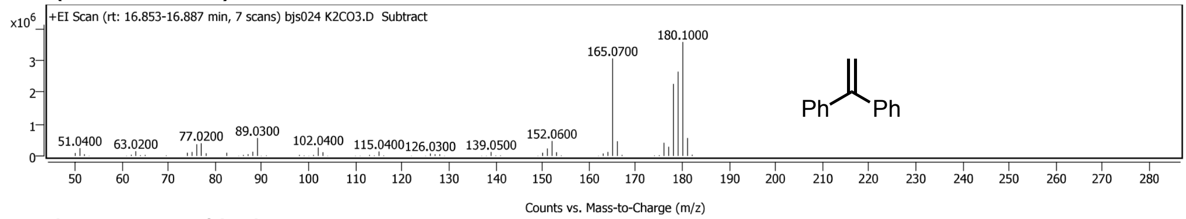

+ Scan (rt: 25.430-25.499 min) Sub

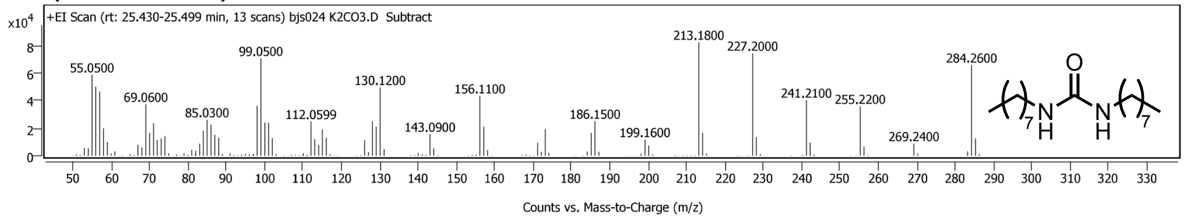

Figure 28: GC-MS data of entry 3 (Table S2).

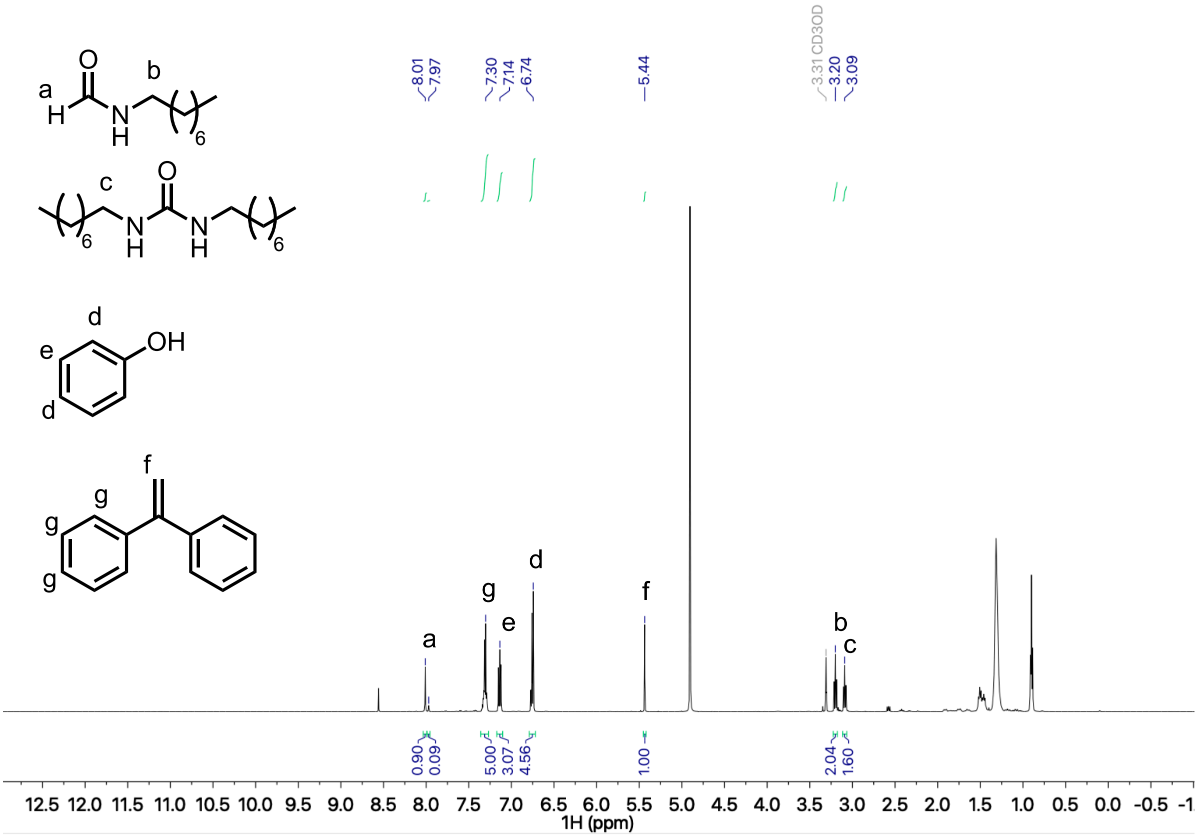

Figure 29: ^{1}H NMR (d[4]-MeOD, 500 MHz) spectrum of entry 4 (Table S2).

Sample Chromatograms

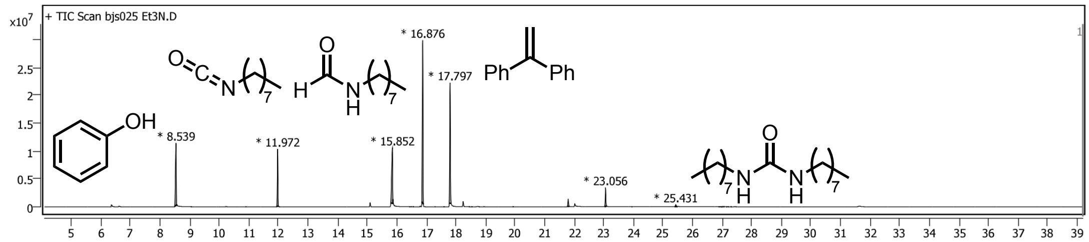

Sample Spectra

+ Scan (rt: 15.818-15.858 min) Sub

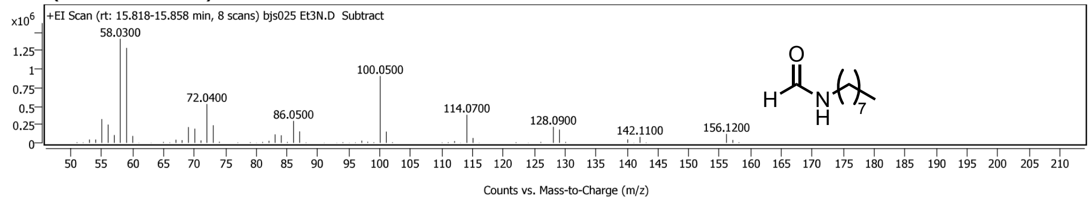

+ Scan (rt: 8.516-8.551 min) Sub

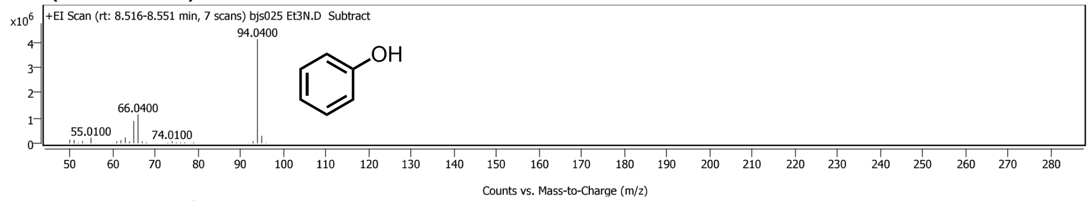

+ Scan (rt: 11.961-11.984 min) Sub

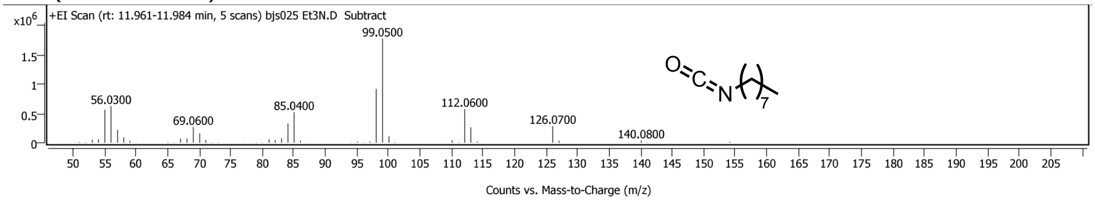

+ Scan (rt: 16.853-16.888 min) Sub

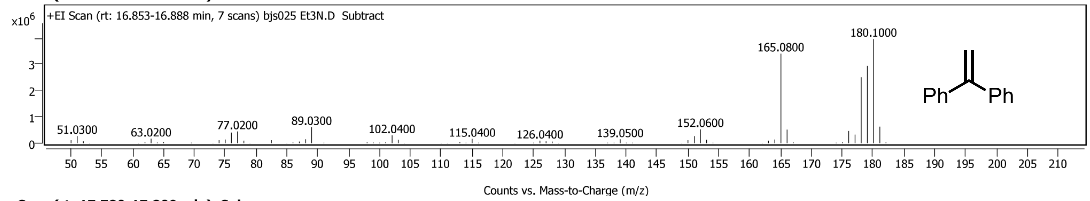

+ Scan (rt: 17.780-17.809 min) Sub

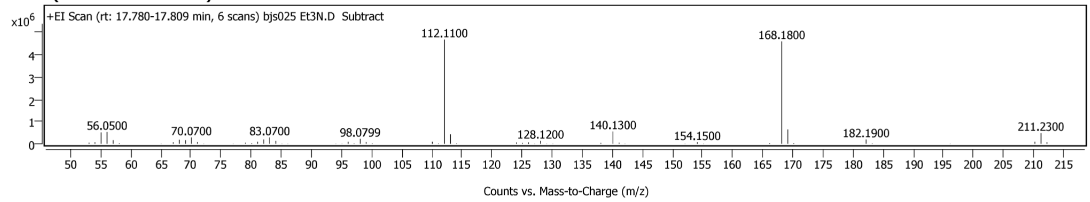

+ Scan (rt: 23.044-23.079 min) Sub

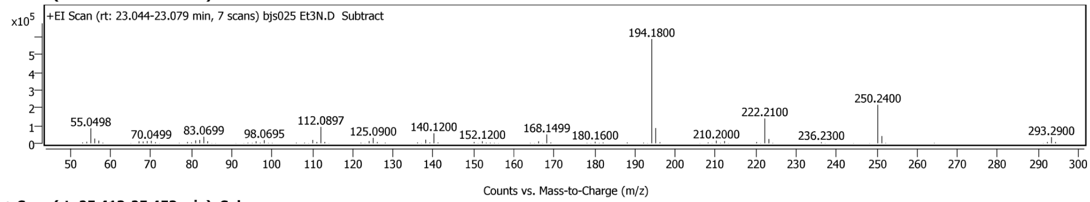

+ Scan (rt: 25.413-25.453 min) Sub

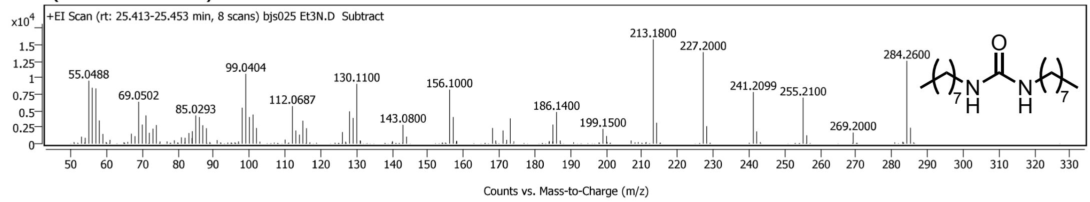

Figure 30: GC-MS data of entry 4 (Table S2). Unknown products at 17.7 and 23.1 min.

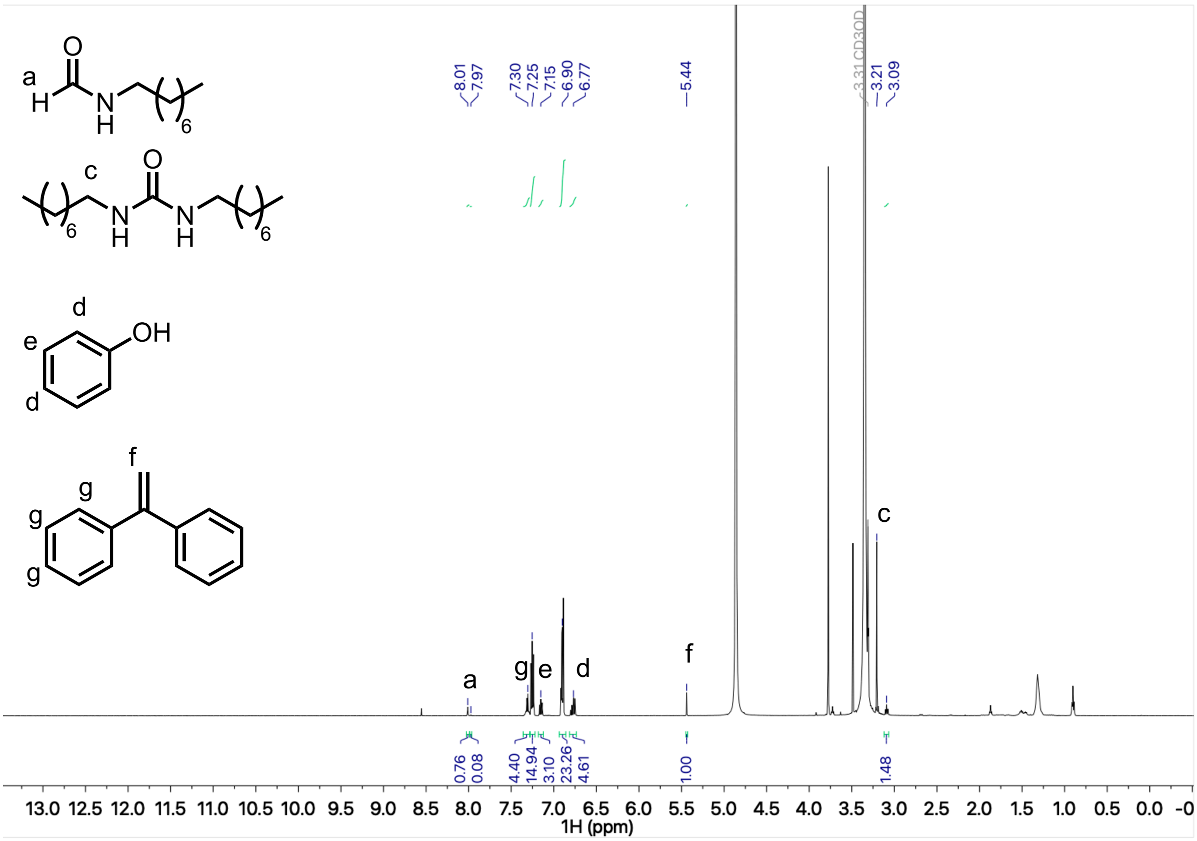

Figure 31: ^{1}H NMR (d^{4}-MeOD, 500 MHz) spectrum of entry 5 (Table S2).

Sample Chromatograms

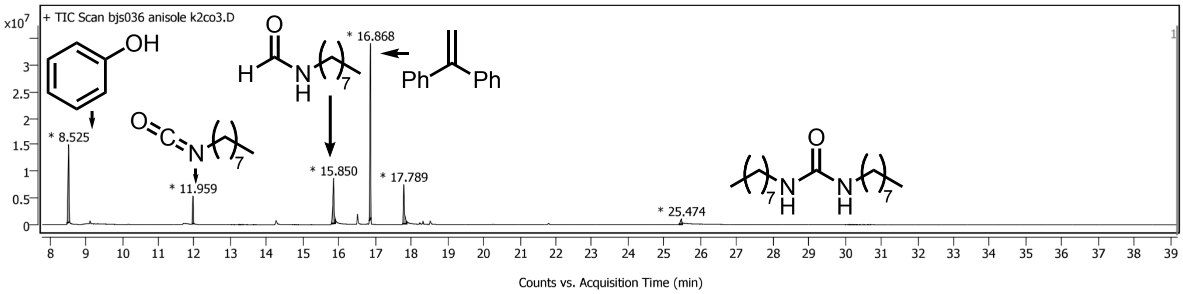

Sample Spectra

+ Scan (rt: 8.497-8.537 min) Sub

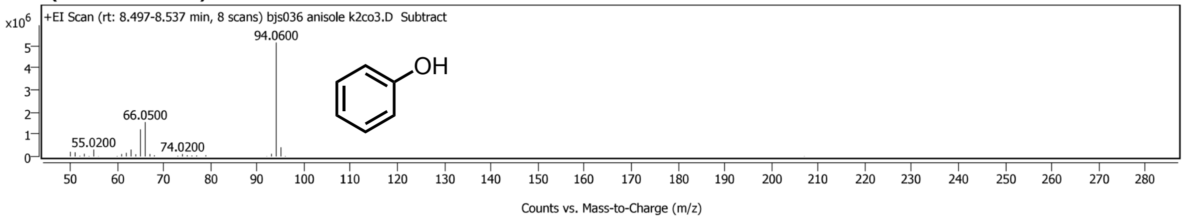

+ Scan (rt: 11.947-11.976 min) Sub

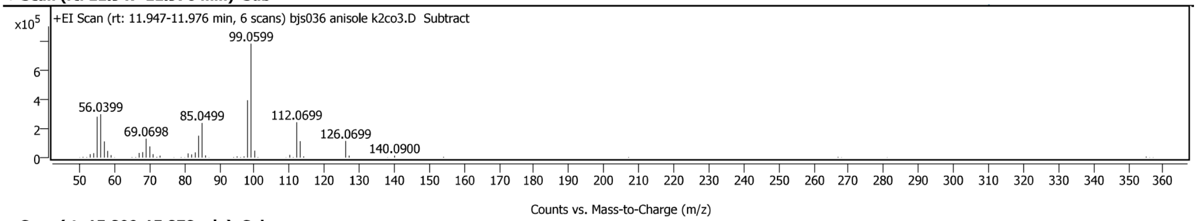

+ Scan (rt: 15.809-15.878 min) Sub

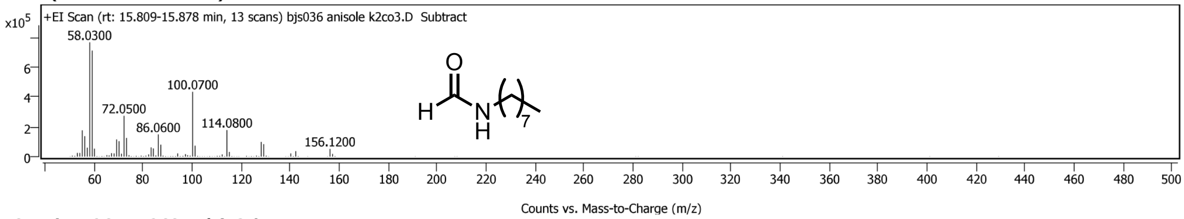

+ Scan (rt: 16.851-16.885 min) Sub

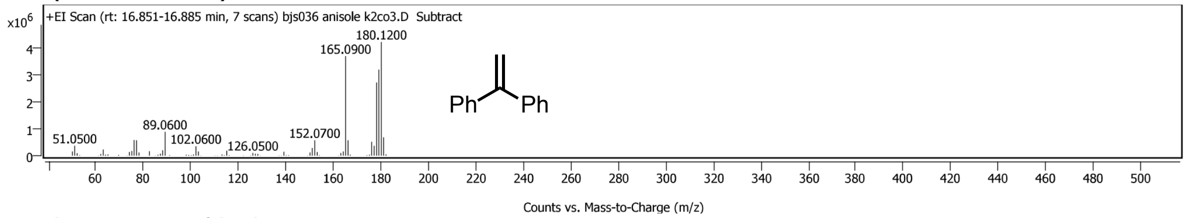

+ Scan (rt: 17.772-17.829 min) Sub

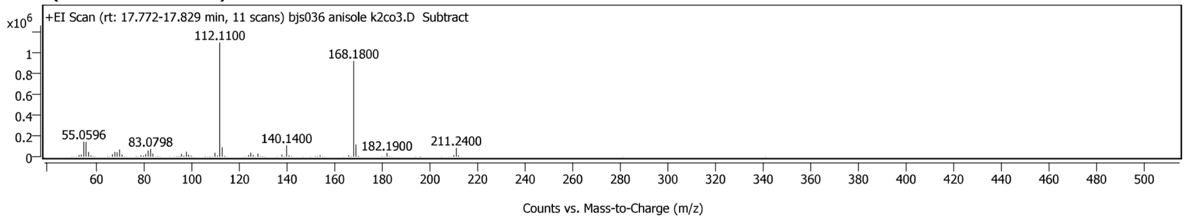

+ Scan (rt: 25.428-25.485 min) Sub

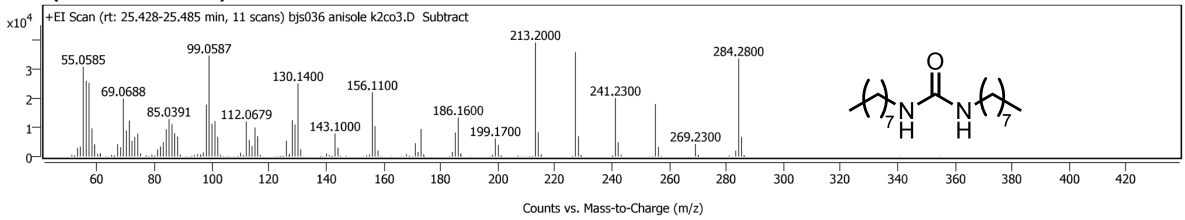

Figure 32: GC-MS data of entry 5 (Table S2). Unknown products at 17.7 min.

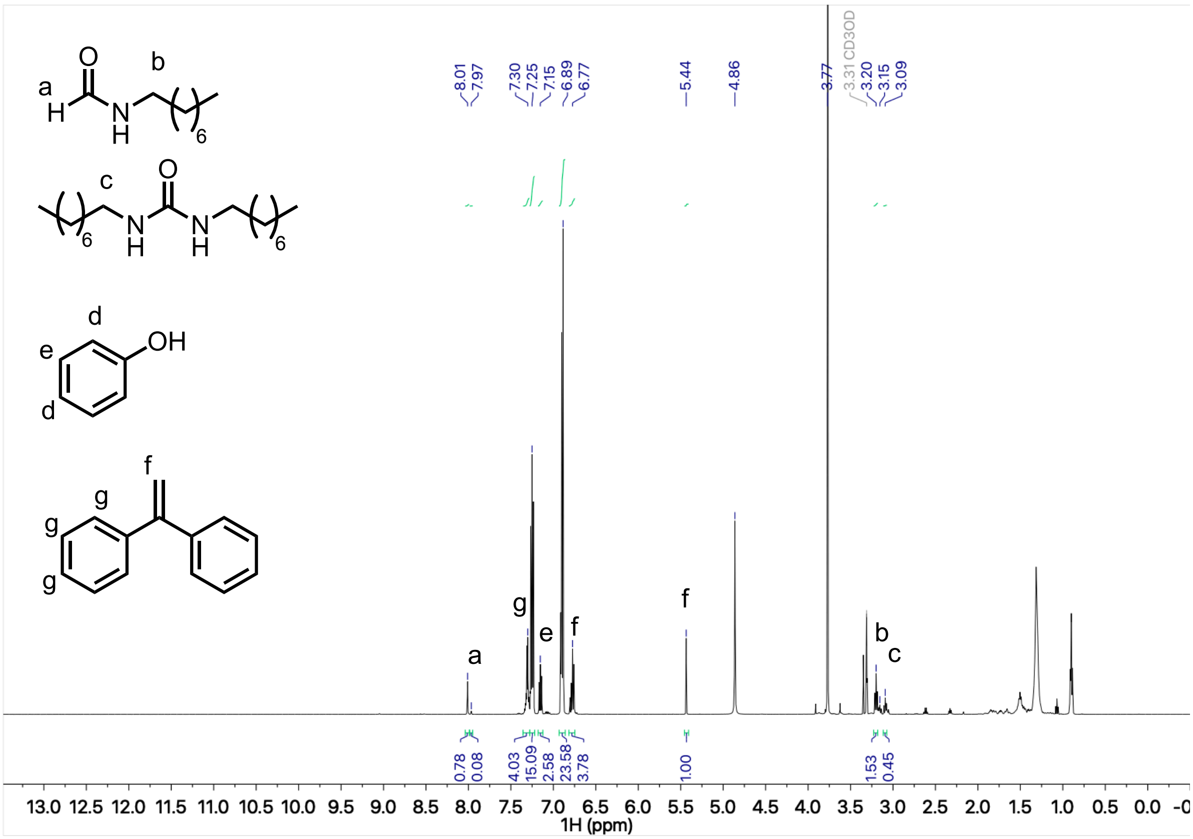

Figure 33: ^{1}H NMR (d^{4}-MeOD, 500 MHz) spectrum of entry 6 (Table S2).

Sample Chromatograms

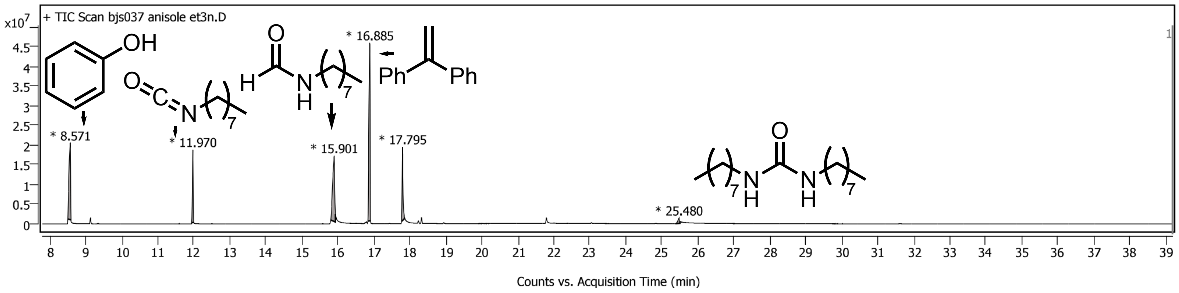

Sample Spectra

+ Scan (rt: 8.525-8.577 min) Sub

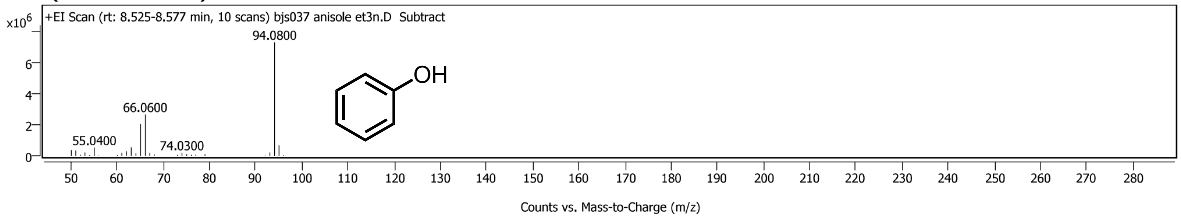

+ Scan (rt: 11.953-11.981 min)

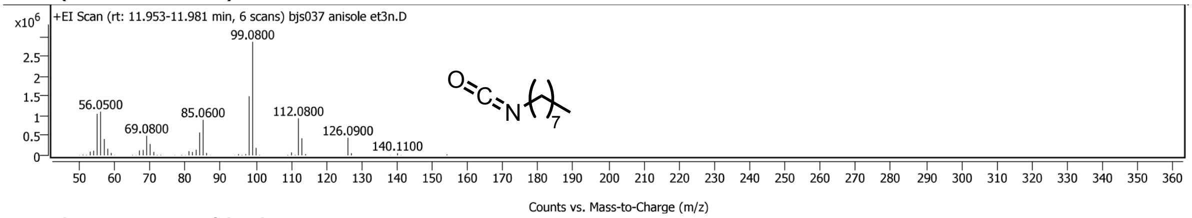

+ Scan (rt: 15.838-15.912 min) Sub

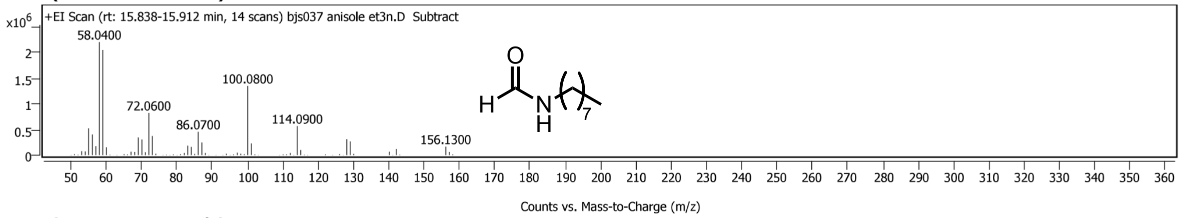

+ Scan (rt: 16.851-16.885 min)

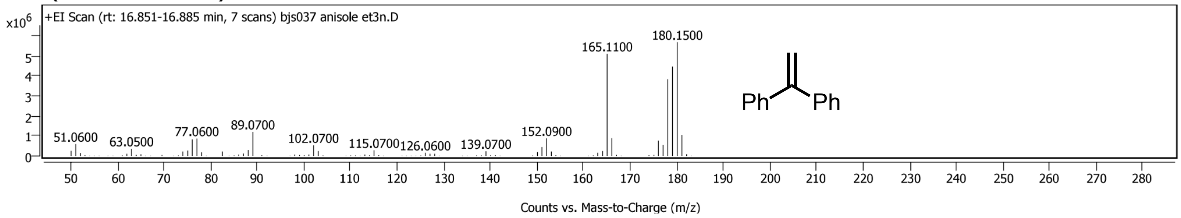

+ Scan (rt: 17.772-17.829 min)

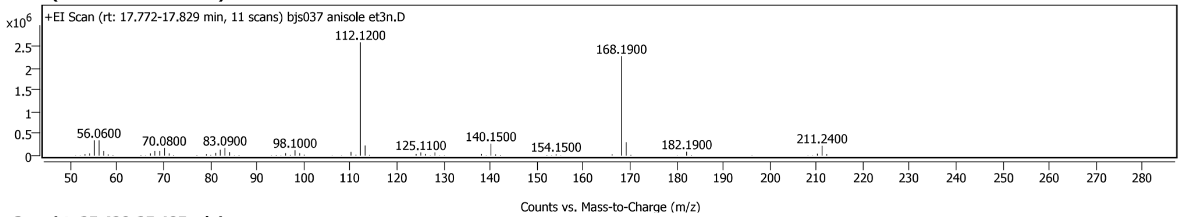

+ Scan (rt: 25.428-25.485 min)

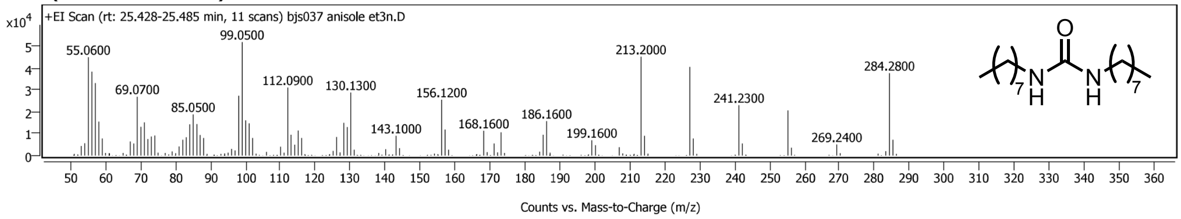

Figure 34: GC-MS spectrum of entry 6. Unknown products at 17.7 min.

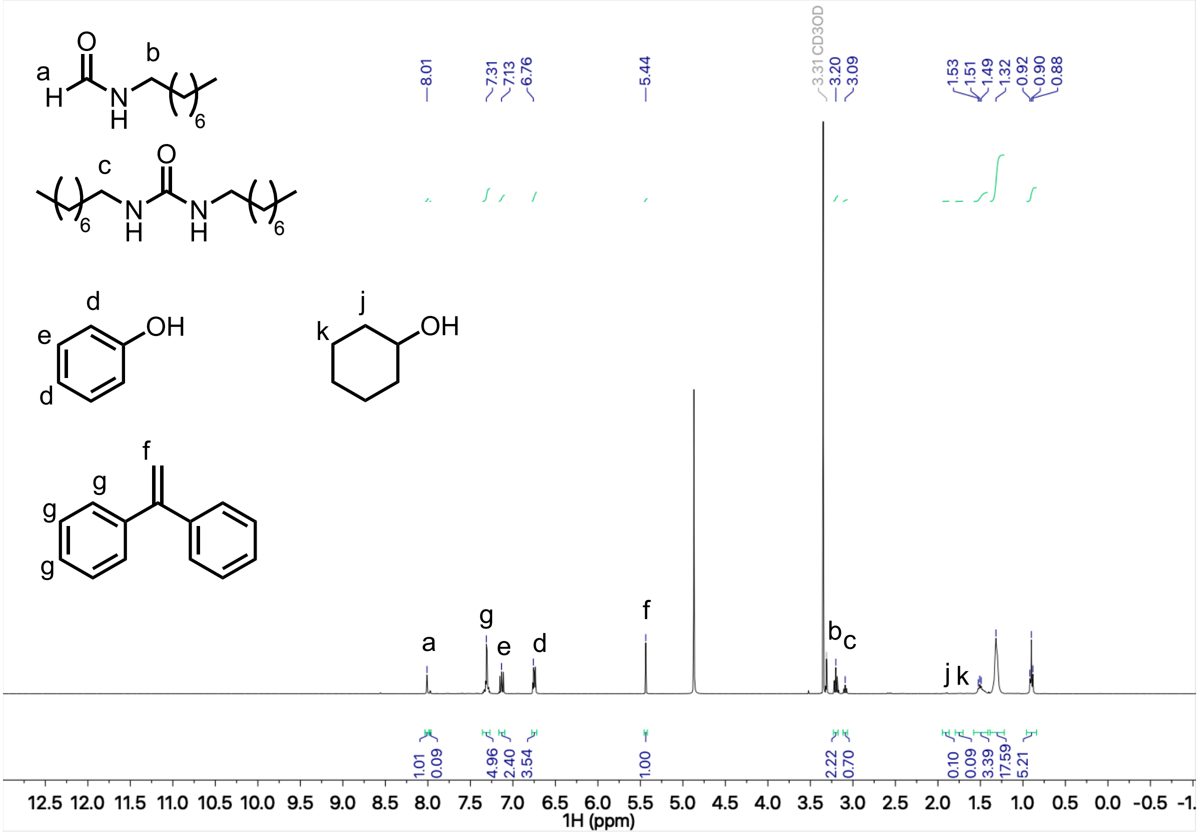

Figure 35: ^{1}H NMR (d[4]-MeOD, 500 MHz) spectrum of entry 7.

Sample Chromatograms

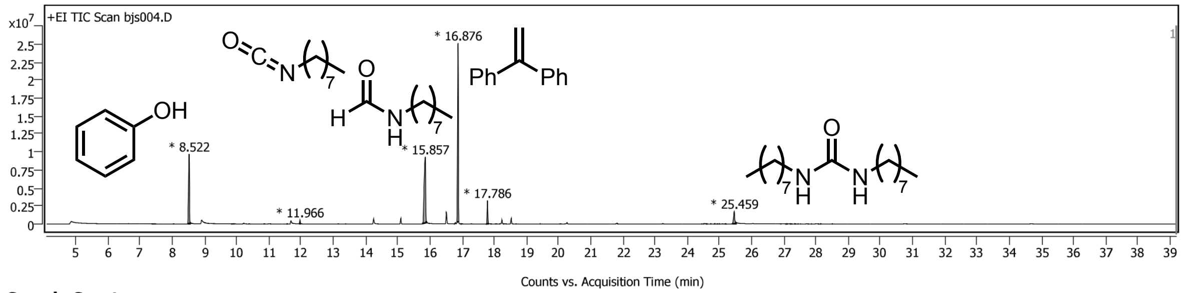

Sample Spectra

+ Scan (rt: 8.493-8.539 min) Sub

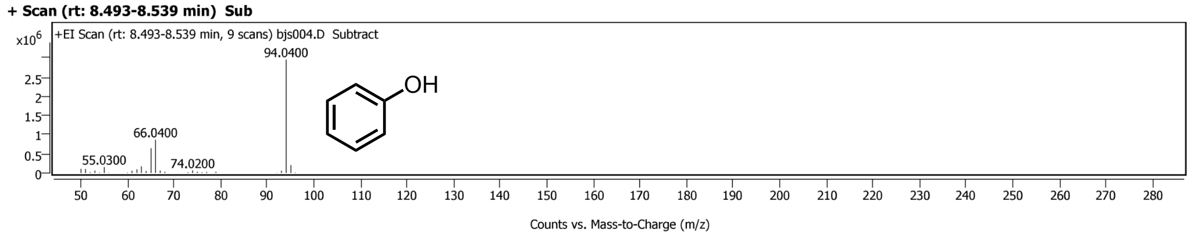

+ Scan (rt: 11.949-11.984 min) Sub

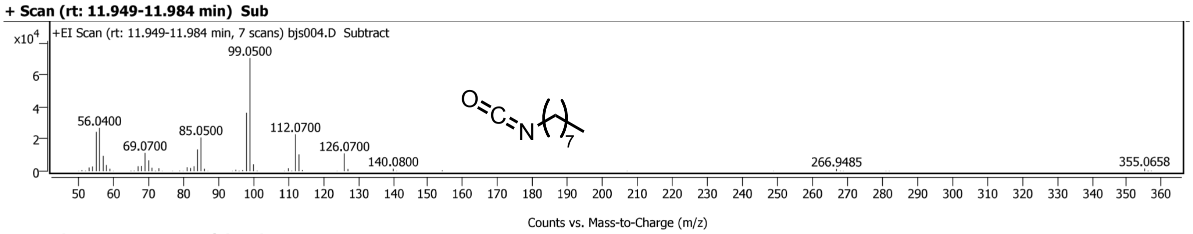

+ Scan (rt: 15.800-15.875 min) Sub

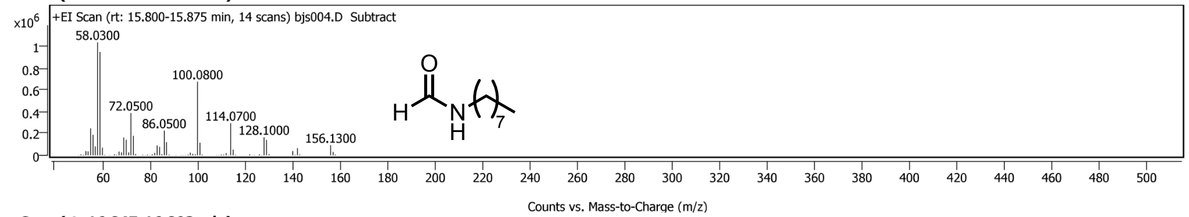

+ Scan (rt: 16.847-16.893 min)

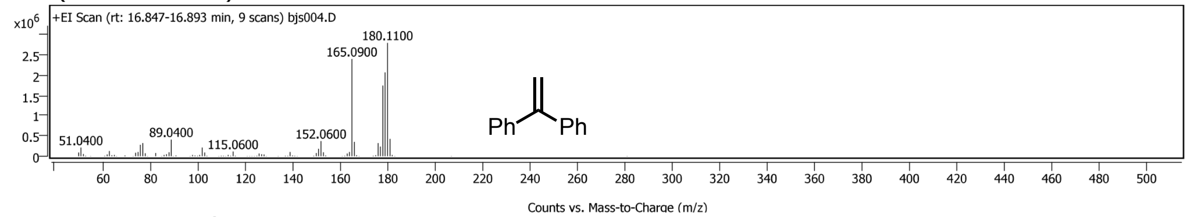

+ Scan (rt: 25.413-25.493 min) Sub

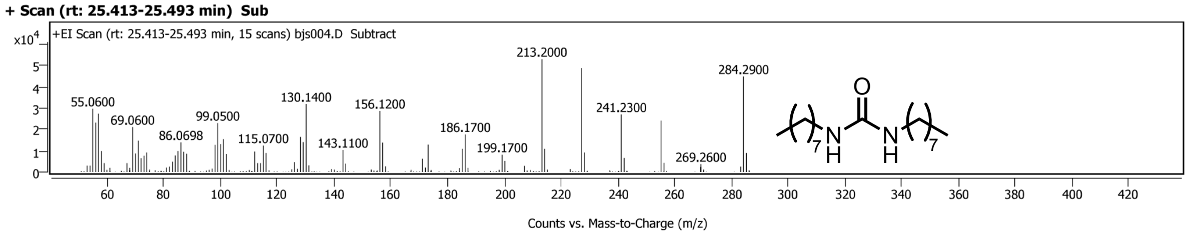

Figure 36: GC-MS data of entry 7 (Table S2). Unknown product at 17.7 min

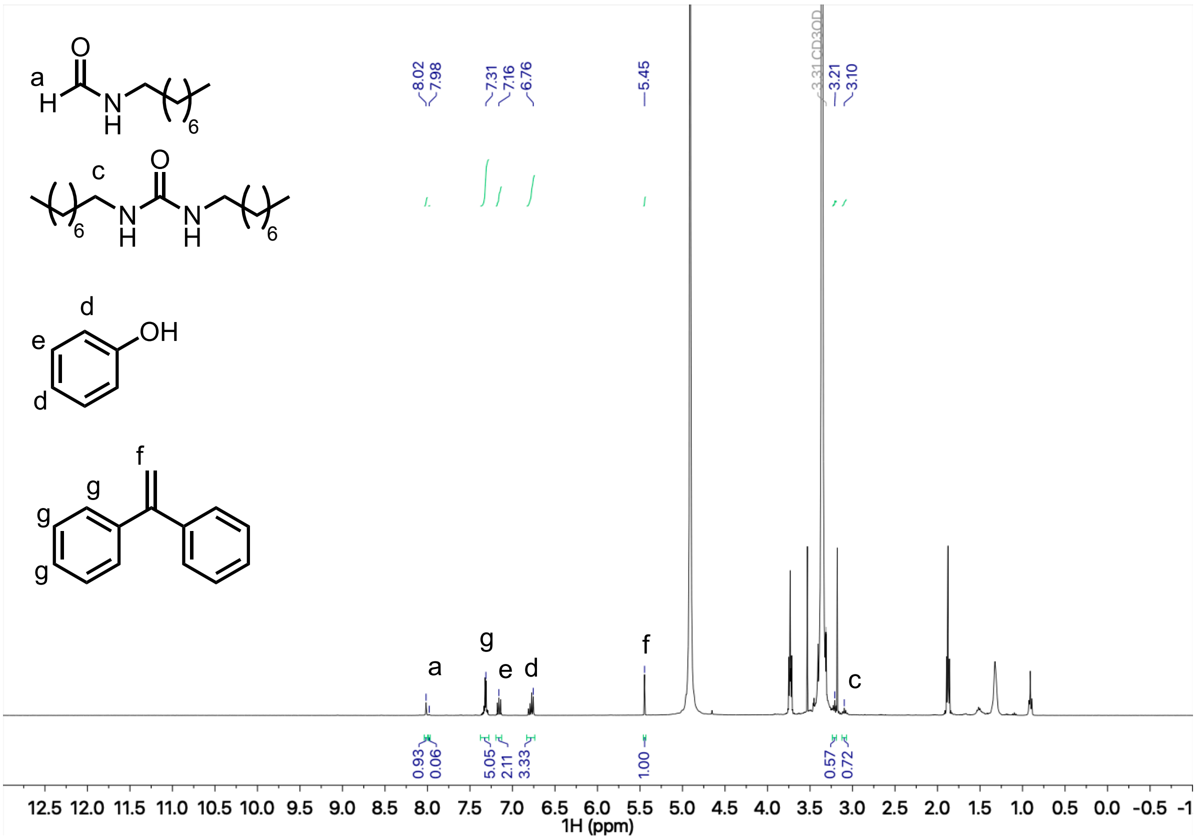

Figure 37: ^{1}H NMR (d[4]-MeOD, 500 MHz) spectrum of entry 8 (Table S2).

Sample Chromatograms

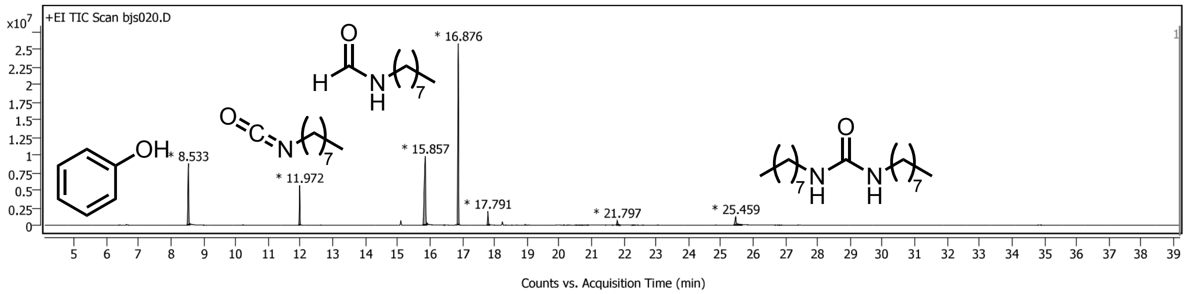

Sample Spectra

+ Scan (rt: 8.505-8.550 min) Sub

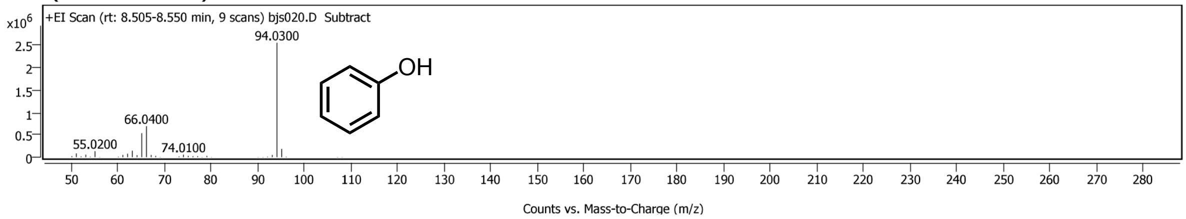

+ Scan (rt: 11.955-11.989 min) Sub

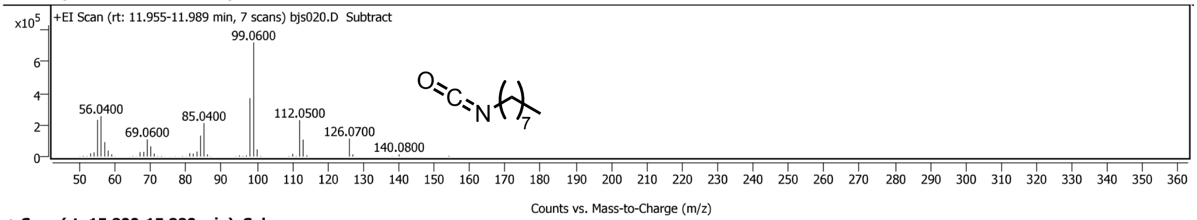

+ Scan (rt: 15.800-15.880 min) Sub

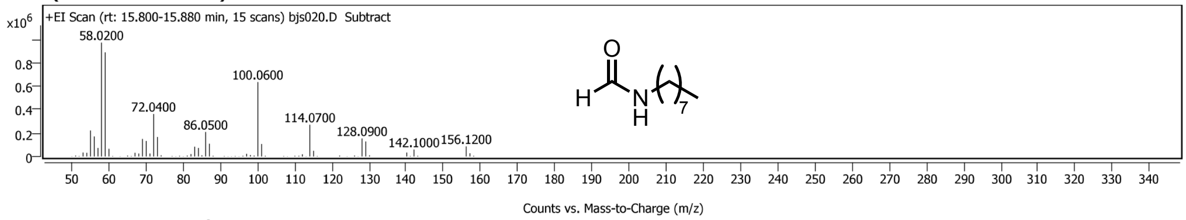

+ Scan (rt: 16.847-16.893 min) Sub

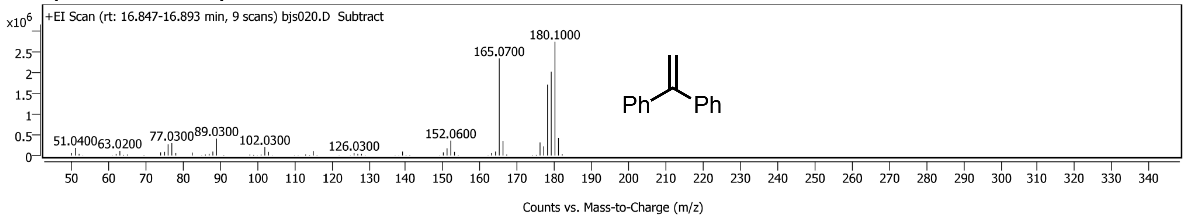

+ Scan (rt: 17.786-17.803 min) Sub

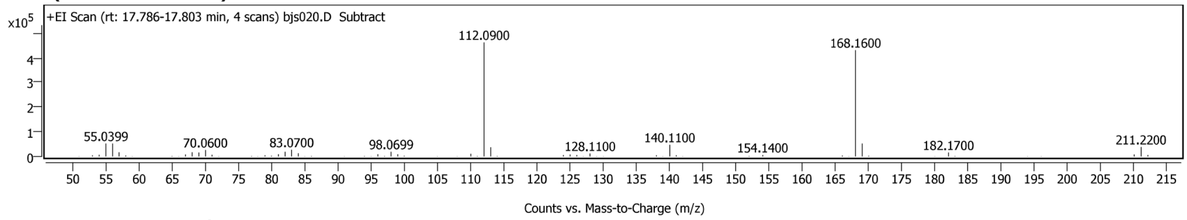

+ Scan (rt: 25.407-25.573 min) Sub

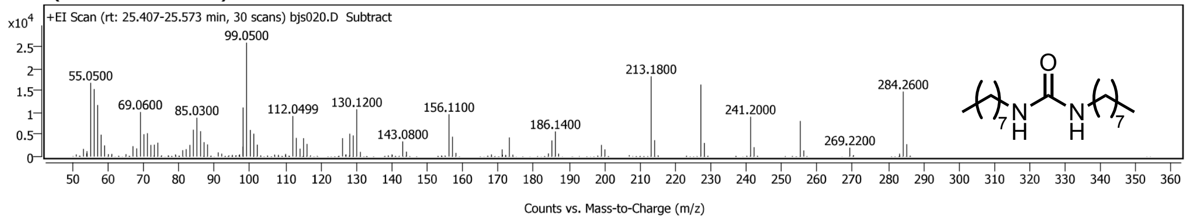

Figure 38: GC-MS data of entry 8 (Table S2). Unknown product at 17.7 and 21.8 min

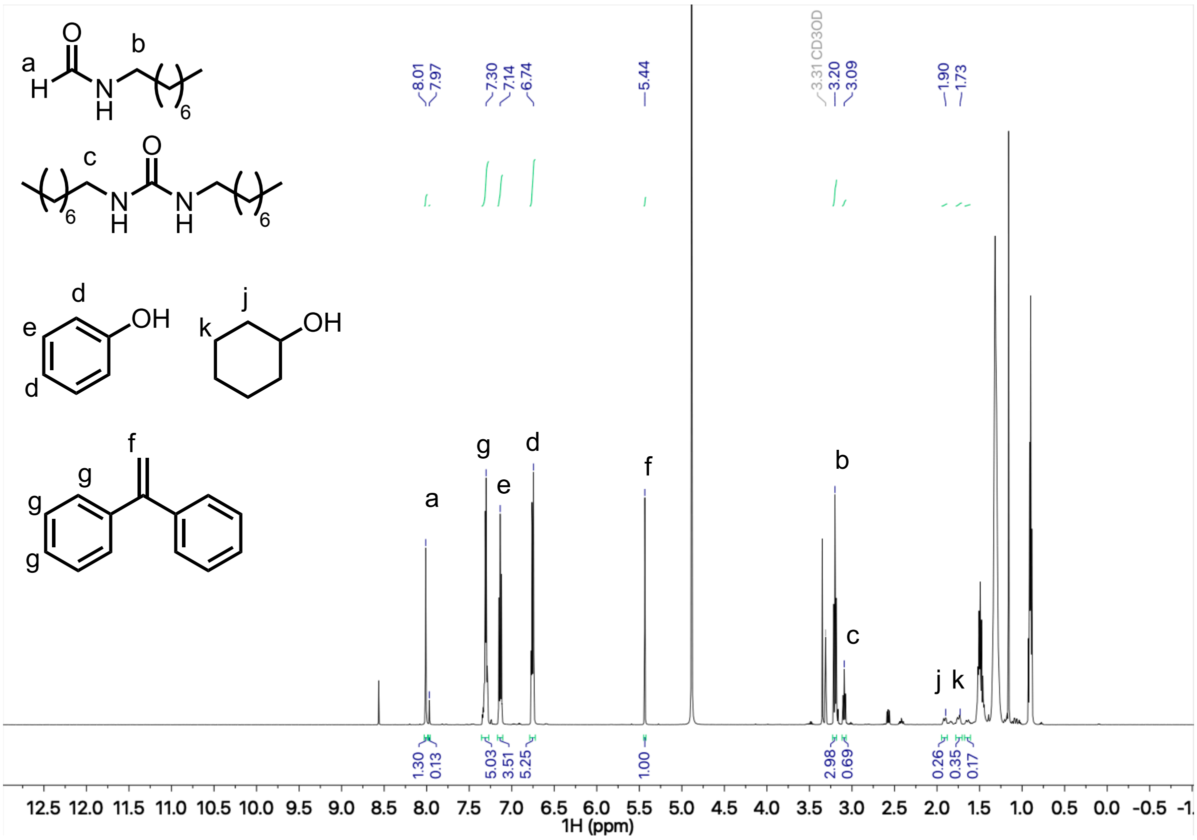

Figure 39: ^{1}H NMR (d[4]-MeOD, 500 MHz) spectrum of entry 9 (Table S2).

Sample Chromatograms

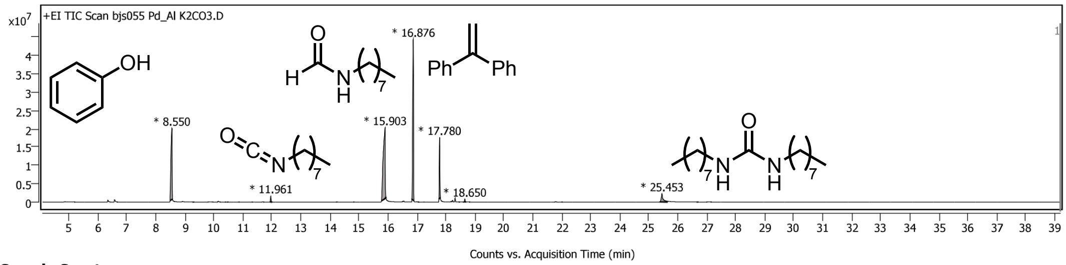

Sample Spectra

+ Scan (rt: 8.510-8.562 min) Sub

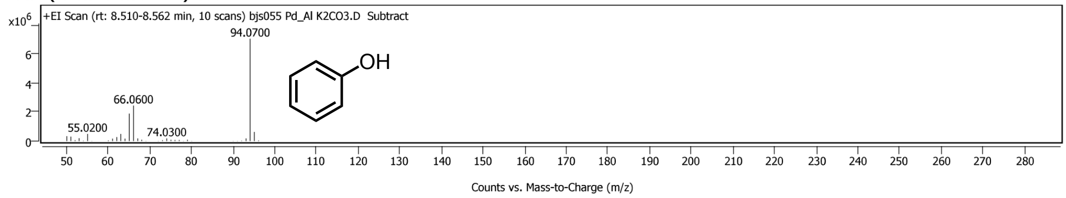

+ Scan (rt: 11.949-11.972 min) Sub

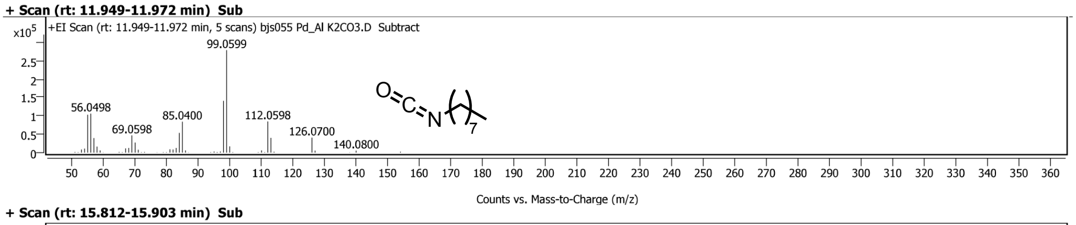

+ Scan (rt: 15.812-15.903 min) Sub

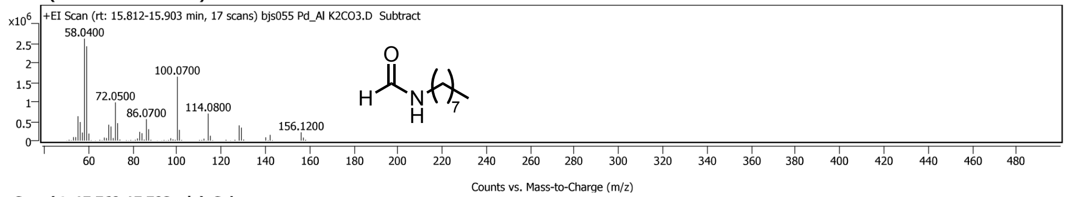

+ Scan (rt: 17.769-17.792 min) Sub

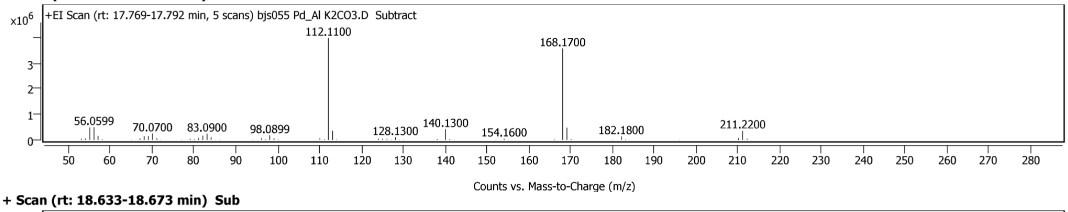

+ Scan (rt: 18.633-18.673 min) Sub

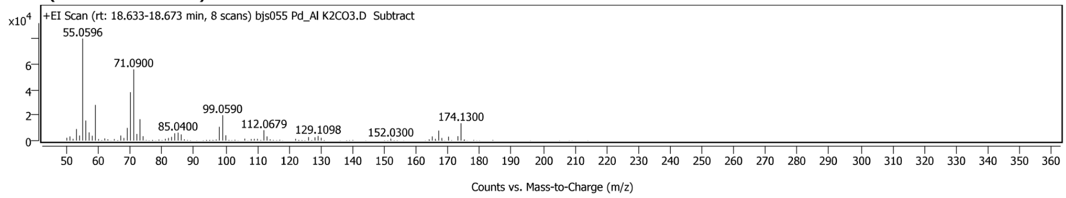

+ Scan (rt: 16.847-16.882 min) Sub

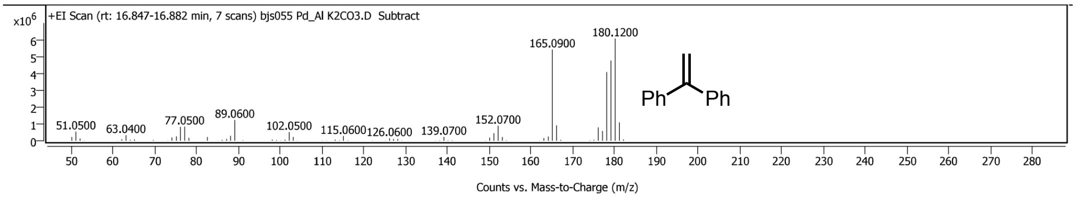

+ Scan (rt: 25.408-25.556 min) Sub

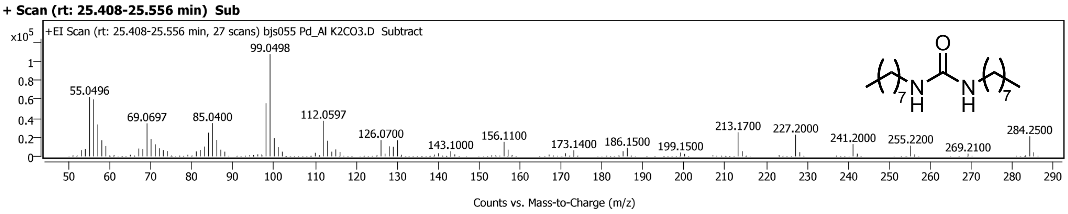

Figure 40: GC-MS data of entry 9 (Table S2). Unknown products at 17.1 and 18.6 min

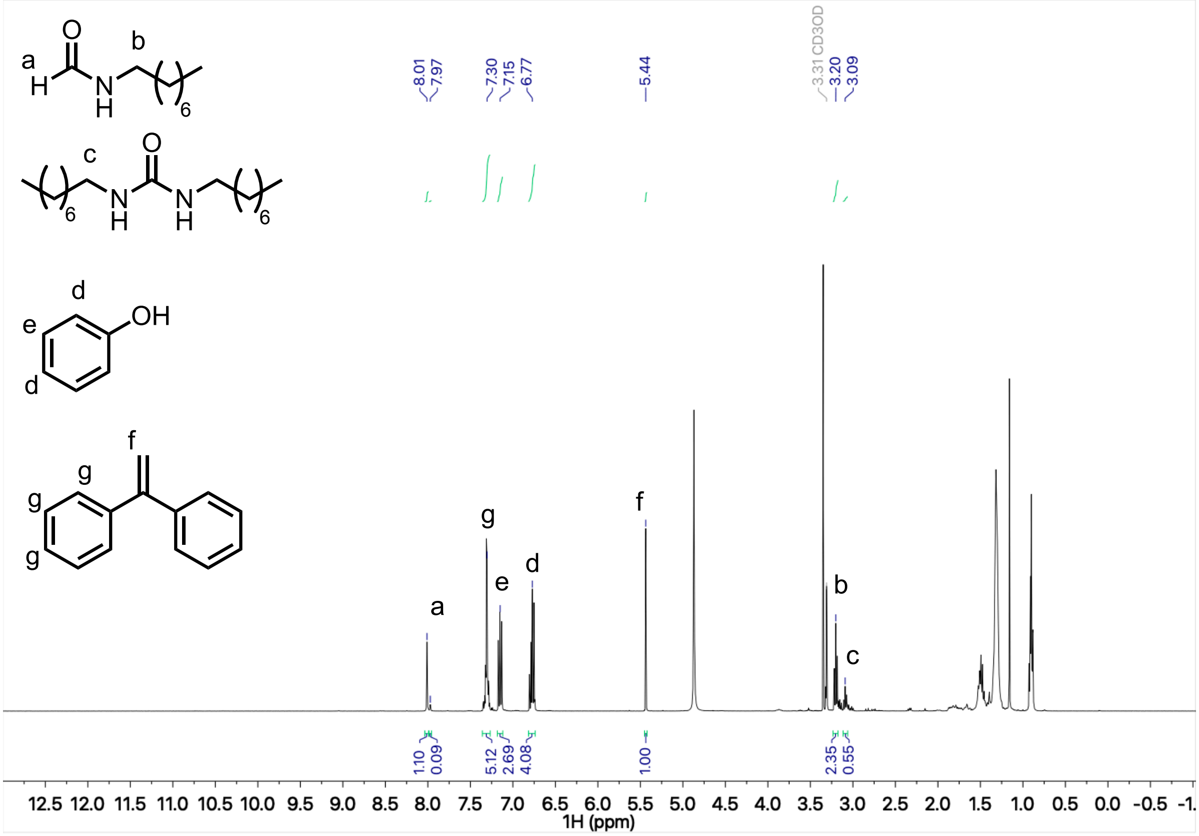

Figure 41: ^{1}H NMR (d[4]-MeOD, 500 MHz) spectrum of entry 10 (Table S2).

Sample Chromatograms

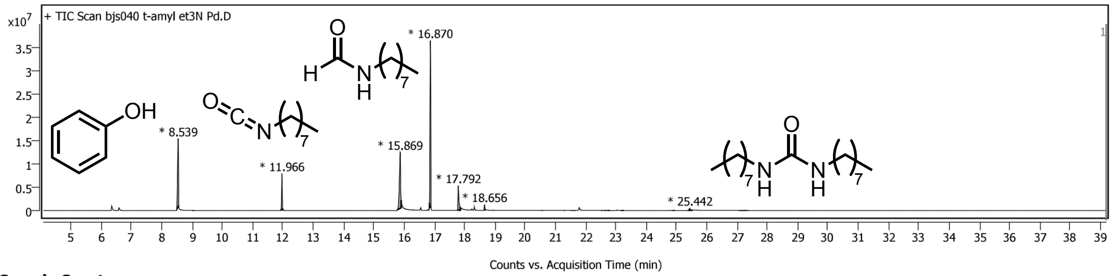

Sample Spectra

+ Scan (rt: 8.510-8.550 min) Sub

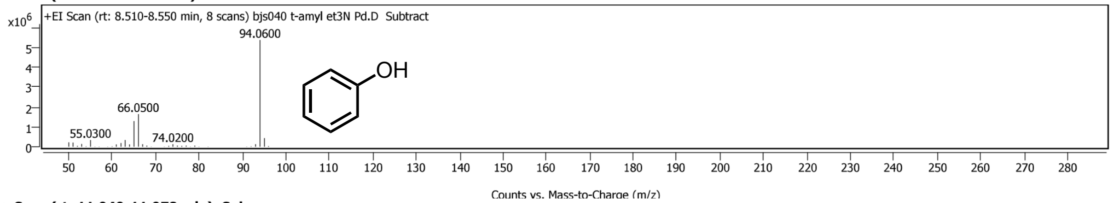

+ Scan (rt: 11.949-11.972 min) Sub

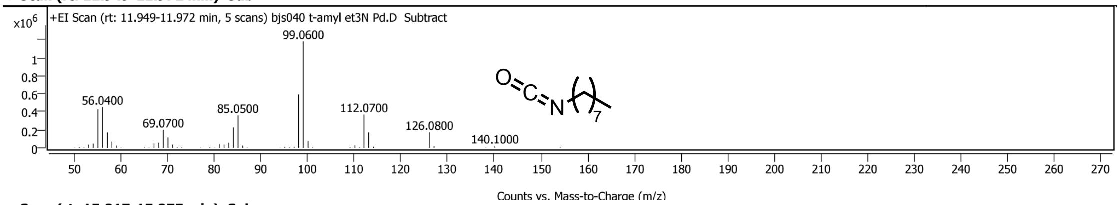

+ Scan (rt: 15.817-15.875 min) Sub

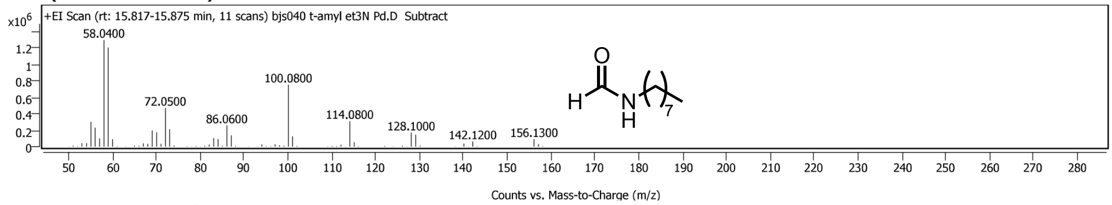

+ Scan (rt: 16.842-16.876 min) Sub

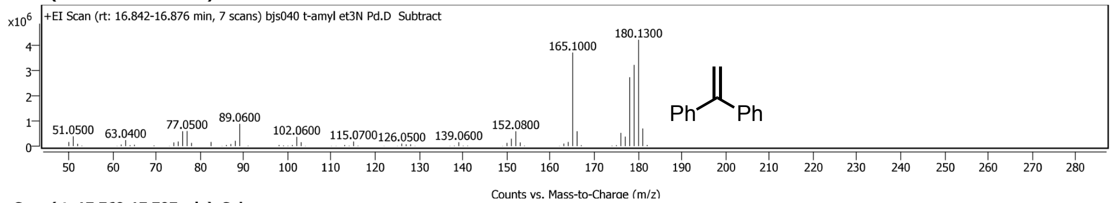

+ Scan (rt: 17.769-17.797 min) Sub

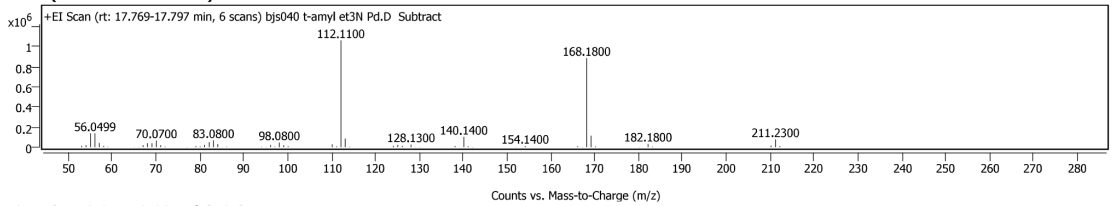

+ Scan (rt: 18.644-18.667 min) Sub

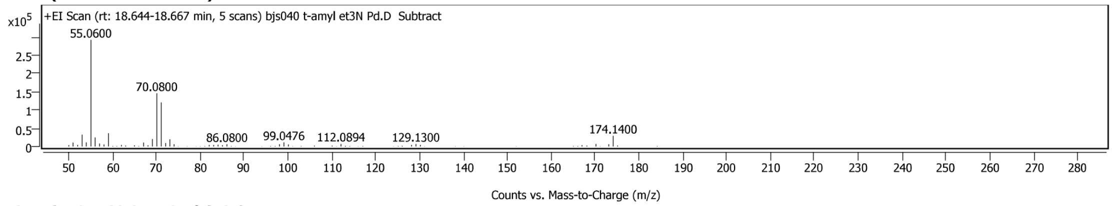

+ Scan (rt: 25.408-25.459 min) Sub

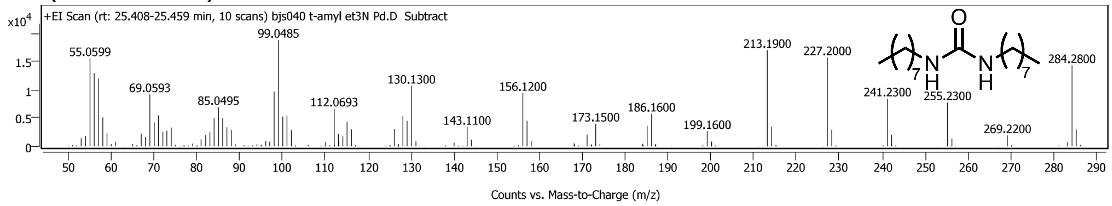

Figure 42: GC-MS data of entry 10 (Table S2). Unknown products at 17.1 and 18.6 min

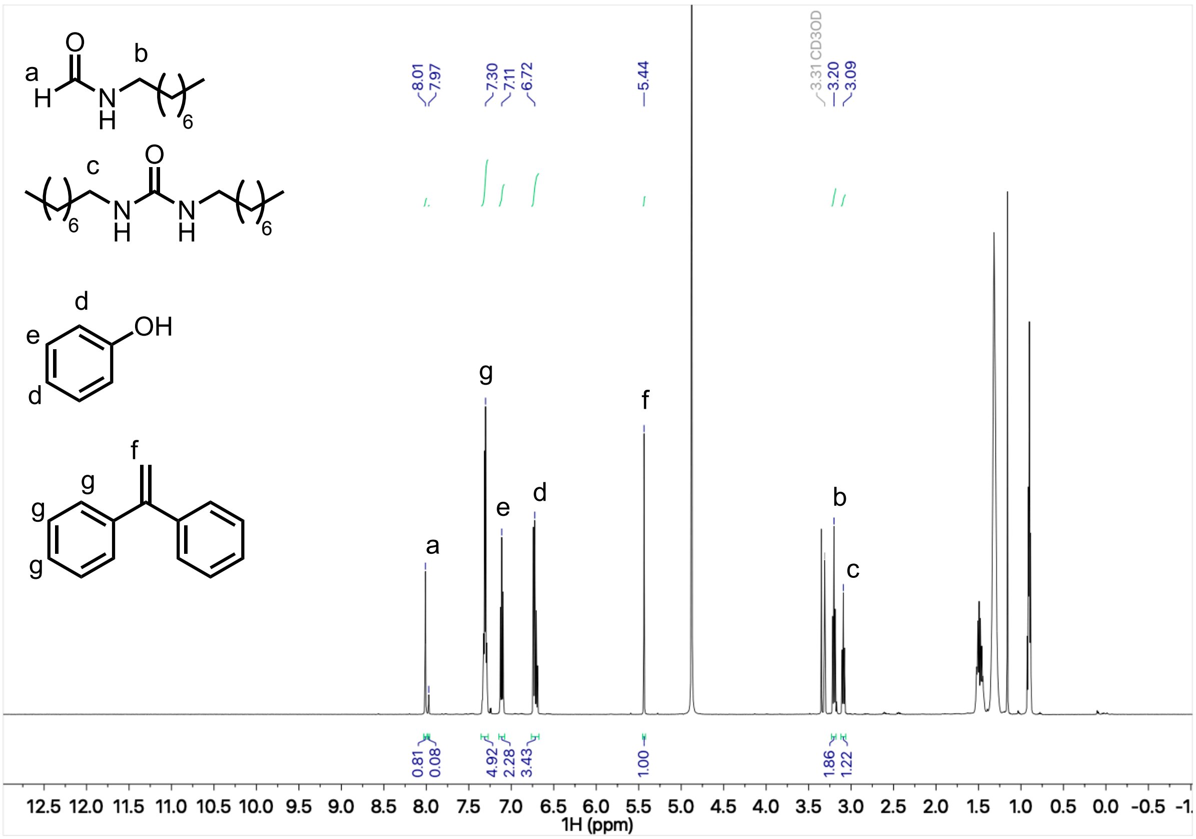

Figure 43: ^{1}H NMR (d^4-MeOD, 500 MHz) spectrum of entry 11 (Table S2).

Sample Chromatograms

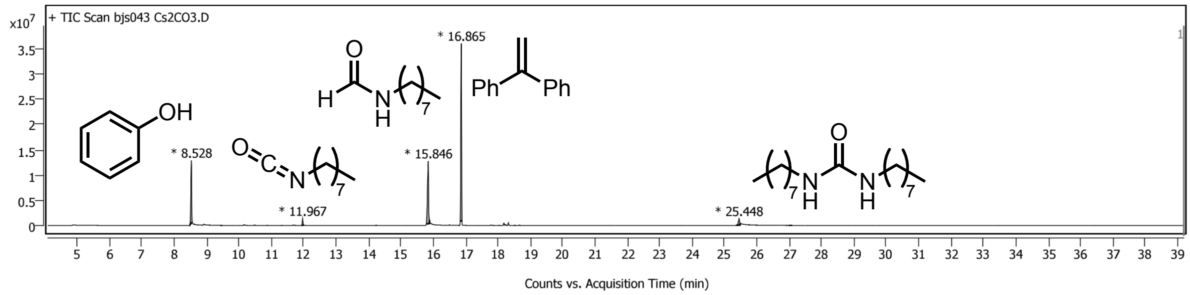

Sample Spectra

+ Scan (rt: 8.505-8.545 min) Sub

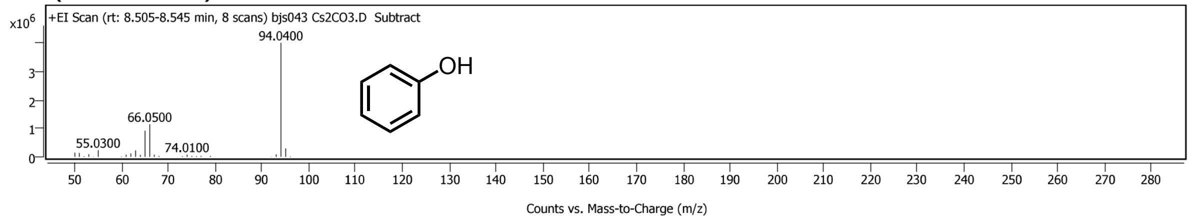

+ Scan (rt: 11.949-11.978 min) Sub

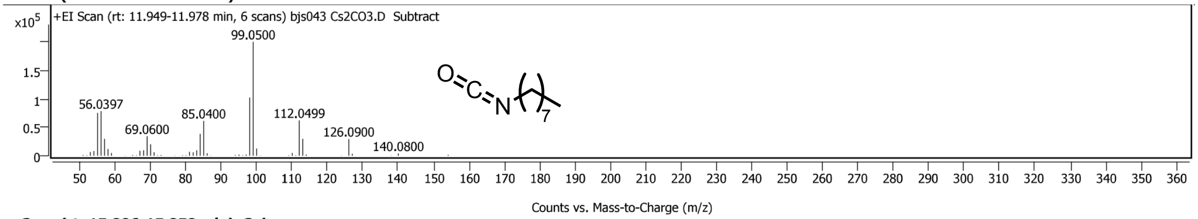

+ Scan (rt: 15.806-15.858 min) Sub

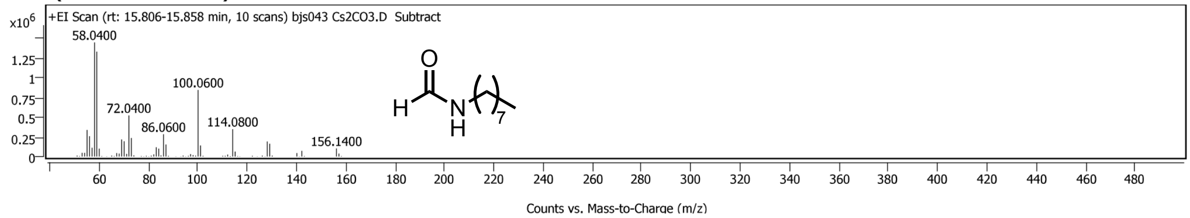

+ Scan (rt: 16.842-16.882 min) Sub

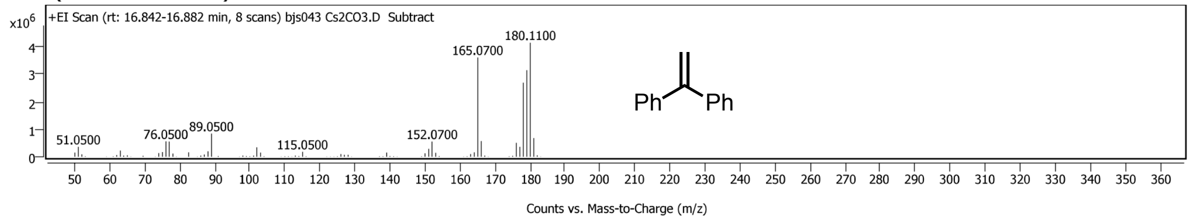

+ Scan (rt: 25.408-25.459 min) Sub

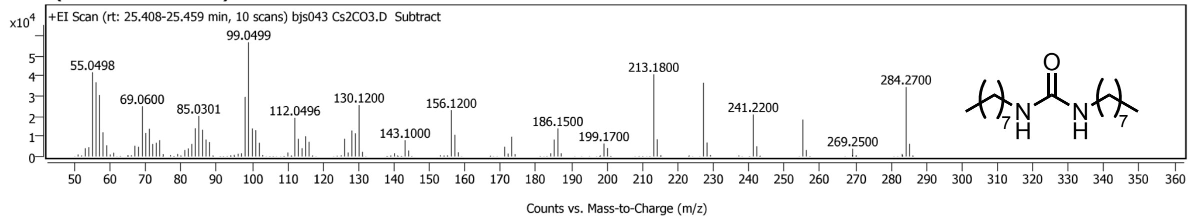

Figure 44: GC-MS data of entry 11 (Table S2).

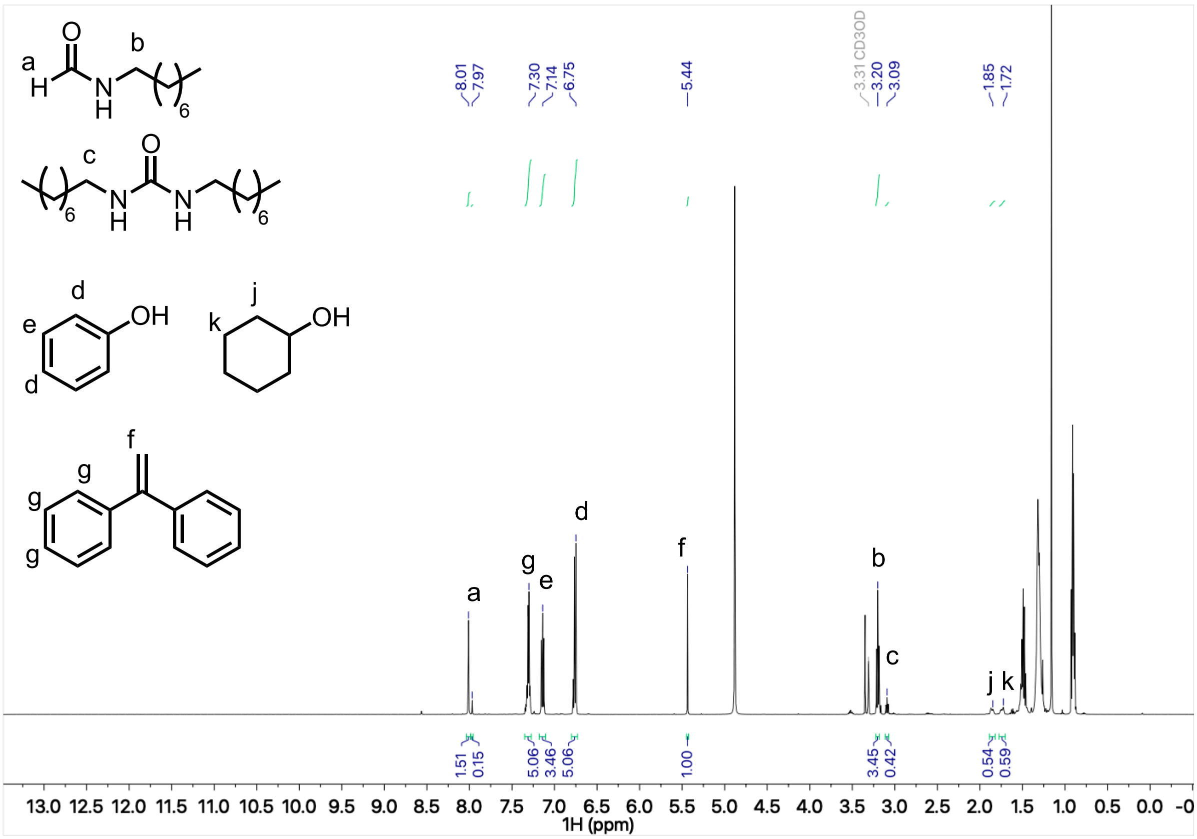

Figure 45: ^{1}H NMR (d[4]-MeOD, 500 MHz) spectrum of entry 12 (Table S2).

Sample Chromatograms

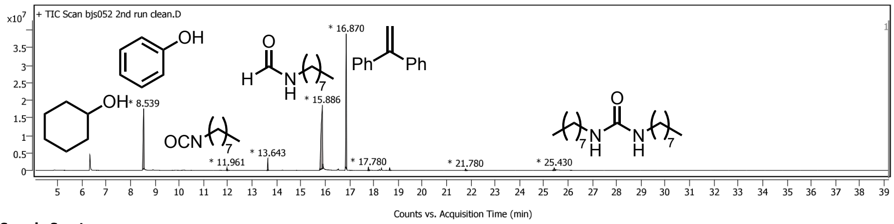

Sample Spectra

+ Scan (rt: 6.313-6.347 min) Sub

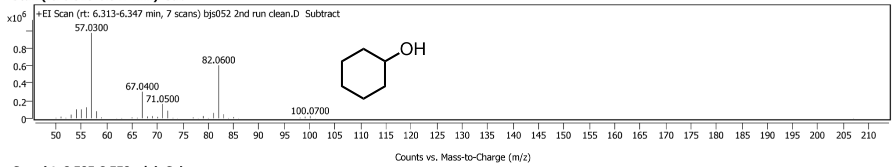

+ Scan (rt: 8.505-8.550 min) Sub

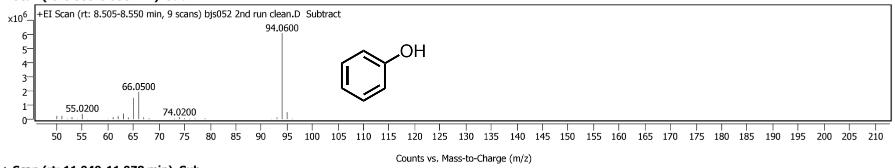

+ Scan (rt: 11.949-11.978 min) Sub

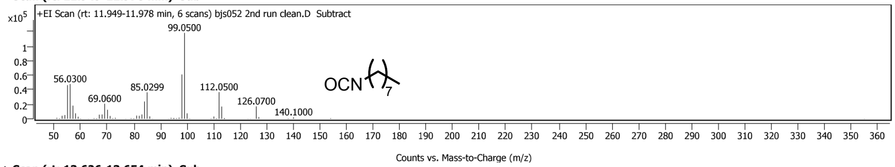

+ Scan (rt: 13.626-13.654 min) Sub

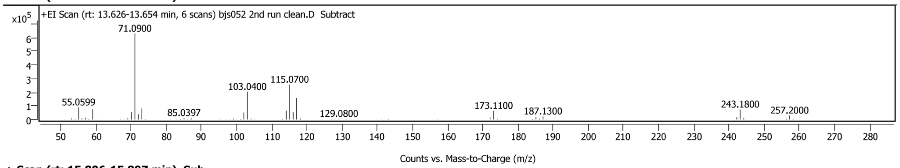

+ Scan (rt: 15.806-15.897 min) Sub

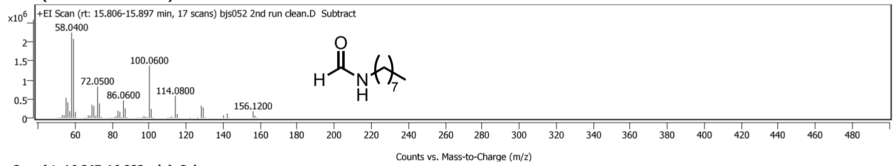

+ Scan (rt: 16.847-16.882 min) Sub

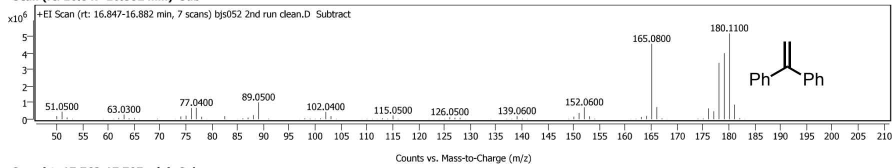

+ Scan (rt: 17.763-17.797 min) Sub

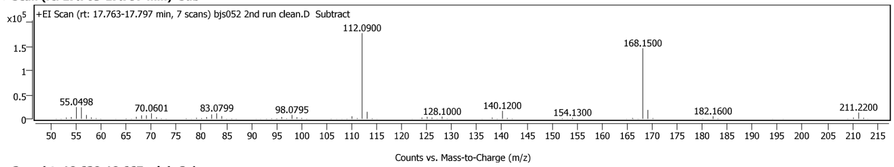

+ Scan (rt: 18.638-18.667 min) Sub

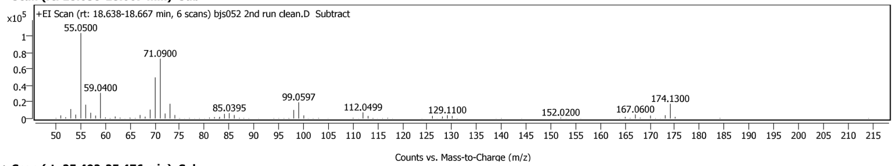

+ Scan (rt: 25.402-25.476 min) Sub

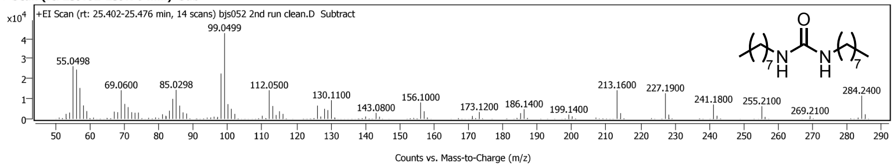

Figure 46: GC-MS data of entry 12 (Table S2). Unknown products at 13.6, 17.8 and 18.6 min in GC.

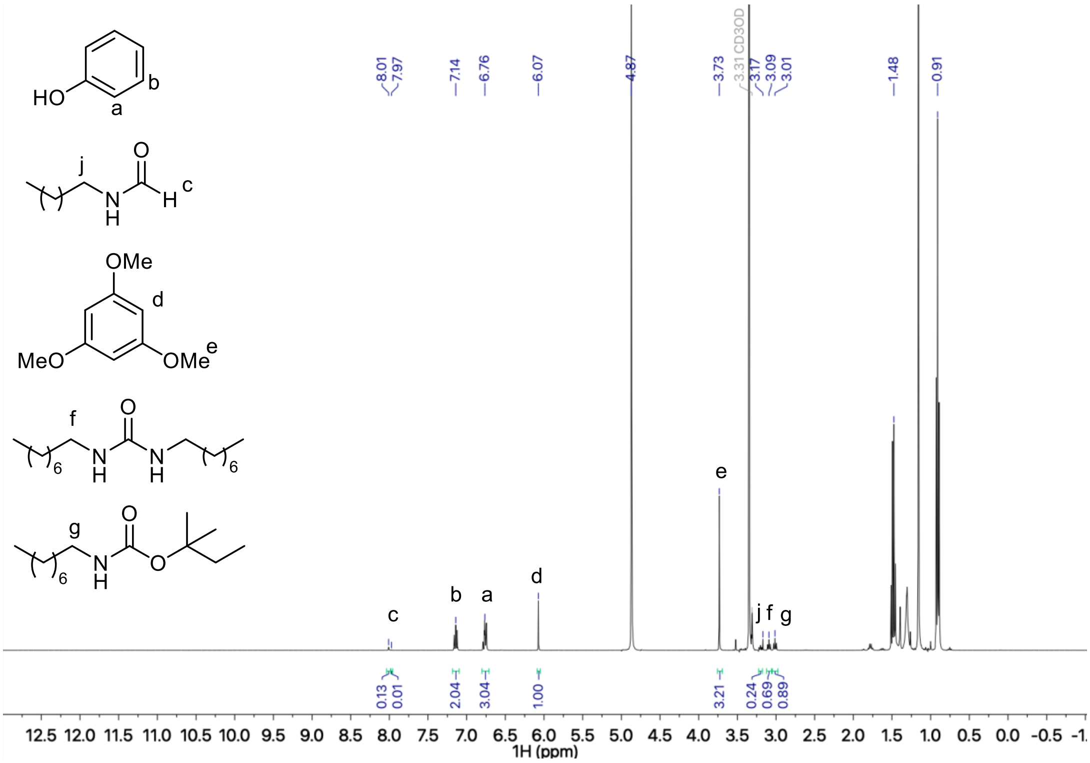

Figure 47:^{1}H NMR (d^4-MeOD, 500 MHz) spectrum of entry 13 (Table S2).

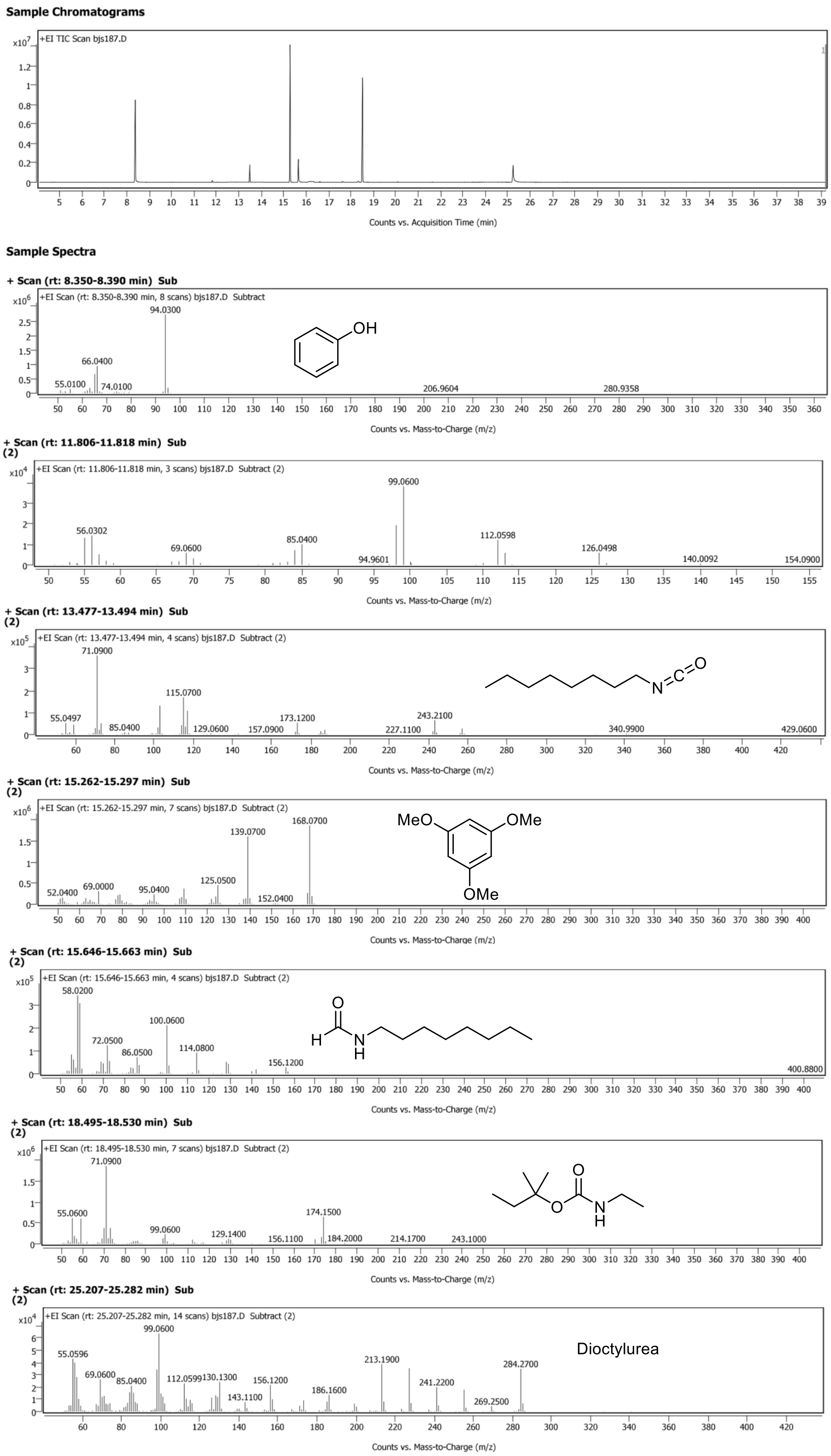

Figure 48: GC-MS data of entry 13 (Table S2).

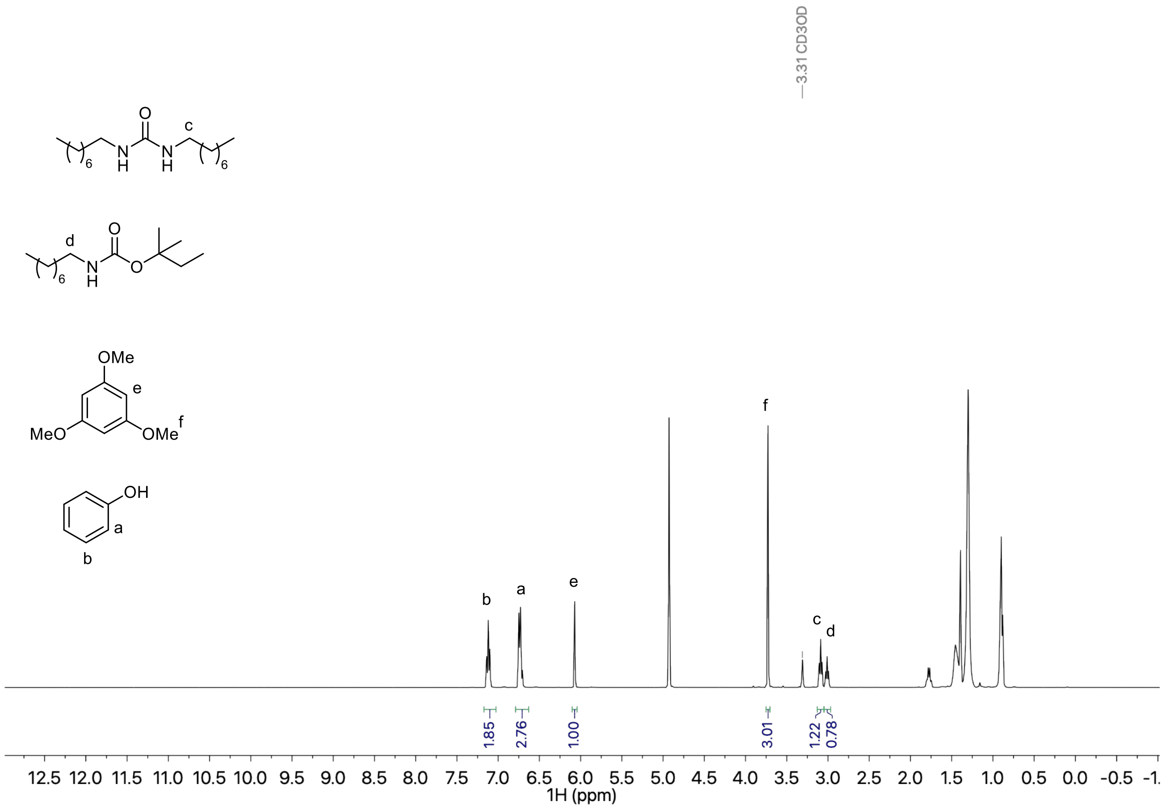

Figure 49:[1]*H NMR (d*[4]*-MeOD, 500 MHz) spectrum of entry 14 (Table S2).*

Sample Chromatograms

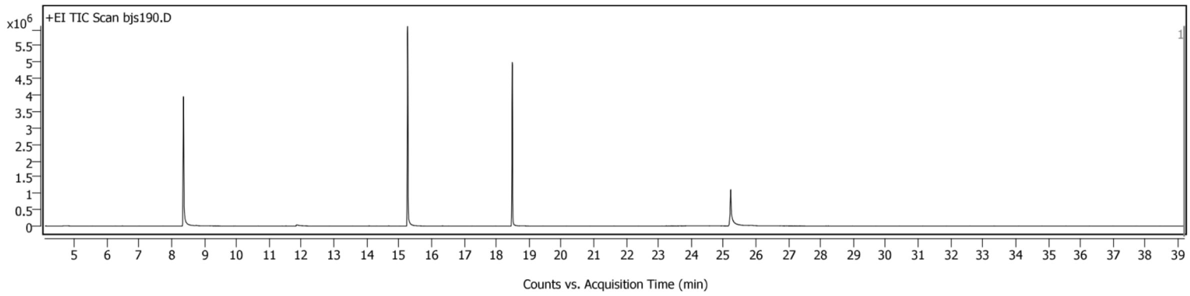

Sample Spectra

+ Scan (rt: 8.350-8.384 min) Sub

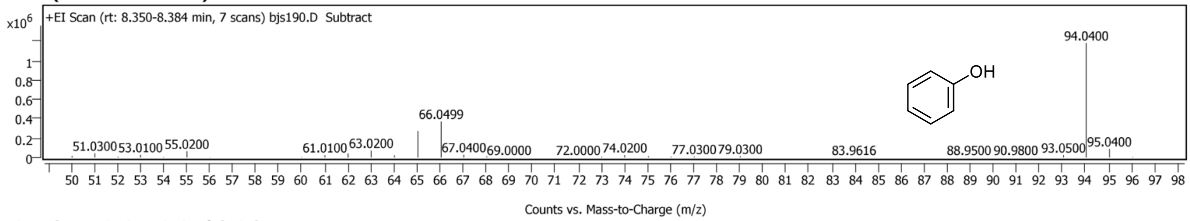

+ Scan (rt: 15.256-15.279 min) Sub

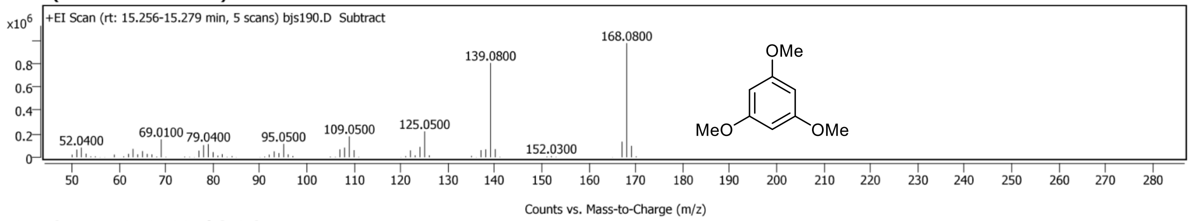

+ Scan (rt: 18.478-18.506 min) Sub

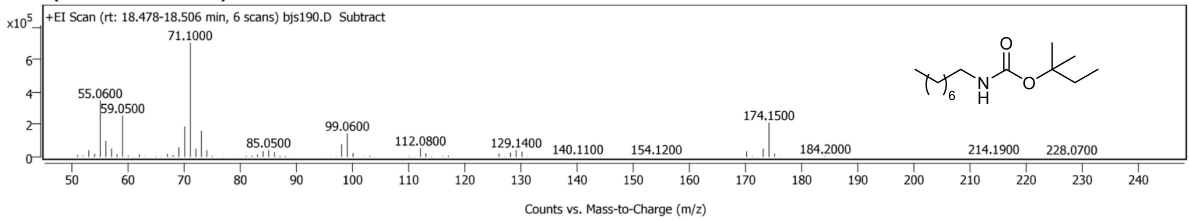

+ Scan (rt: 25.196-25.281 min) Sub

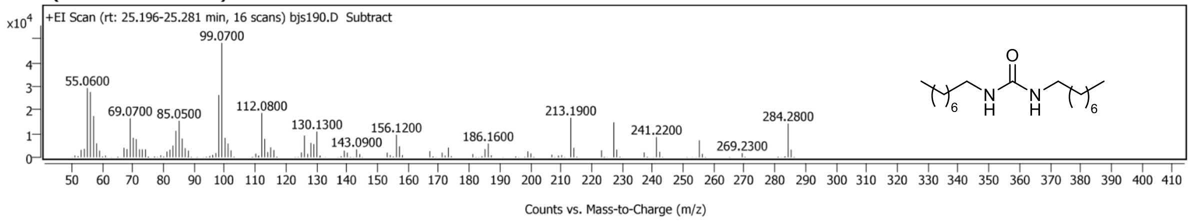

Figure 50: GC-MS data of entry 14 (Table S2).

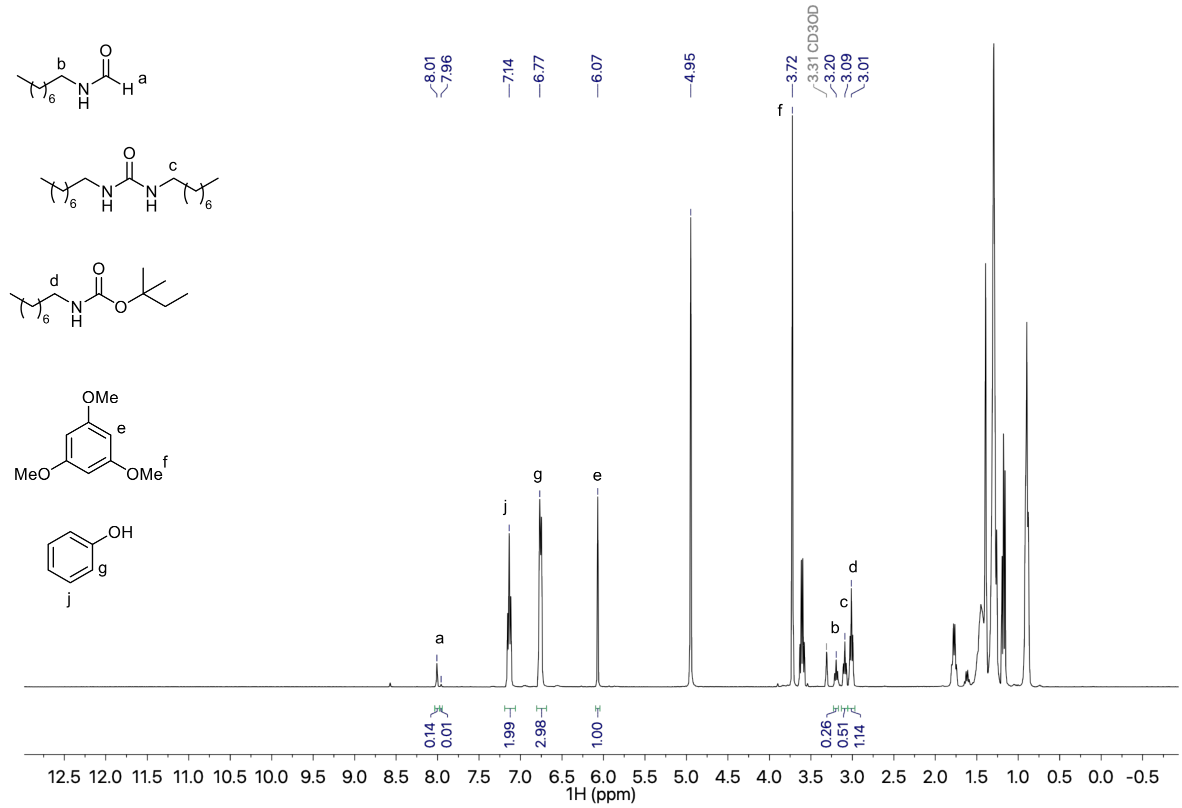

Figure 51: ¹H NMR (d⁴-MeOD, 500 MHz) spectrum of entry 15 (Table S2).

Sample Chromatograms

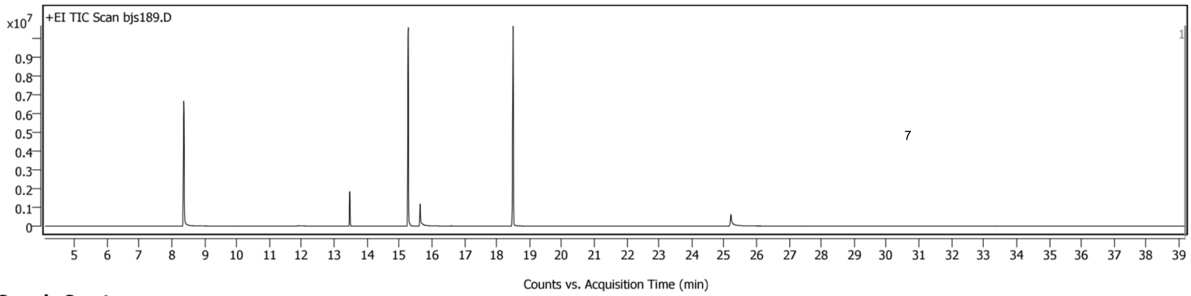

Sample Spectra

+ Scan (rt: 8.345-8.385 min) Sub

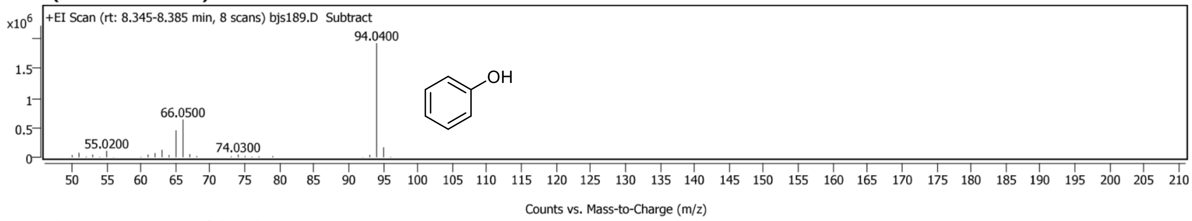

+ Scan (rt: 13.472-13.489 min) Sub

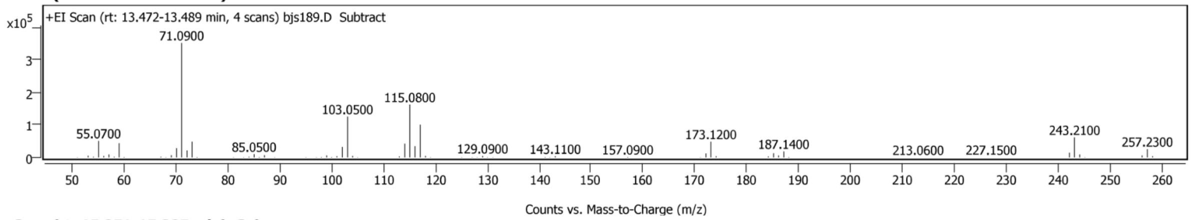

+ Scan (rt: 15.251-15.285 min) Sub

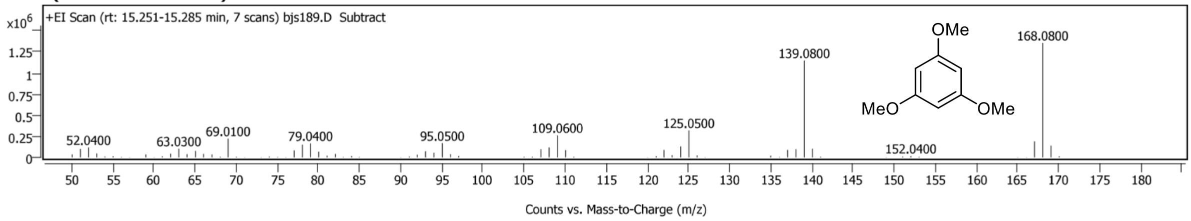

+ Scan (rt: 15.634-15.646 min) Sub

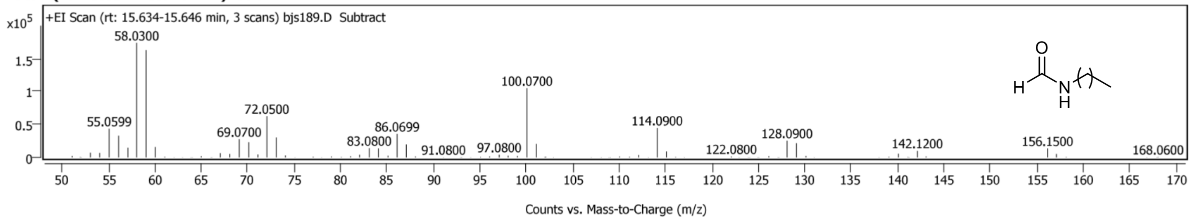

+ Scan (rt: 18.478-18.513 min) Sub

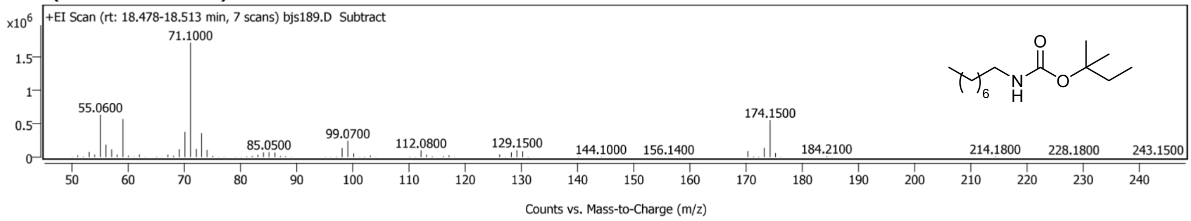

+ Scan (rt: 25.185-25.265 min) Sub

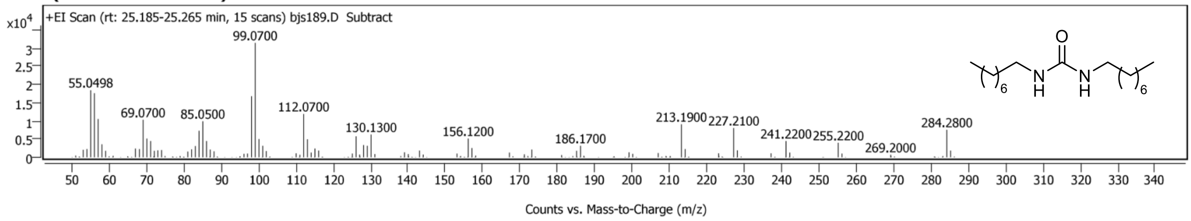

Figure 52: GC-MS data of entry 15 (Table S2).

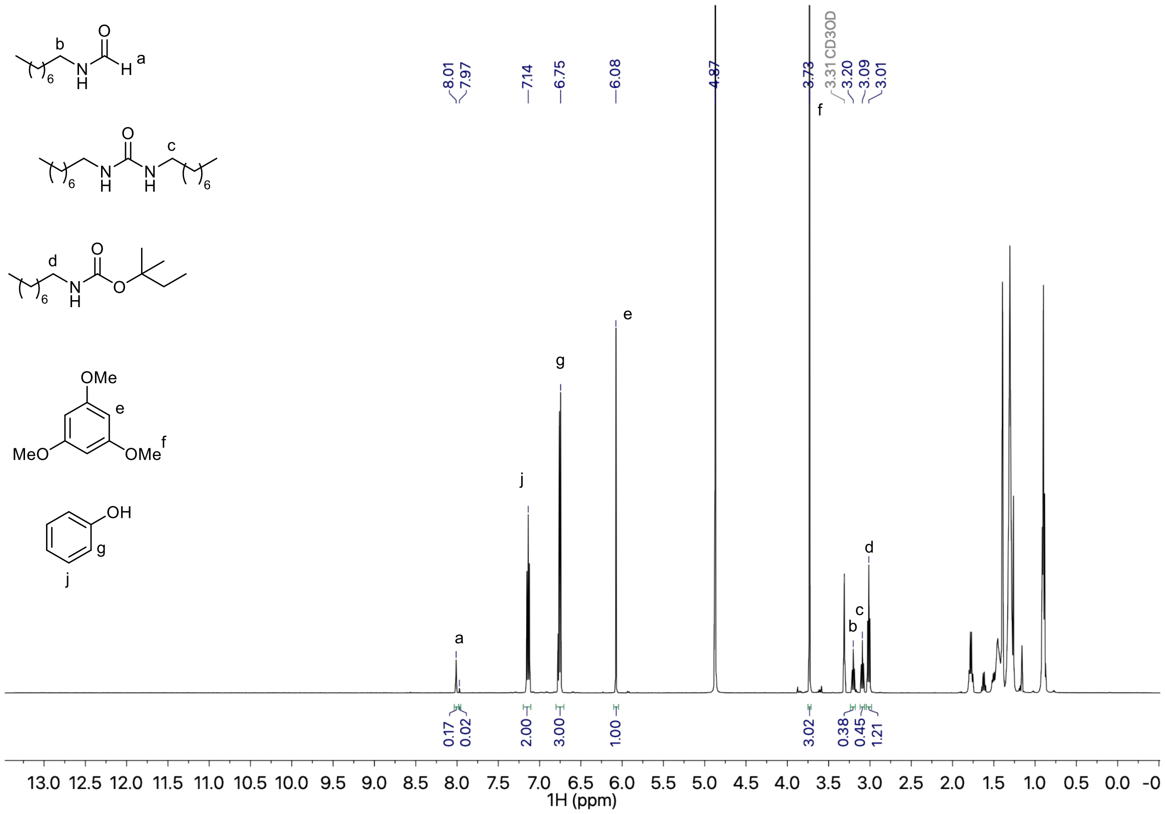

Figure 53:^{1}H NMR (d[4]-MeOD, 500 MHz) spectrum of entry 16 (Table S2).

Sample Chromatograms

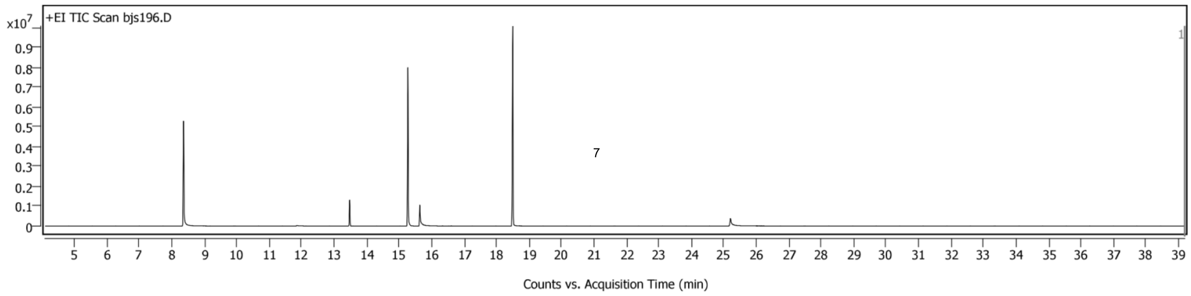

Sample Spectra

+ Scan (rt: 8.344-8.379 min) Sub

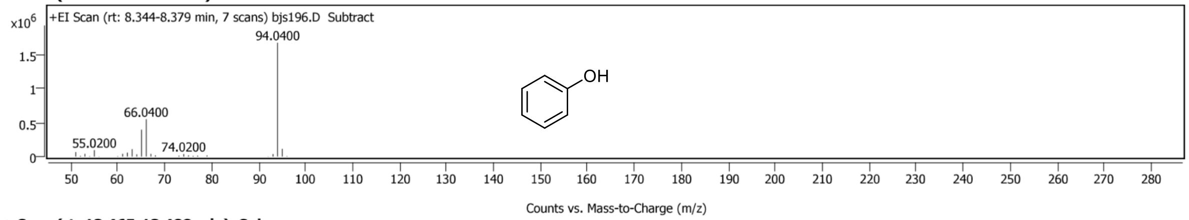

+ Scan (rt: 13.465-13.488 min) Sub

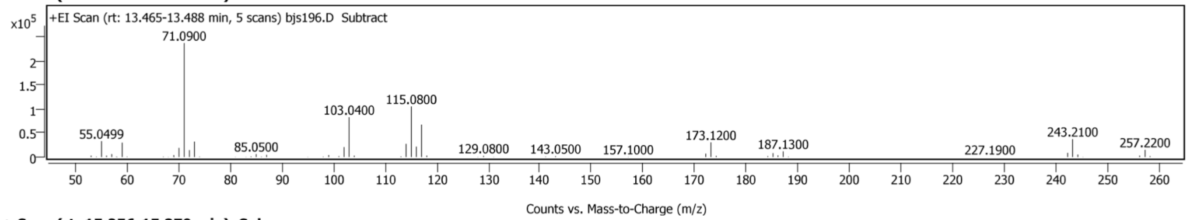

+ Scan (rt: 15.256-15.279 min) Sub

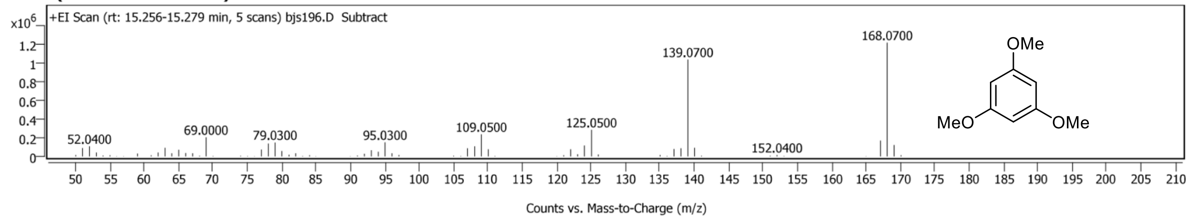

+ Scan (rt: 15.623-15.651 min) Sub

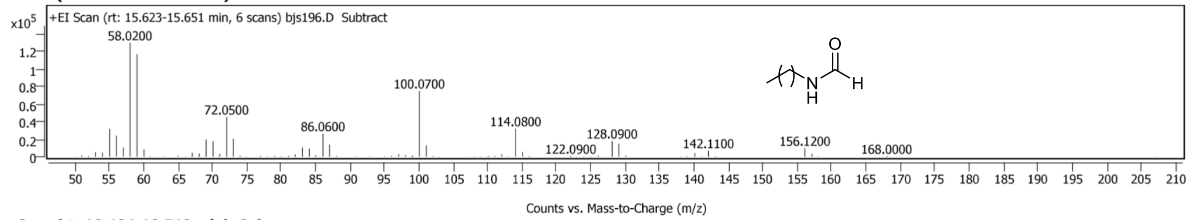

+ Scan (rt: 18.484-18.512 min) Sub

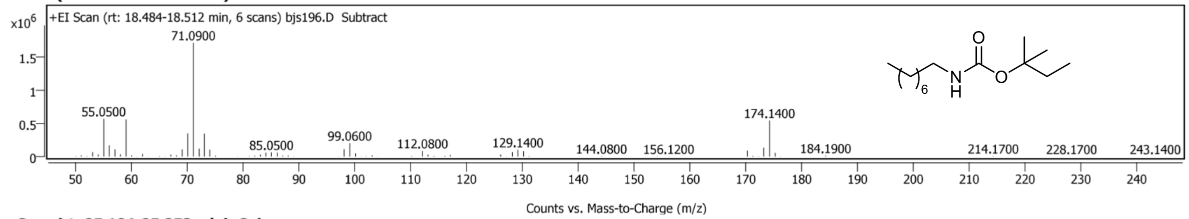

+ Scan (rt: 25.184-25.253 min) Sub

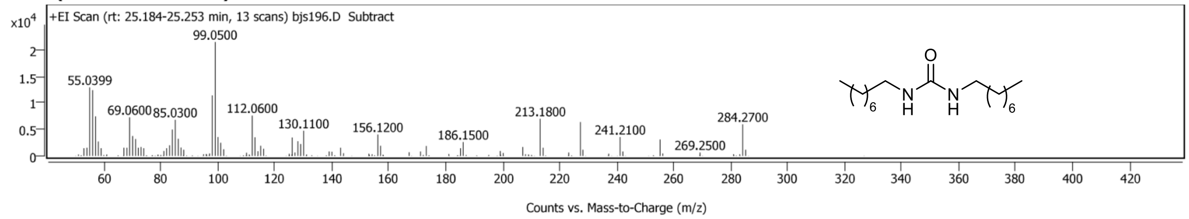

Figure 54: GC-MS data of entry 16 (Table S2).

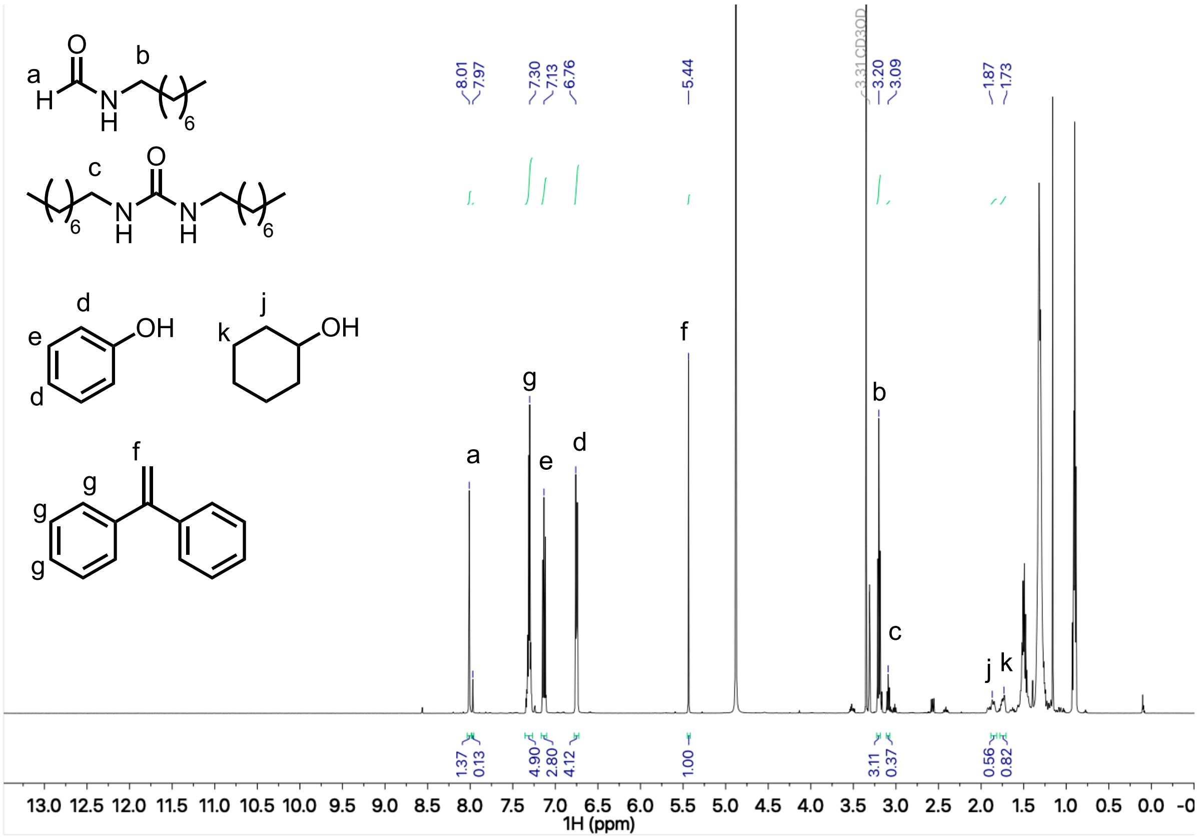

Figure 55: ^{1}H NMR (d[4]-MeOD, 500 MHz) spectrum of entry 17 (Table S2).

Sample Chromatograms

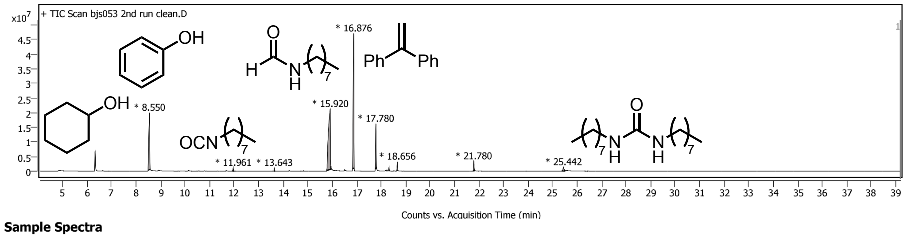

Sample Spectra

+ Scan (rt: 6.313-6.347 min) Sub

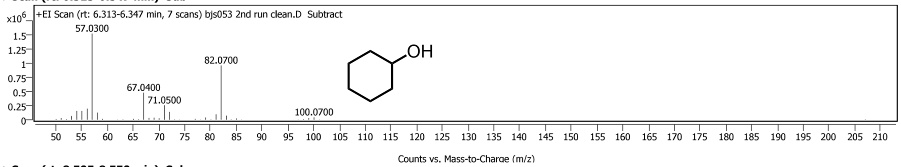

+ Scan (rt: 8.505-8.550 min) Sub

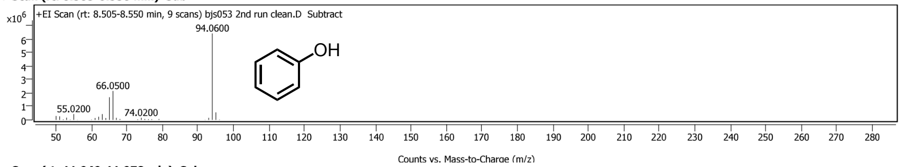

+ Scan (rt: 11.949-11.978 min) Sub

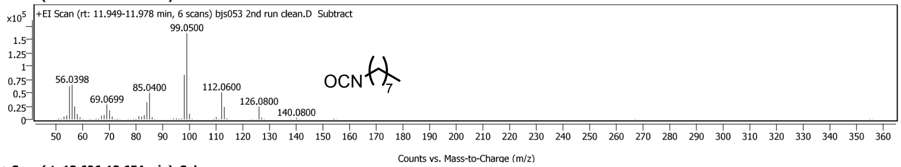

+ Scan (rt: 13.626-13.654 min) Sub

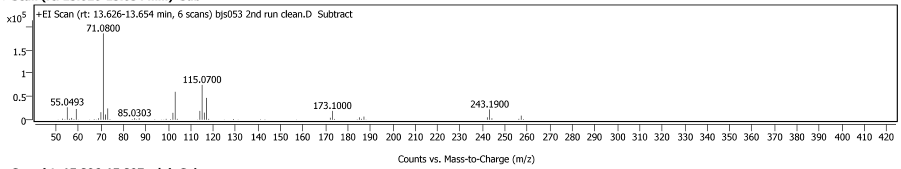

+ Scan (rt: 15.806-15.897 min) Sub

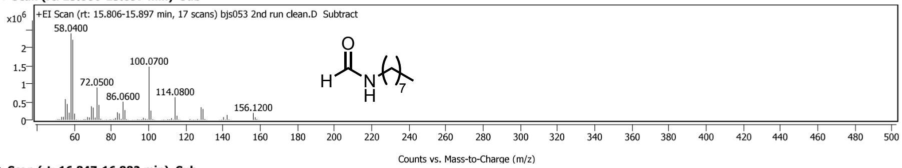

+ Scan (rt: 16.847-16.882 min) Sub

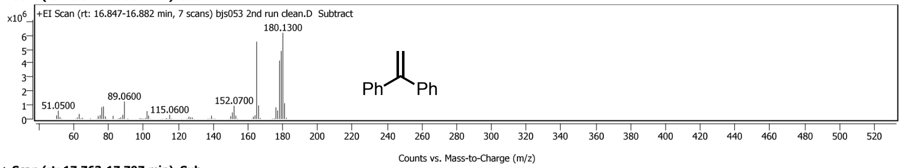

+ Scan (rt: 17.763-17.797 min) Sub

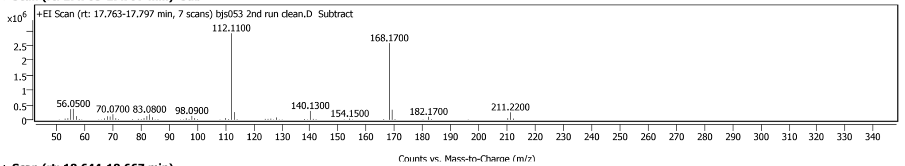

+ Scan (rt: 18.644-18.667 min)

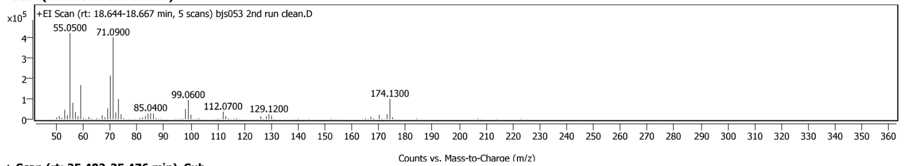

+ Scan (rt: 25.402-25.476 min) Sub

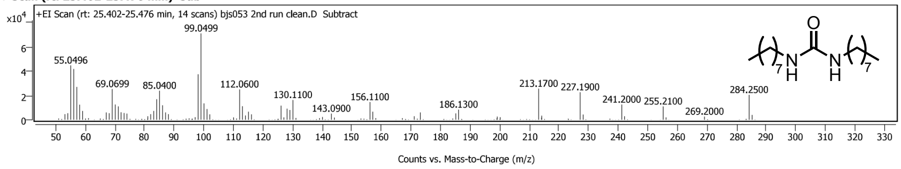

Figure 56: GC-MS data of entry 17 (Table S2). Unknown products at 13.6, 17.8 and 18.6 min in GC.

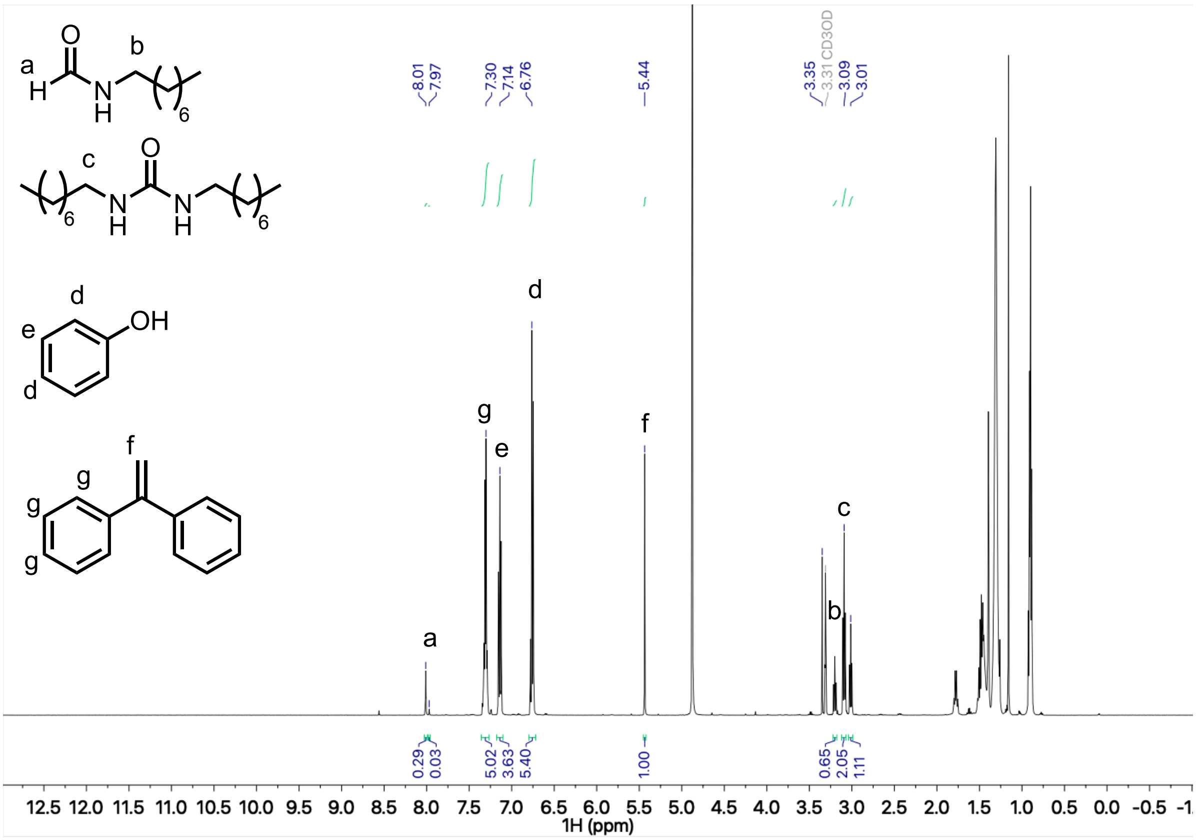

Figure 57: ^{1}H NMR (d^4-MeOD, 500 MHz) spectrum of entry 18 (Table S2).

Sample Chromatograms

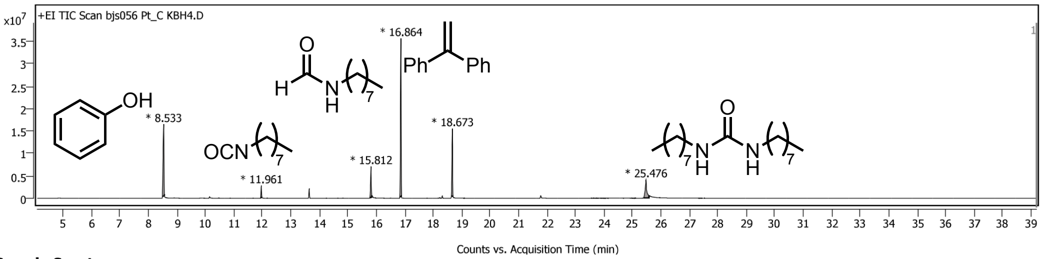

Sample Spectra

+ Scan (rt: 8.505-8.545 min) Sub

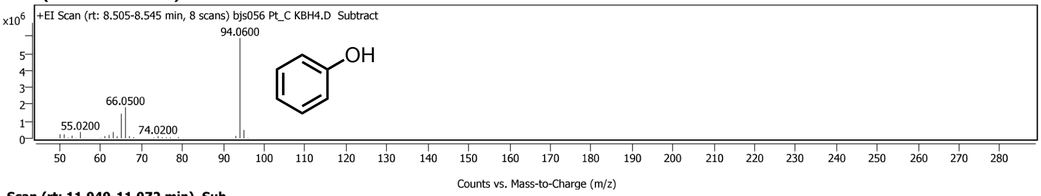

+ Scan (rt: 11.949-11.972 min) Sub

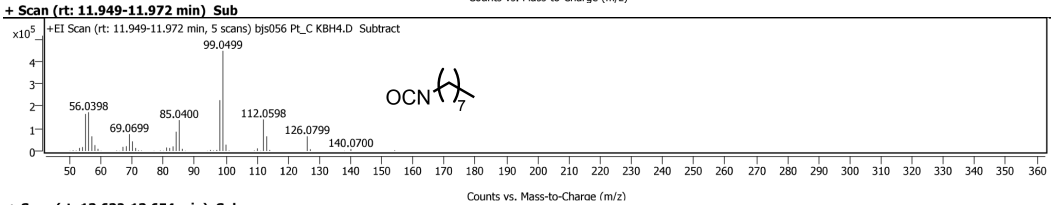

+ Scan (rt: 13.632-13.654 min) Sub

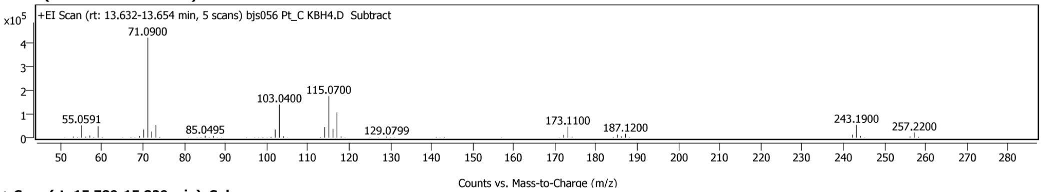

+ Scan (rt: 15.789-15.829 min) Sub

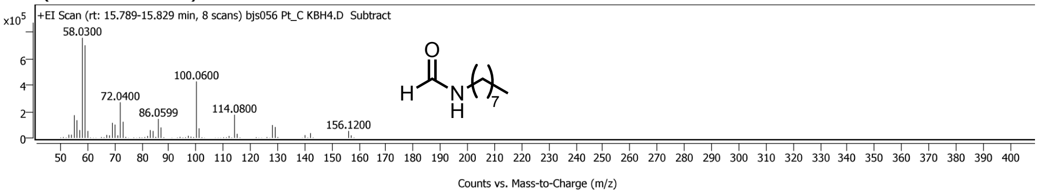

+ Scan (rt: 25.430-25.533 min) Sub

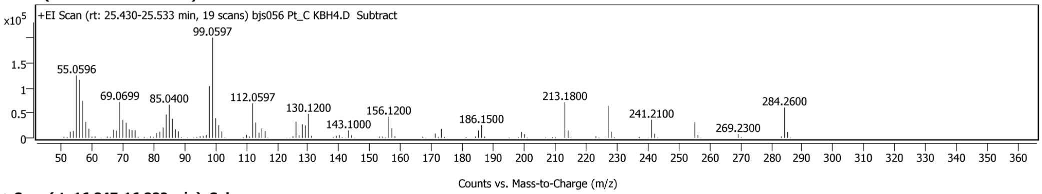

+ Scan (rt: 16.847-16.882 min) Sub

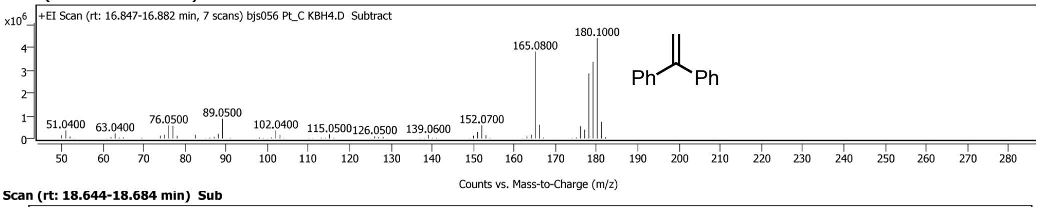

+ Scan (rt: 18.644-18.684 min) Sub

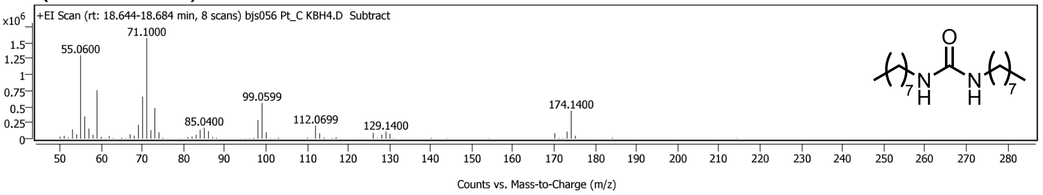

Figure 58: GC-MS data of entry 18 (Table S2). Unknown products at 13.6 and 18.6 min in GC

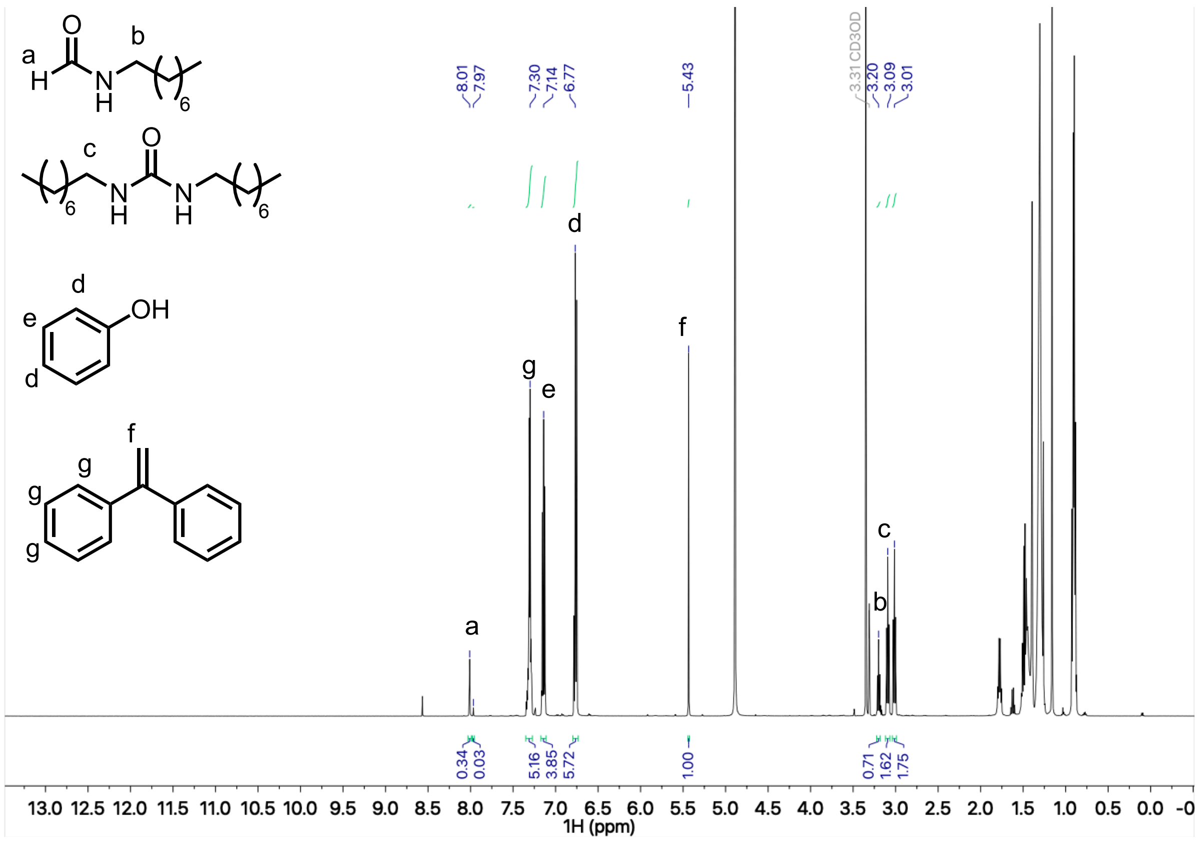

Figure 59: ^{1}H NMR (d^{4}-MeOD, 500 MHz) spectrum of entry 19 (Table S2).

Sample Chromatograms

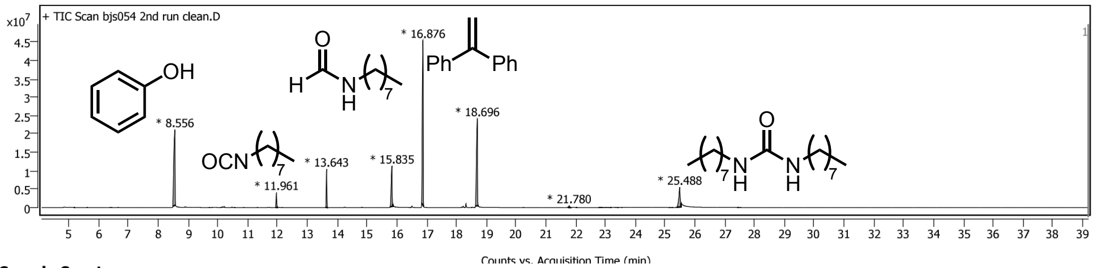

Sample Spectra

+ Scan (rt: 8.505-8.551 min) Sub

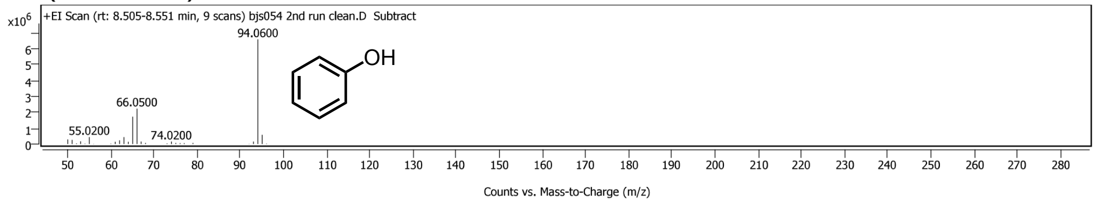

+ Scan (rt: 11.949-11.978 min) Sub

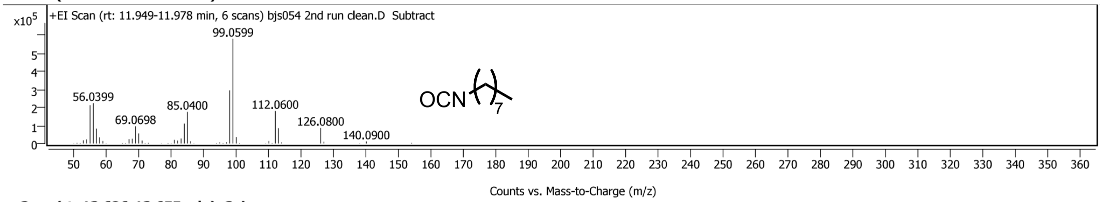

+ Scan (rt: 13.626-13.655 min) Sub

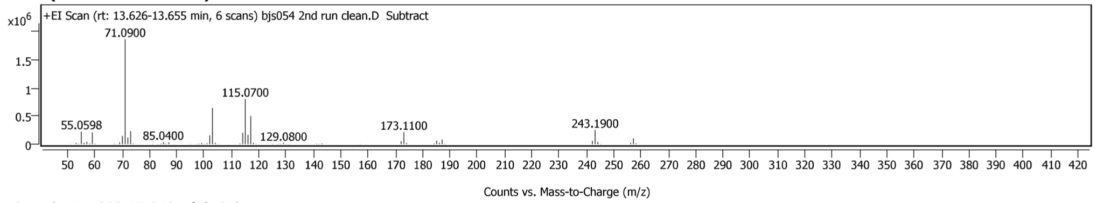

+ Scan (rt: 15.800-15.840 min) Sub

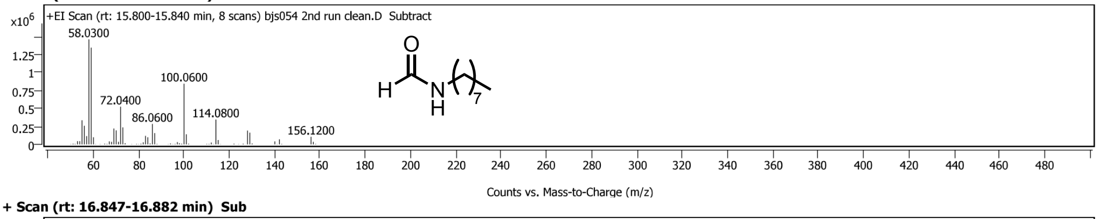

+ Scan (rt: 16.847-16.882 min) Sub

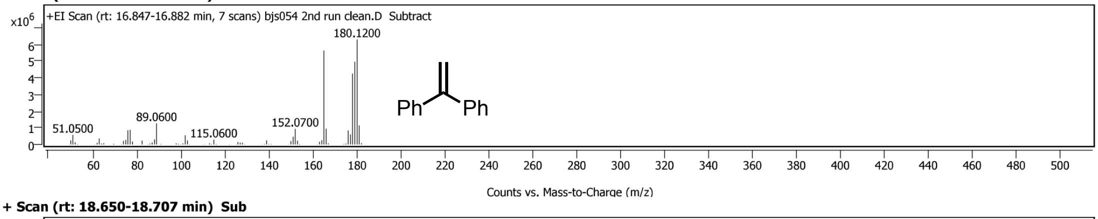

+ Scan (rt: 18.650-18.707 min) Sub

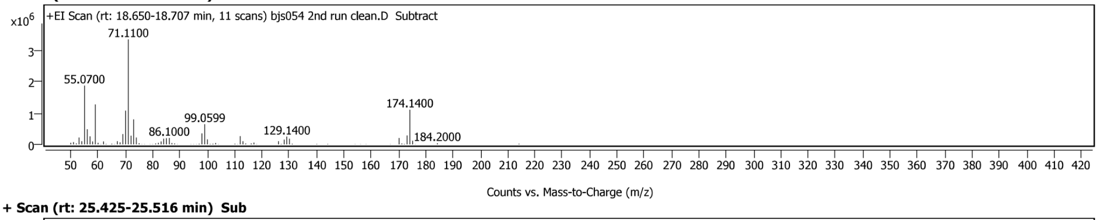

+ Scan (rt: 25.425-25.516 min) Sub

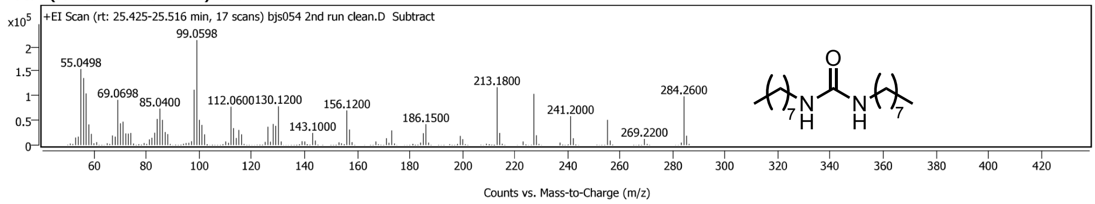

Figure 60: GC-MS data of entry 19 (Table S2). Unknown products at 13.6 and 18.6 min in the GC.

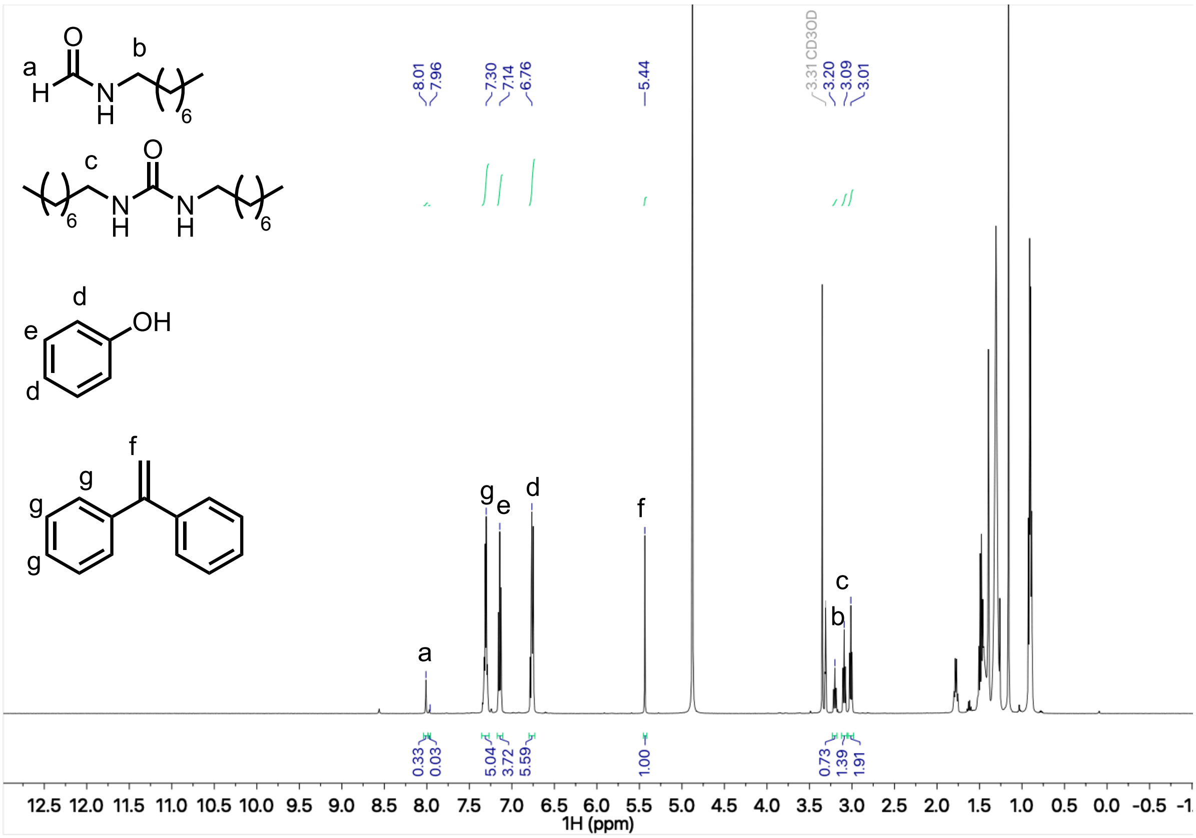

Figure 61: ^{1}H NMR (d[4]-MeOD, 500 MHz) spectrum of entry 20 (Table S2).

Sample Chromatograms

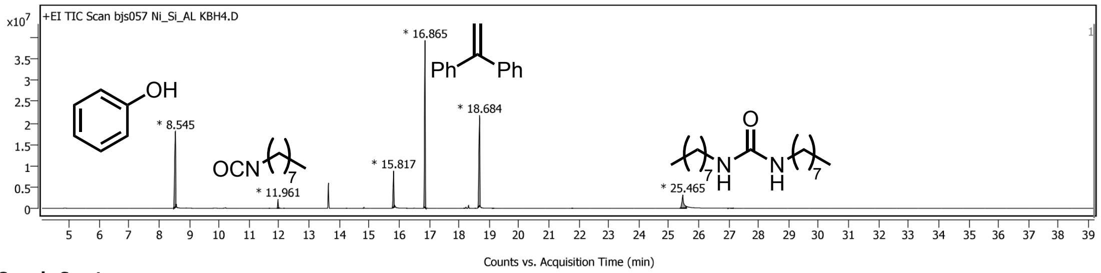

Sample Spectra

+ Scan (rt: 8.505-8.545 min) Sub

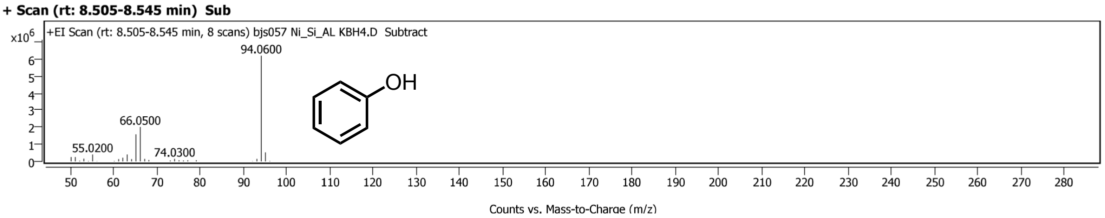

+ Scan (rt: 11.949-11.972 min) Sub

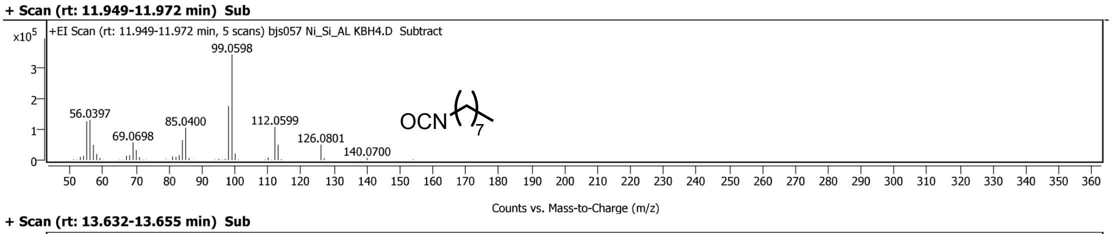

+ Scan (rt: 13.632-13.655 min) Sub

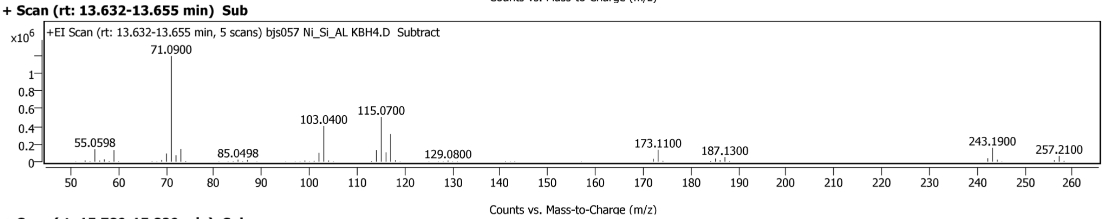

+ Scan (rt: 15.789-15.829 min) Sub

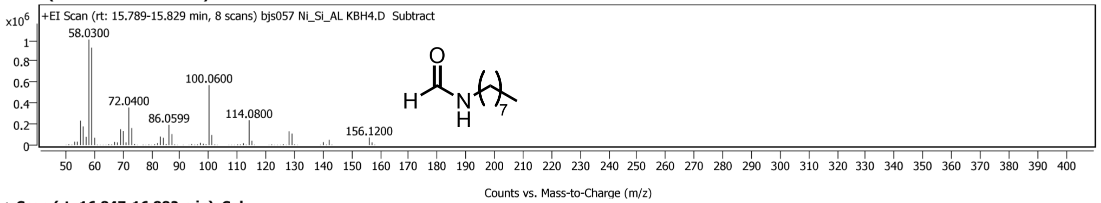

+ Scan (rt: 16.847-16.882 min) Sub

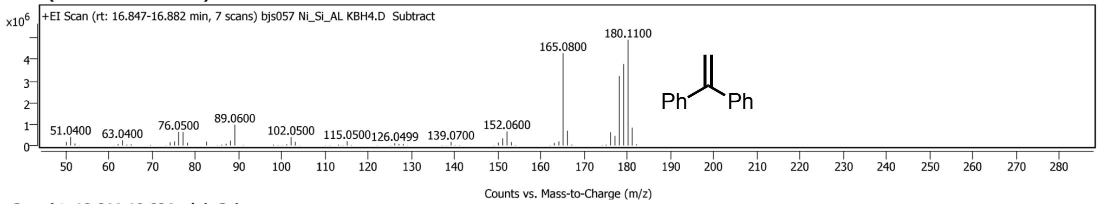

+ Scan (rt: 18.644-18.684 min) Sub

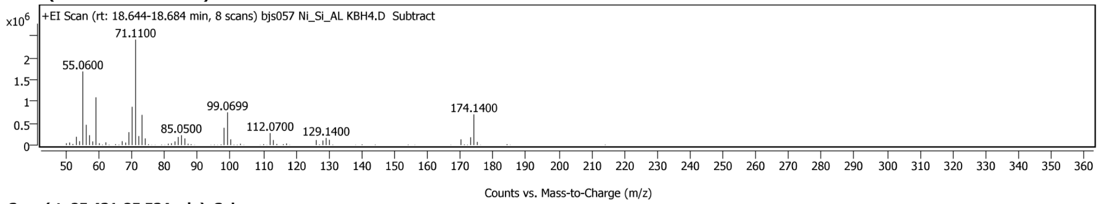

+ Scan (rt: 25.431-25.534 min) Sub

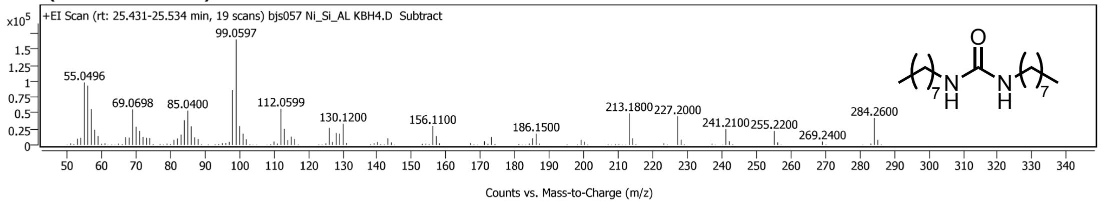

Figure 62: GC-MS data of entry 20 (Table S2). Unknown products at 13.6 and 18.6 min in the GC.

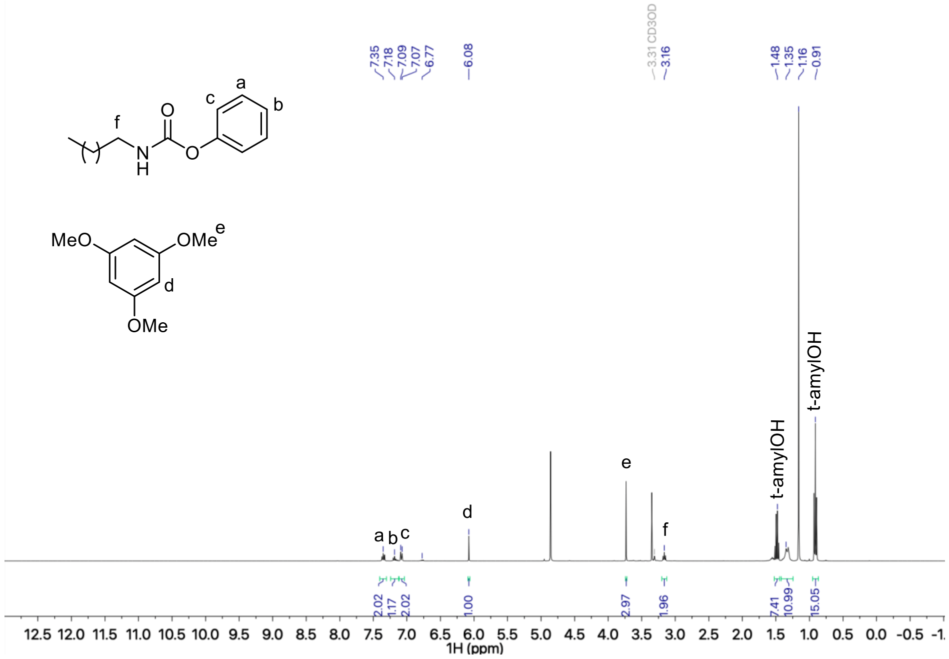

Figure 63: ^{1}H NMR (d[4]-MeOD, 500 MHz) spectrum of entry 21 (Table S2).

Sample Chromatograms

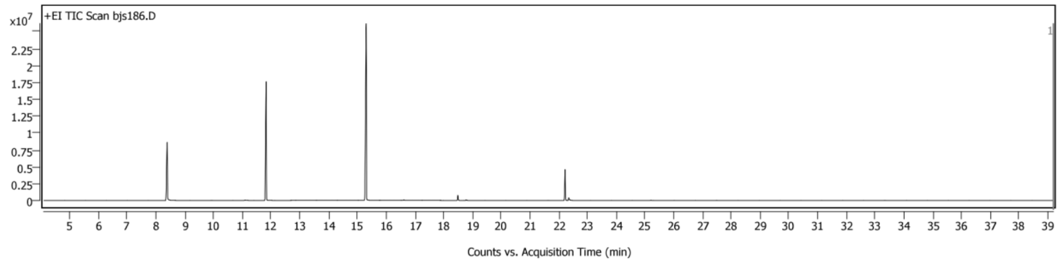

Sample Spectra

+ Scan (rt: 8.361-8.396 min) Sub

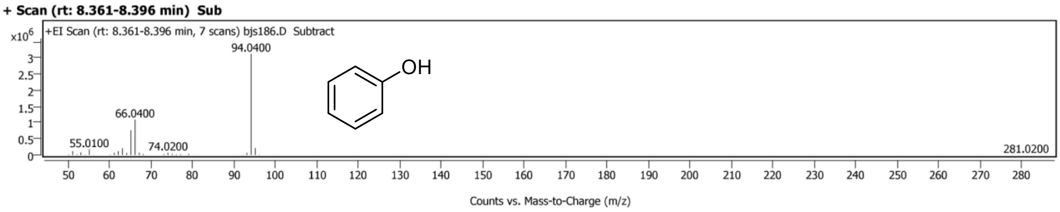

+ Scan (rt: 11.806-11.835 min) Sub

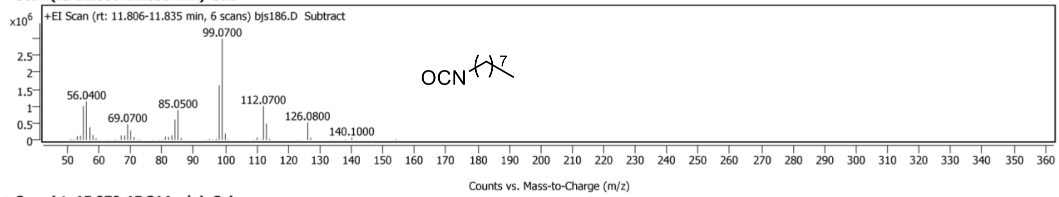

+ Scan (rt: 15.279-15.314 min) Sub

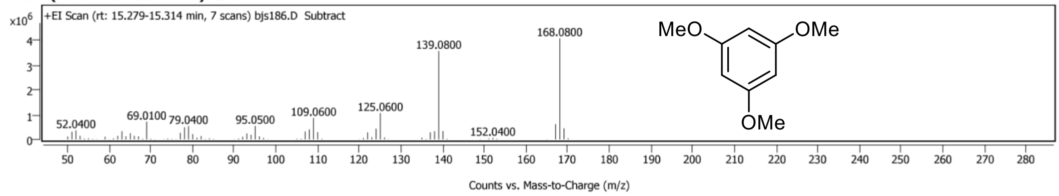

+ Scan (rt: 22.197-22.232 min) Sub

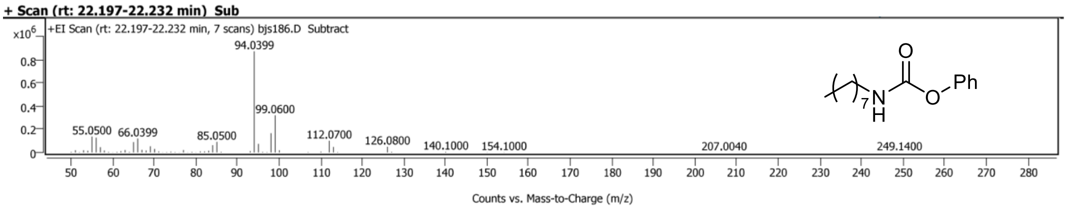

Figure 64: GC-MS data of entry 21 (Table S2).

4. Hydrogenation of carbamates and ureas

4.1 Procedure for the hydrogenation of carbamates and ureas

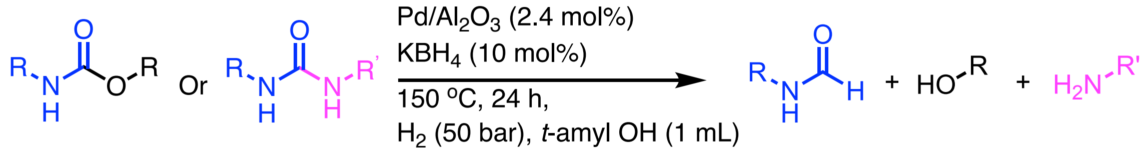

Figure 56: General Scheme for the hydrogenation of carbamates or ureas.

The carbamate or urea (1 mmol), Pd/Al_2O_3 (2.4 mol%), and KBH_4 (10 mol%) were weighed under air and transferred to a microwave vial (5 mL) containing a stirrer bar. The vial was sealed using a septum and degassed under argon by placing a needle through the septum. Dry and degassed *t*-amyl alcohol (1 mL) was transferred through a syringe to the vial. The septum was pierced with two needles and placed in a stainless-steel autoclave under an argon atmosphere. The autoclave was sealed and degassed three times using H_2 gas. Upon degassing, the autoclave was pressurized with 50 bar of H_2 gas. The autoclave was placed in a preheated oil bath at 150 °C and left for 24 hours. After completion of the reaction time, the autoclave was cooled in cold water for 30 minutes, and hydrogen gas was slowly vented off. Trimethoxybenzene (0.33 mmol) or 1,1'-diphenylethylene (0.25-0.5 mmol) was added as internal standard. Yields of formamide we calculated from formamide peaks around 8-9 ppm. Conversion was calculated by the reduction of starting material peaks in the ^{1}H NMR spectroscopy. Alcohol or amine yields were calculated based on their corresponding alkyl peaks in ^{1}H NMR spectroscopy.

4.2 Analytical data for the hydrogenation of carbamates and ureas

Table S3: Optimization for catalytic hydrogenation of carbamates and ureas

Entry	Carbamate/Urea	Conversion	Formamide Yield	R'XH Yield
1	Phenyl *N*-octylcarbamate	100%	83%	85%
2	Phenyl *N*-cyclohexylcarbamate	100%	81%	81%
3	diphenylcarbamate	100%	57%	86%
4	Methyl *N*-phenyl carbamate	64%	18%	25%
5	Phenyl urethane	69%	29%	25%
6	Phenyl (6-hydroxyhexyl)carbamate	95%	64%	76%
7	Phenyl N-benzylcarbamate	89%	71%	77%
8	Phenyl N-(4-methoxy)phenyl carbamate	79%	78%	62%
9	Diphenyl 1,4-pehnylenedicarbamate	10%	5%	10%
10	Diphenyl urea	88%	85%	73%
11	1,3-bis(4-fluorophenyl) urea	96%	94%	67%
12	1,3-bis(4-methoxyphenyl) urea	26%	12%	34%
13	Dibenzyl urea	38%	21%	-

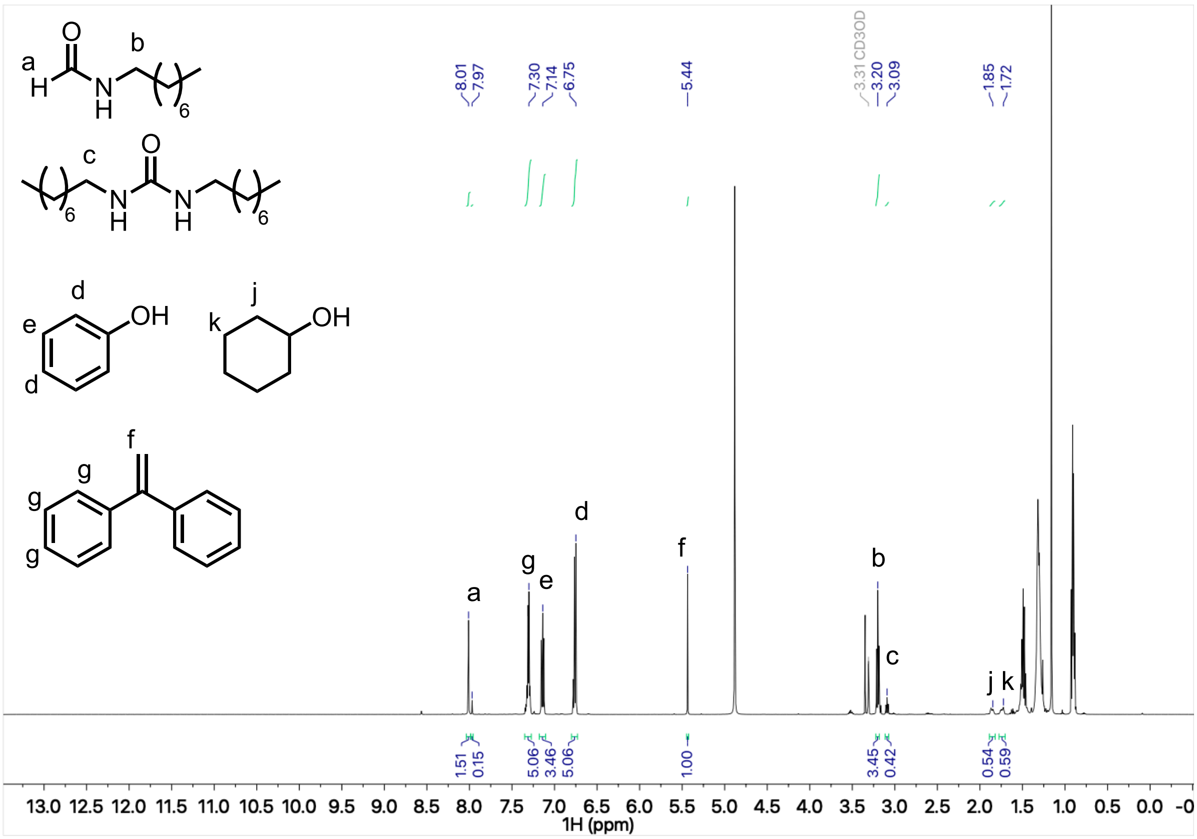

Figure 65: ^{1}H NMR (d^4-MeOD, 500 MHz) spectrum of entry 1 (Table S3). 1,1'-diphenylethylene (0.25 mmol) was used as internal standard. The NMR data of the formamide[3] and urea derivative[4] shown here match well with the literature.

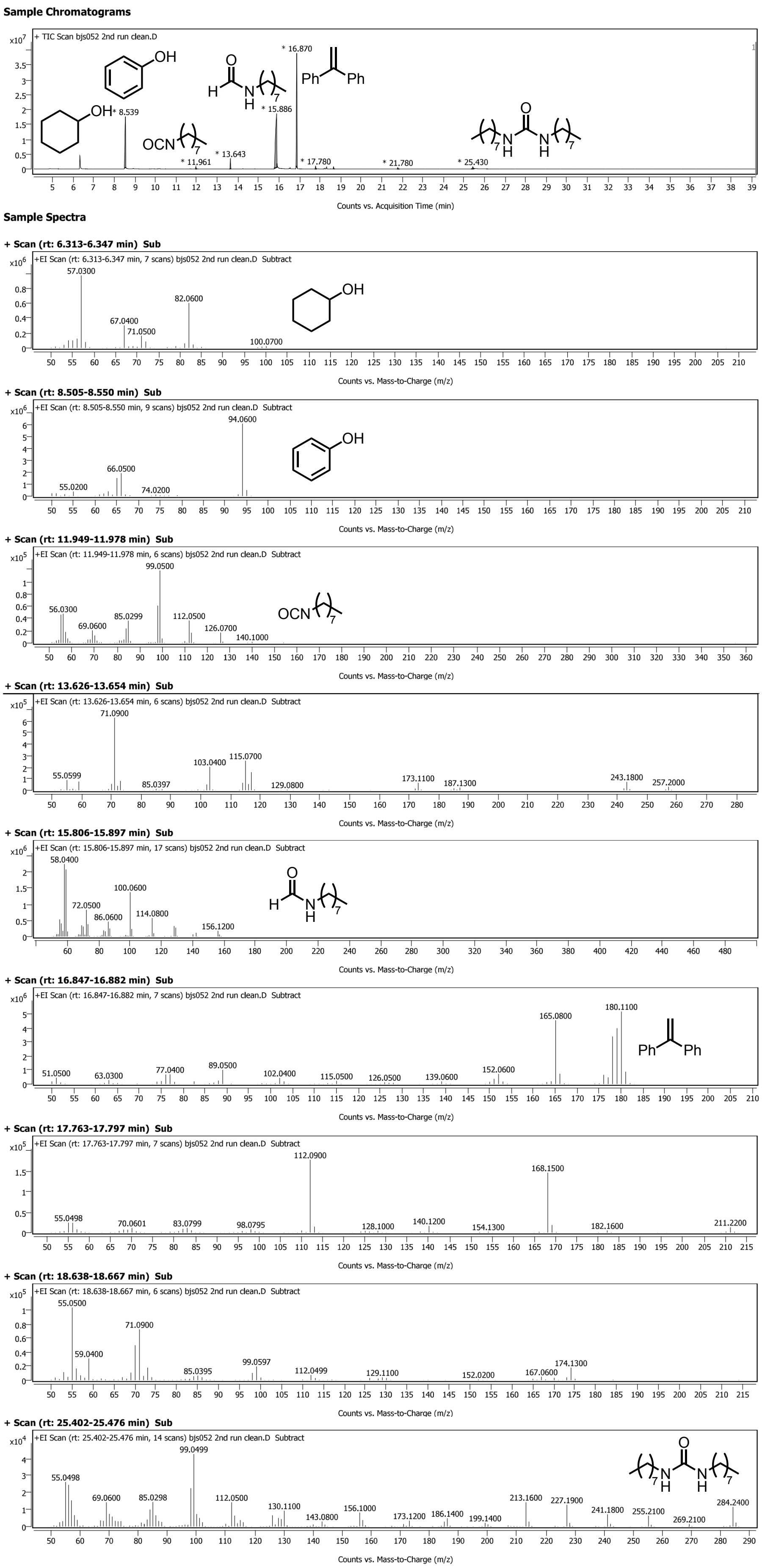

Figure 66: GC-MS data of entry 1 (Table S3) with unknown products at 13.6 17.8 and 18.6 min

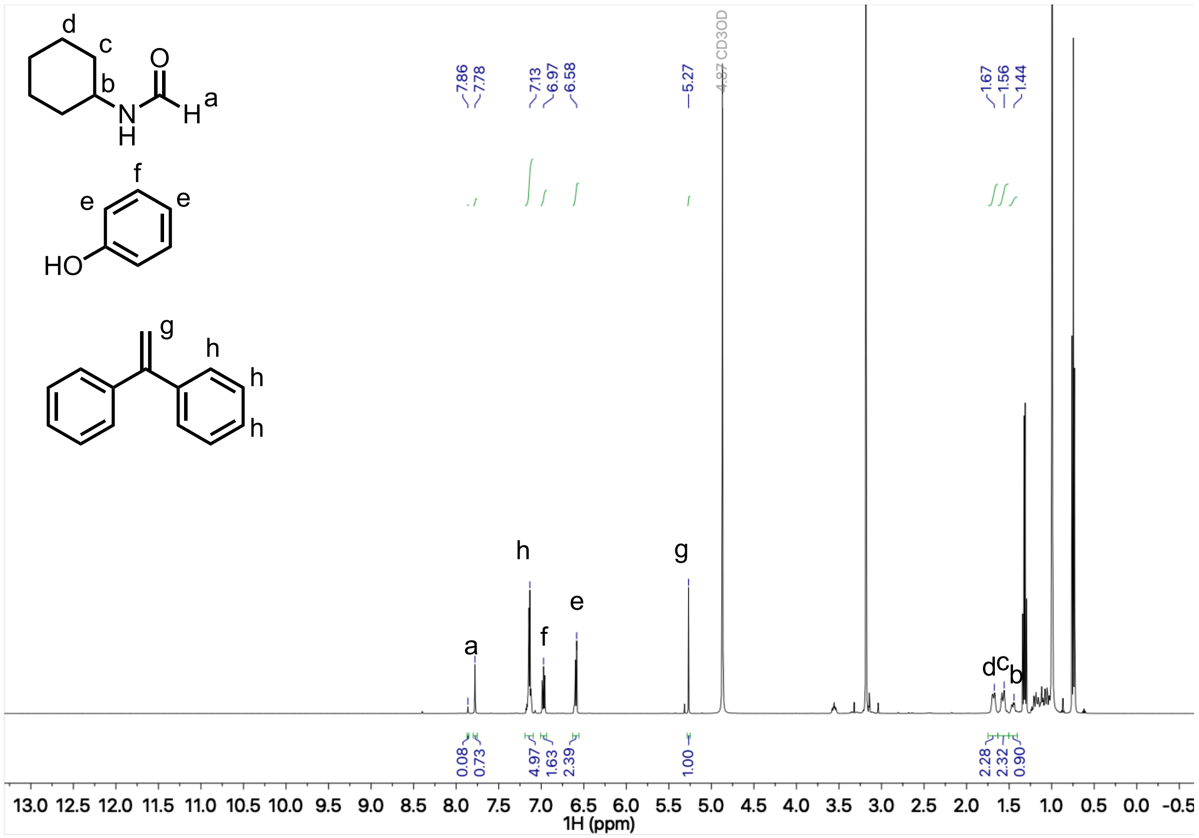

Figure 67: ^{1}H NMR (d^{4}-MeOD, 500 MHz) spectrum of entry 2 (Table S3). 1,1'-diphenylethylene (0.50 mmol) was used as internal standard. The NMR data of the formamide[3] shown here match well with the literature.

Sample Chromatograms

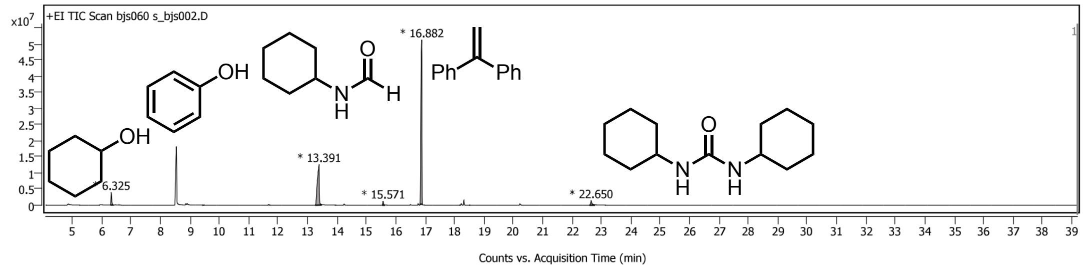

Sample Spectra

+ Scan (rt: 5.947-6.010 min) Sub

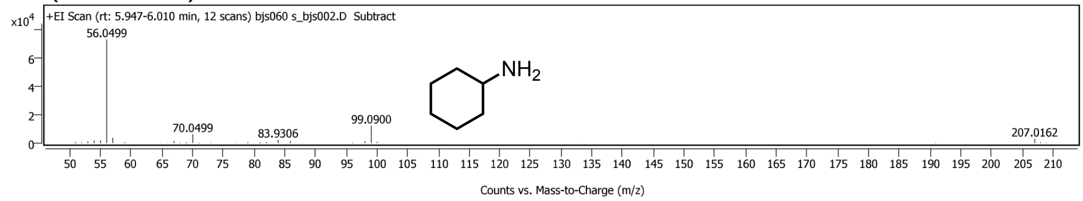

+ Scan (rt: 6.307-6.353 min) Sub

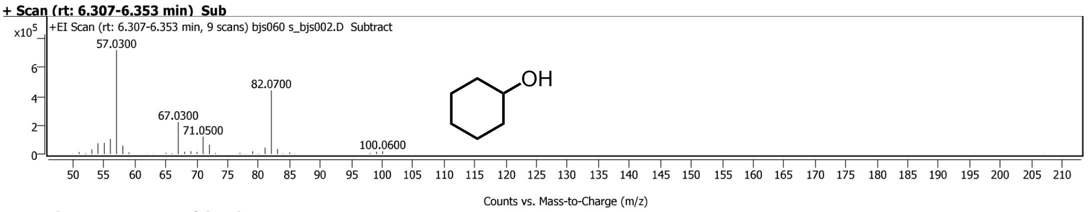

+ Scan (rt: 13.300-13.403 min) Sub

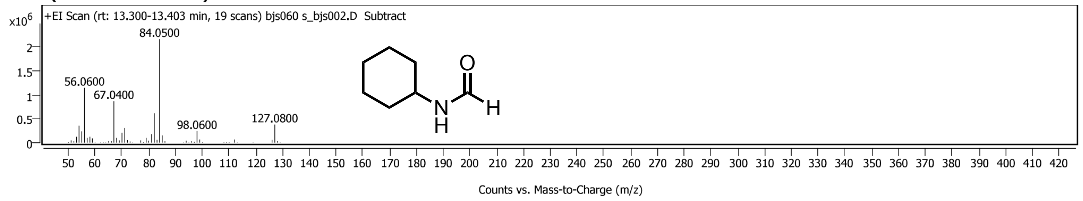

+ Scan (rt: 15.554-15.589 min) Sub

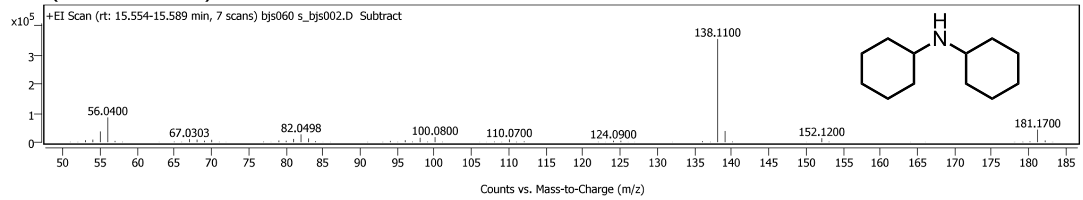

+ Scan (rt: 16.842-16.893 min) Sub

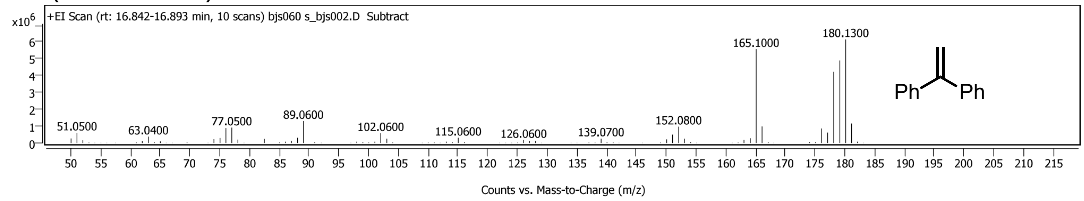

+ Scan (rt: 22.621-22.701 min) Sub

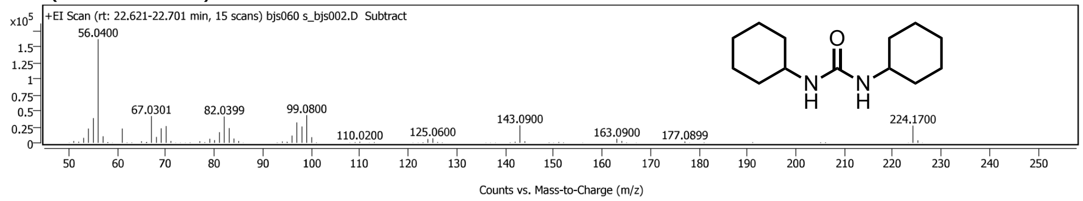

+ Scan (rt: 8.510-8.550 min) Sub

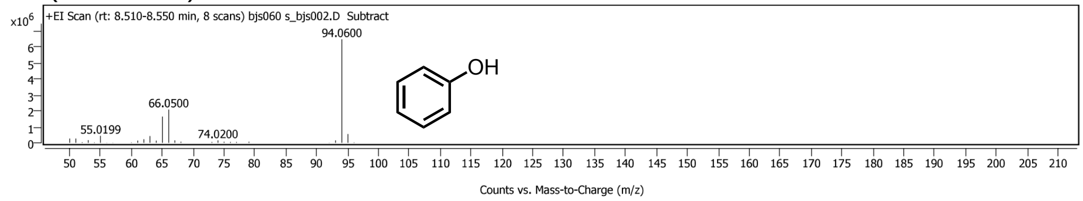

Figure 68: GC-MS data of entry 2 (Table S3). Trace cyclohexylamine, cyclohexanol and dicyclohexylamine.

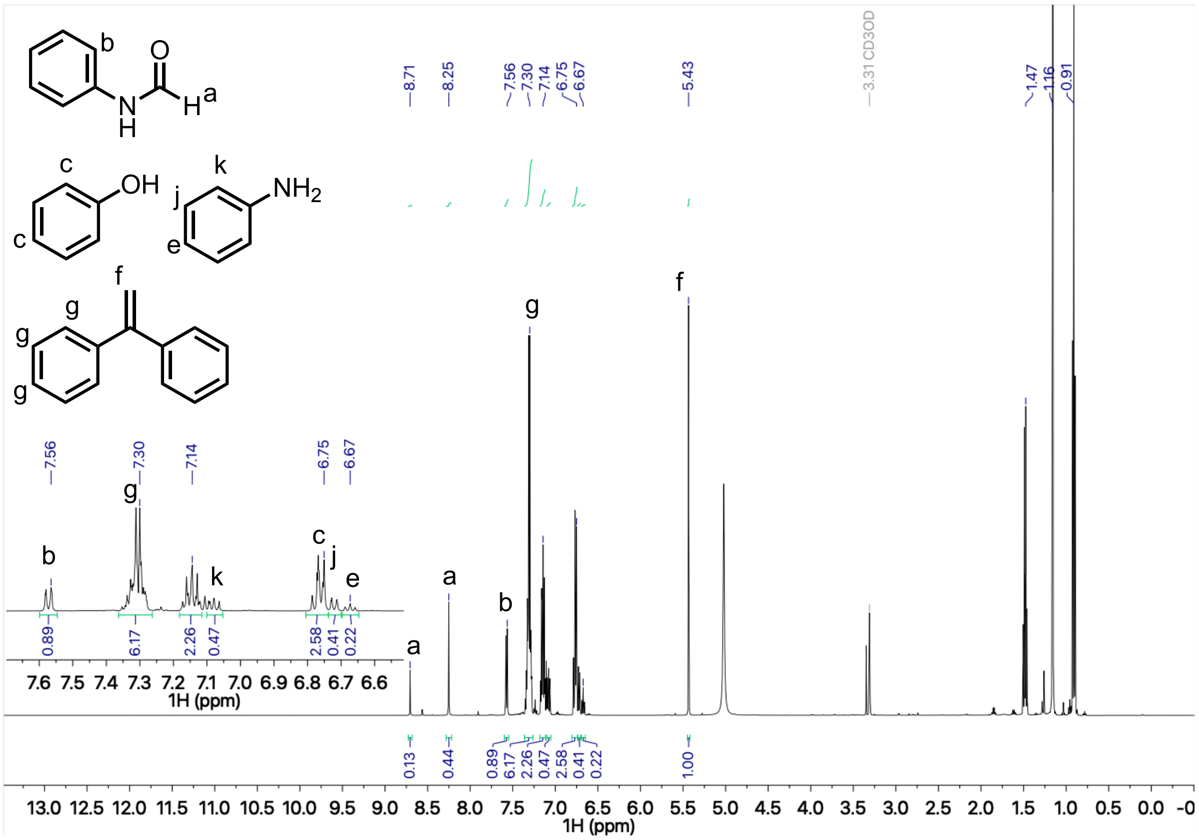

Figure 69: ^{1}H NMR (d[4]-MeOD, 500 MHz) spectrum of entry 3 (Table S3). 1,1'-diphenylethylene (0.50 mmol) was used as an internal standard. The NMR data of the formamide[1] shown here match well with the literature.

Sample Chromatograms

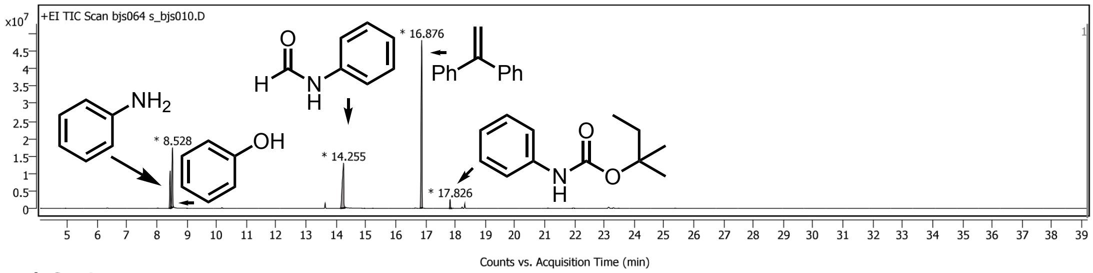

Sample Spectra

+ Scan (rt: 6.325-6.370 min) Sub

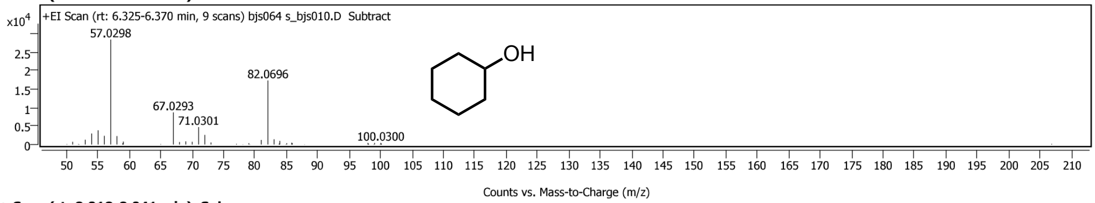

+ Scan (rt: 8.018-8.041 min) Sub

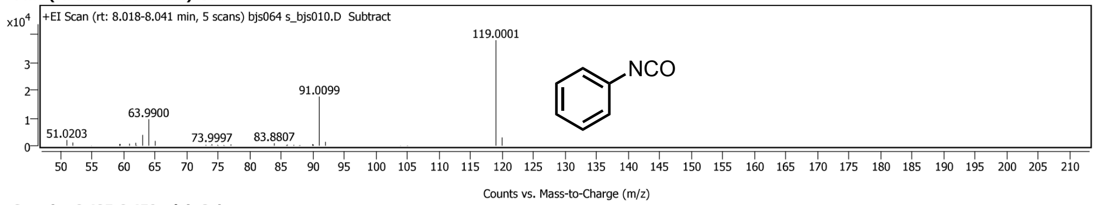

+ Scan (rt: 8.425-8.459 min) Sub

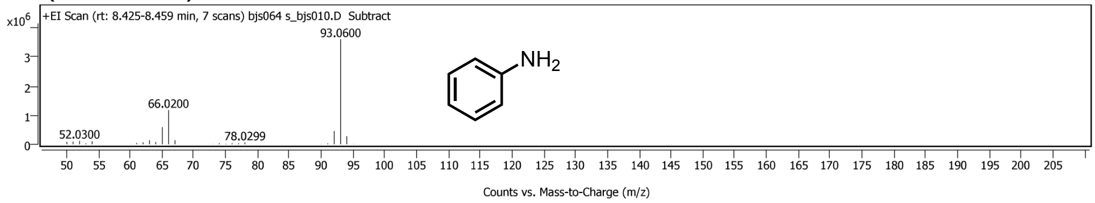

+ Scan (rt: 8.499-8.533 min) Sub

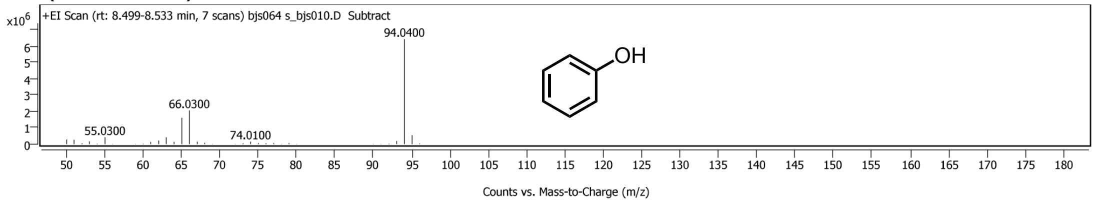

+ Scan (rt: 14.192-14.267 min) Sub

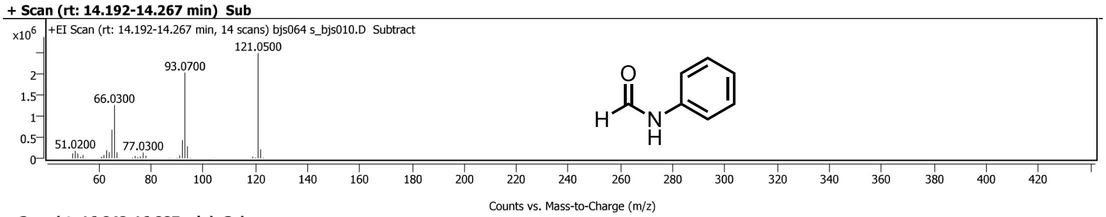

+ Scan (rt: 16.842-16.887 min) Sub

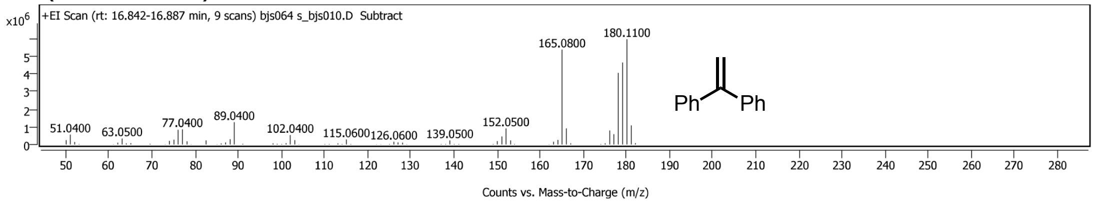

+ Scan (rt: 17.809-17.837 min) Sub

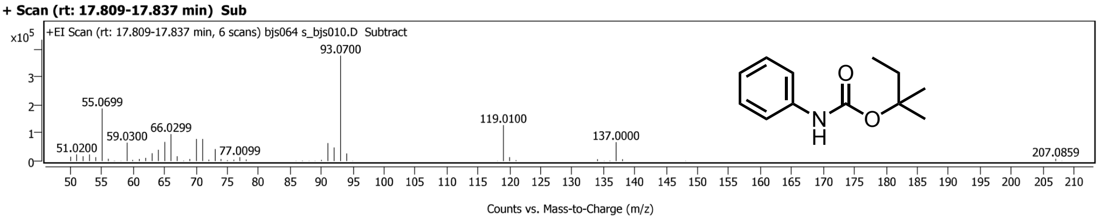

Figure 70: GC-MS data of entry 3 (Table S3). Trace cyclohexanol and phenylisocyanate.

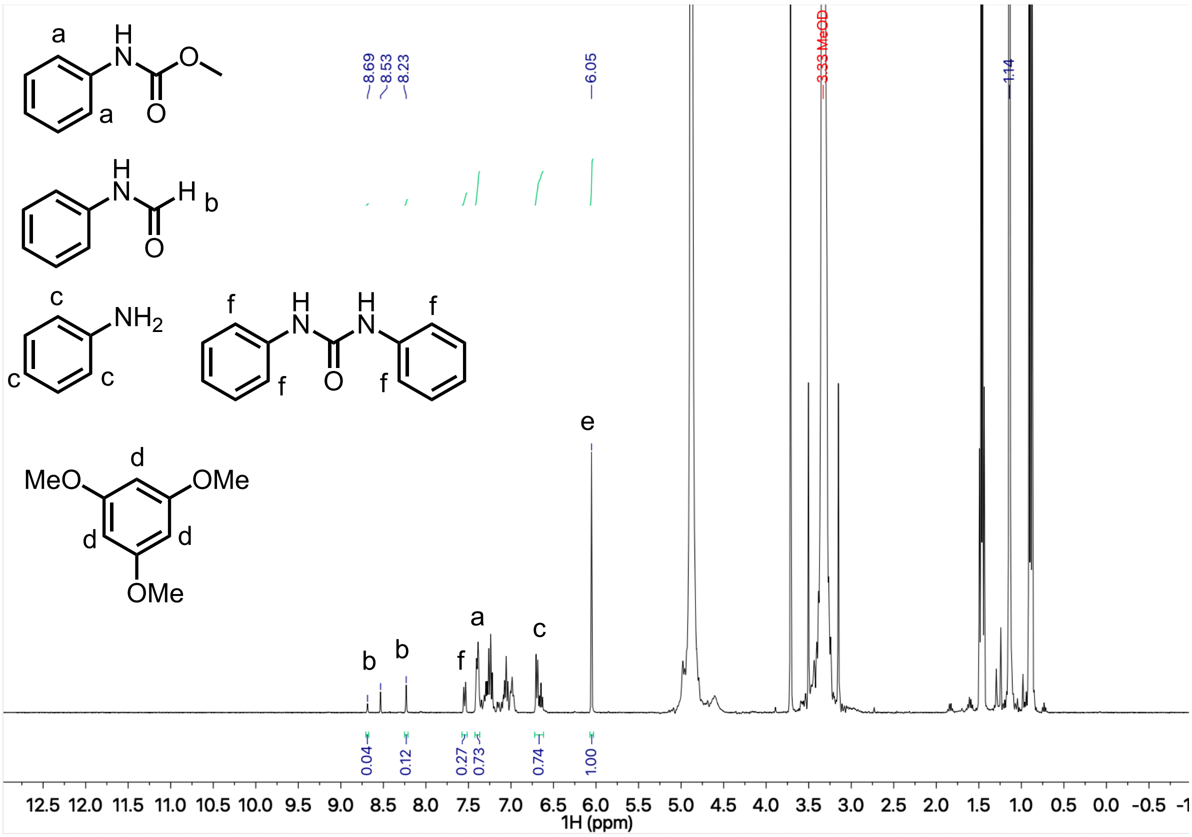

Figure 71: ^{1}H NMR (d^4-MeOD, 500 MHz) spectrum of entry 4 (Table S3). Trimethoxybenzene (0.33 mmol) was used as an internal standard. The NMR data of the formamide[1] and urea[5] shown here match well with the literature.

Sample Chromatograms

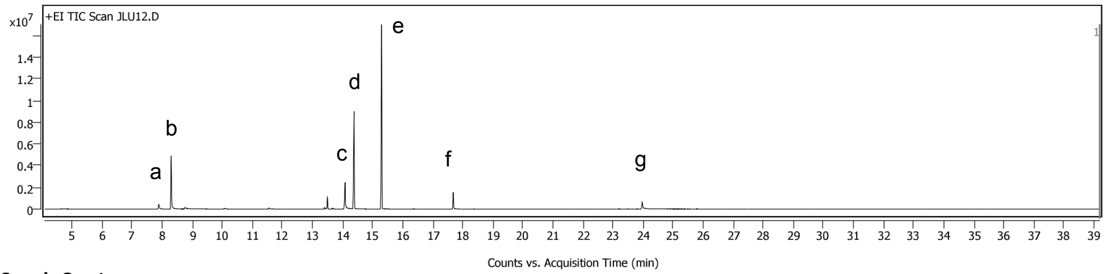

Sample Spectra

+ Scan (rt: 7.881-7.898 min) Sub

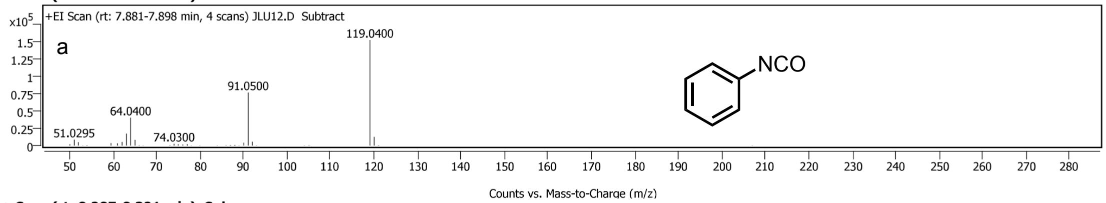

+ Scan (rt: 8.287-8.321 min) Sub

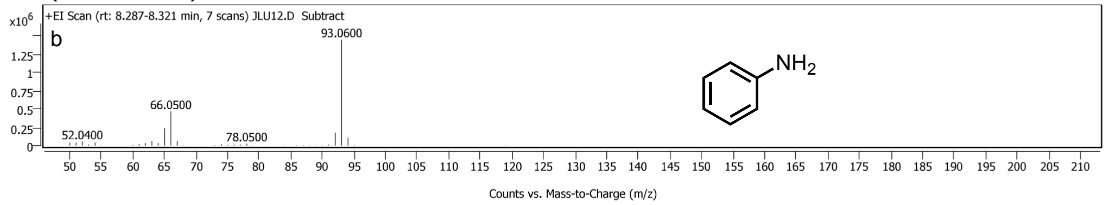

+ Scan (rt: 14.066-14.089 min) Sub

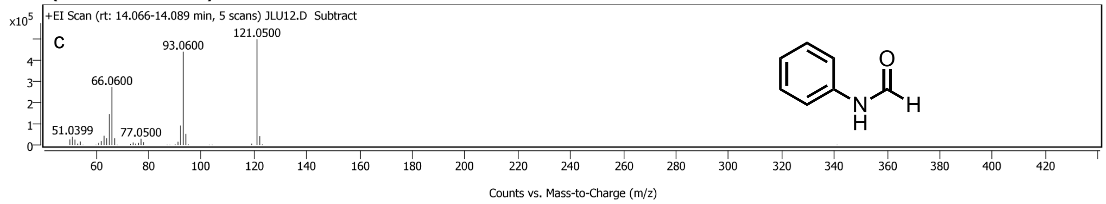

+ Scan (rt: 14.358-14.392 min) Sub

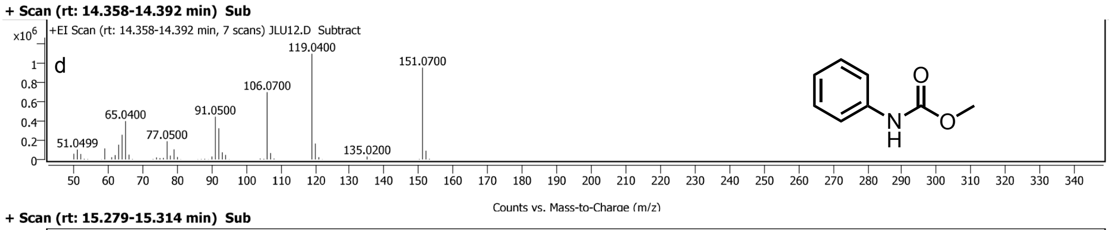

+ Scan (rt: 15.279-15.314 min) Sub

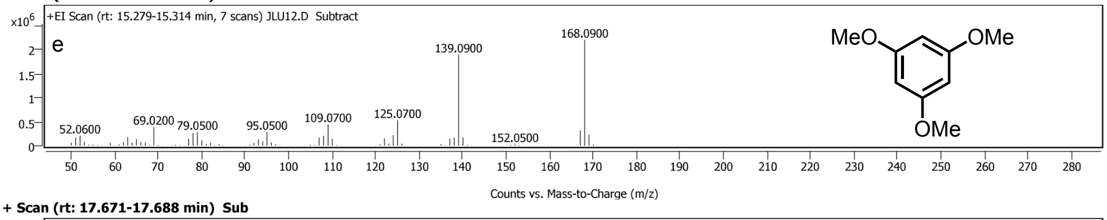

+ Scan (rt: 17.671-17.688 min) Sub

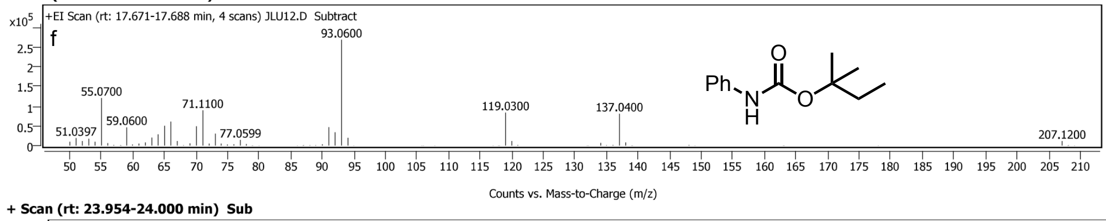

+ Scan (rt: 23.954-24.000 min) Sub

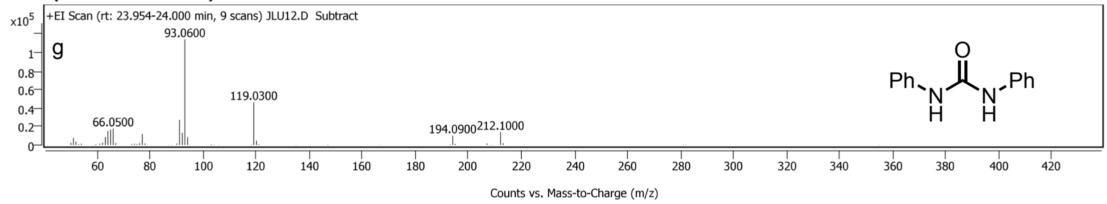

Figure 72: GC-MS data of entry 4 (Table S3).

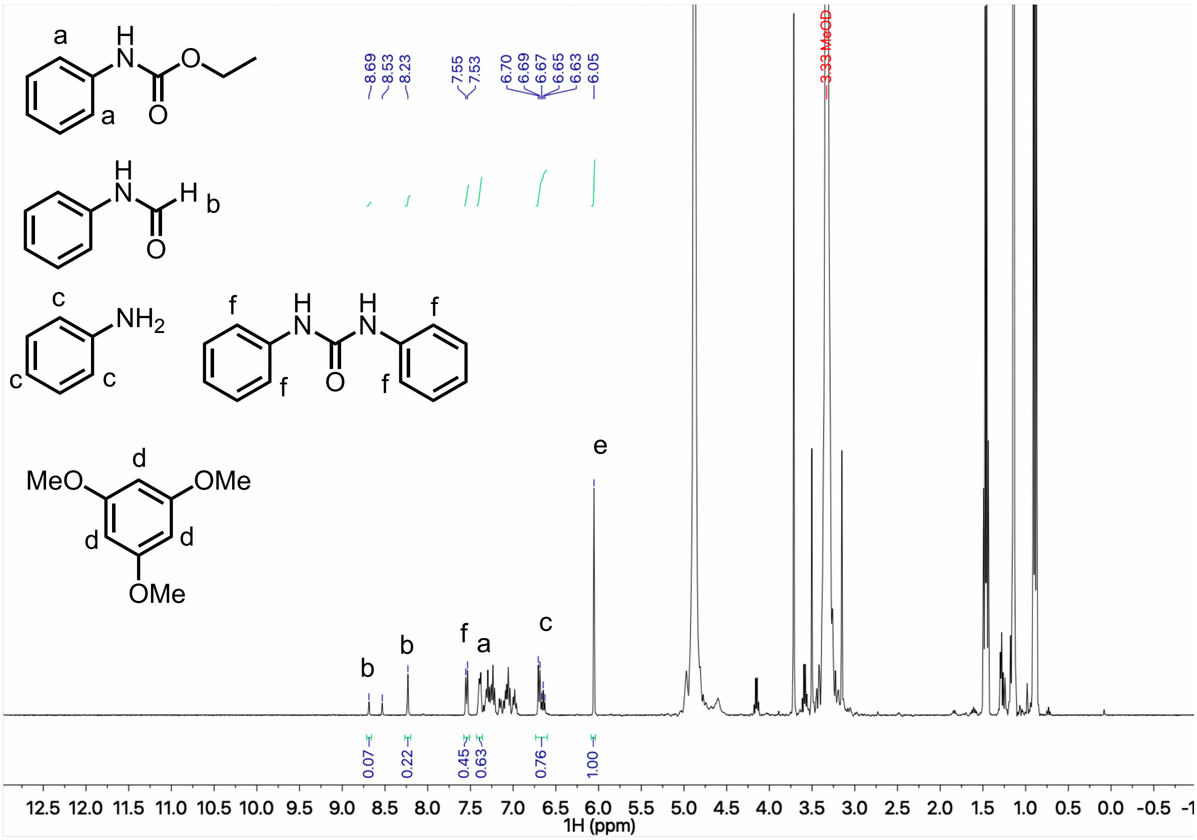

Figure 73: ^{1}H NMR (d^4-MeOD, 500 MHz) spectrum of entry 5 (Table S3). Trimethoxybenzene (0.33 mmol) was used as an internal standard. The NMR data of the formamide[1] and urea[5] shown here match well with the literature.

Sample Chromatograms

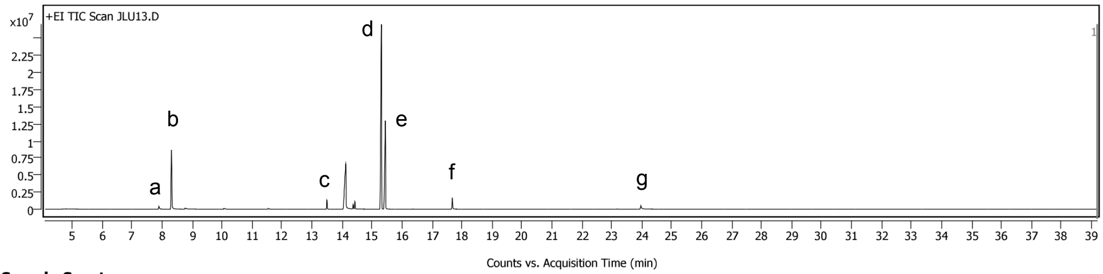

Sample Spectra

+ Scan (rt: 7.886-7.904 min)

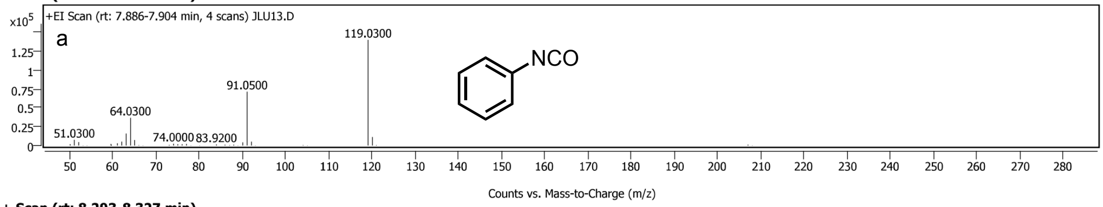

+ Scan (rt: 8.293-8.327 min)

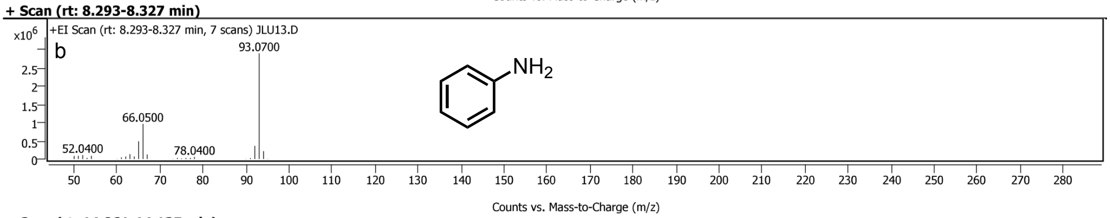

+ Scan (rt: 14.061-14.135 min)

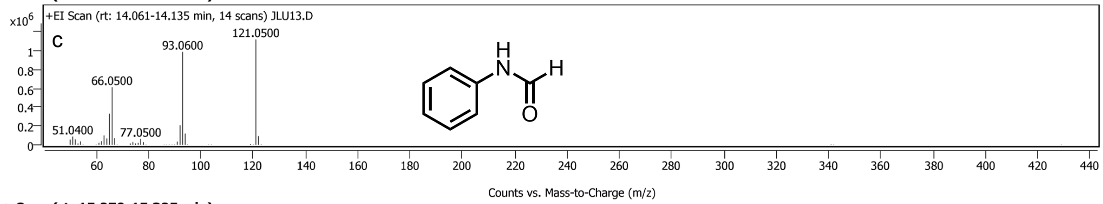

+ Scan (rt: 15.279-15.325 min)

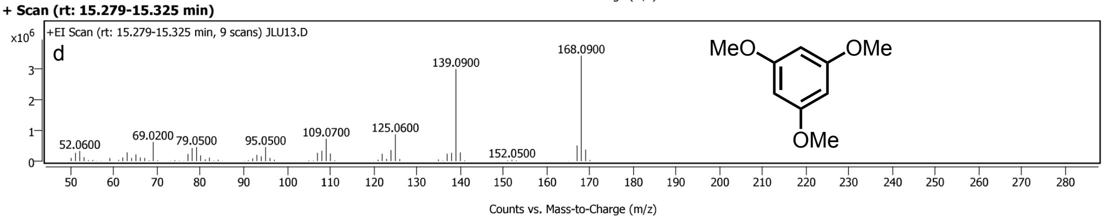

+ Scan (rt: 15.411-15.451 min)

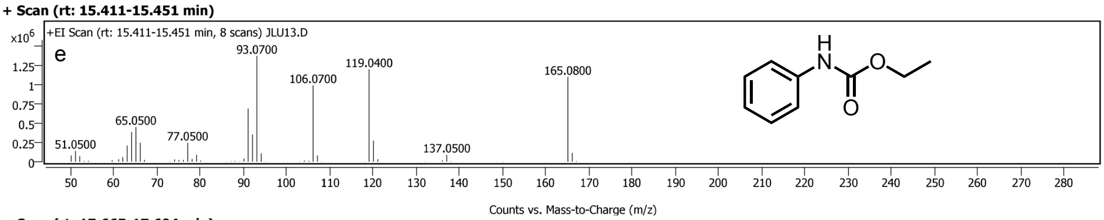

+ Scan (rt: 17.665-17.694 min)

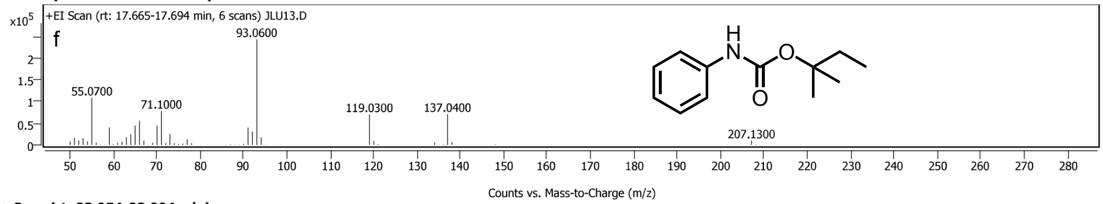

+ Scan (rt: 23.954-23.994 min)

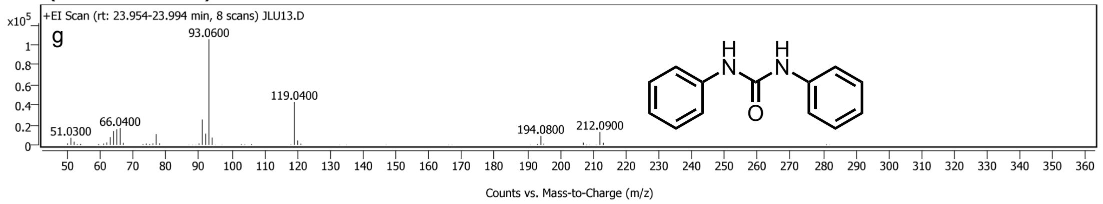

Figure 74: GC-MS data of entry 5 (Table S3).

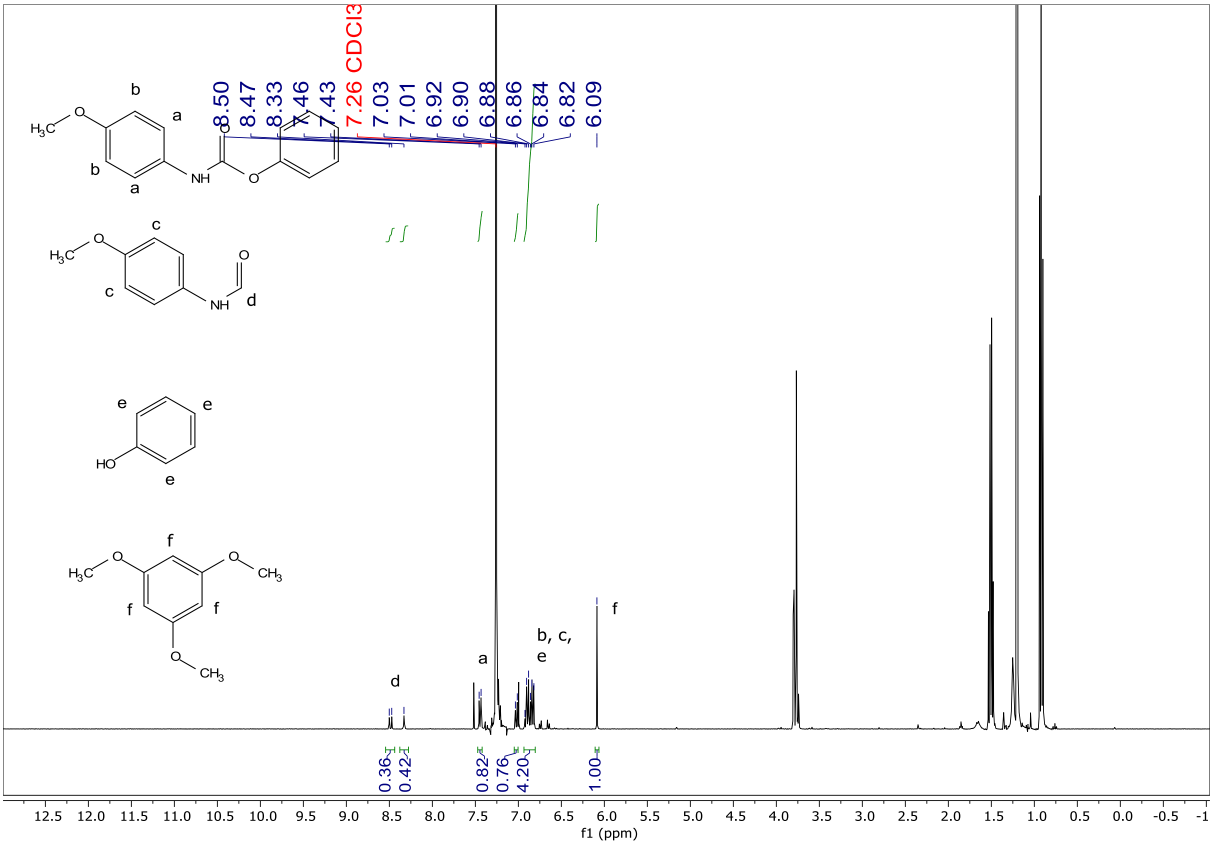

Figure 75: ^{1}H NMR ($CDCl_3$, 500 MHz) spectrum of entry 6 (Table S3). Trimethoxybenzene (0.33 mmol) was used as an internal standard. The NMR data of the formamide shown here match well with the literature.[6]

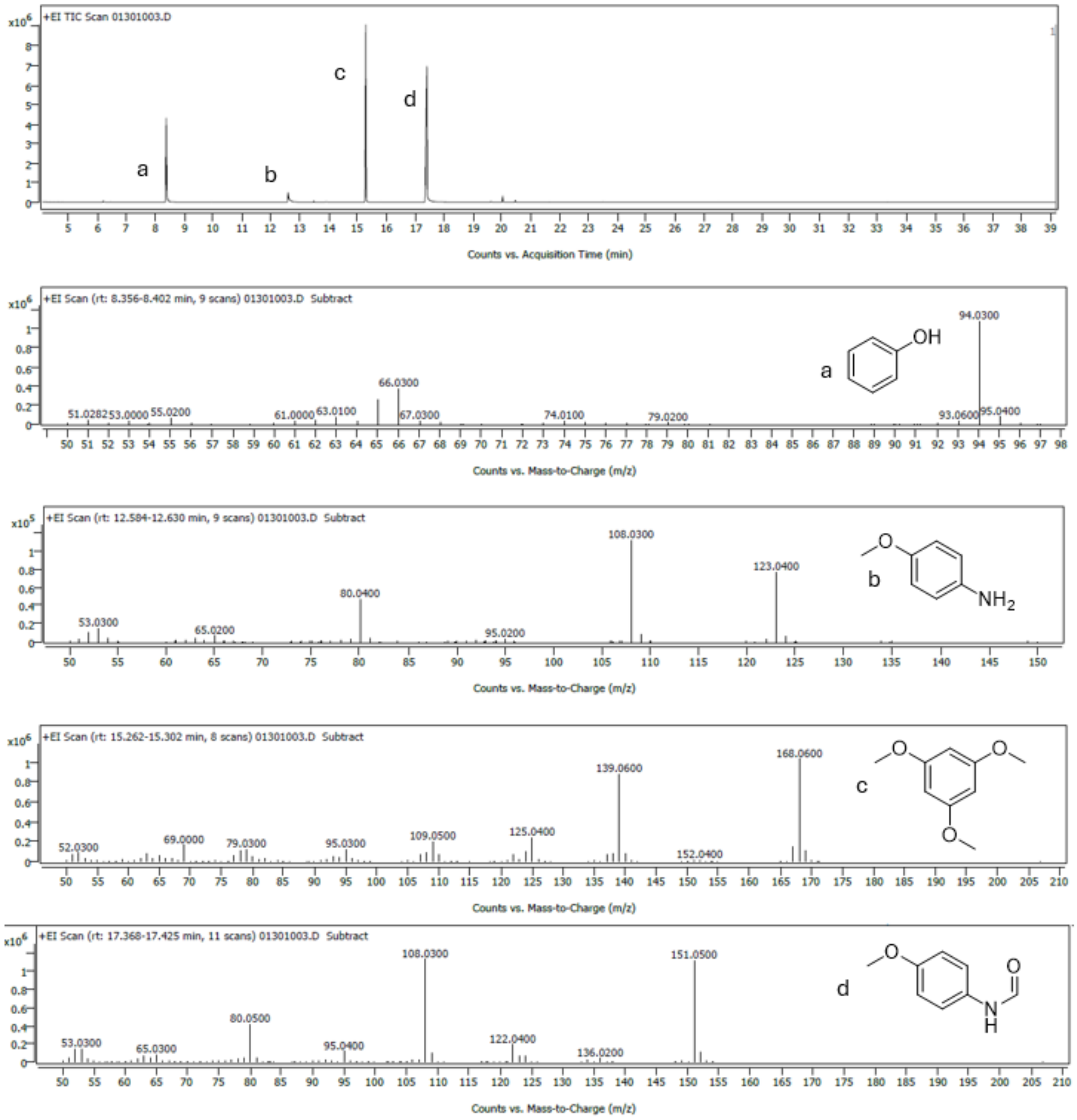

Figure 76: GC-MS data for entry 6 (Table S3).

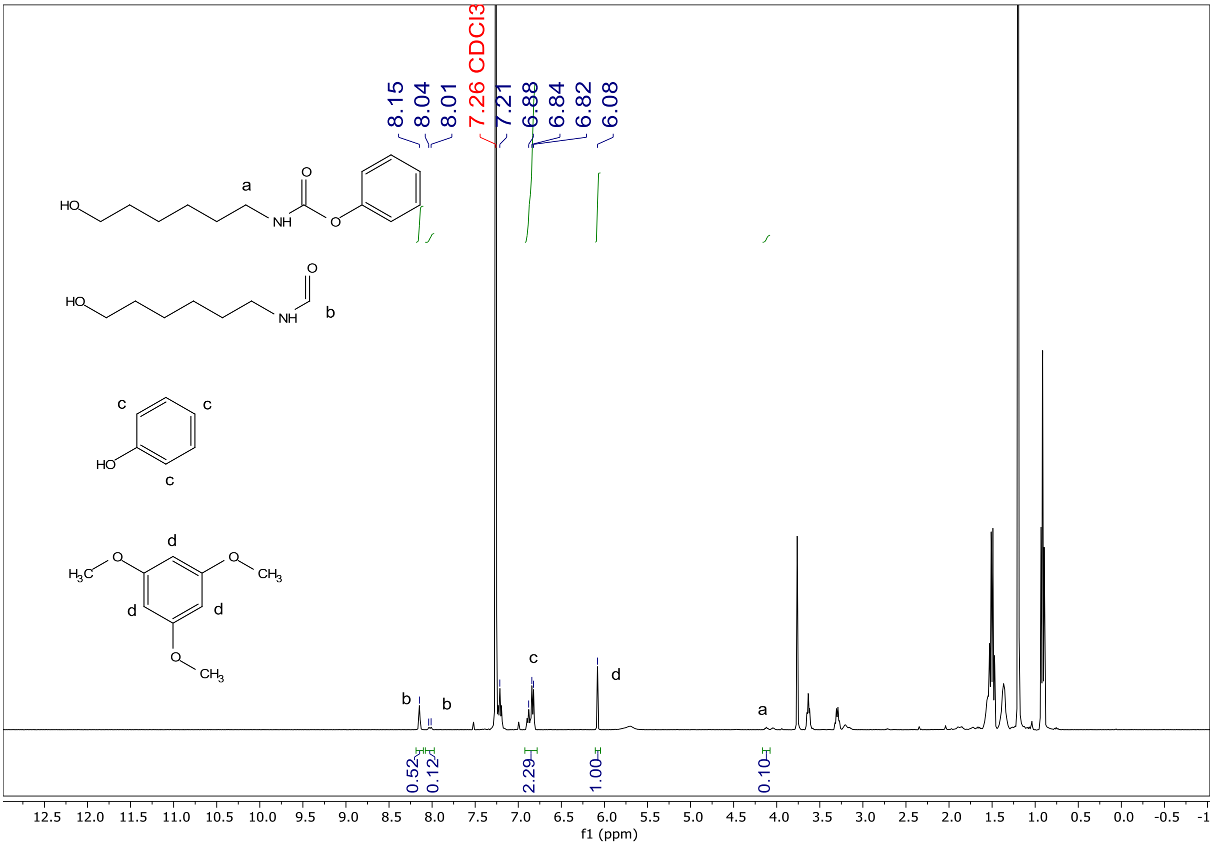

Figure 77: ${}^{1}H$ NMR ($CDCl_3$, 500 MHz) spectrum of entry 7 (Table S3). Trimethoxybenzene (0.33 mmol) was used as an internal standard. The NMR data of the formamide[7] shown here match well with the literature.

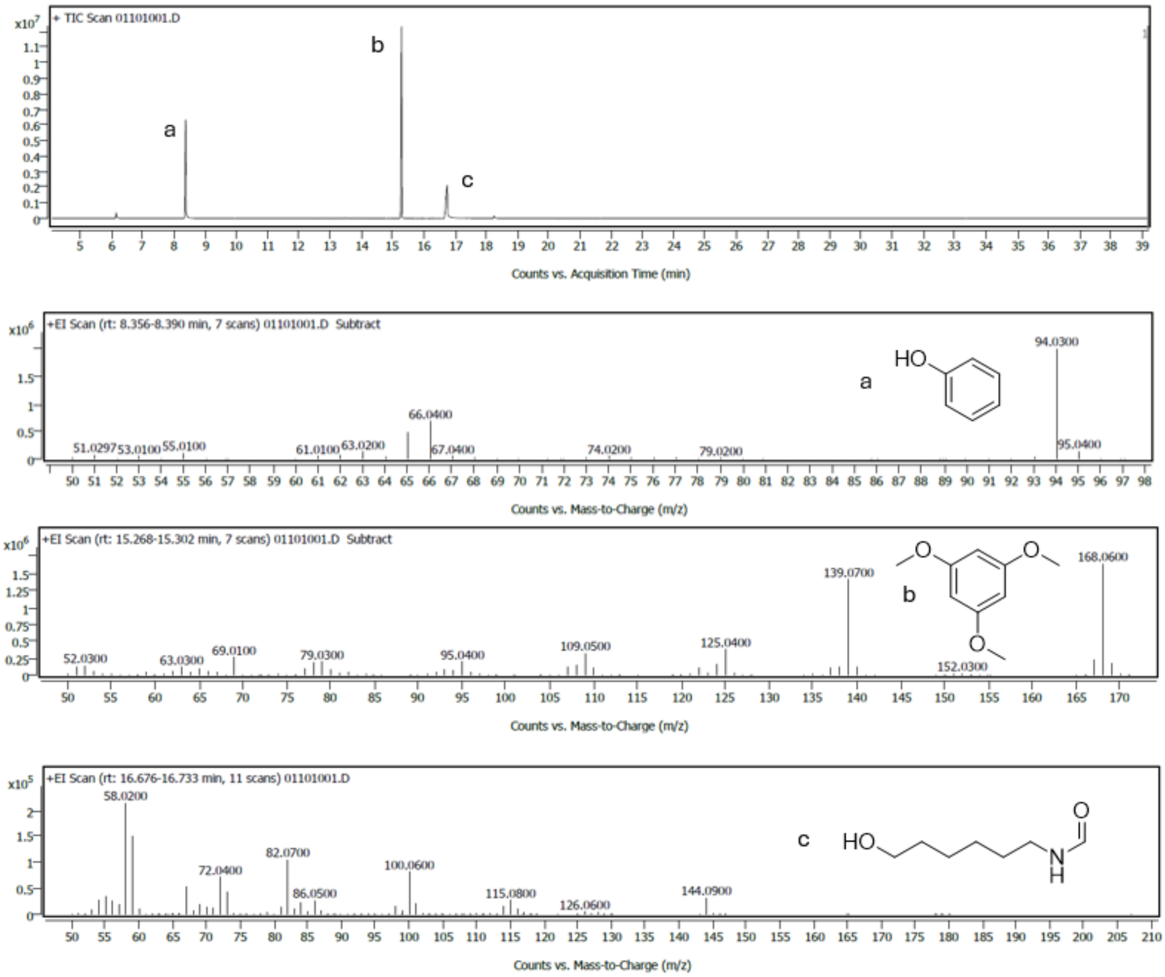

Figure 78: GC-MS data of entry 7 (Table S3).

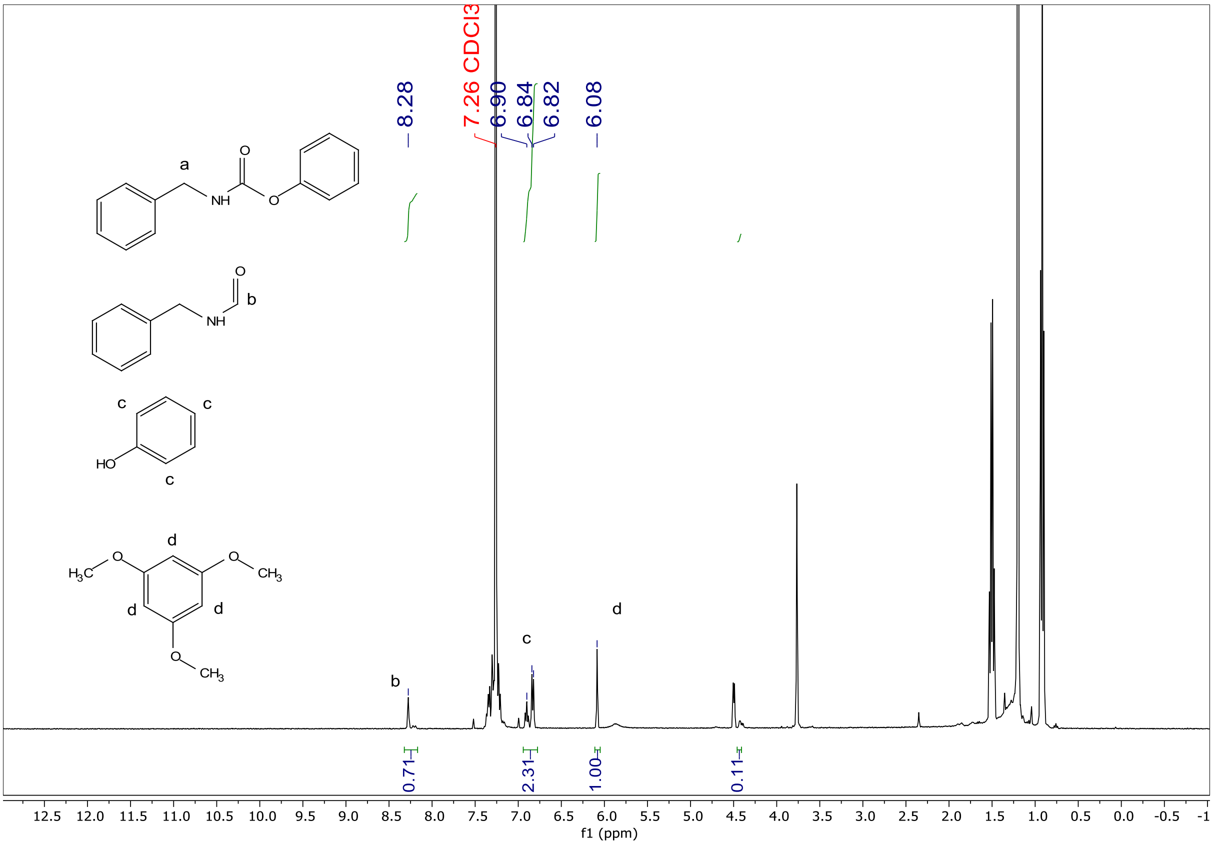

Figure 79: ^{1}H NMR ($CDCl_3$, 500 MHz) spectrum of entry 8 (Table S3). Trimethoxybenzene (0.33 mmol) was used as an internal standard. The NMR data of the formamide shown here match well with the literature.[8]

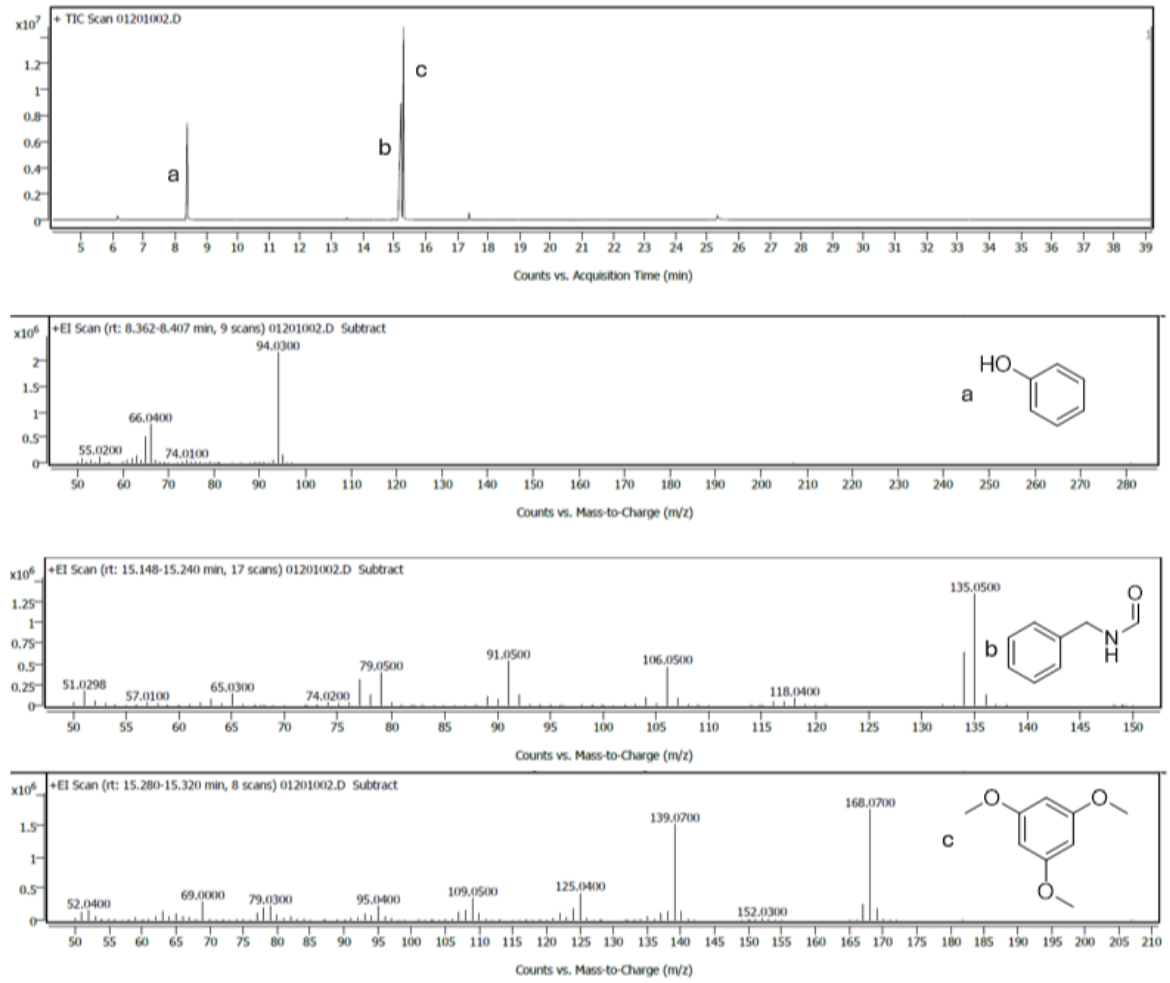

Figure 80: GC-MS data of entry 8 (Table S3).

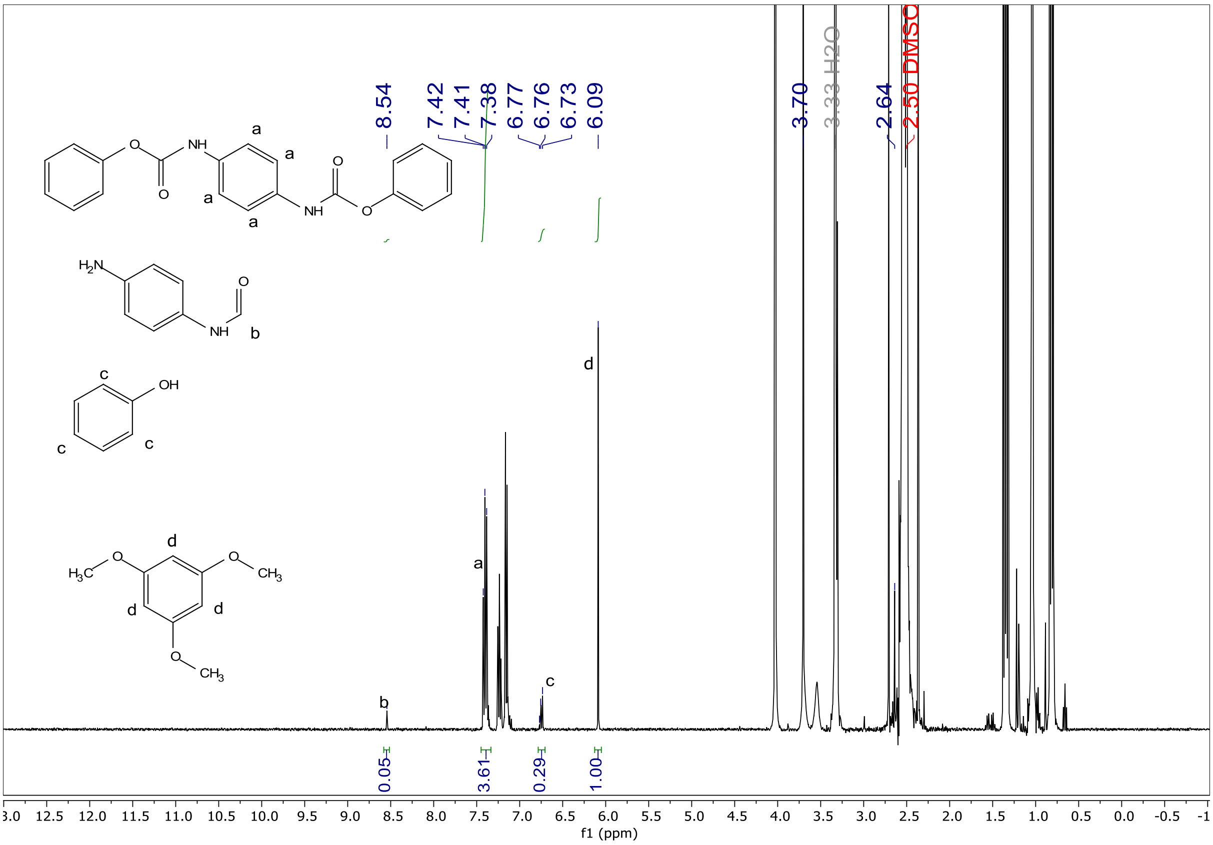

Figure 81: ¹H NMR (CDCl₃, 500 MHz) spectrum of entry 9 (Table S3). Trimethoxybenzene (0.33 mmol) was used as an internal standard. . The NMR data of the formamide shown here match well with the literature.[9]

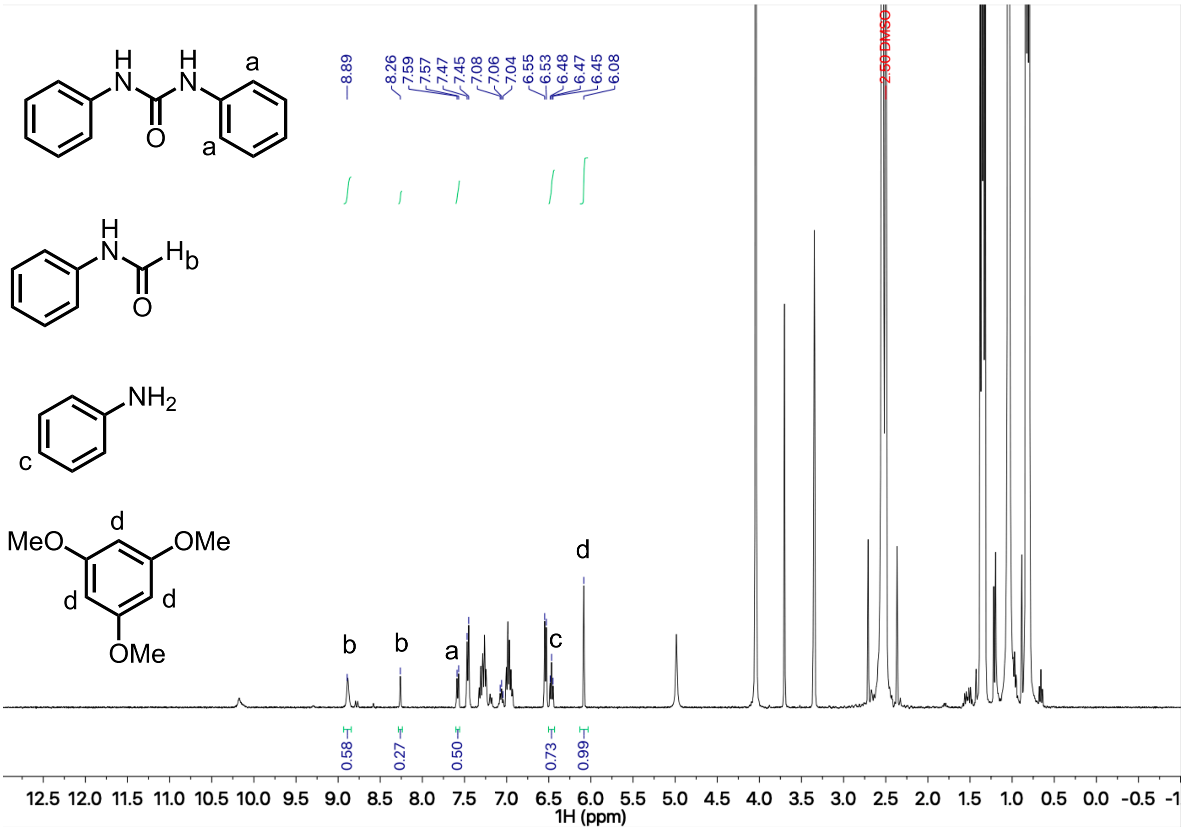

Figure 82: ^{1}H NMR (d_5-DMSO, 500 MHz) spectrum of entry 10 (Table S3). Trimethoxybenzene (0.33 mmol) was used as an internal standard. . The NMR data of the formamide shown here match well with the literature.[1]

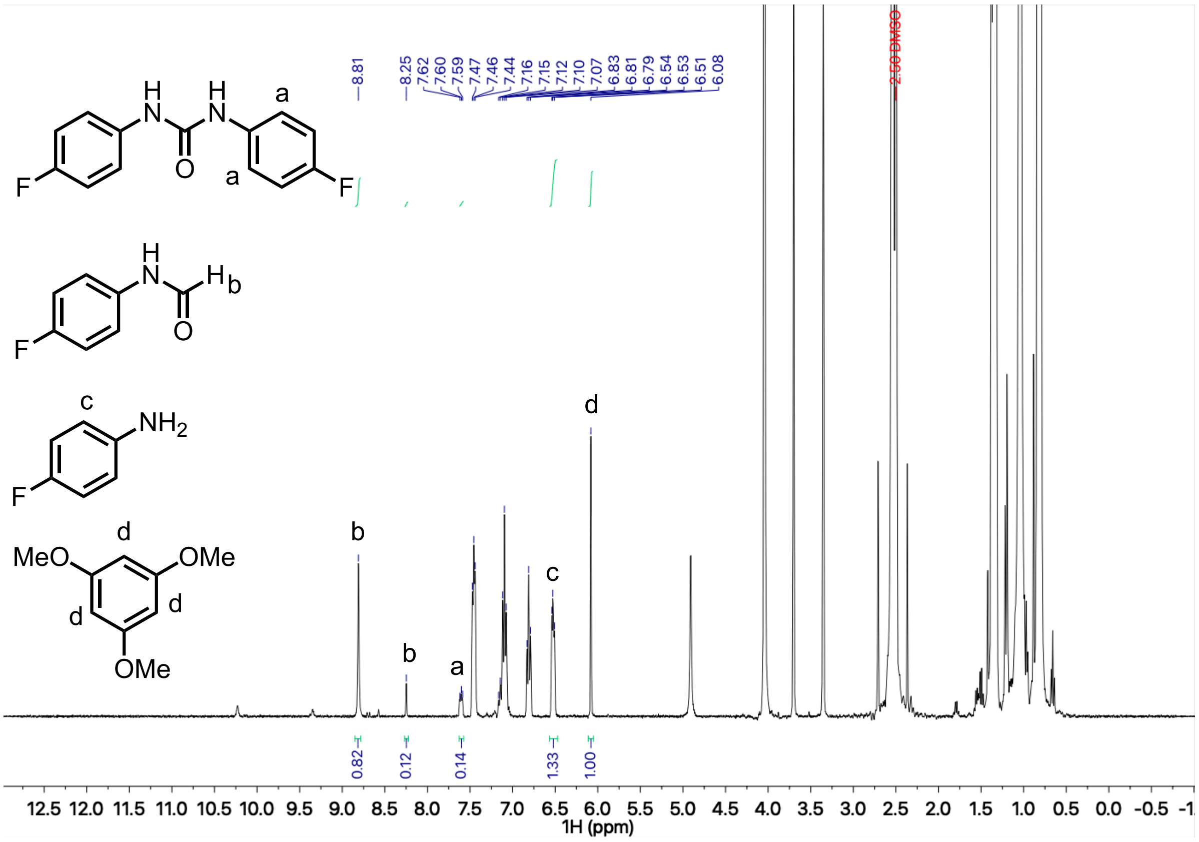

Figure 83: ^{1}H NMR (d_5-DMSO, 500 MHz) spectrum of entry 11 (Table S3). Trimethoxybenzene (0.33 mmol) was used as an internal standard. The NMR data of the formamide shown here match well with the literature.[10]

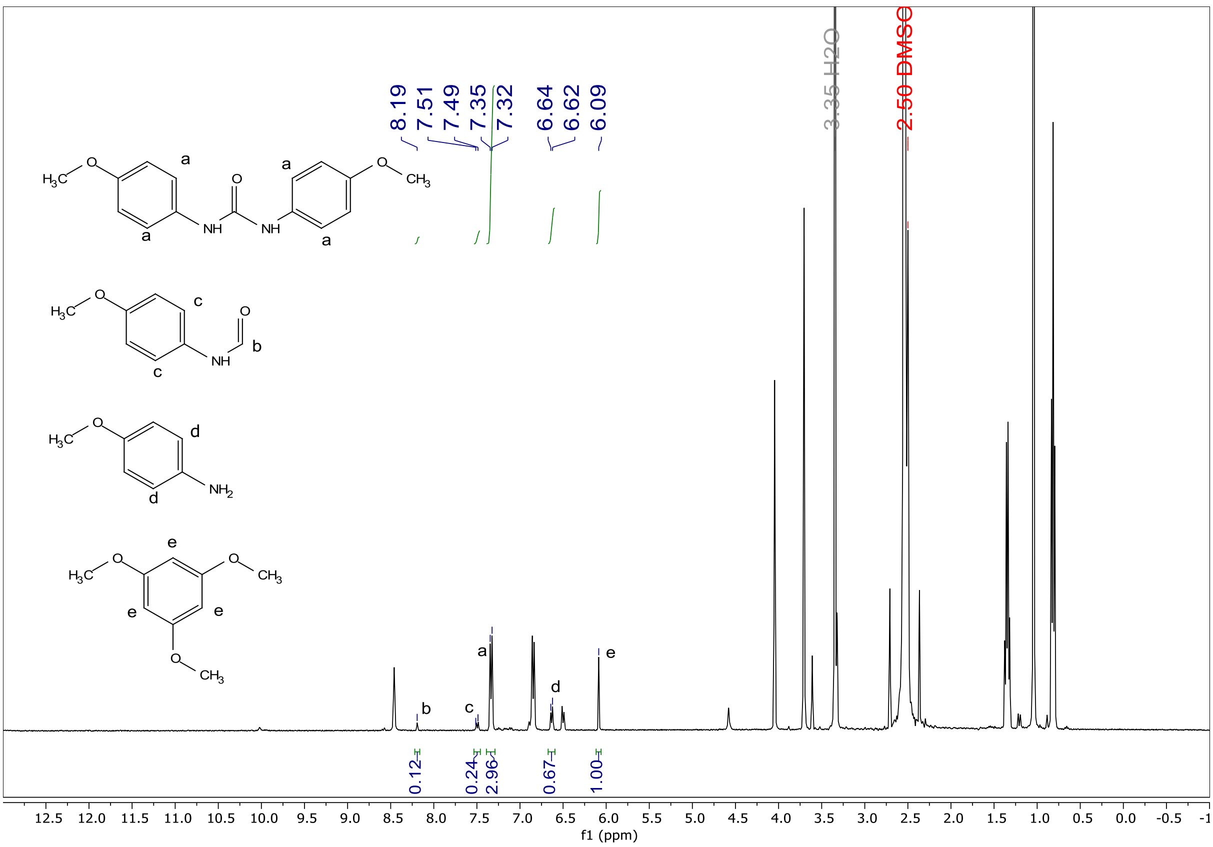

Figure 84: ^{1}H NMR (DMSO, 500 MHz) spectrum of entry 12 (Table S3). Trimethoxybenzene (0.33 mmol) was used as an internal standard. Benzylamine yield could not be determined by NMR. The NMR data of the formamide shown here match well with the literature.[6]

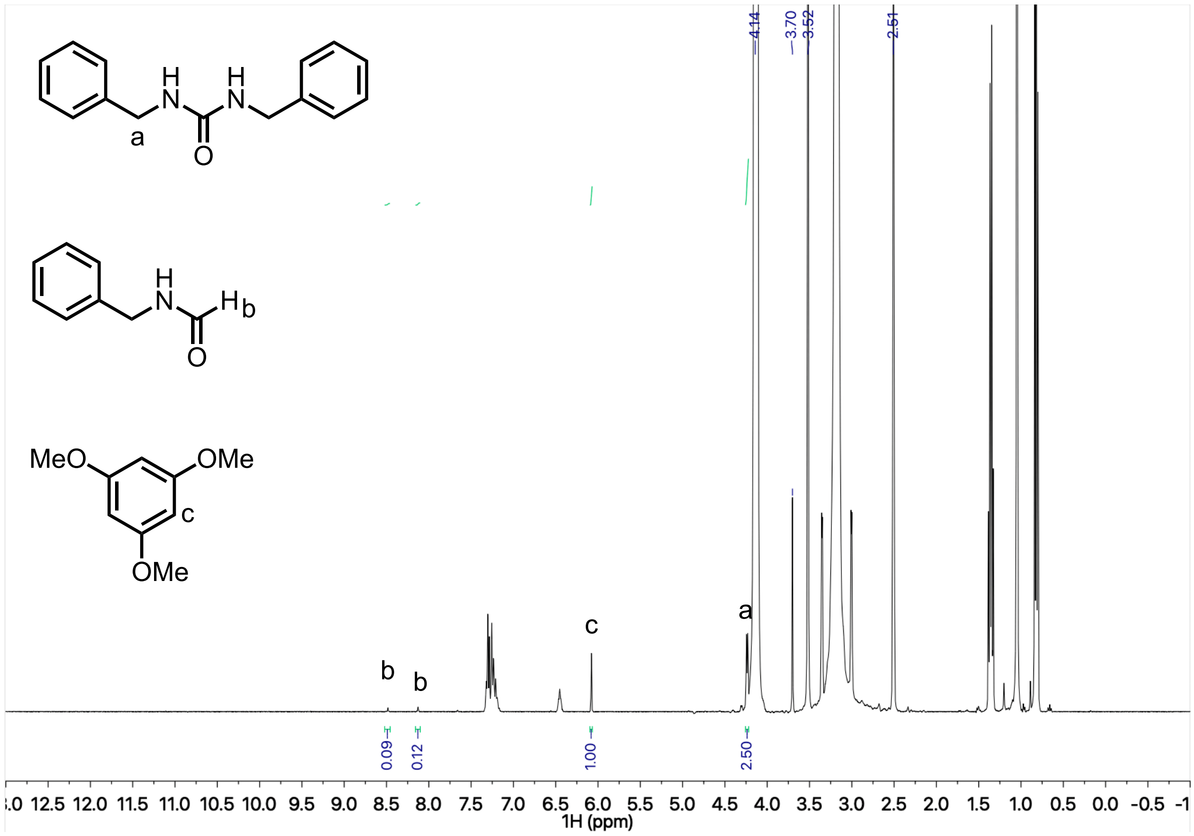

Figure 85: ^{1}H NMR (DMSO, 500 MHz) spectrum of entry 13 (Table S3). Trimethoxybenzene (0.33 mmol) was used as an internal standard. The NMR data of the formamide shown here match well with the literature.[8]

5. Hydrogenative depolymerization of polyurethanes

5.1 Procedure for the hydrogenation of polyurethanes

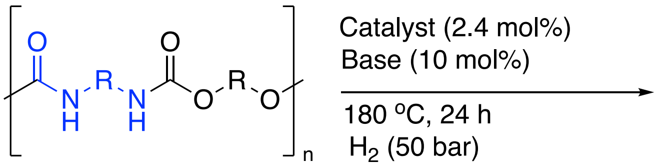

Figure 86: General Scheme for the hydrogenation of polyurethanes

The polyurethane (7.6 mmol, mass calculated based on molecular weight of repeated unit), 5 wt% Pd/Al_2O_3 (2.4 mol%), and base (10 mol%) were weighed under air and transferred to the autoclave body, object D. The autoclave head was securely screwed on, object E, and the autoclave was flushed with nitrogen five times. The solvent (20 mL) was transferred through a syringe. The autoclave was degassed three times using H_2 gas and afterwards pressurized with 50 bar of H_2 gas. The heating jacket, object F, was placed onto the autoclave and heated to 180 °C for 24 h. After completion of the reaction time, the autoclave was left to cool by removing the heating jacket, and the hydrogen gas was slowly vented off. After the solid catalyst was filtered off and the products dissolved in $CHCl_3$, the products were isolated using column chromatography using a hexane: ethyl acetate 1:1 system and subsequently dried.

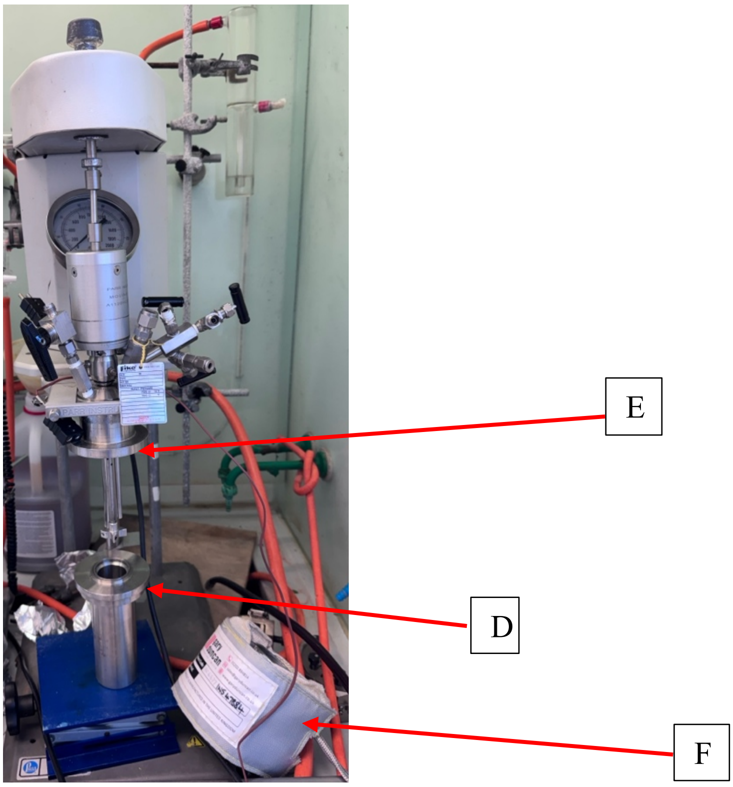

Figure 60: Image of the autoclave with a mechanical stirrer used for the hydrogenation of polyurethanes.

The characterization of the depolymerization products match well with those reported in the literature (Schaub et.al., Hydrogenative Depolymerization of Polyurethanes Catalyzed by a Manganese Pincer Complex. *ChemSusChem* **2022**, *15* (1), e202101606).

5.2 Analytical Data for the starting polyurethanes used in the hydrogenation reactions

Polyether TPU (PU1)

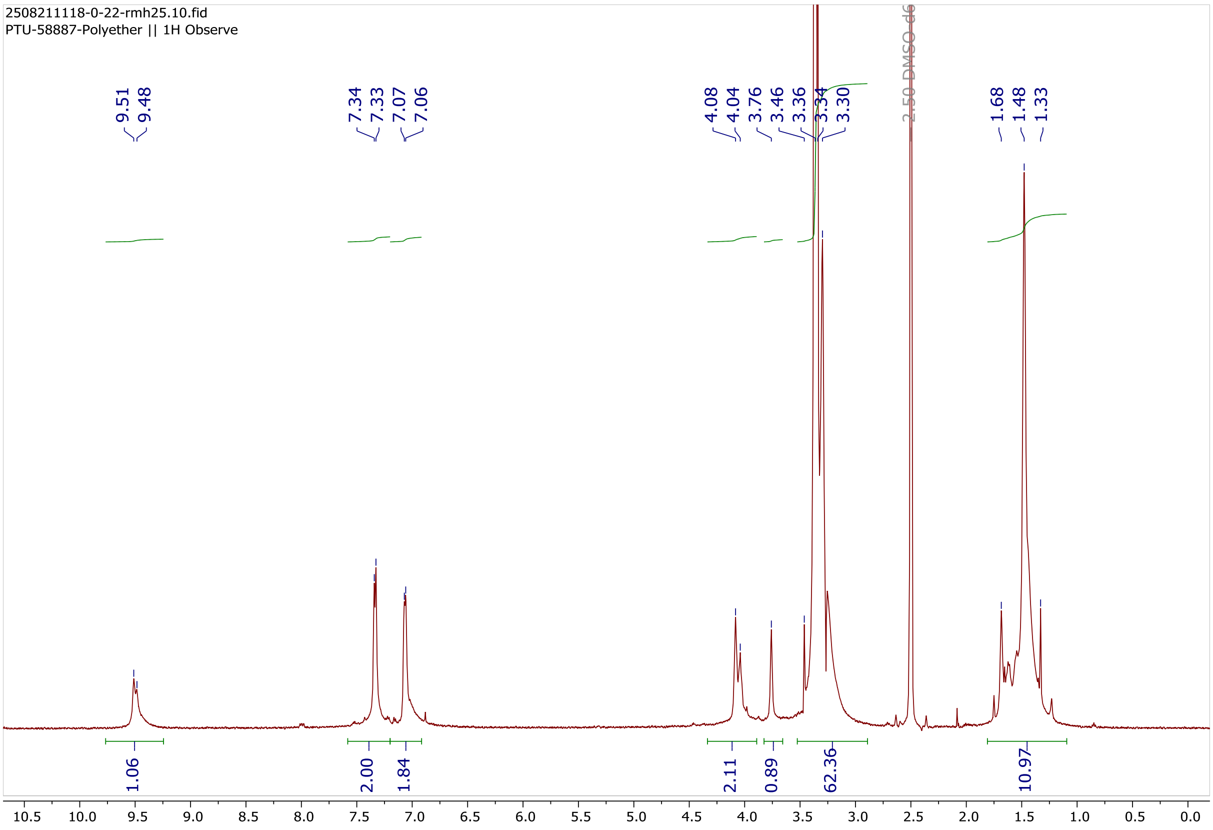

Figure 87: ^{1}H NMR (500 MHz, $(CD_3)_2CO$) spectrum of polyether TPU (PU1).

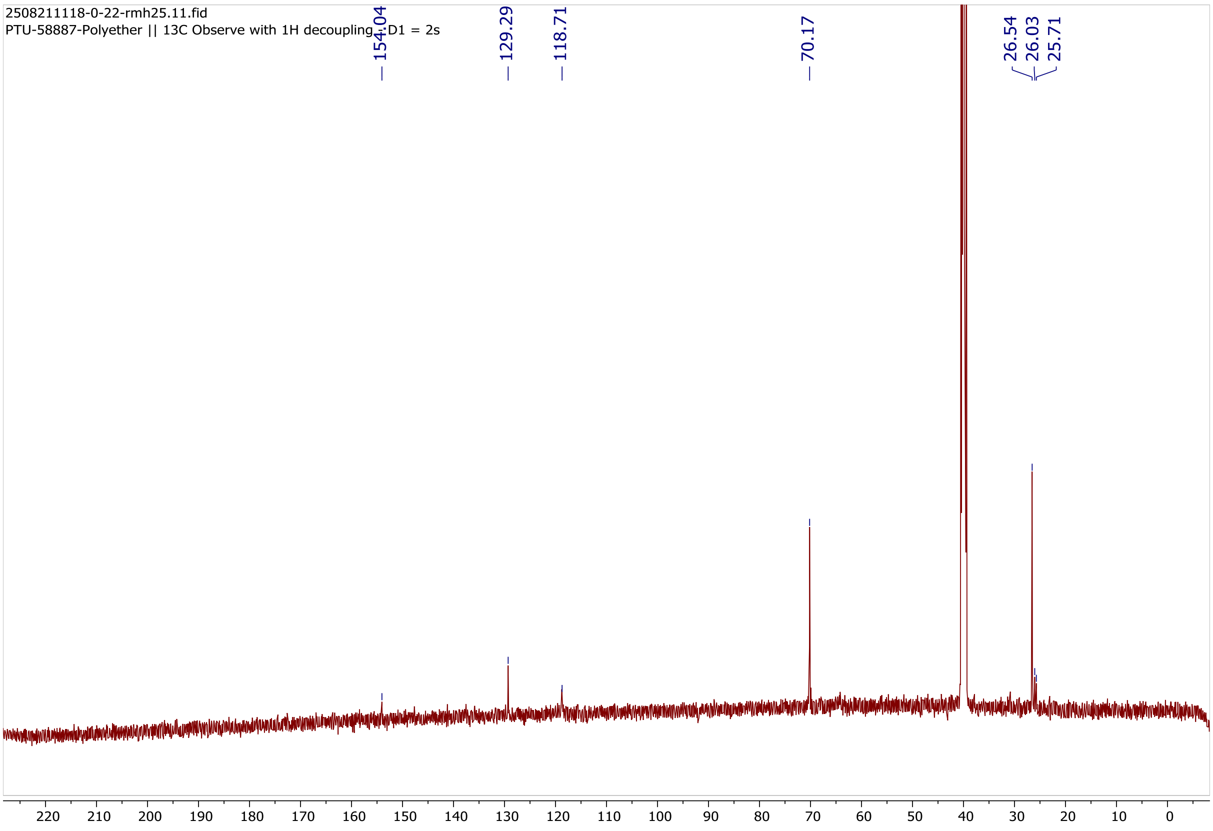

Figure 88: $^{13}C\{^{1}H\}$ NMR (500 MHz, $(CD_3)_2CO$) spectrum of polyether TPU (PU1).

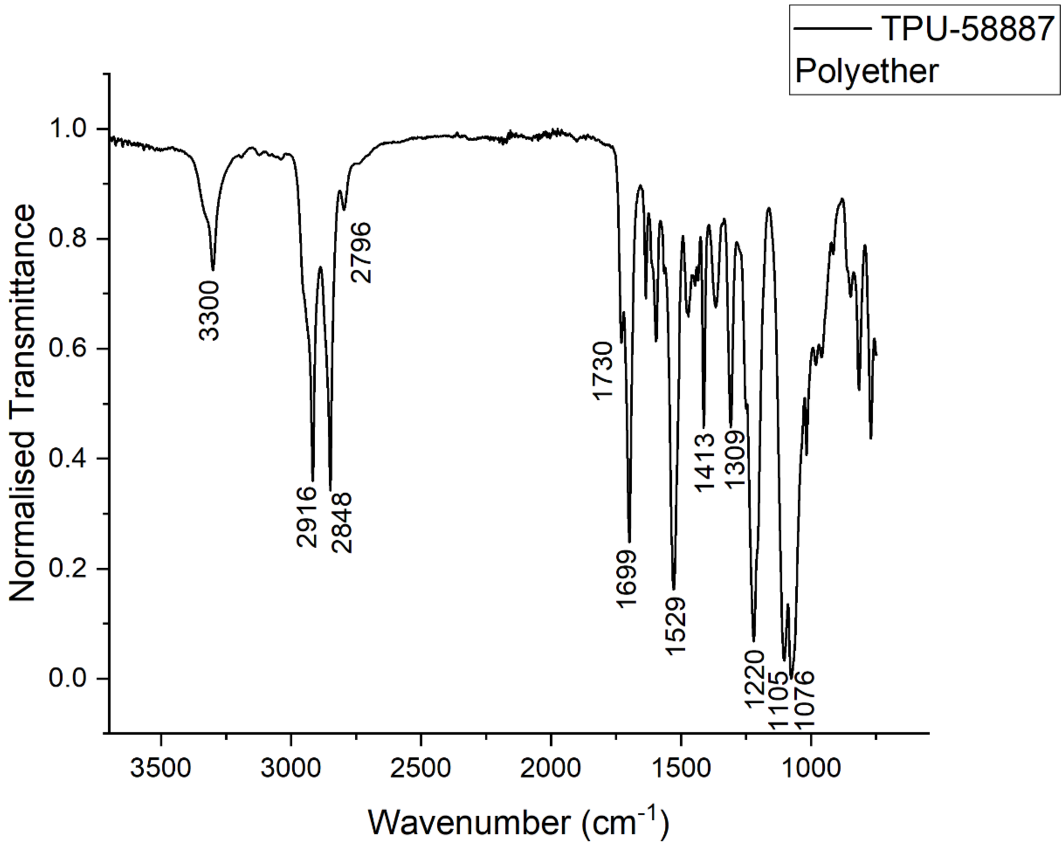

Figure 89: Infrared spectrum from the polyether TPU (PU1).

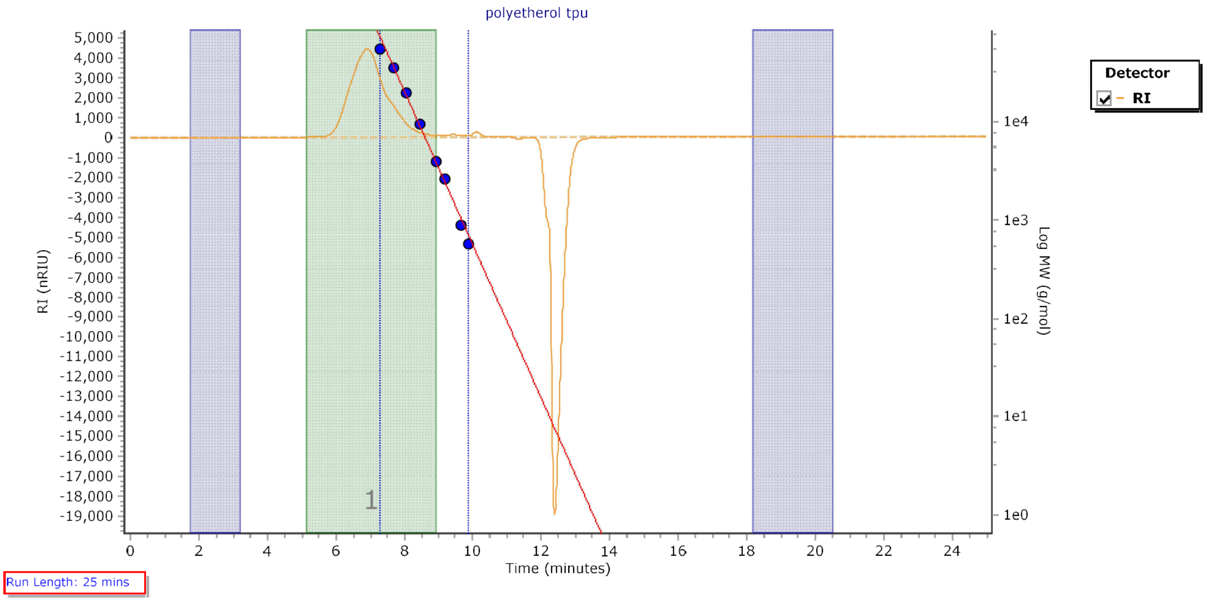

Figure 90: GPC trace of TPU containing polyetherol (PU1) in DMF as solvent system.

Molecular Weight Averages

Peak	Mp (g/mol)	Mn (g/mol)	Mw (g/mol)	Mz (g/mol)	Mz+1(g/mol)	Mv (g/mol)	PD
Peak 1	138740	66337	180657	379731	823744	161194	2.723

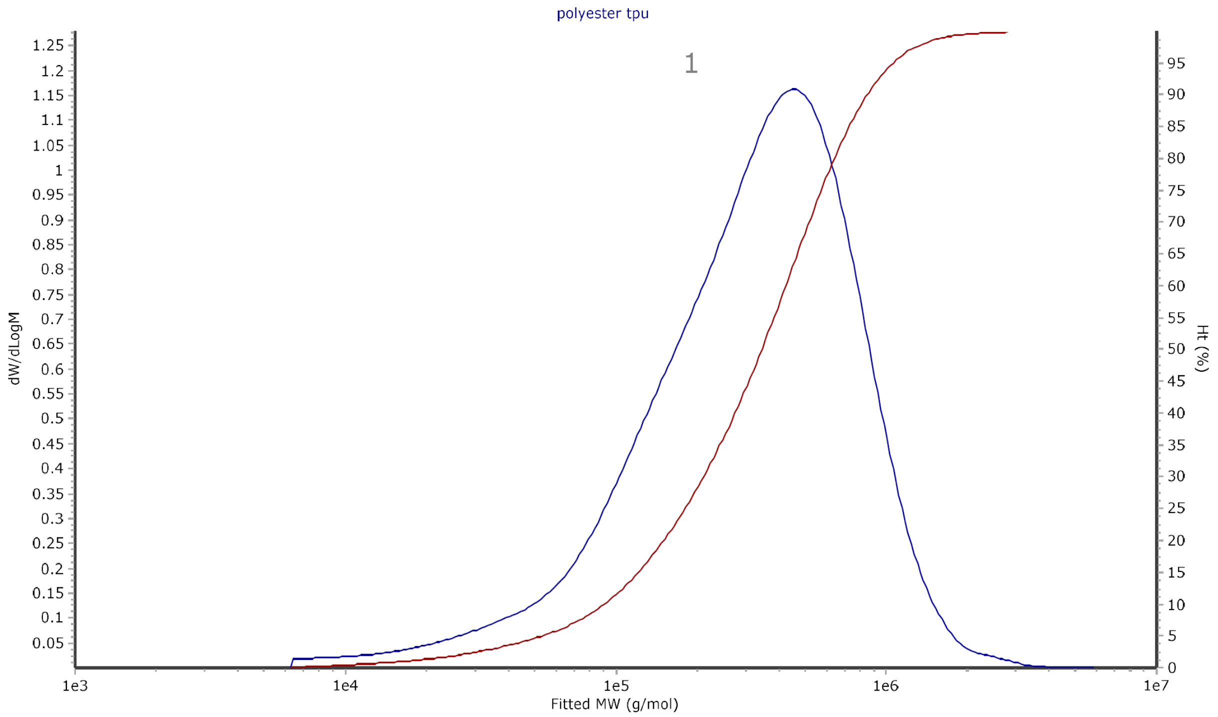

Figure 91: GPC data analysis and GPC trace for TPU containing polyetherol (PU1) in DMF as solvent system.

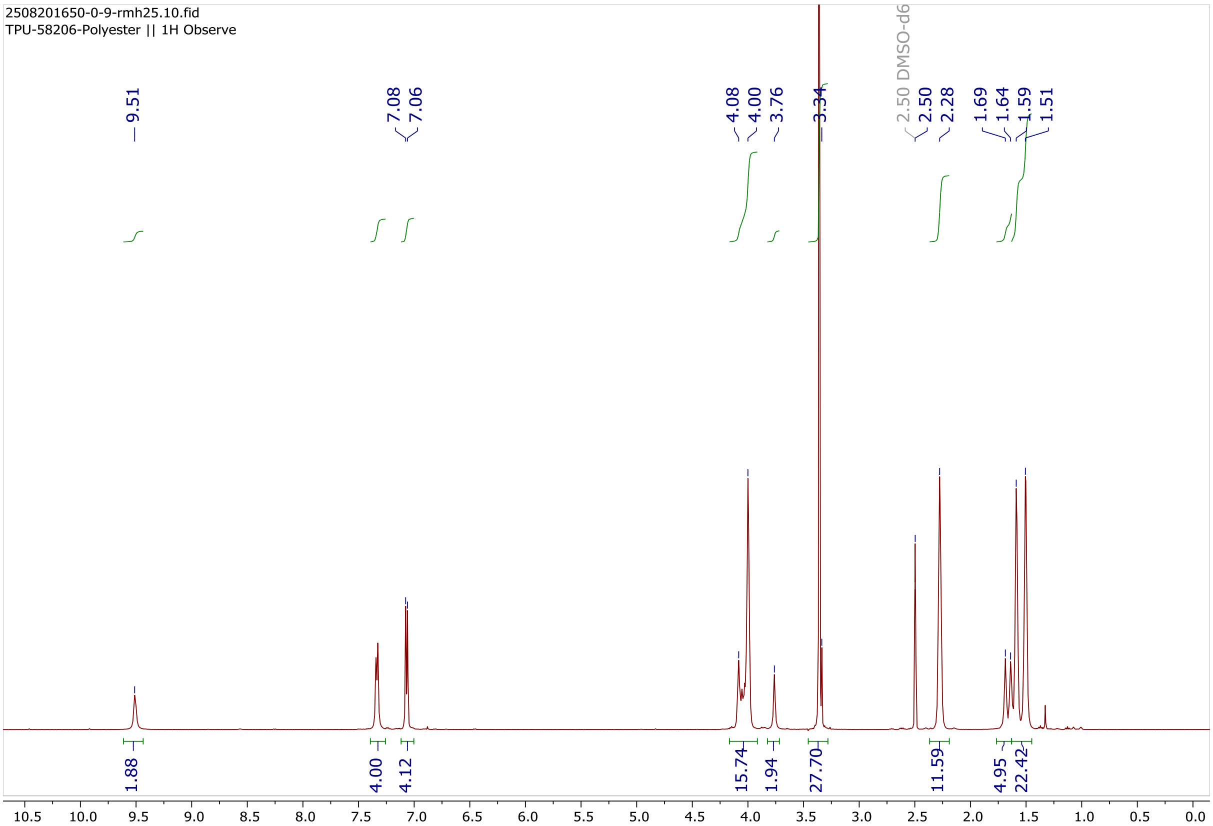

Figure 92: ^{1}H NMR (500 MHz, $(CD_3)_2CO$) spectrum of polyester TPU (PU2).

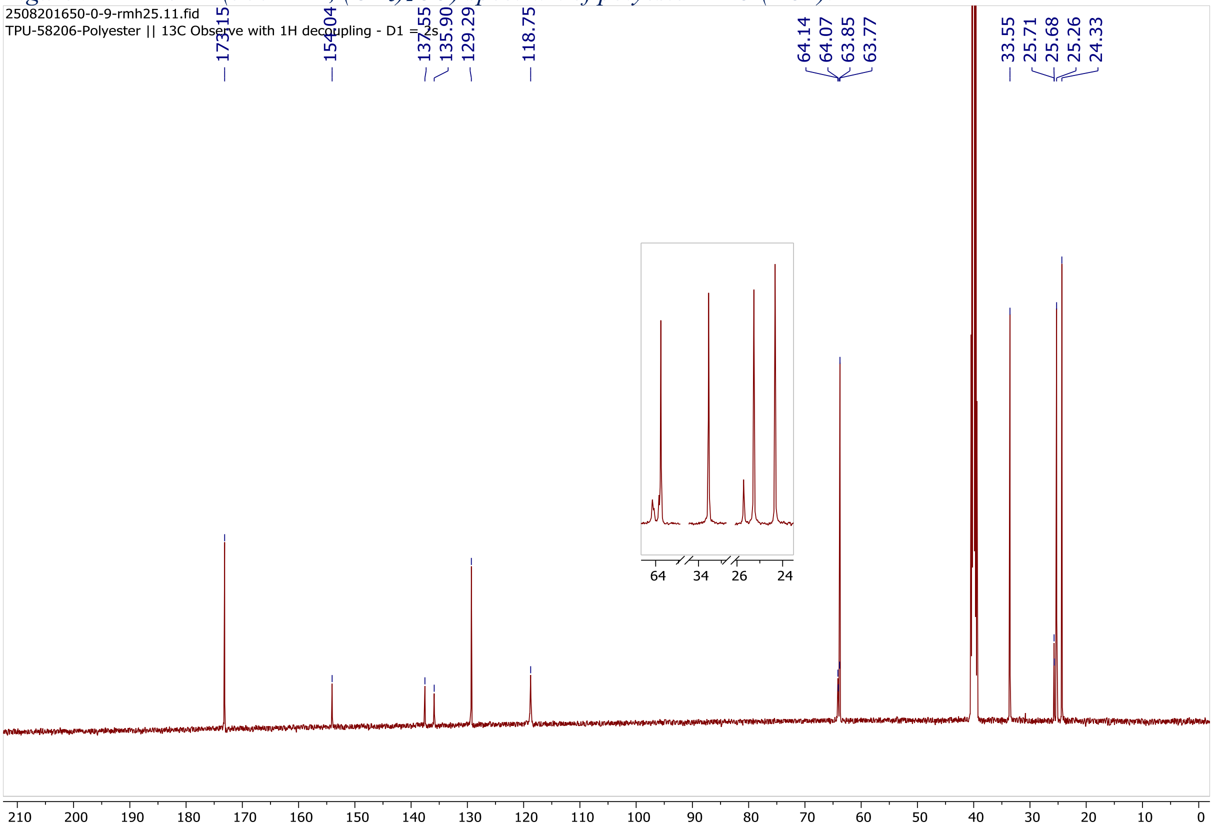

Figure 93: ^{13}C{^{1}H} NMR (500 MHz, $(CD_3)_2CO$) spectrum of polyester TPU (PU2).

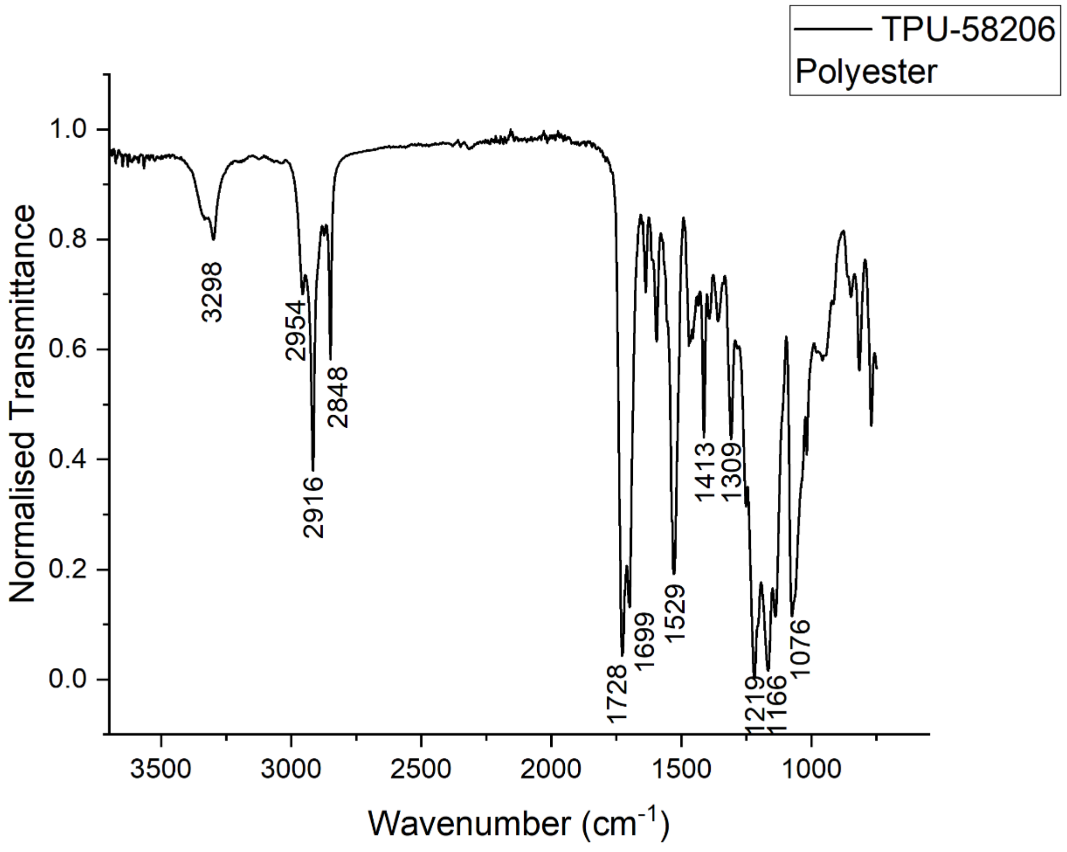

Figure 94: Infrared spectrum from the polyester TPU (PU2).

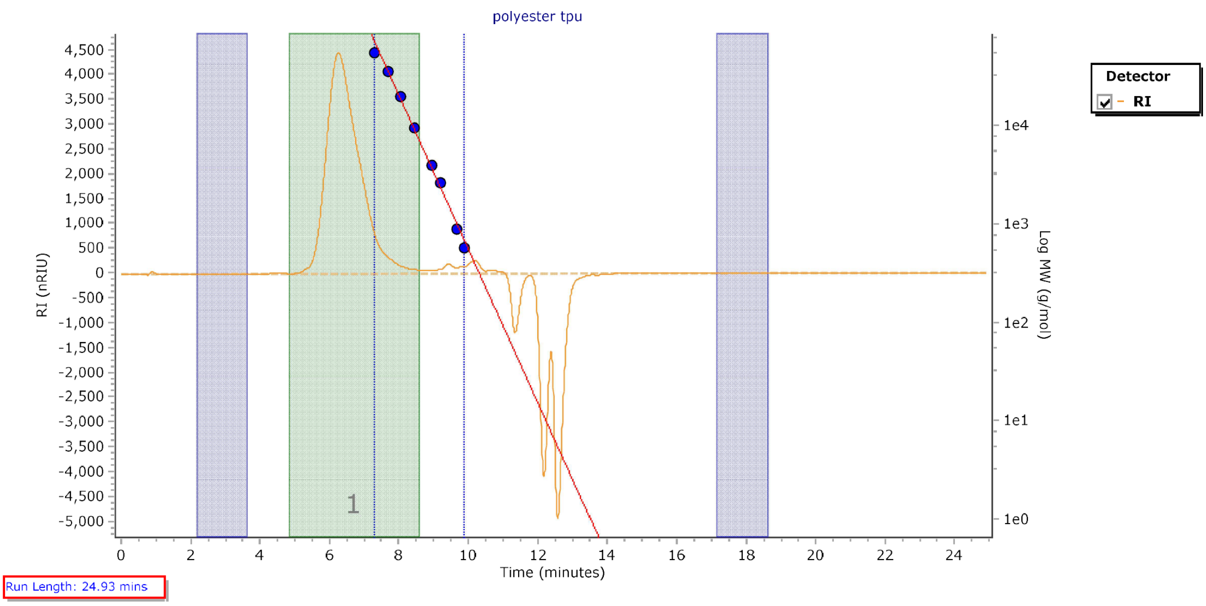

Figure 95: Gel-permeation chromatography (GPC) trace of the polyester TPU (PU2).

Molecular Weight Averages

Peak	Mp (g/mol)	Mn (g/mol)	Mw (g/mol)	Mz (g/mol)	Mz+1(g/mol)	Mv (g/mol)	PD
Peak 1	451036	169204	428901	713161	1072894	393063	2.535

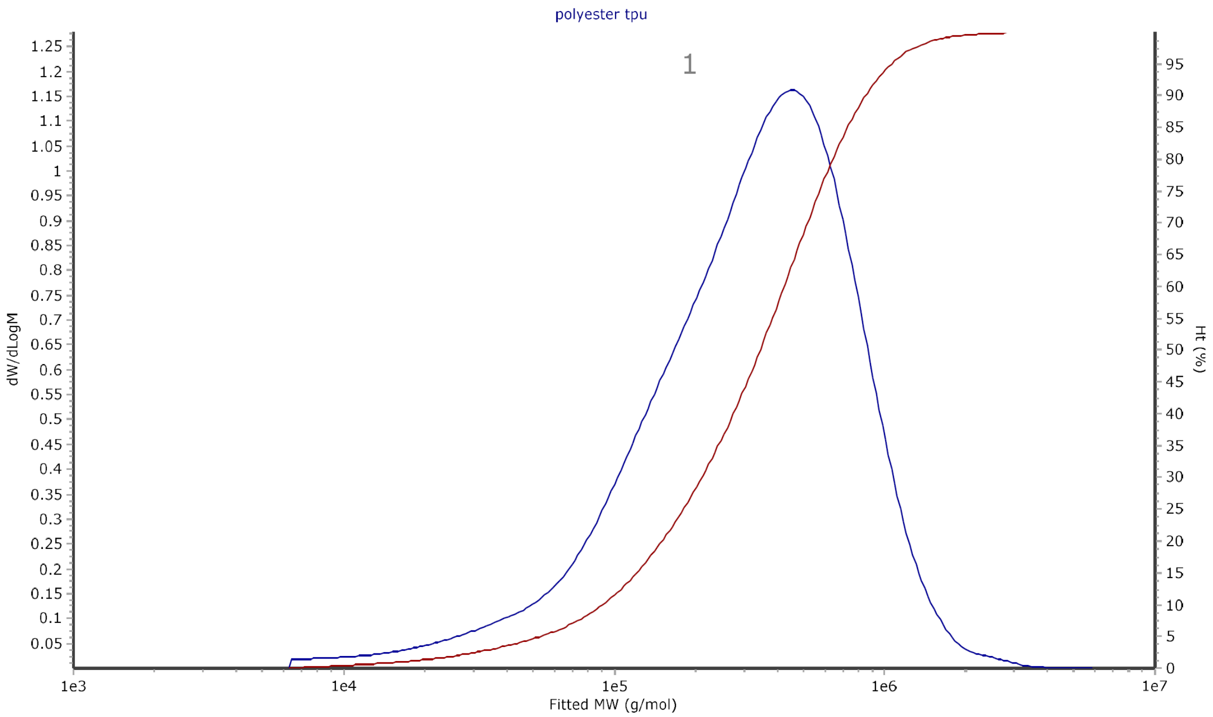

Figure 96: GPC data analysis and analyticalal gel-permeation chromatography (GPC) trace for polyester TPU (PU2) using DMF as solvent system

5.3 Analytical data for the hydrogenation of polyurethanes

Products obtained from the hydrogenative depolymerization of Polyether TPU (PU1)

The color of isolated aromatic diamine, formamide and polyetherpolyol was white in all cases.

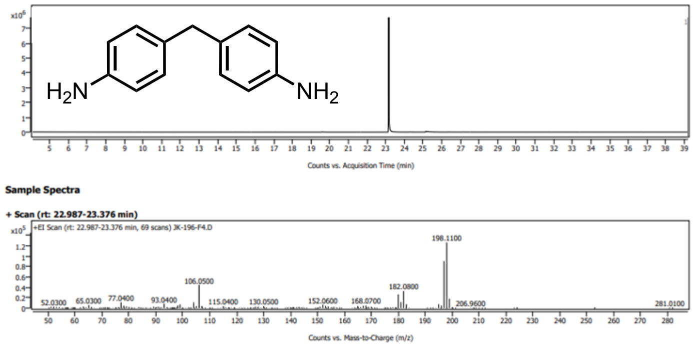

Figure 97: GC-MS data of the isolated diamine from the hydrogenative depolymerization of TPU containing polyetherol (Figure 3A, main manuscript).

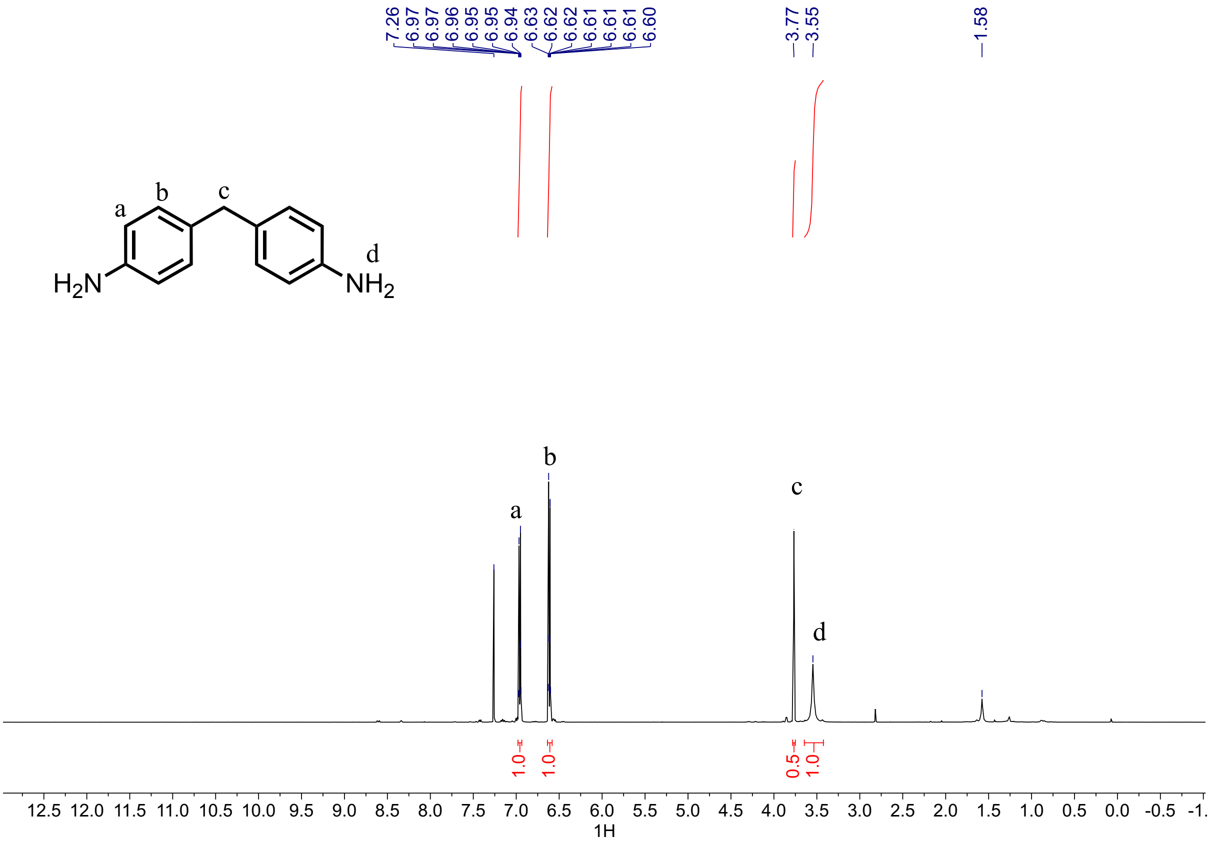

Figure 98: ^{1}H NMR (500 MHz, $CDCl_3$) spectrum of diamine isolated from the hydrogenative depolymerization of TPU containing polyetherol (Figure 3A, main manuscript).

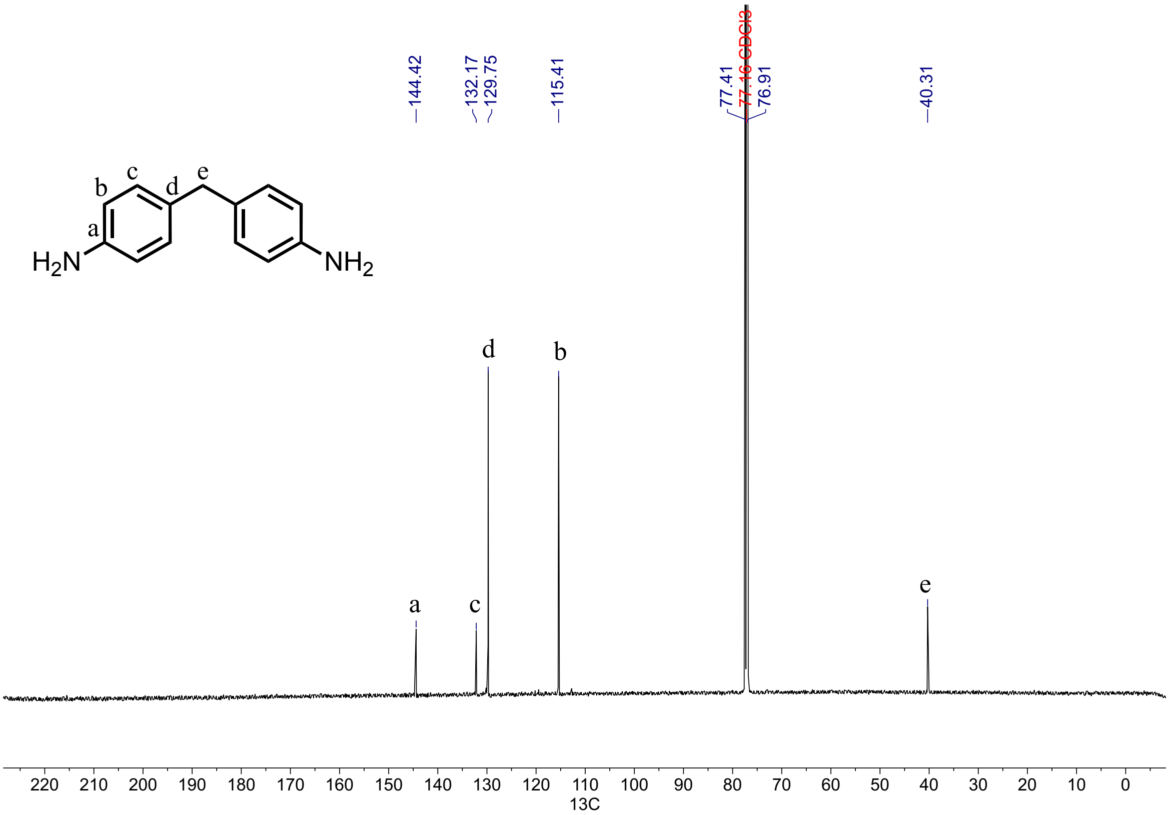

Figure 99: $^{13}C\{^1H\}$ NMR (125 MHz, $CDCl_3$) spectrum of diamine isolated from the hydrogenative depolymerization of TPU containing polyetherol (Figure 3A, main manuscript).

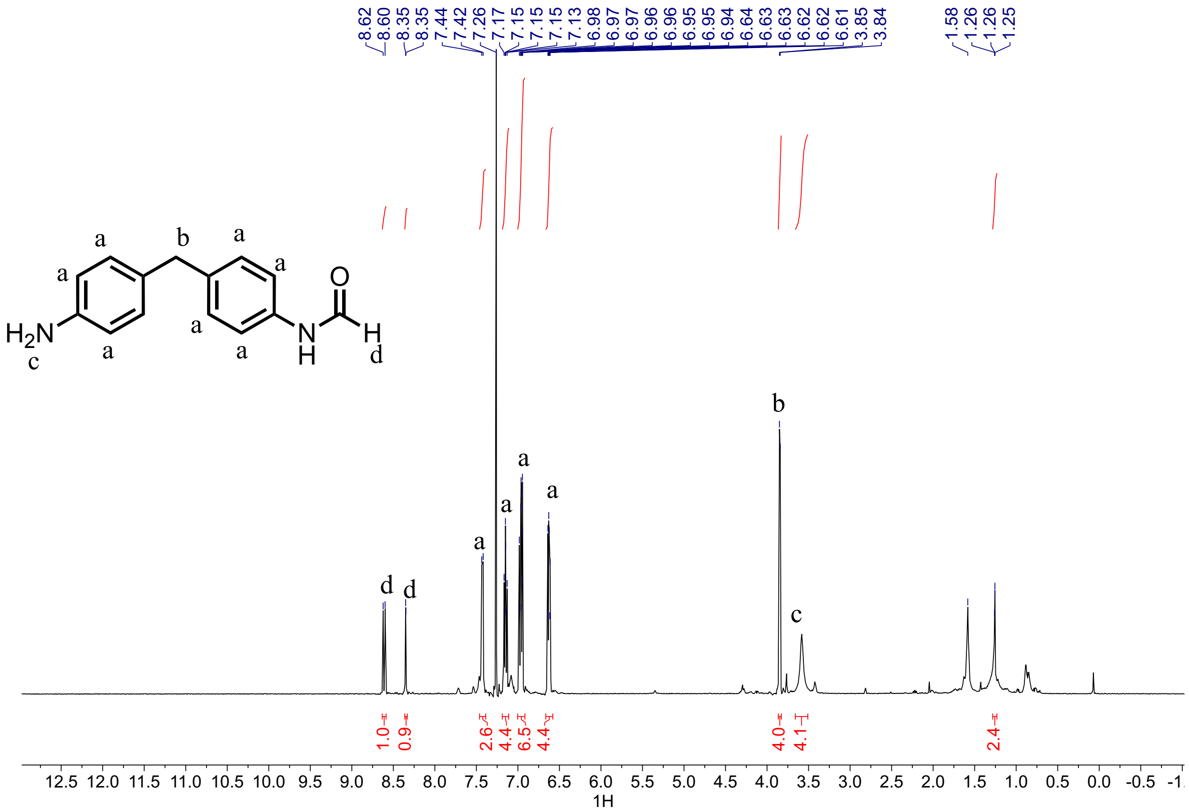

Figure 100: ^1H NMR (500 MHz, $CDCl_3$) spectrum of amine-formamide isolated from the hydrogenative depolymerization of TPU containing polyetherol (Figure 3A, main manuscript).

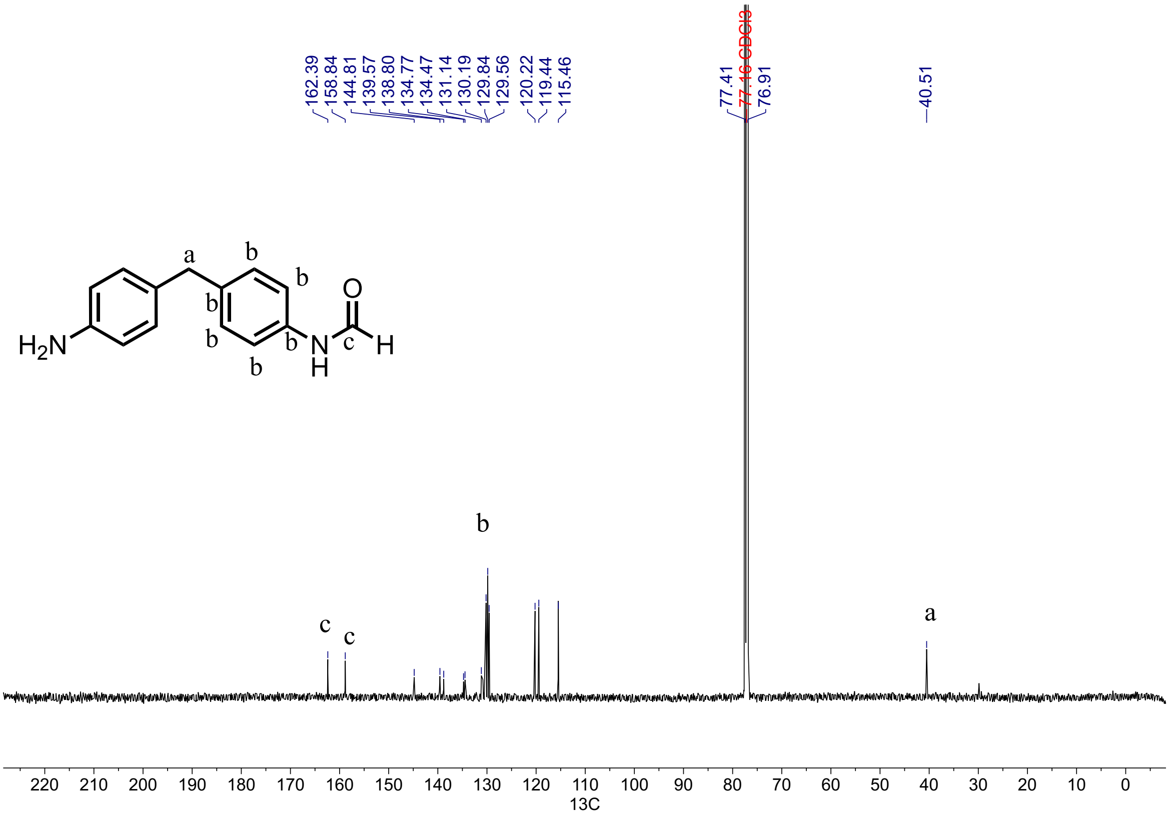

Figure 101: $^{13}C\{^1H\}$ NMR (125 MHz, $CDCl_3$) spectrum of amine-formamide isolated from the hydrogenative depolymerization of TPU containing polyetherol (Figure 3A, main manuscript).

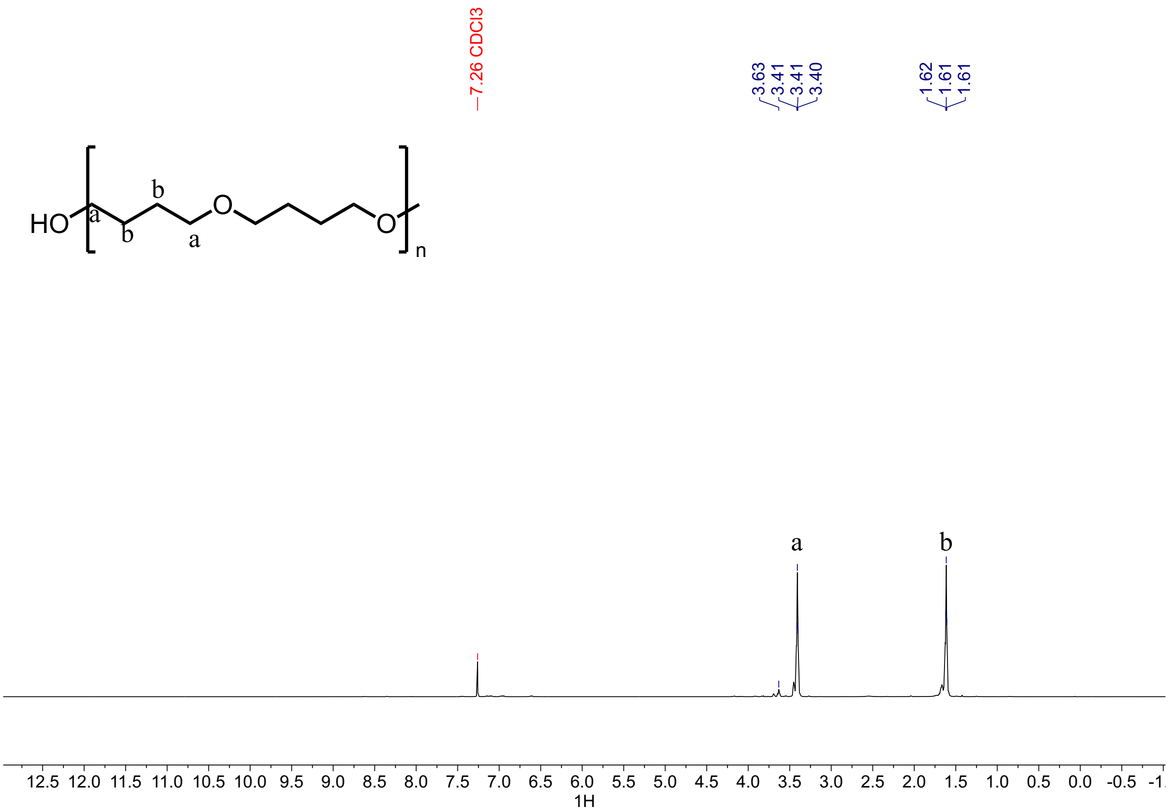

Figure 102: ^{1}H NMR (500 MHz, $CDCl_3$) spectrum of polyol isolated from the hydrogenative depolymerization of TPU containing polyetherol (Figure 3A, main manuscript).

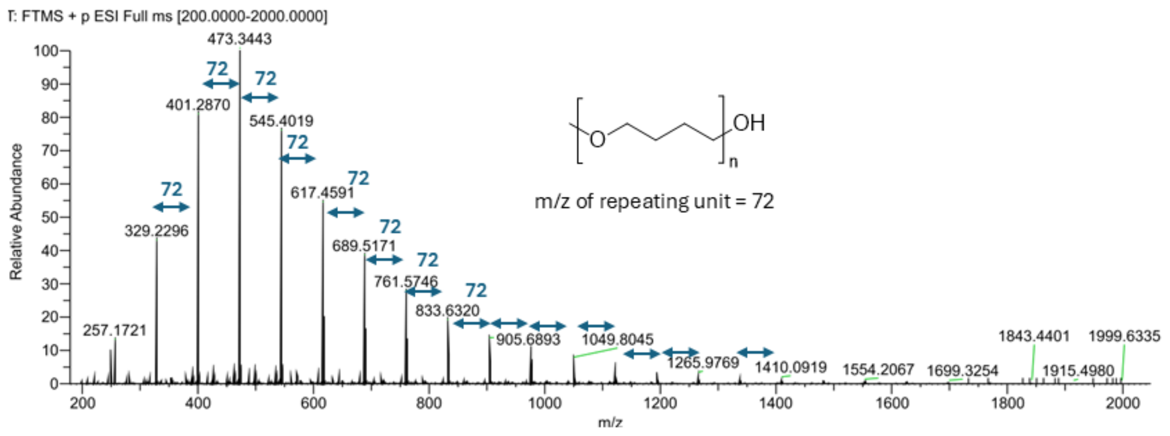

***Figure 103**. ESI-MS data for polyol obtained from the hydrogenative depolymerization of TPU containing polyetherol (**PU1**, Figure 3A, main manuscript).*

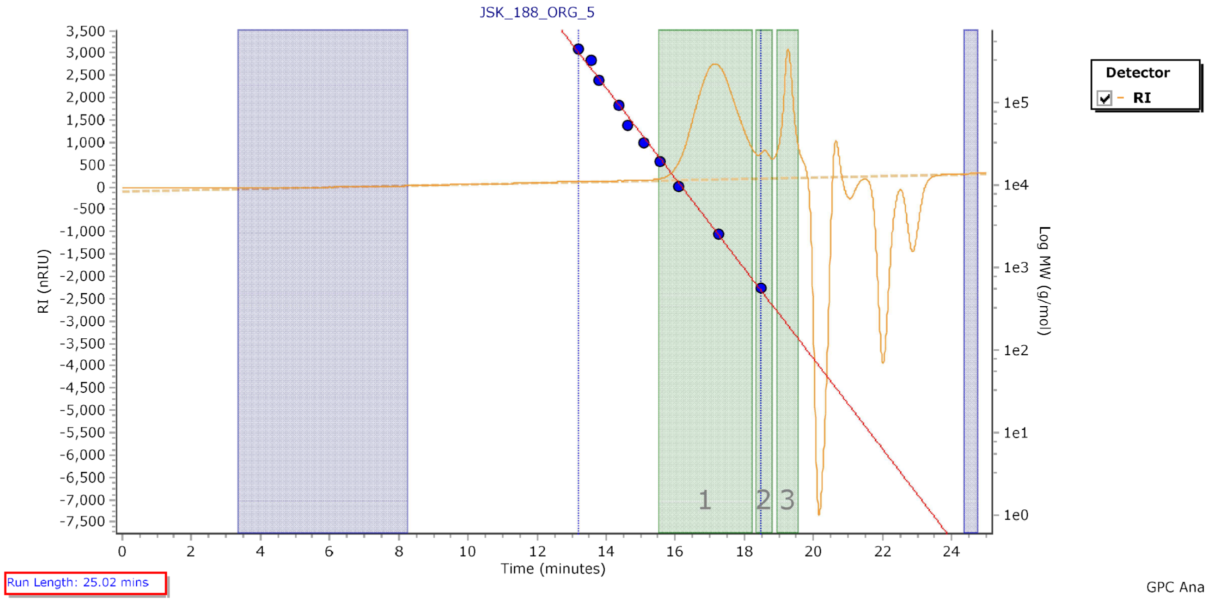

Figure 104: Gel-permeation chromatography (GPC) trace of the product mixture obtained from the hydrogenation of polyether TPU in THF as solvent system.

Molecular Weight Averages

Peak	Mp (g/mol)	Mn (g/mol)	Mw (g/mol)	Mz (g/mol)	Mz+1(g/mol)	Mv (g/mol)	PD
Peak 1	2819	2258	3480	5354	7751	3260	1.541
Peak 2	458	458	473	488	503	471	1.033
Peak 3	195	191	198	204	211	197	1.037

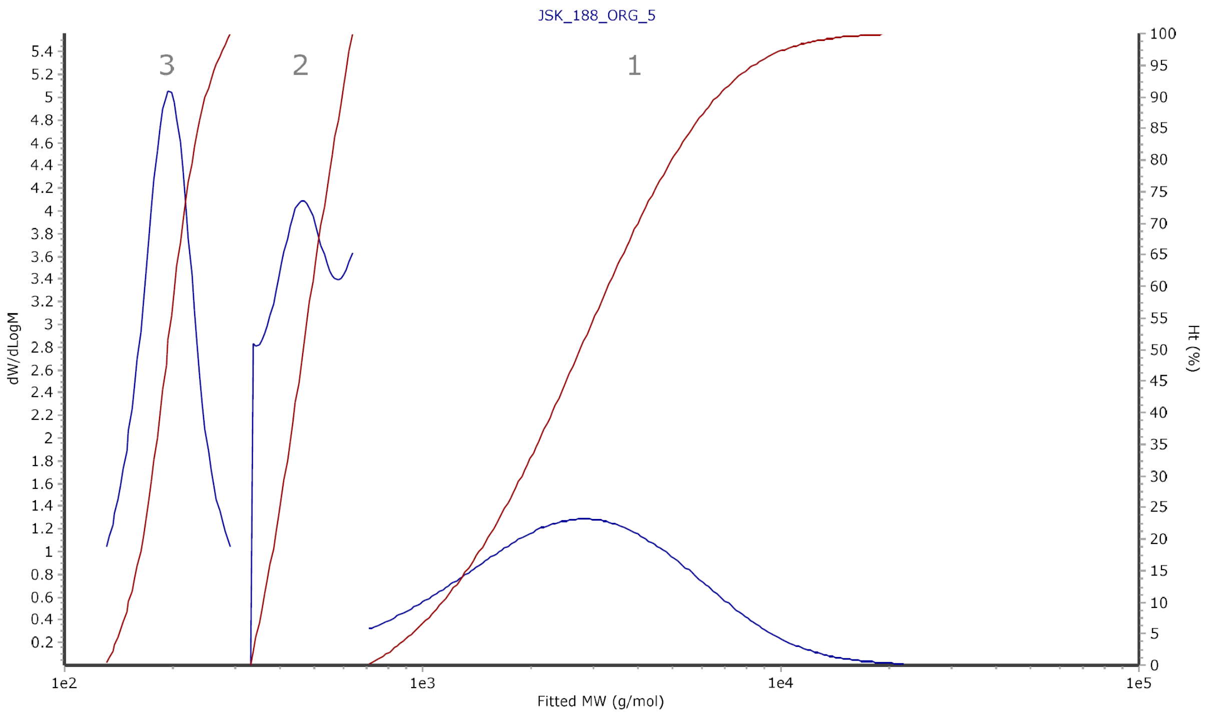

Figure 105: GPC data analysis and analyticalal gel-permeation chromatography (GPC) trace of the product mixture obtained from the hydrogenation of polyether TPU in THF as solvent system.

Analyticalal data of products obtained from the hydrogenation of polyester TPU (PU2):

Analyticalal data of diamines and polyols obtained from the hydrogenative depolymerization of polyurethanes match well with the literature.[11–15]

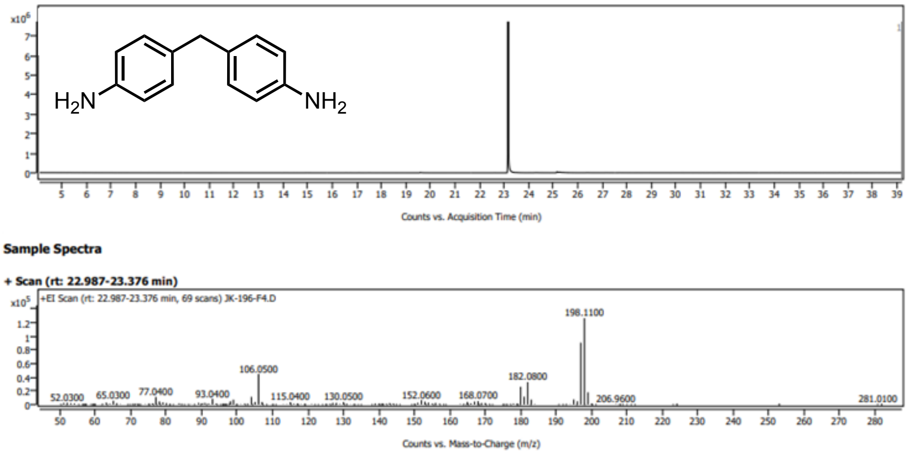

Figure 106: GC-MS data of diamine isolated from the hydrogenative depolymerization of TPU containing polyesterol (Figure 3B, main manuscript).

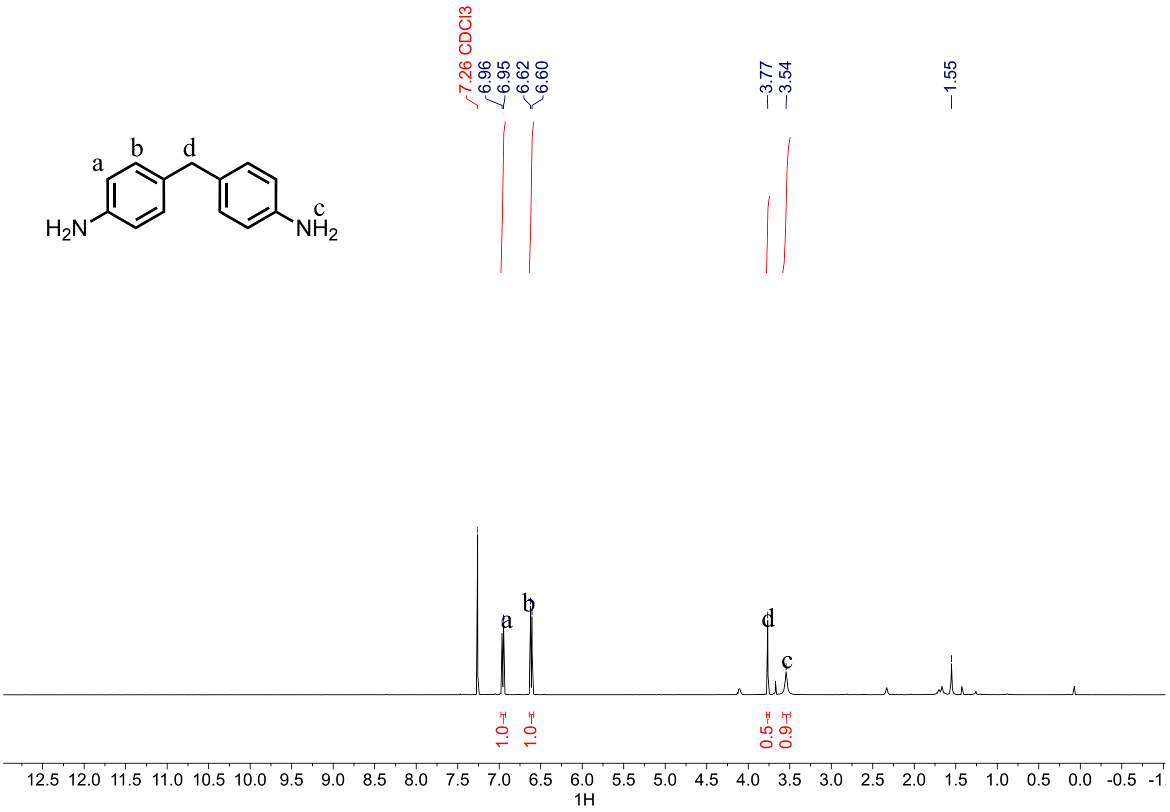

Figure 107: ^{1}H NMR (500 MHz, $CDCl_3$) spectrum of diamine isolated from the hydrogenative depolymerization of TPU containing polyesterol (Figure 3B, main manuscript).

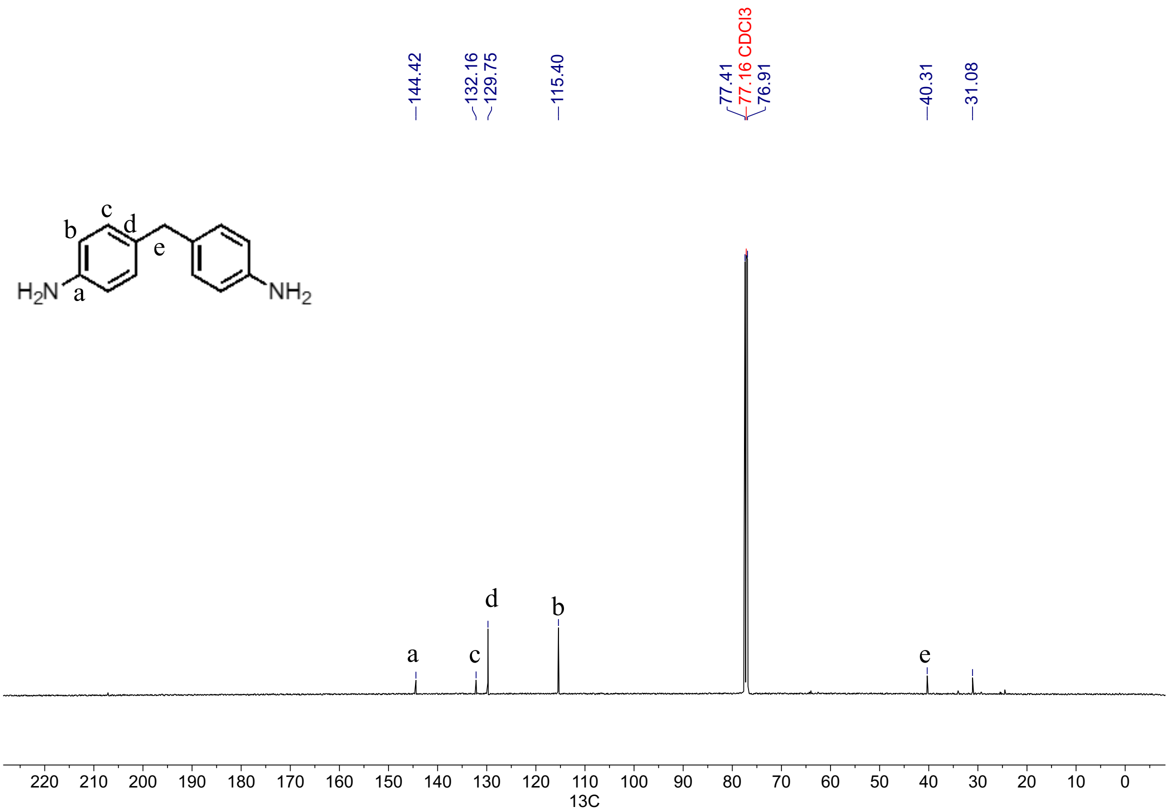

Figure 108: $^{13}C\{^1H\}$ NMR (125 MHz, $CDCl_3$) spectrum of diamine isolated from the hydrogenative depolymerization of TPU containing polyesterol (Figure 3B, main manuscript).

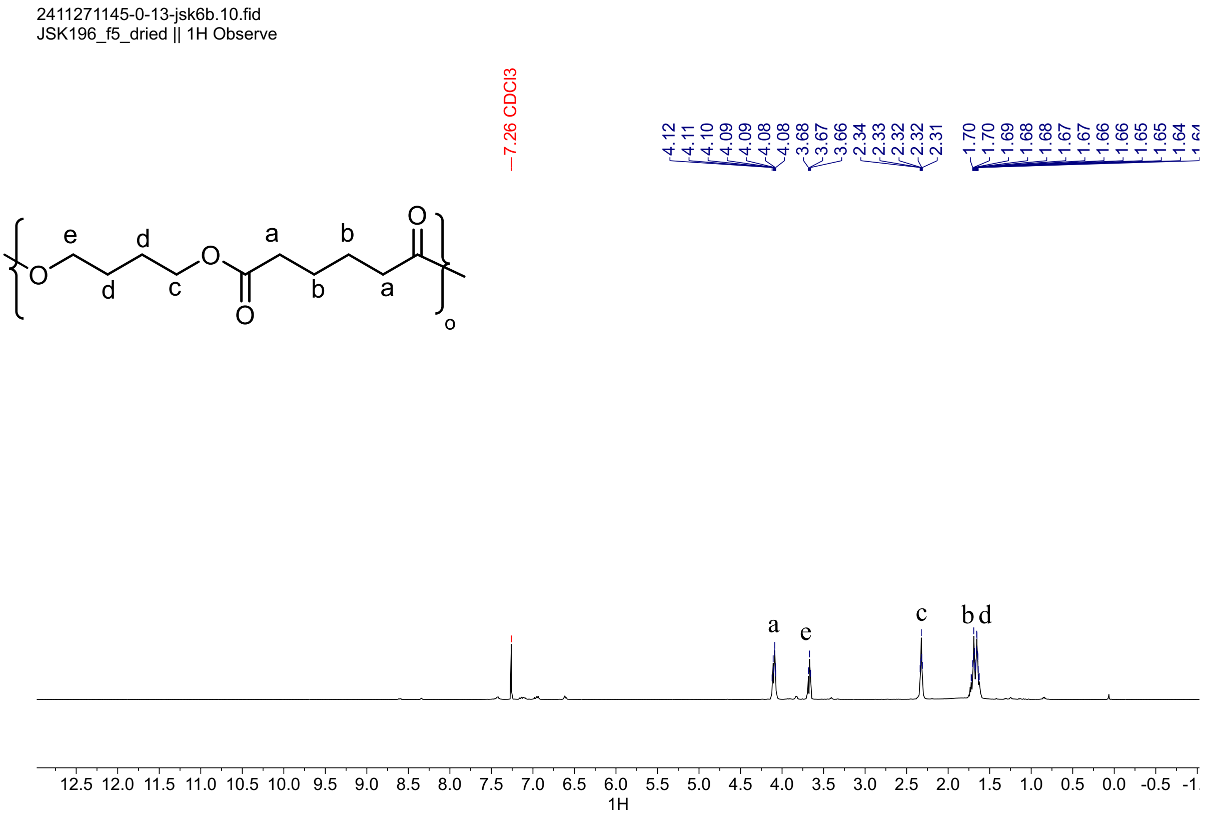

Figure 109: ^{1}H NMR (500 MHz, $CDCl_3$) spectrum of polyol isolated from the hydrogenative depolymerization of TPU containing polyesterol (Figure 3B, main manuscript).

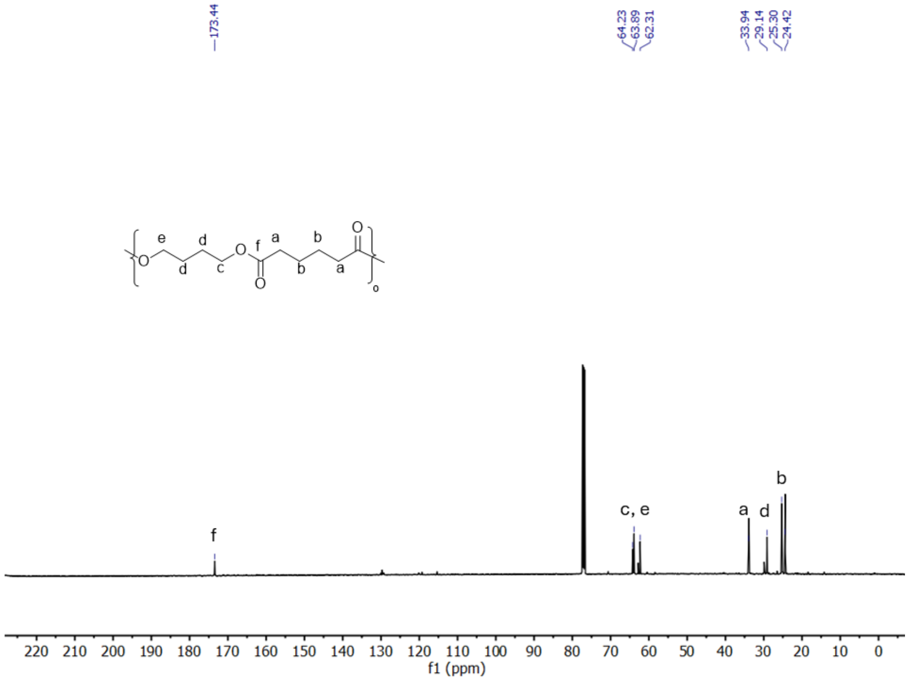

Figure 110:^{13}C{^{1}H} NMR (500 MHz, $CDCl_3$) spectrum of polyol isolated from the hydrogenative depolymerization of TPU containing polyesterol (Figure 3B, main manuscript).

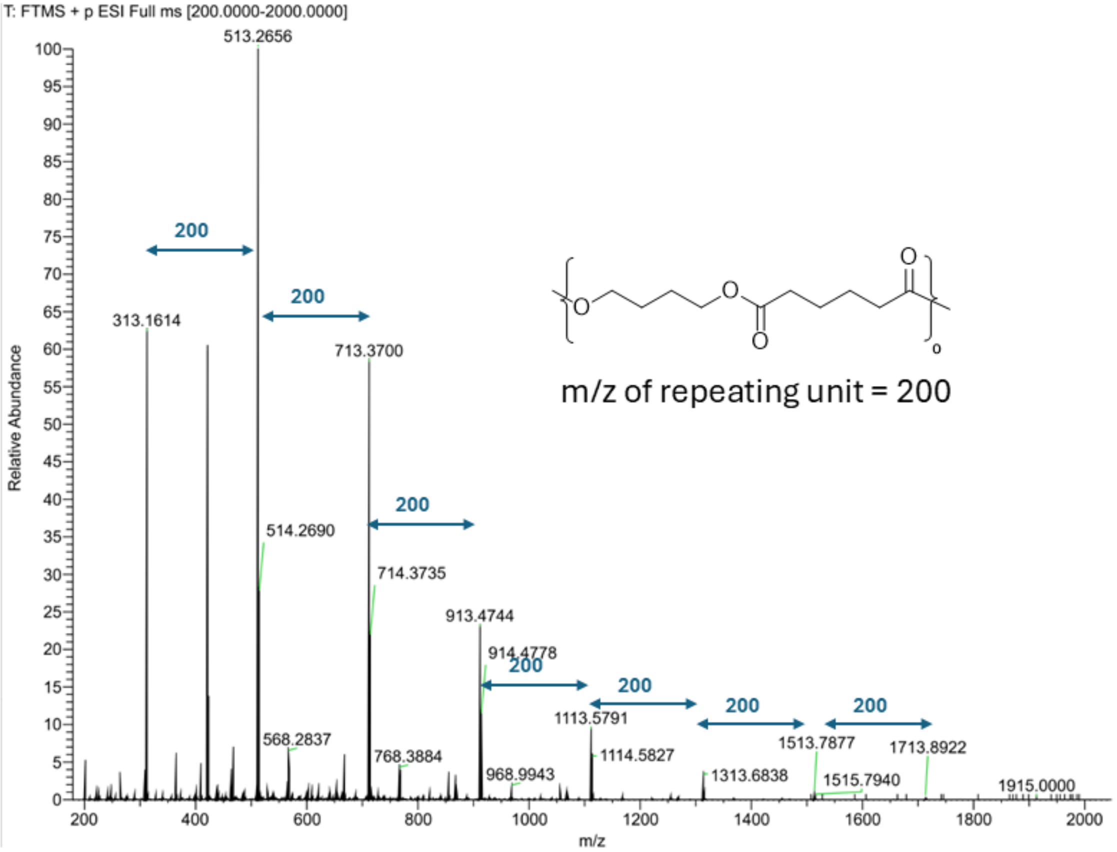

Figure 111: GC-MS data of polyol isolated from the hydrogenative depolymerization of TPU containing polyesterol (Figure 3B, main manuscript).

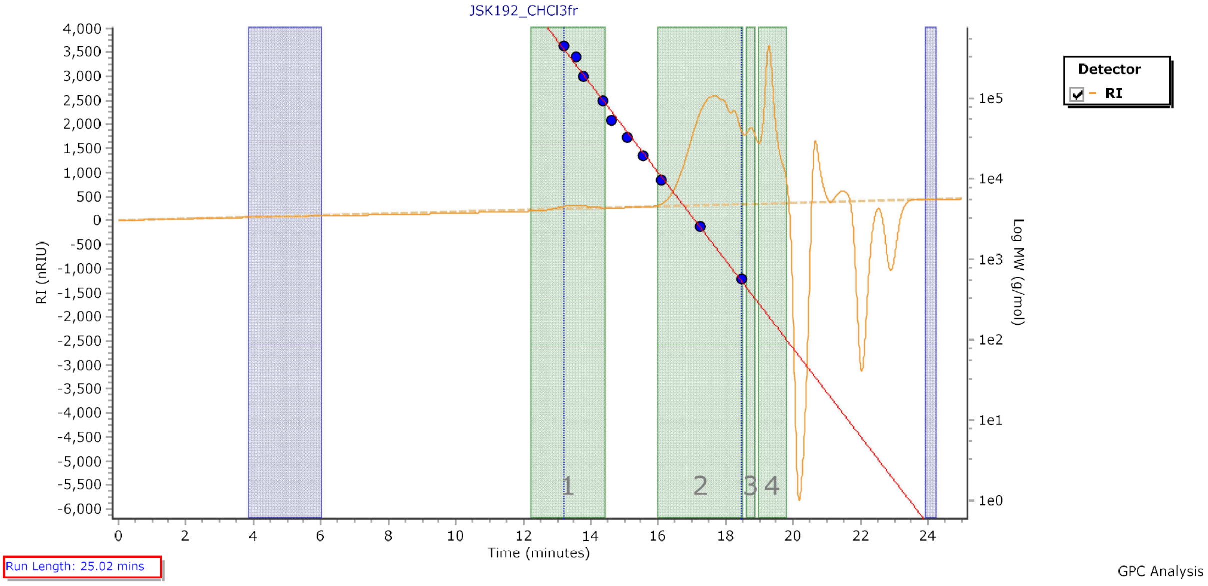

Figure 112: Gel-permeation chromatography (GPC) trace of the product mixture obtained from the hydrogenation of polyester TPU in THF as solvent system.

Molecular Weight Averages

Peak	Mp (g/mol)	Mn (g/mol)	Mw (g/mol)	Mz (g/mol)	Mz+1(g/mol)	Mv (g/mol)	PD
Peak 1	300283	243096	292770	343577	390402	285452	1.204
Peak 2	1427	1249	1964	3118	4504	1829	1.572
Peak 3	369	361	365	369	373	365	1.011
Peak 4	190	174	185	195	205	183	1.063

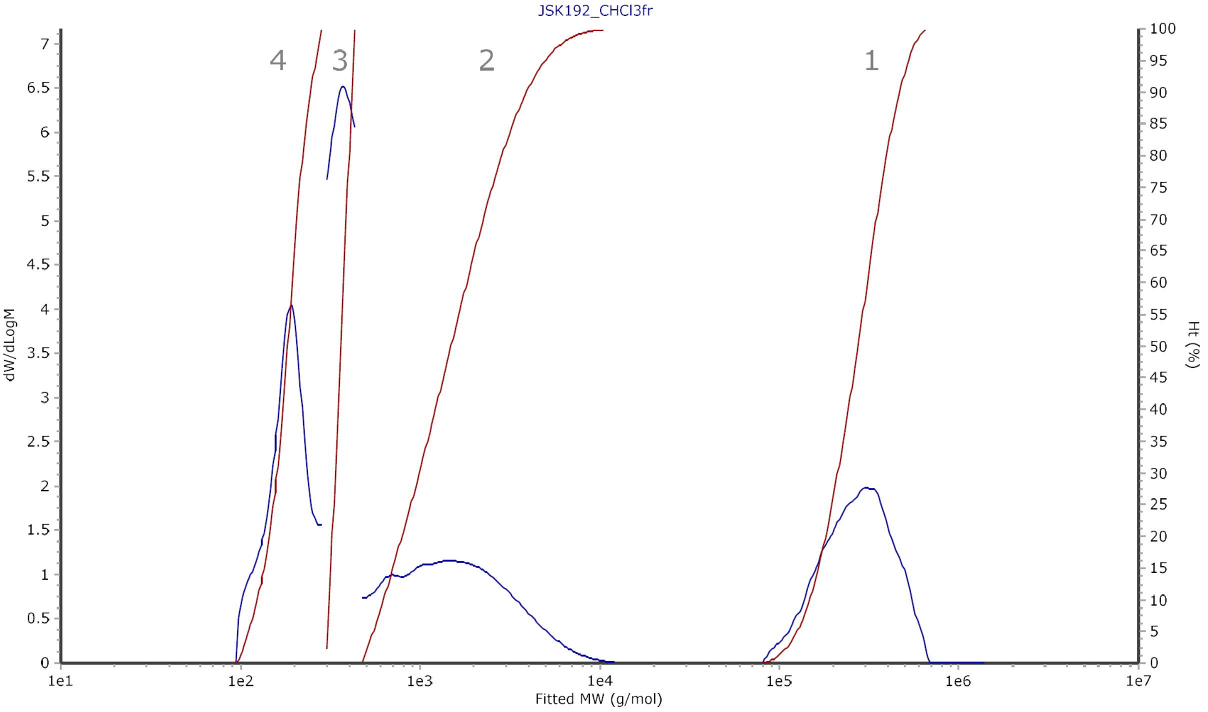

Figure 113: GPC data analysis and analyticalal gel-permeation chromatography (GPC) trace for product mixture obtained from the hydrogenation of polyester TPU in THF as solvent system.

Analyticalal data of products obtained from the hydrogenation of sponge:

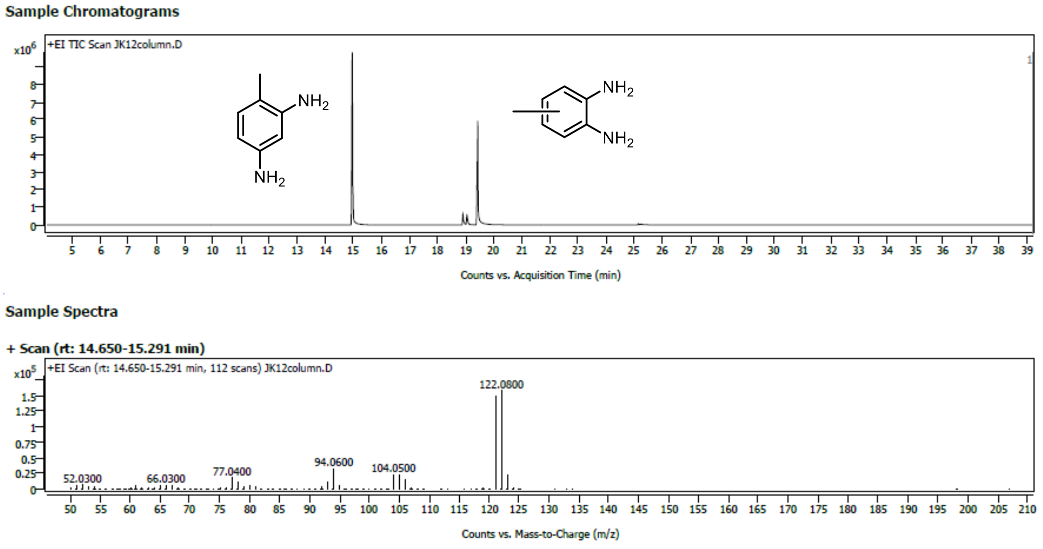

Figure 114: GC-MS data for diamine recovered from the hydrogenation of sponge.

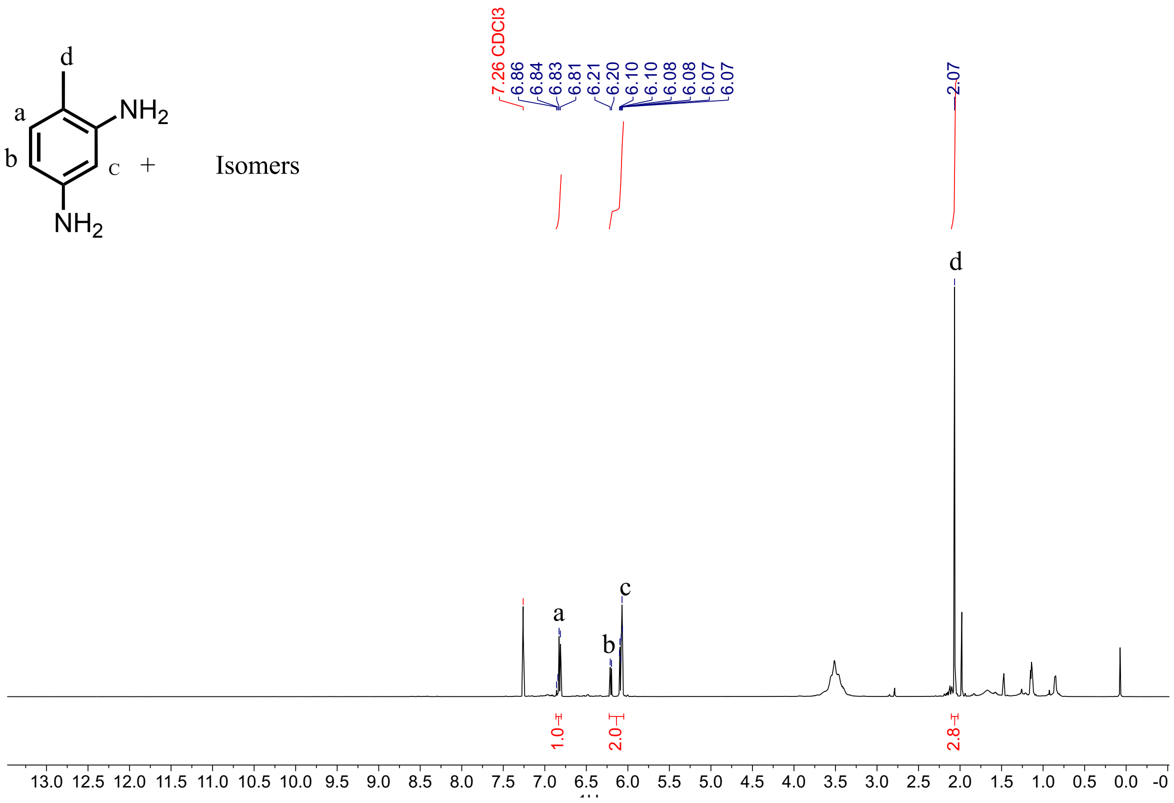

Figure 115: ¹H NMR (500 MHz, $CDCl_3$) spectrum of diamine isolated from the hydrogenative depolymerization of sponge (Figure 3C, main manuscript).

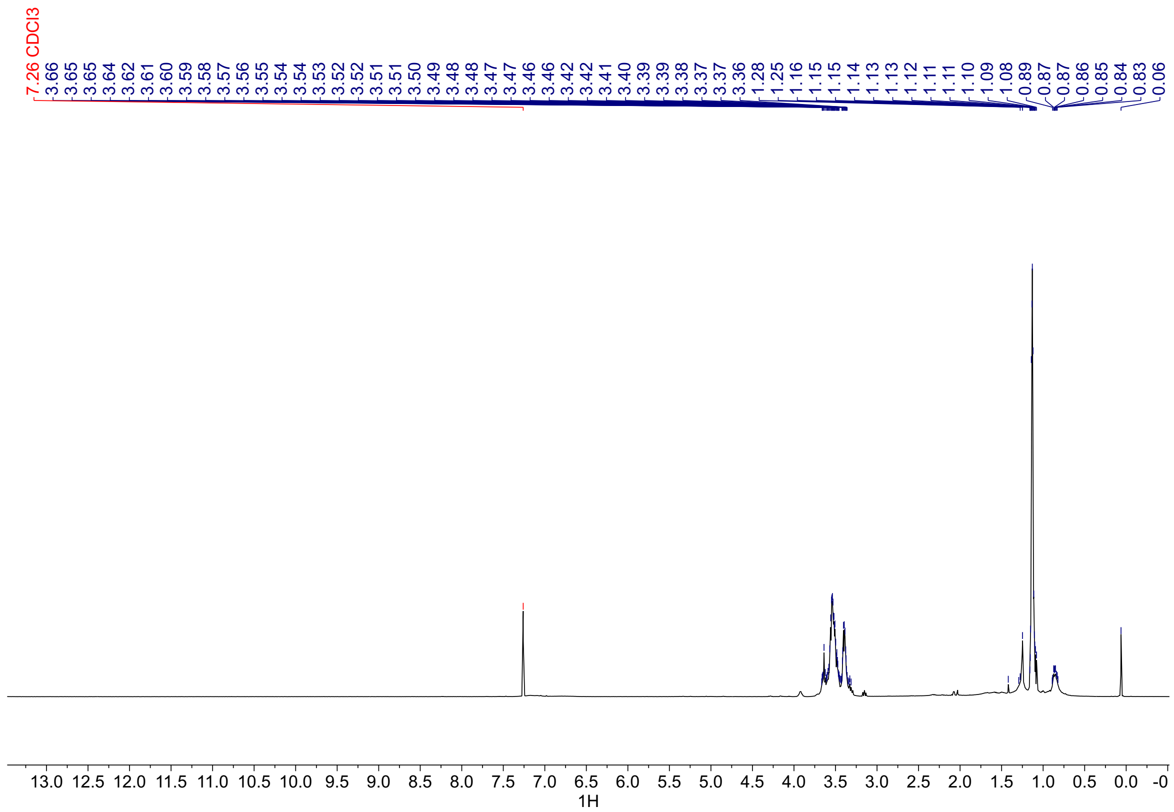

Figure 116: ¹H NMR (500 MHz, $CDCl_3$) spectrum of polyol isolated from the hydrogenative depolymerization of sponge (Figure 3C, main manuscript).

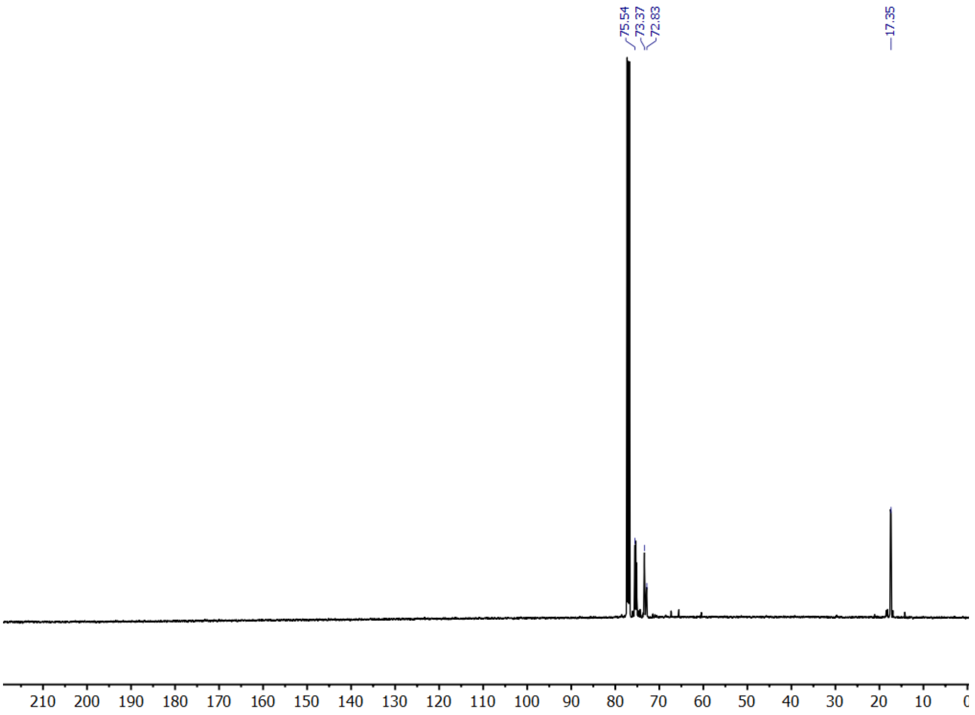

Figure 117: $^{13}C\{^1H\}$ NMR (500 MHz, $CDCl_3$) spectrum of polyol isolated from the hydrogenative depolymerization of sponge (Figure 3C, main manuscript).

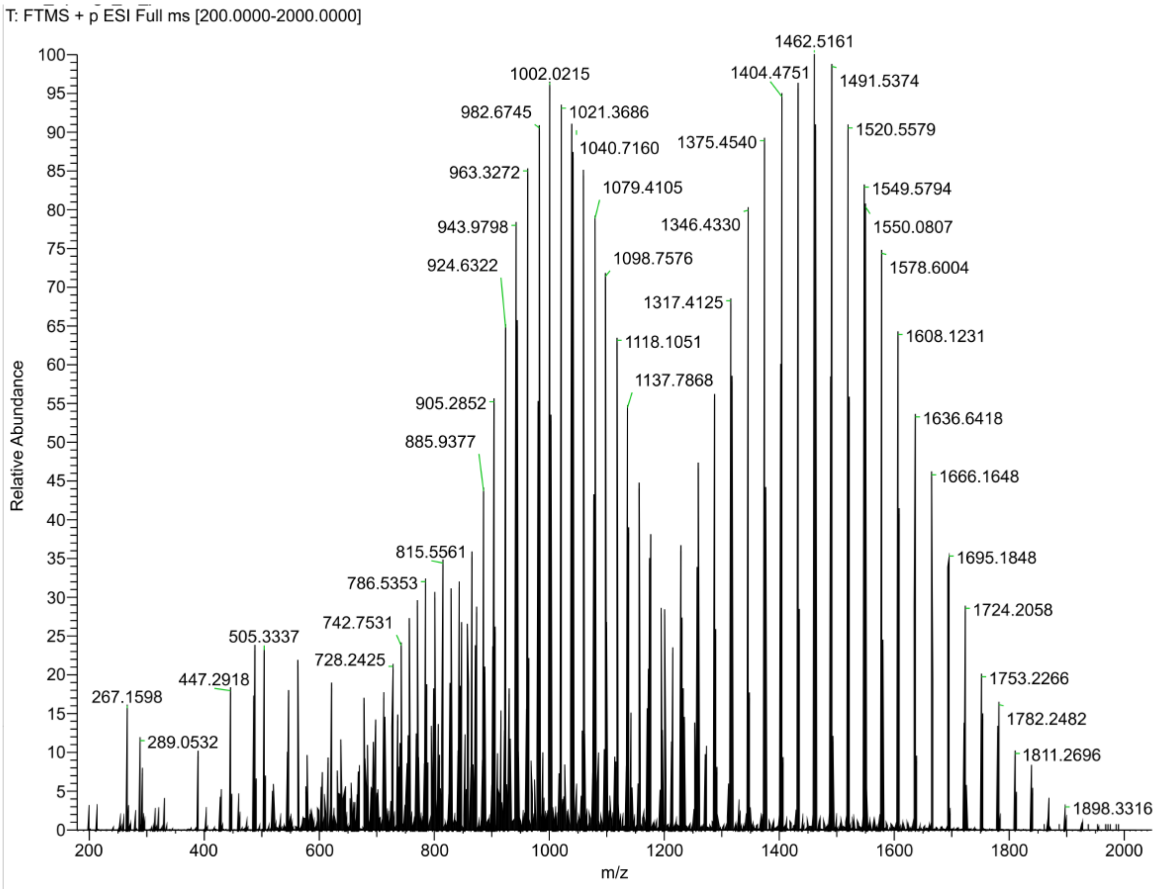

Figure 118: ESI-MS of the polyol isolated from the hydrogenative depolymerization of sponge (Figure 3C, main manuscript).

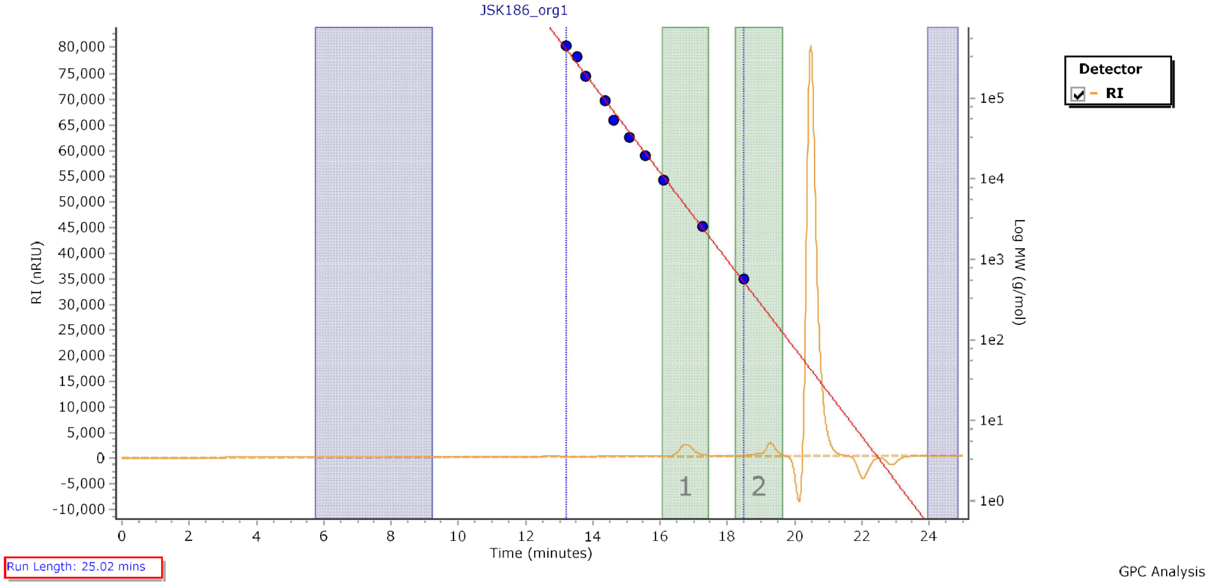

Figure 119: Gel-permeation chromatography (GPC) trace of the product mixture obtained from the hydrogenation of sponge.

Molecular Weight Averages

Peak	Mp (g/mol)	Mn (g/mol)	Mw (g/mol)	Mz (g/mol)	Mz+1(g/mol)	Mv (g/mol)	PD
Peak 1	4521	4110	4512	4939	5409	4452	1.098
Peak 2	190	204	232	278	346	227	1.137

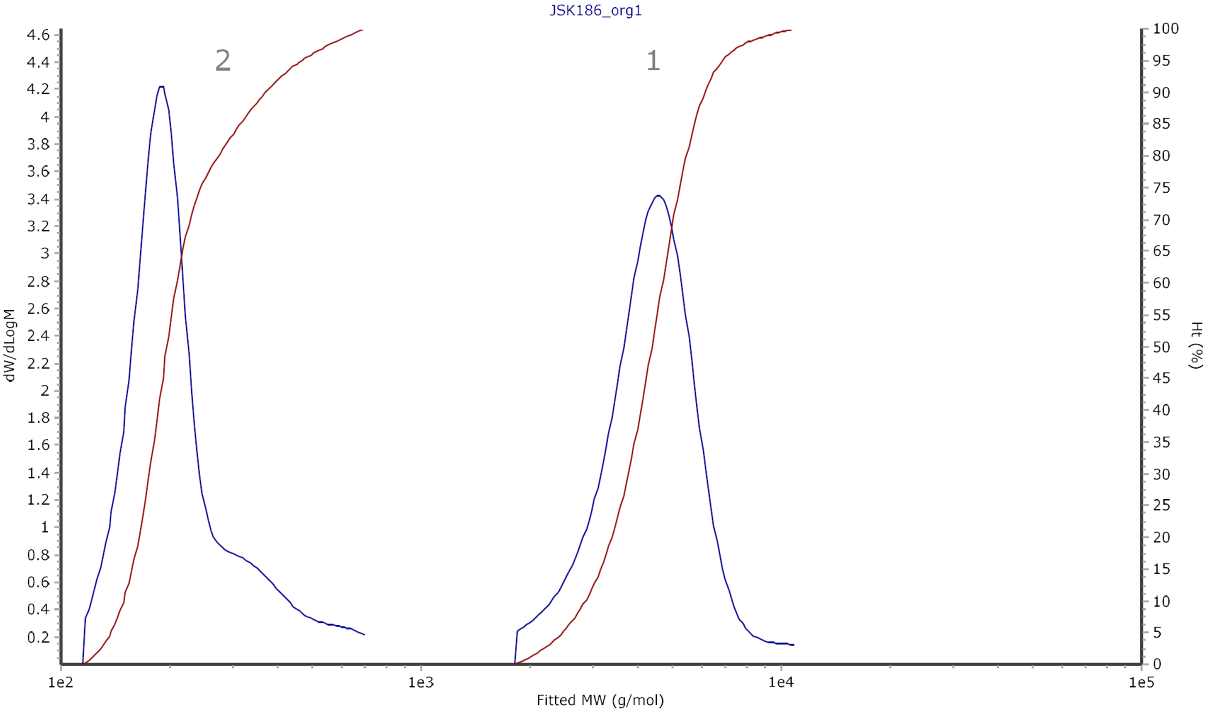

Figure 120: GPC data analysis and analyticalal gel-permeation chromatography (GPC) trace for the product mixture obtained from the hydrogenation of sponge.

6. MP-AES study

Residual palladium analysis was performed using 4210 Microwave plasma atomic emission spectrometer (MP-AES) from Agilent technologies. A 2% HNO_3 solution was prepared as stock solution to further prepare the samples for the calibration curve and the analysed samples. Blank samples were used directly from the 2% HNO_3 stock solution, meanwhile the calibration curve was prepared by diluting a 1000 ppm Pd standard solution into 2, 10, 20 and 30 ppm solutions. The calibration curve and the samples were analysed by triplicate at the characteristic's wavelengths for palladium (324.27, 340.45, 342.12 & 363.47, see below for the calibration example at 340.45nm). The samples were prepared by dispersing the crude reaction of TPU (recycling samples 2,3) into the HNO_3 stock solution to generate a 1mg/mL sample solution. The samples were tested directly without further dilution.

No palladium was detected in the reaction mixture after separation of the catalyst confirming catalyst's stability.

Calibration Standard for Pd (340.45 nm)

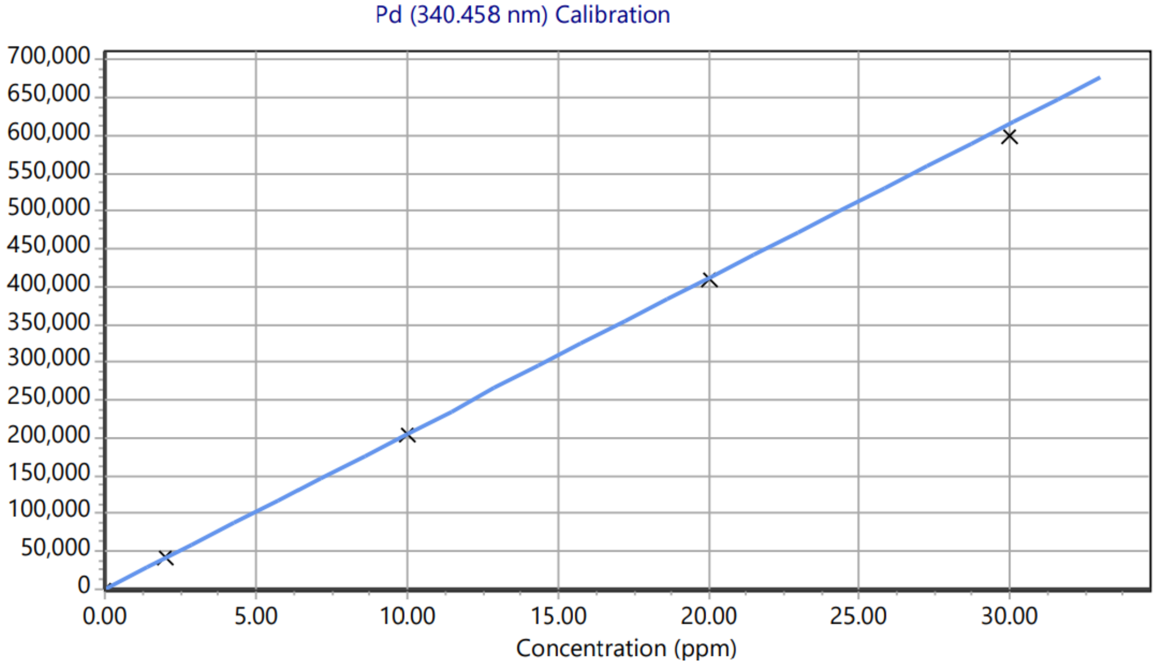

*Figure 121: Calibration Standard for Pd at 340.458 nm. (Y axis) Intensity= 20546.71*Concentration+19.16088735 Correlation coefficient: 0.99984*

Sample: Reaction mixture after catalyst separation from the hydrogenative depolymerisation of polyether TPU (PU1)

Label	Replicate 1	Replicate 2	Replicate 3	Units
Pd (324.270 nm)	0.00333513	-0.00407391	-0.00081400	ppm
Pd (340.458 nm)	-0.01100549	-0.01192132	-0.01167497	ppm
Pd (342.124 nm)	0.00666897	0.00583764	0.00522744	ppm
Pd (360.955 nm)	-0.00163560	-0.00218476	-0.00084255	ppm
Pd (363.470 nm)	-0.00056539	-0.00161732	-0.00092664	ppm

Sample: Reaction mixture after catalyst separation from the hydrogenative depolymerisation of polyether TPU (PU1) from the 1st catalyst recycling

Label	Replicate 1	Replicate 2	Replicate 3	Units
Pd (324.270 nm)	0.00294808	0.00206554	0.00421054	ppm
Pd (340.458 nm)	-0.0139443	-0.01247201	-0.01162333	ppm
Pd (342.124 nm)	0.00405048	0.00766258	0.00742696	ppm
Pd (360.955 nm)	0.00064742	-0.00140431	-0.00111286	ppm
Pd (363.470 nm)	-0.00050451	-0.00080454	-0.00003159 u	ppm

7. TGA-MS study for the thermal cracking of phenyl *N*-octyl carbamate

To verify our hypothesis that the hydrogenation of carbamates occurs through an isocyanate intermediate, we studied the thermal cracking of phenyl *N*-octyl carbamate using TGA-MS (Thermogravimetric Analysis - Mass Spectrometry). In this study, phenyl N-octyl carbamate (~65 mg) in the presence of Pd/Al_2O_3 (13 mg) or Pd/C (5 mg) as well as without any metal catalyst was heated in an N_2 atmosphere at 150 ºC for 1 h. Mass loss was monitored over time along with the release of molecular species by mass spectrometry. Interestingly, in all cases, the formation of *N*-octyl isocyanate was observed by mass spectrometry suggesting that under the reaction conditions, thermal dissociation of carbamates to isocyanates and alcohols is possible, and the rate of dissociation gets enhanced in the presence of Pd/Al_2O_3 and Pd/C.

Method: TGA-MS was run in an Al_2O_3 crucible under N_2 (50 ml/min). The samples were heated to 120 °C at 5 K/min and then to 150 °C at 2 K/min. The samples were held for an isothermal phase at 150 °C for 1 hour. The MS detector was active between the ranges of 40 and 160 amu, with a step width of 1 amu. A SCB scan type was chosen. The cathode was set to –70 V and SEM to 800 V.

phenyloctyl carbamate (64.2 mg) and Pd/Al (13 mg)

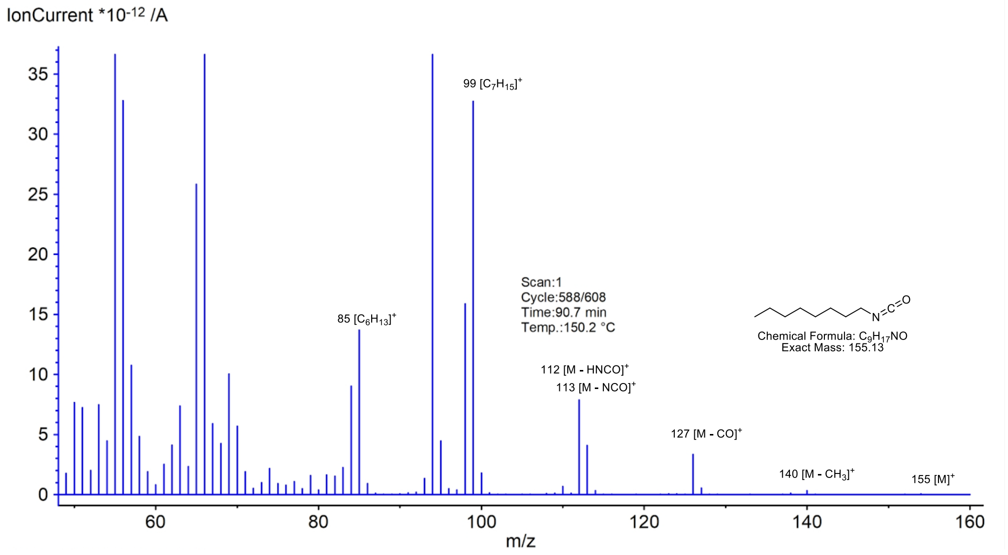

phenyl octylcarbamate (65 mg) and Pd/C (5 mg)

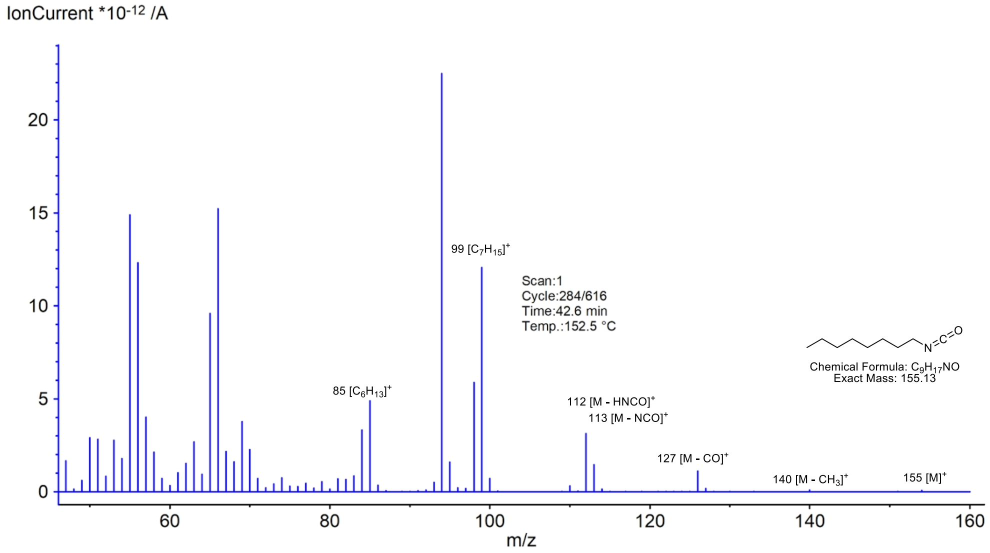

Phenyl octylcarbamate only (65.3 mg)

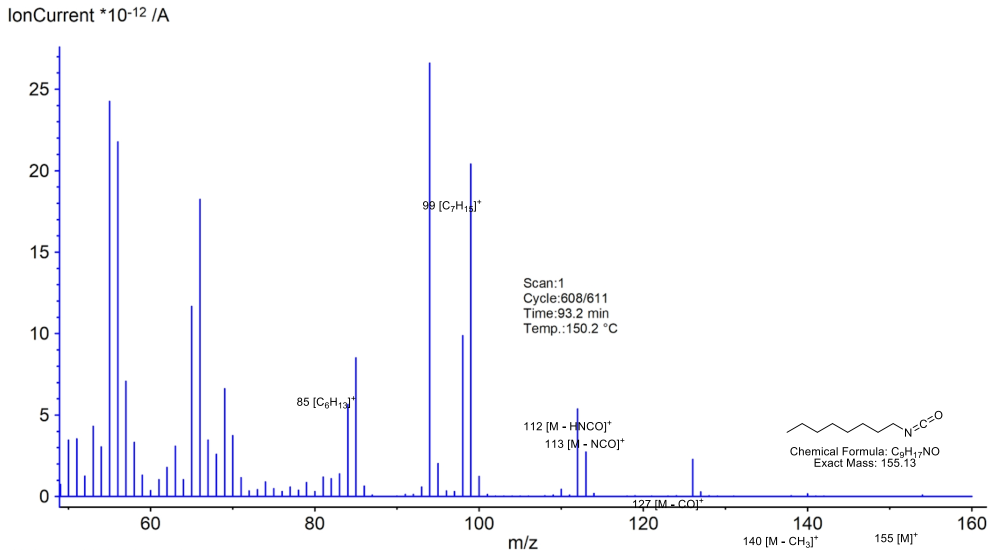

Figure 122: *MS data from the TGA-MS study of phenyl N-octyl carbamate.*

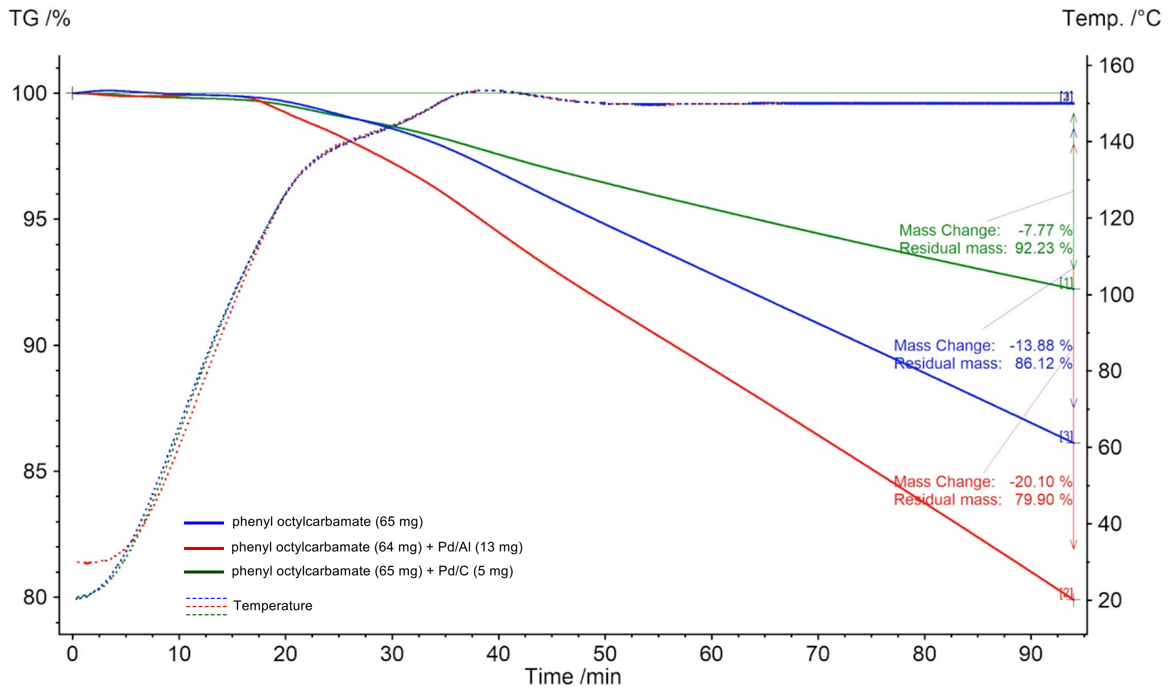

Figure 123. *Thermal data from the TGA-MS study of phenyl N-octyl carbamate.*

8. Powder XRD:

Powder XRD of the commercial Pd/Al_2O_3 was compared with the catalyst separated after the hydrogenation of isocyanate (Table 1, entry 6). Catalyst was separated by filtration followed by subsequent drying.

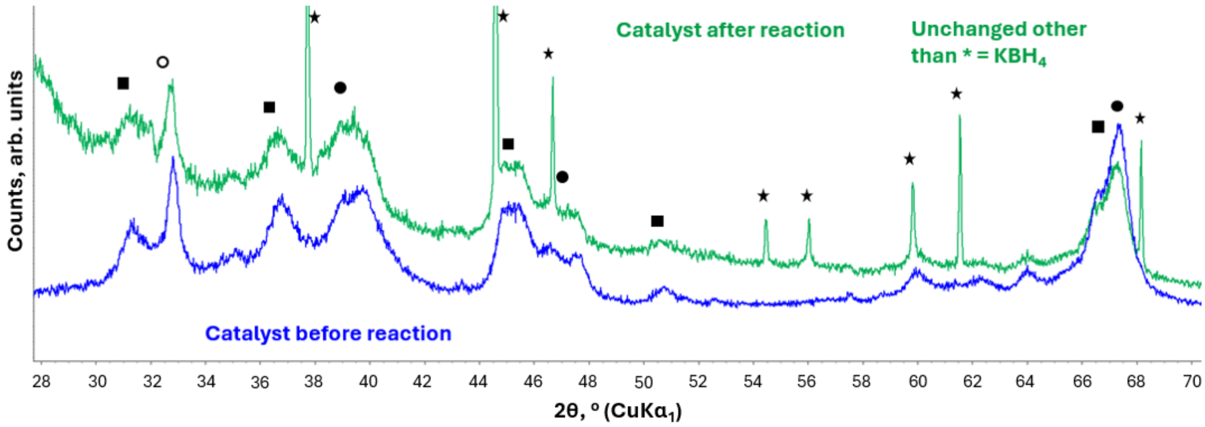

*Figure 124: Powder XRD pattern of Pd/Al_2O_3 sample (blue) as well as the catalyst recovered after the hydrogenation of isocyanate, Table 1, entry 6 (green), * represents signals from KBH_4; • – Pd; ◦ – PdO; ▪ – γ-Al_2O_3.*

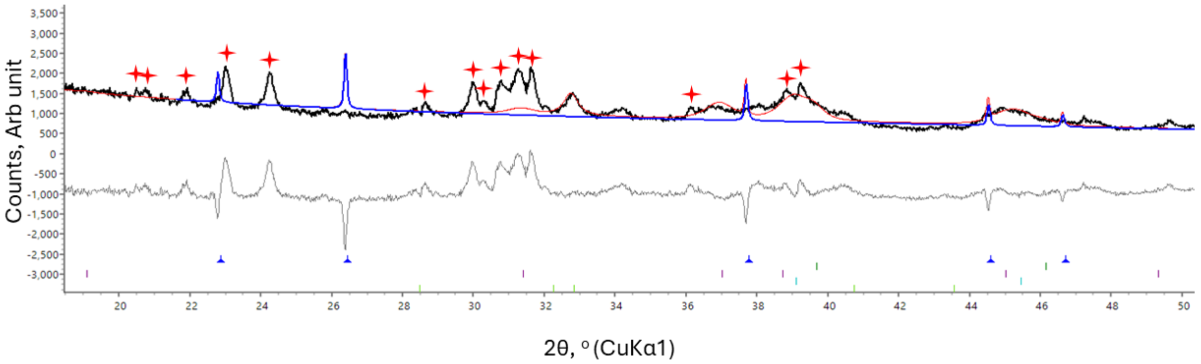

Figure 125: Black line – measured. Red line- fitting using Pd, PdO, PdH, gamma-Al_2O_3. Blue line – where peaks of KBH_4 should be located, but there are no measured peaks confirming there is no KBH_4 within detectable limits of p-XRD. Labelled signals are possibly due to semicrystalline organic molecules such as carbamates, phenol, formamides, urea.

9. Polyurethane (PU1) recycling

Experimental procedure:

In a 100 mL reactor, 1.65 g of commercial flexible polyurethane (**PU1**) and 16.5 mg of base (KBH_4, 1 wt%) were charged, followed by the addition of 165 mg of Pd/Al_2O_3 (10 wt% relative to polymer) under ambient conditions. The reactor was sealed and purged with argon, after which 25 mL of dry, degassed tert-amyl alcohol was introduced under an inert atmosphere. The reactor was then purged with H_2 to displace residual argon, pressurized to 50 bar H_2, and heated to 180 °C with stirring at 500 rpm for 24 h.
After completion of time, heating was stopped, and the reactor was allowed to cool to room temperature before releasing the hydrogen pressure. The reaction mixture was collected by decantation into a 50 mL Falcon tube and centrifuged at 3000 rpm, 18 °C for 10 min to separate the solid catalyst. The supernatant was decanted into a round-bottom flask, and the solvent was removed under reduced pressure to afford the crude product mixture. This mixture was dissolved in 15 mL of THF, and 1 mmol of diphenylethylene was added as an internal standard. An aliquot (1 mL) was further diluted to 2 mg/mL for GPC analysis. For NMR analysis, 20–30 mg of product was placed in a 5 mL vial, the THF was evaporated under vacuum, and the residue was dissolved in $CDCl_3$.
The recovered solid catalyst was washed three times with 30 mL of methanol. Each wash involved vigorous stirring for 3 min, centrifugation (3000 rpm, 18 °C, 10 min), and decantation of the supernatant. After washing, the catalyst was suspended in 5 mL of methanol or chloroform, concentrated using a rotary evaporator, and dried under high vacuum for at least 1 h. The dried catalyst was reused in subsequent experiments under identical conditions (polymer: 1.65 g; base: 16.5 mg).

Table S4. Catalyst recycling study for the depolymerisation of commercial polyurethane (PU1)

Cycle	Catalyst	Solvent	Base	Diamine yield (mmol)	M_n of polyol	M_w of polyol	*Đ* of polyol
1	Pd/Al_2O_3	*t*-amyl OH	KBH_4	1.18	1748	3224	1.84
2	Pd/Al_2O_3	*t*-amyl OH	KBH_4	1.20	1772	2987	1.68
3	Pd/Al_2O_3	*t*-amyl OH	KBH_4	1.22	1802	3029	1.68
4	Pd/Al_2O_3	*t*-amyl OH	KBH_4	1.33	1830	3107	1.69
5	Pd/Al_2O_3	*t*-amyl OH	KBH_4	1.24	1820	3270	1.79
6	Pd/Al_2O_3	*t*-amyl OH	KBH_4	1.26	1866	3195	1.71
7	Pd/Al_2O_3	*t*-amyl OH	KBH_4	1.18	1720	2938	1.71
8	Pd/Al_2O_3	*t*-amyl OH	KBH_4	1.24	1613	2754	1.70
9	Pd/Al_2O_3	*t*-amyl OH	KBH_4	1.23	1722	3190	1.85
10	Pd/Al_2O_3	*t*-amyl OH	KBH_4	1.47	1708	2978	1.74

All reactions were tested for 24 h at 180 °C under 50 H_2 bar of pressure, using 1.65g of the polymer and 10 wt% of the catalyst and 1 wt% of the base and tert-amyl alcohol as solvent (25 mL).

GPC Analysis

The following chromatograms correspond to each cycle of the catalyst recycling study, where the peak labelled as "1" corresponds to the polyol obtained from the depolymerisation reaction, the remaining peaks are under the calibration curve, and correspond to small molecules formed during the reaction (likely diamines).

Cycle 1

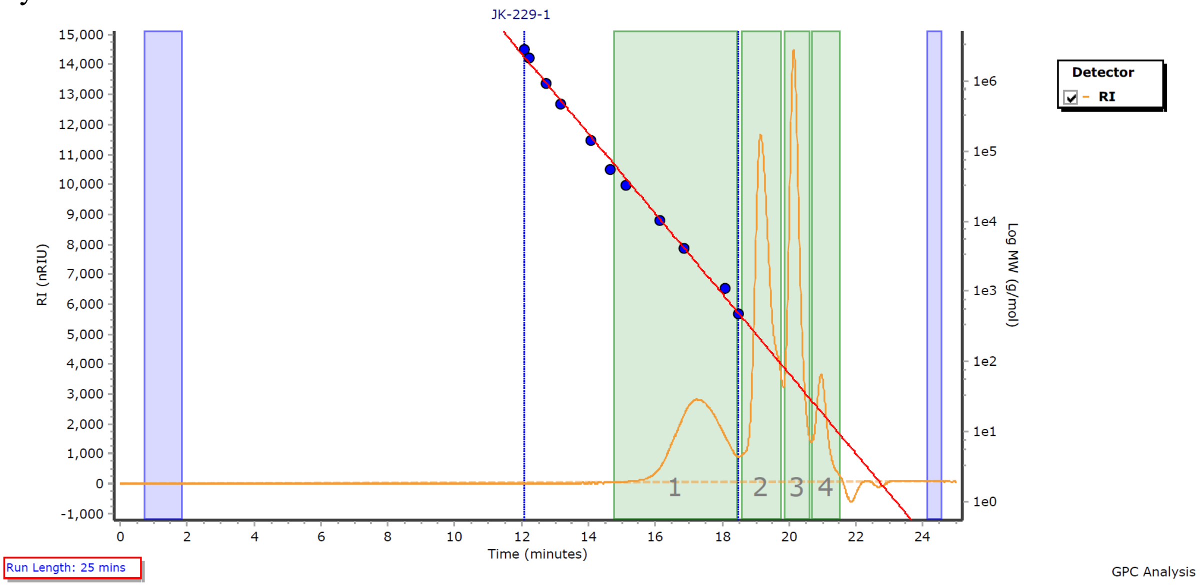

Molecular Weight Averages

Peak	Mp (g/mol)	Mn (g/mol)	Mw (g/mol)	Mz (g/mol)	Mz+1 (g/mol)	Mv (g/mol)	PD
Peak 1	2473	1748	3224	6412	12533	2924	1.844
Peak 2	200	168	187	207	228	184	1.113
Peak 3	54	51	53	56	58	53	1.039
Peak 4	19	17	18	19	20	18	1.059

Cycle 2

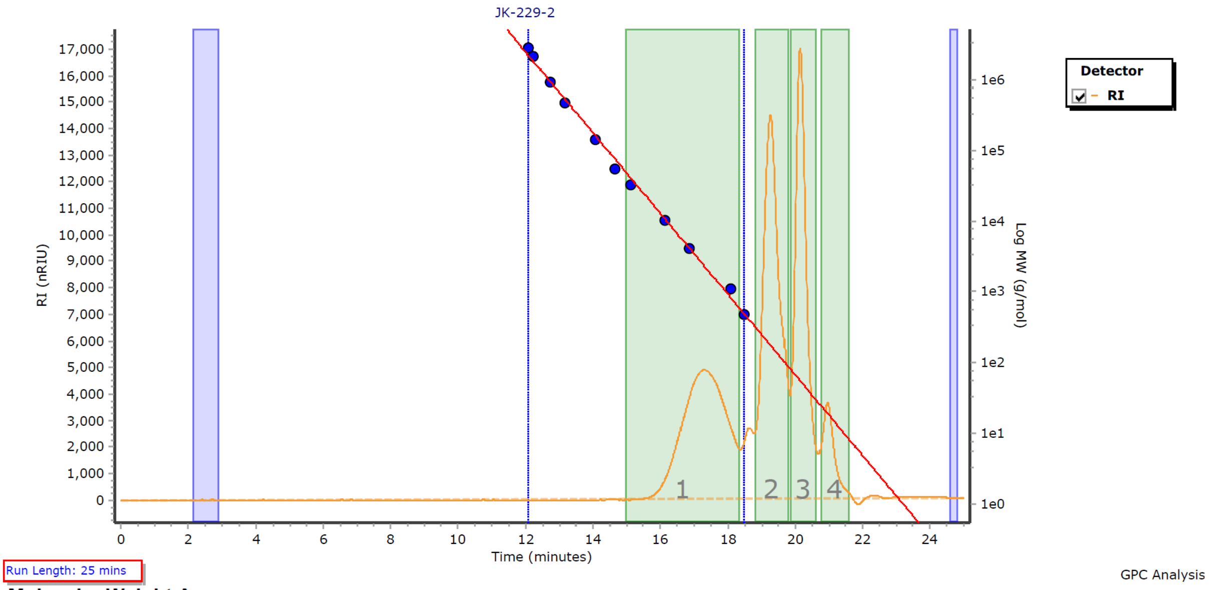

Molecular Weight Averages

Peak	Mp (g/mol)	Mn (g/mol)	Mw (g/mol)	Mz (g/mol)	Mz+1 (g/mol)	Mv (g/mol)	PD
Peak 1	2250	1772	2987	4979	7439	2760	1.686
Peak 2	172	154	169	184	198	167	1.097
Peak 3	54	50	52	55	57	52	1.04
Peak 4	19	17	18	18	19	17	1.059

Cycle 3

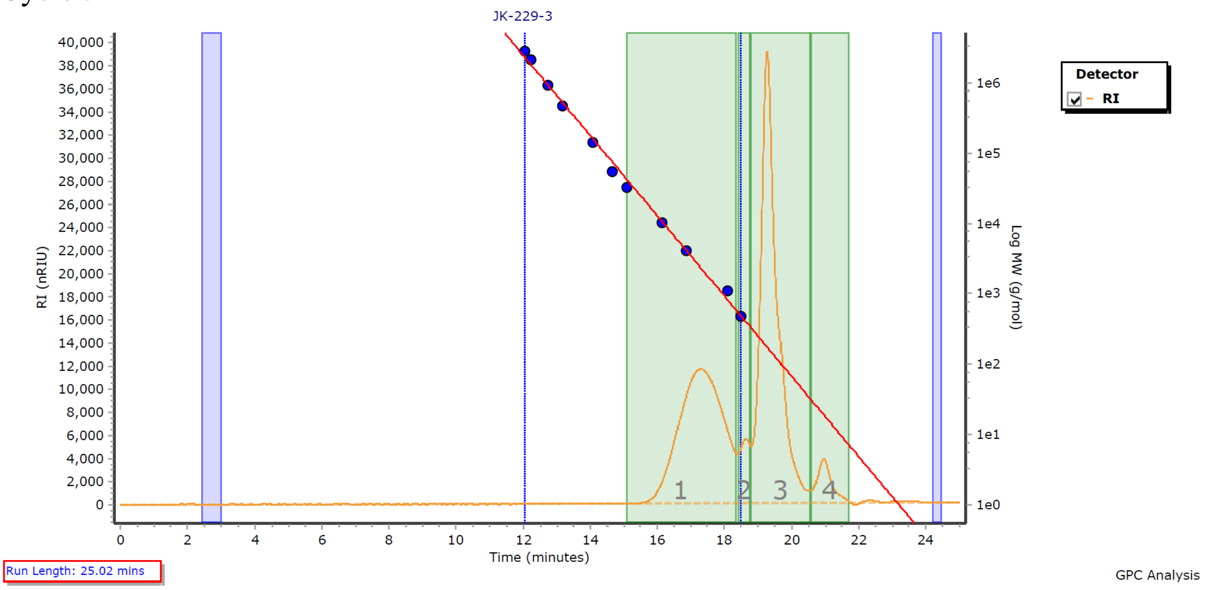

Molecular Weight Averages

Peak	Mp (g/mol)	Mn (g/mol)	Mw (g/mol)	Mz (g/mol)	Mz+1 (g/mol)	Mv (g/mol)	PD
Peak 1	2293	1802	3029	5029	7481	2800	1.681
Peak 2	395	410	417	424	432	416	1.017
Peak 3	170	132	156	176	193	153	1.182
Peak 4	19	17	18	19	21	18	1.059

Cycle 4

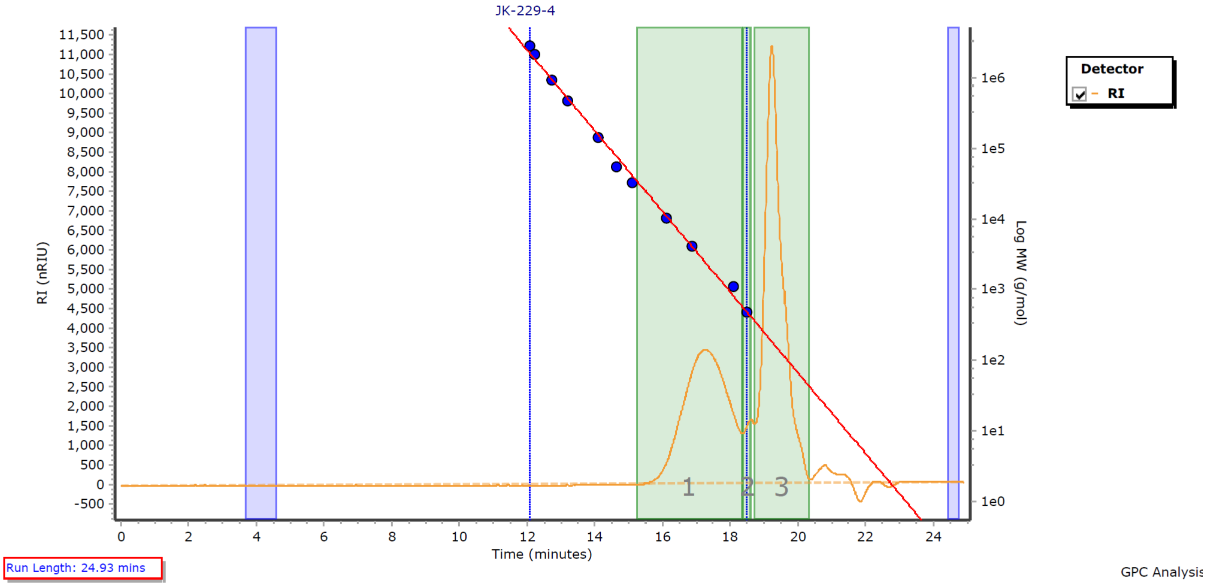

Molecular Weight Averages

Peak	Mp (g/mol)	Mn (g/mol)	Mw (g/mol)	Mz (g/mol)	Mz+1 (g/mol)	Mv (g/mol)	PD
Peak 1	2381	1830	3107	5197	7802	2868	1.698
Peak 2	406	452	456	460	465	455	1.009
Peak 3	177	144	168	190	210	164	1.167

Cycle 5

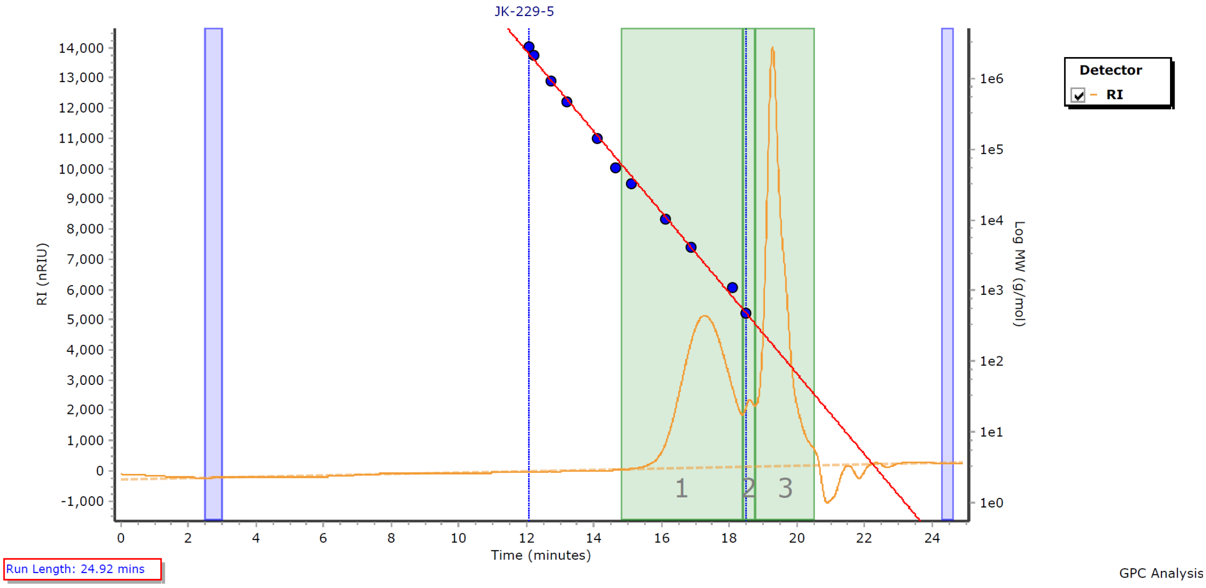

Molecular Weight Averages

Peak	Mp (g/mol)	Mn (g/mol)	Mw (g/mol)	Mz (g/mol)	Mz+1 (g/mol)	Mv (g/mol)	PD
Peak 1	2359	1820	3270	6293	11850	2977	1.797
Peak 2	406	407	414	421	427	413	1.017
Peak 3	169	126	151	173	192	148	1.198

Cycle 6

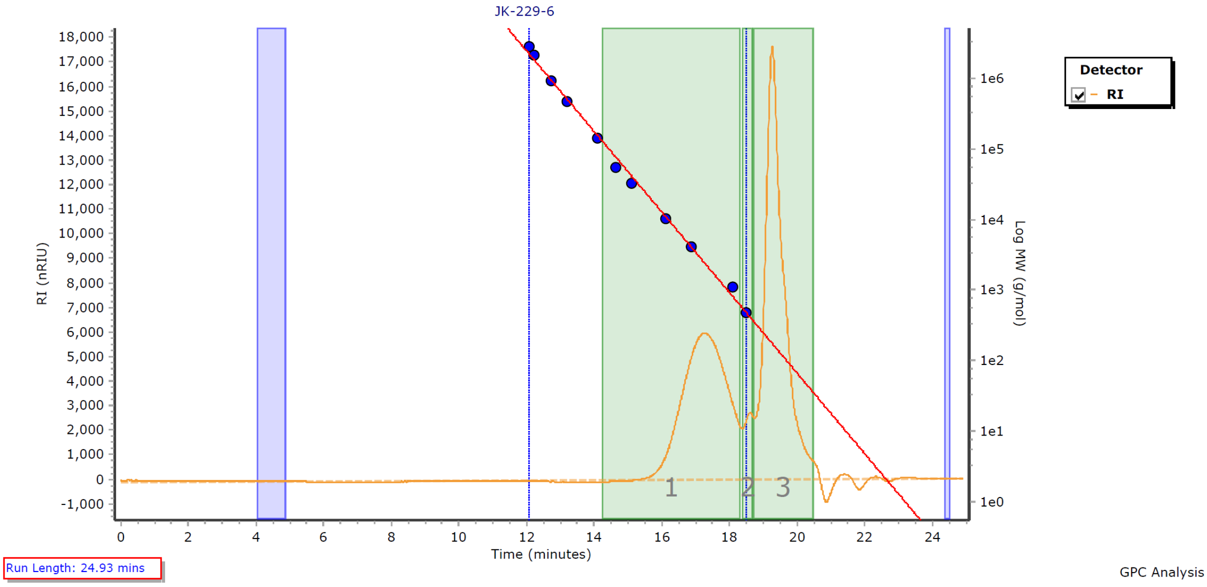

Molecular Weight Averages

Peak	Mp (g/mol)	Mn (g/mol)	Mw (g/mol)	Mz (g/mol)	Mz+1 (g/mol)	Mv (g/mol)	PD
Peak 1	2359	1866	3195	5565	8918	2939	1.712
Peak 2	406	433	439	446	452	439	1.014
Peak 3	170	130	157	181	202	154	1.208

Cycle 7

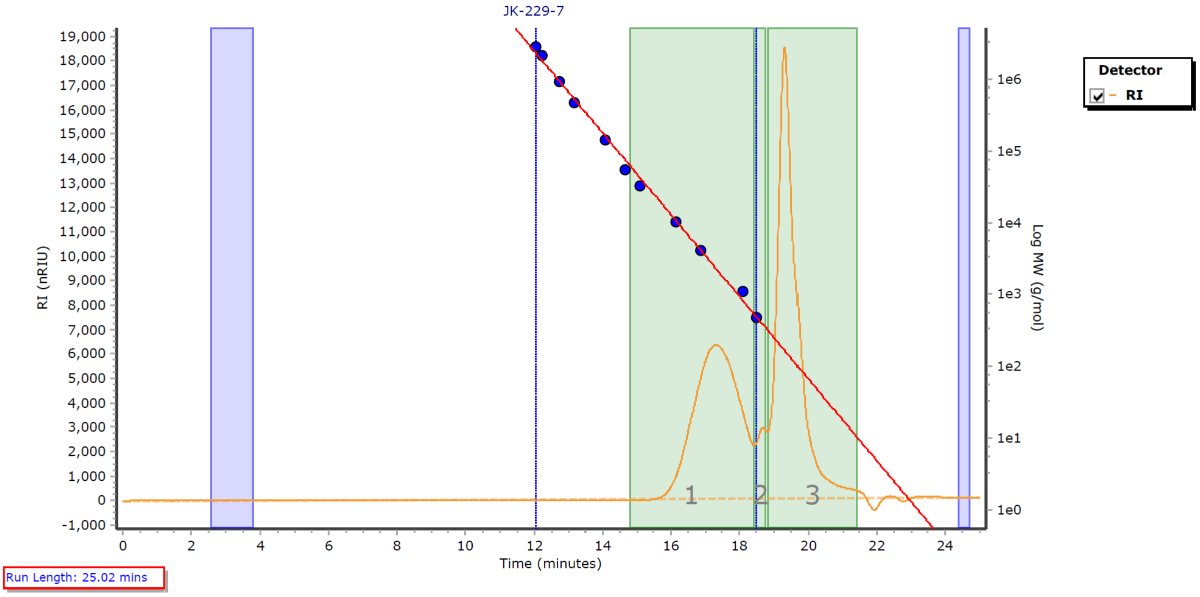

Molecular Weight Averages

Peak	Mp (g/mol)	Mn (g/mol)	Mw (g/mol)	Mz (g/mol)	Mz+1 (g/mol)	Mv (g/mol)	PD
Peak 1	2229	1720	2938	4931	7388	2710	1.708
Peak 2	377	388	393	399	404	392	1.013
Peak 3	162	103	144	168	187	140	1.398

Cycle 8

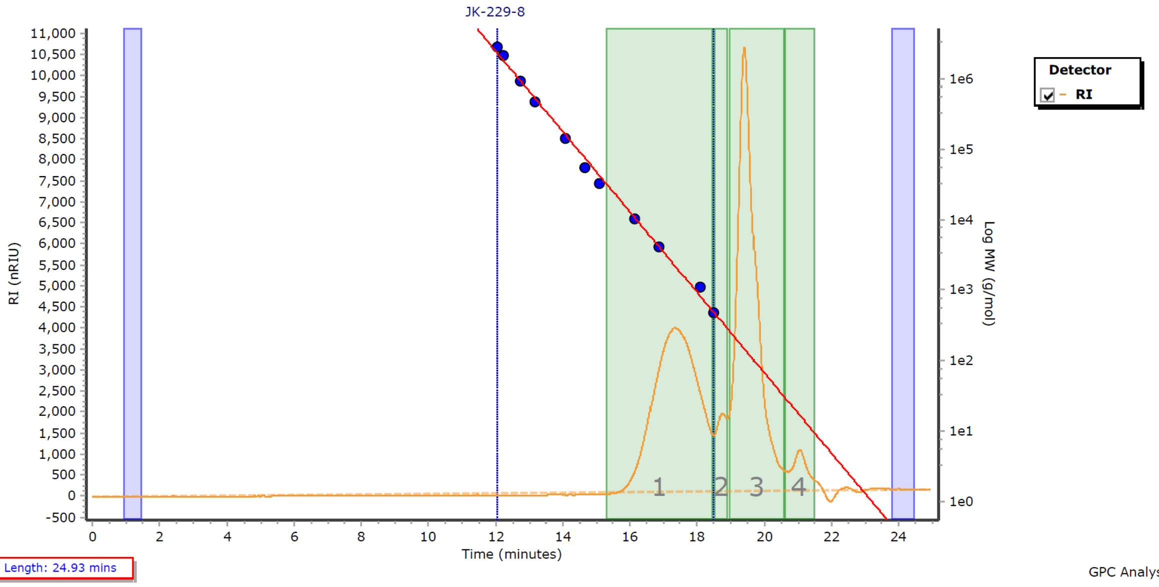

Run Length: 24.93 mins

GPC Analysis

Molecular Weight Averages

Peak	Mp (g/mol)	Mn (g/mol)	Mw (g/mol)	Mz (g/mol)	Mz+1 (g/mol)	Mv (g/mol)	PD
Peak 1	2167	1613	2754	4574	6727	2543	1.707
Peak 2	340	342	350	358	366	349	1.023
Peak 3	142	109	130	147	162	127	1.193
Peak 4	16	16	17	19	20	17	1.063

Cycle 9

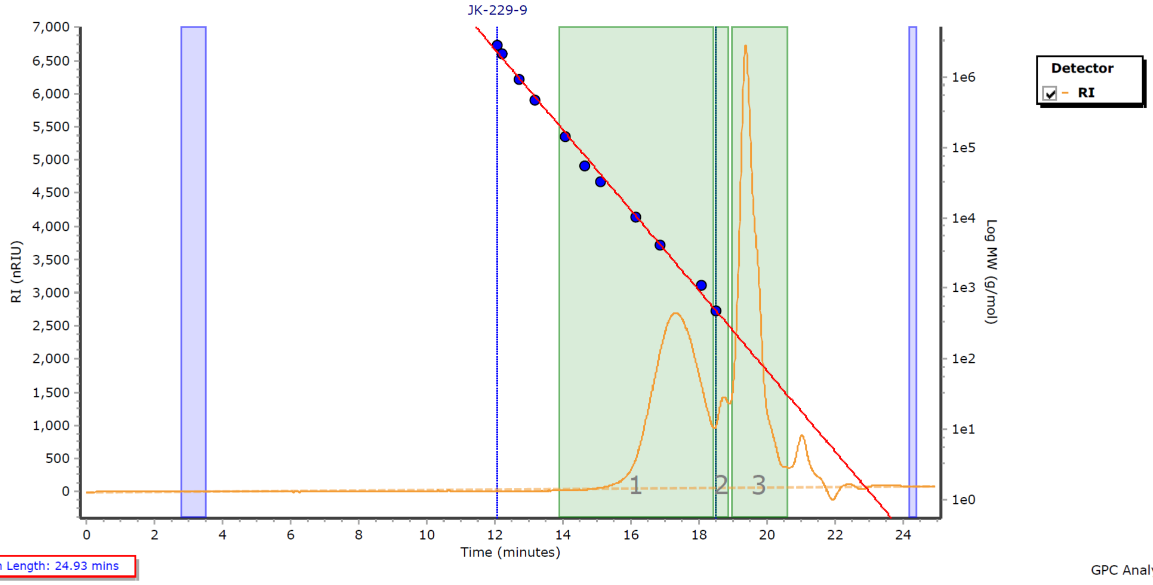

Run Length: 24.93 mins

GPC Analysis

Molecular Weight Averages

Peak	Mp (g/mol)	Mn (g/mol)	Mw (g/mol)	Mz (g/mol)	Mz+1 (g/mol)	Mv (g/mol)	PD
Peak 1	2208	1722	3190	6578	13137	2882	1.852
Peak 2	349	359	366	373	380	365	1.019
Peak 3	149	113	134	152	167	132	1.186

Cycle 10

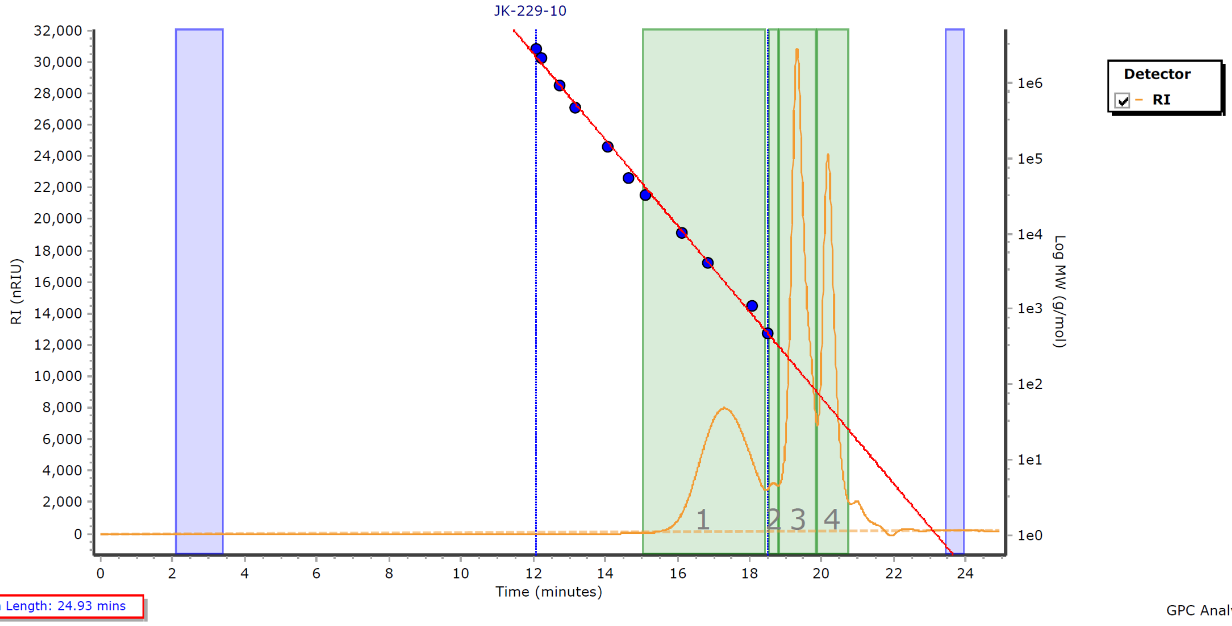

Run Length: 24.93 mins

GPC Analysis

Molecular Weight Averages

Peak	Mp (g/mol)	Mn (g/mol)	Mw (g/mol)	Mz (g/mol)	Mz+1 (g/mol)	Mv (g/mol)	PD
Peak 1	2250	1708	2978	5101	7806	2739	1.744
Peak 2	384	372	377	382	387	376	1.013
Peak 3	161	143	155	168	182	153	1.084
Peak 4	52	48	50	53	55	50	1.042

^{1}H and ^{13}C NMR data for the crude reaction mixture obtained from the depolymerisation of PU1 during catalyst recycling study

Cycle 1

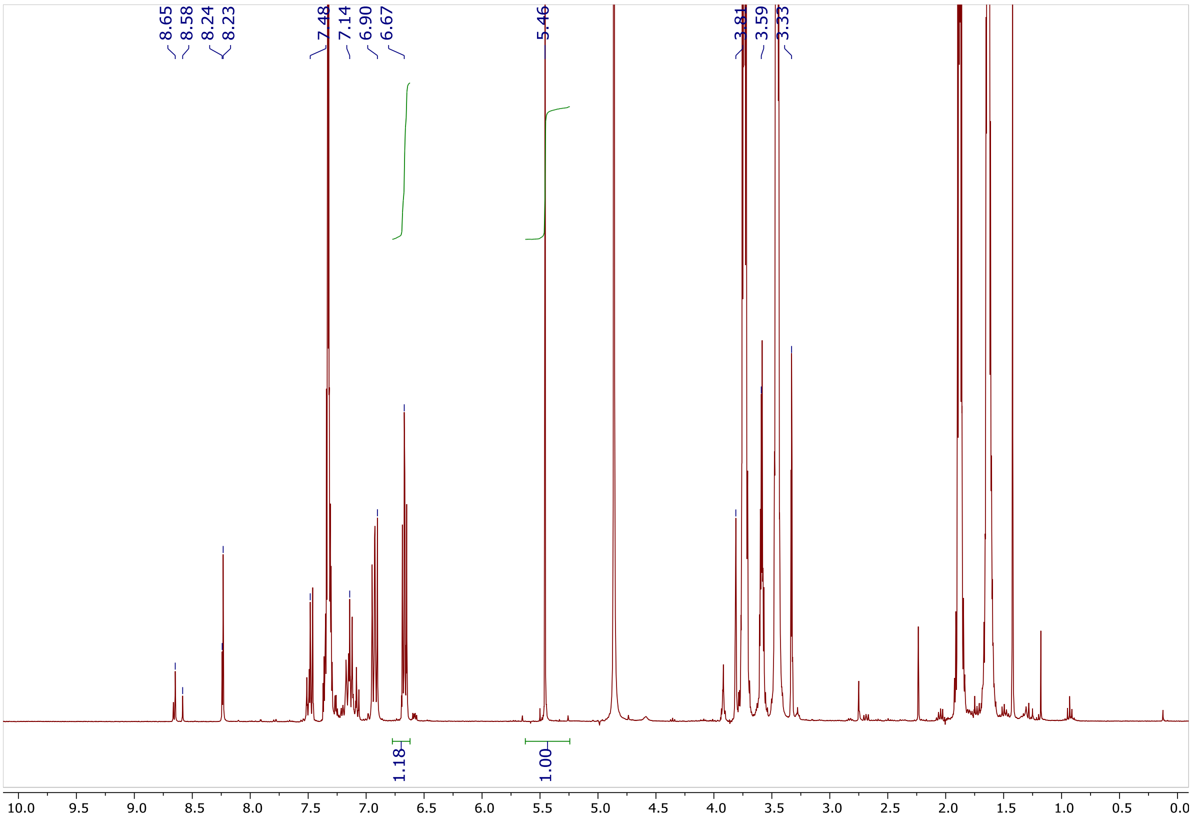

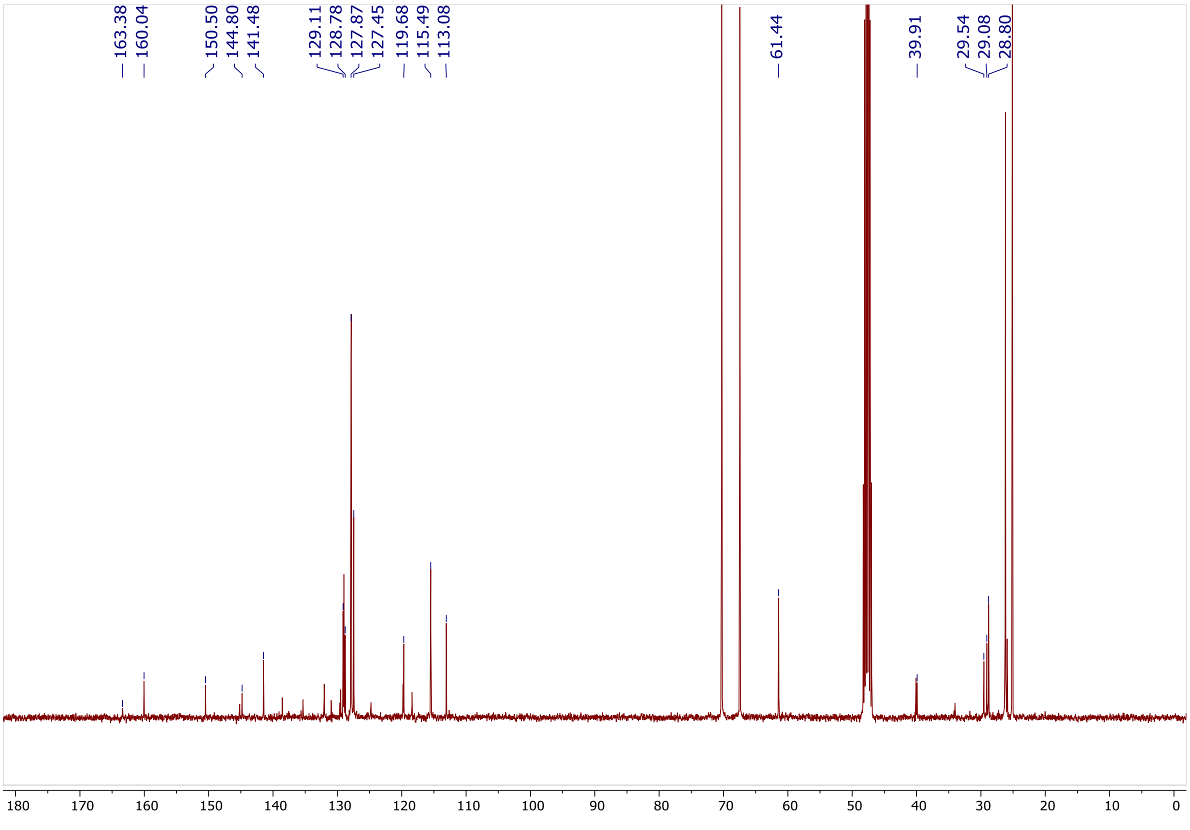

Cycle 2

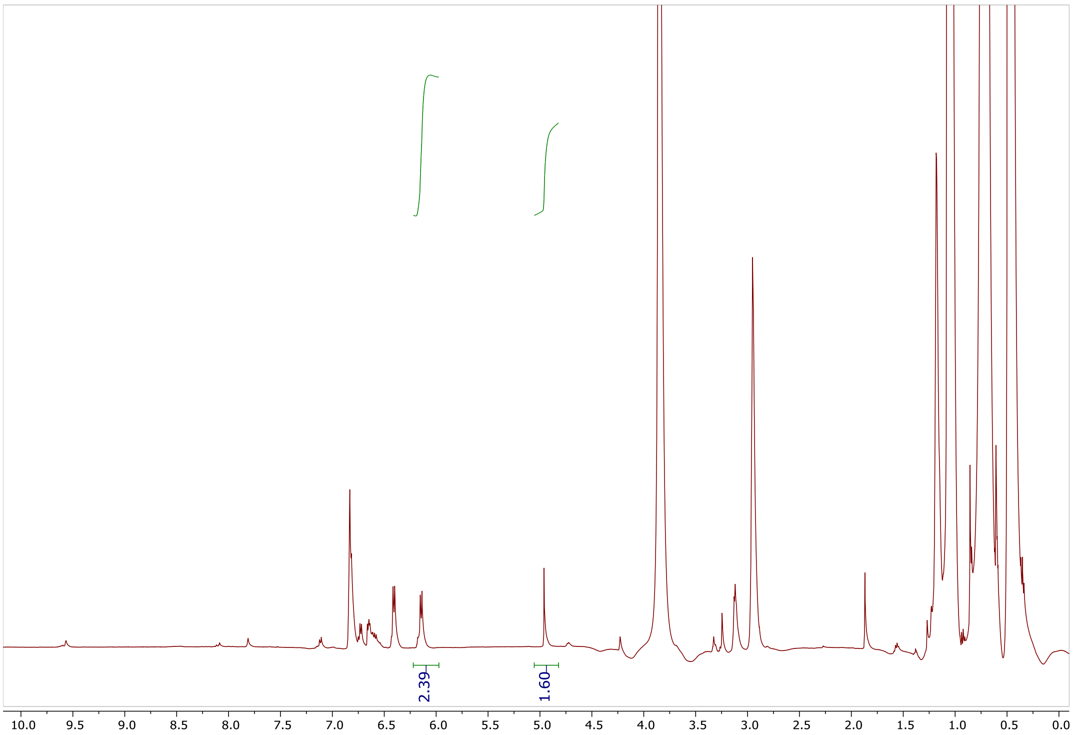
2.39
1.60
10.0 9.5 9.0 8.5 8.0 7.5 7.0 6.5 6.0 5.5 5.0 4.5 4.0 3.5 3.0 2.5 2.0 1.5 1.0 0.5 0.0

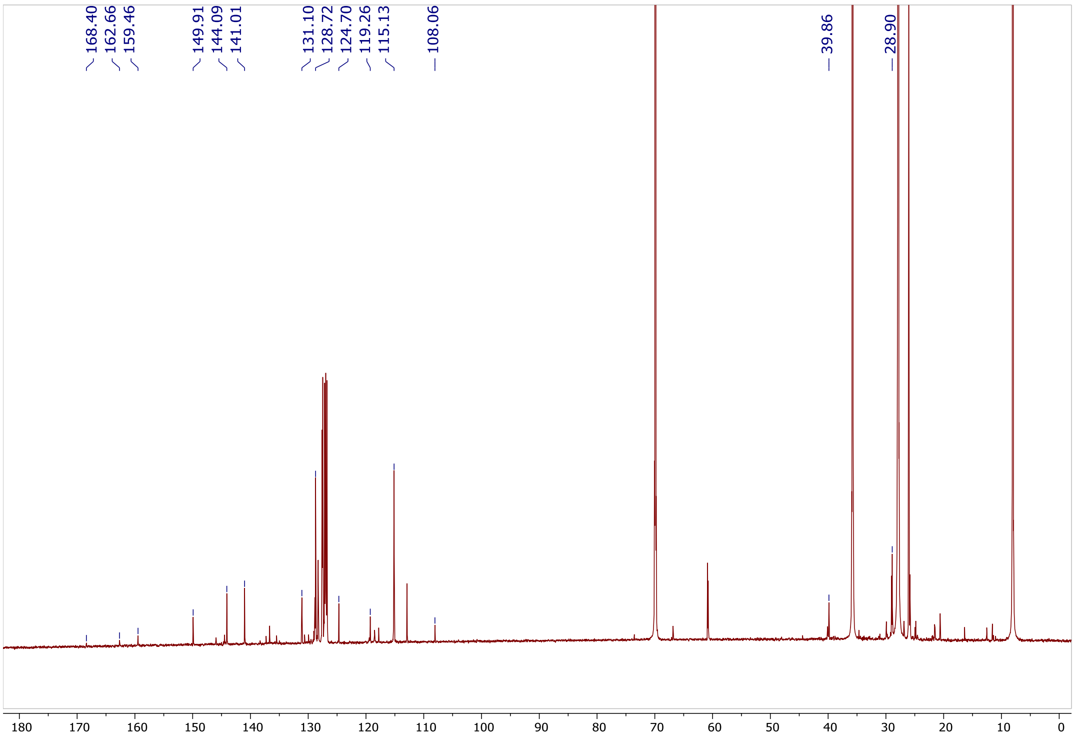
168.40
162.66
159.46
149.91
144.09
141.01
131.10
128.72
124.70
119.26
115.13
108.06
39.86
28.90
180 170 160 150 140 130 120 110 100 90 80 70 60 50 40 30 20 10 0

Cycle 3

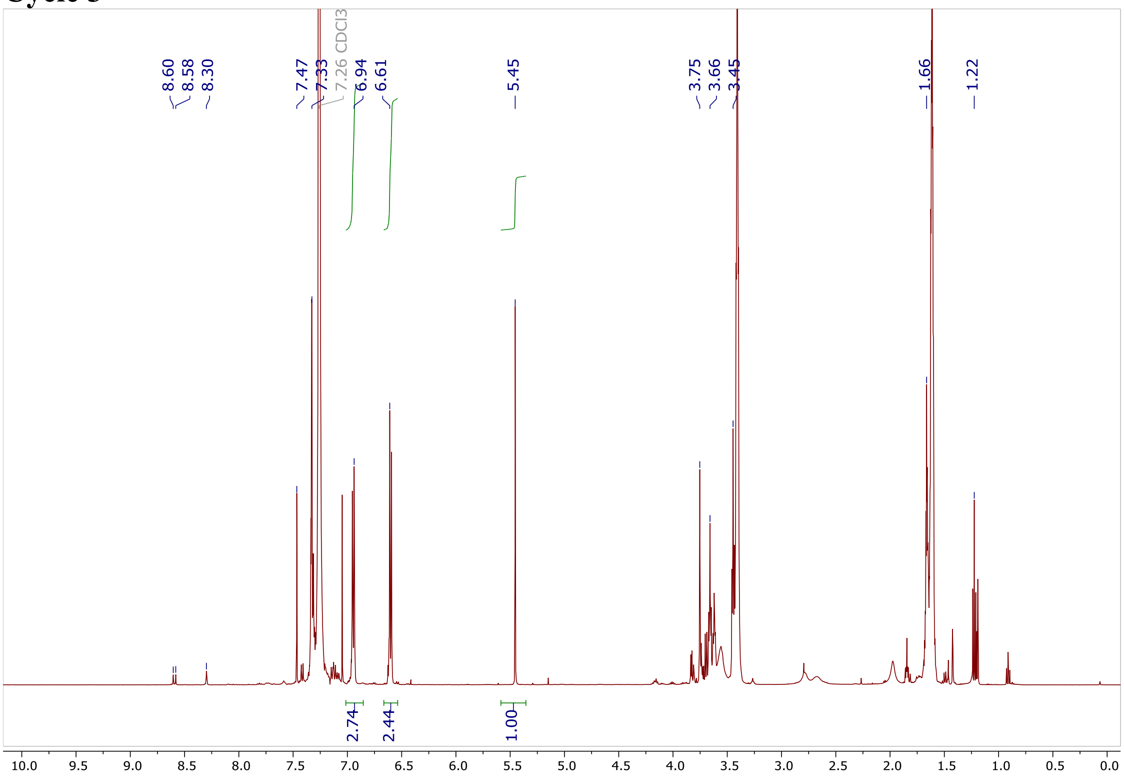

8.60
8.58
8.30
7.47
7.33
7.26 CDCl3
6.94
6.61
5.45
3.75
3.66
3.45
1.66
1.22
2.74
2.44
1.00
10.0 9.5 9.0 8.5 8.0 7.5 7.0 6.5 6.0 5.5 5.0 4.5 4.0 3.5 3.0 2.5 2.0 1.5 1.0 0.5 0.0

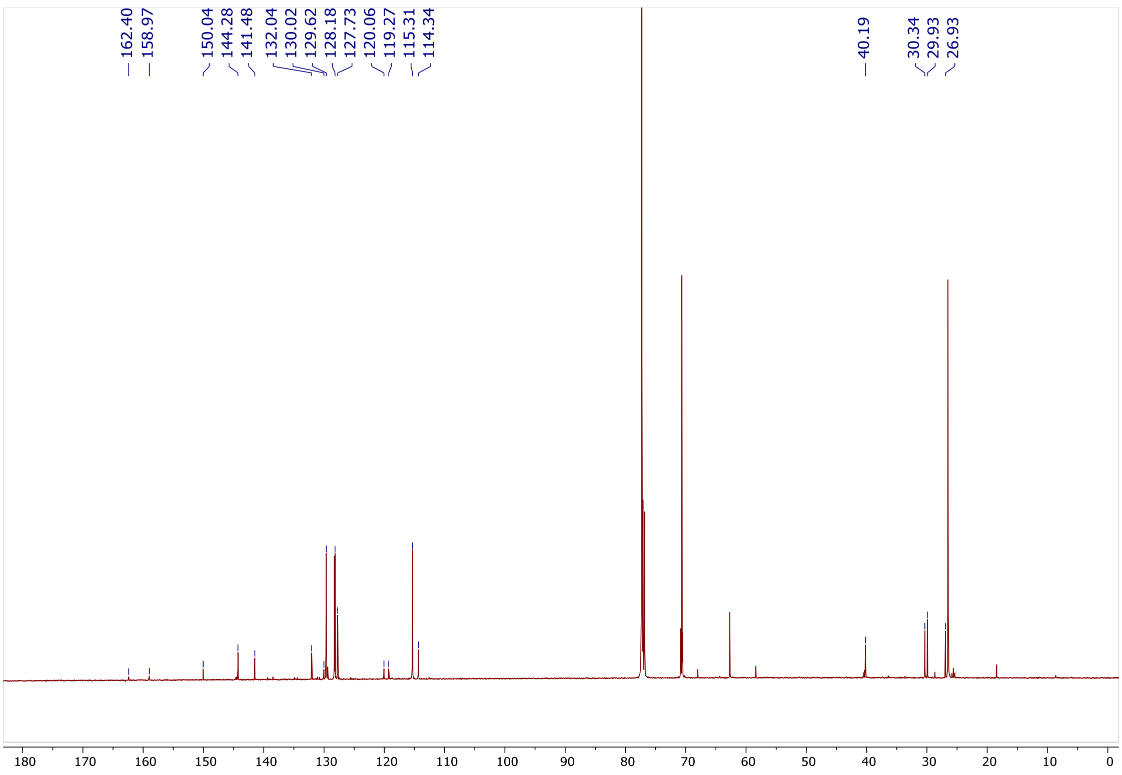

162.40
158.97
150.04
144.28
141.48
132.04
130.02
129.62
128.18
127.73
120.06
119.27
115.31
114.34
40.19
30.34
29.93
26.93
180 170 160 150 140 130 120 110 100 90 80 70 60 50 40 30 20 10 0

Cycle 4

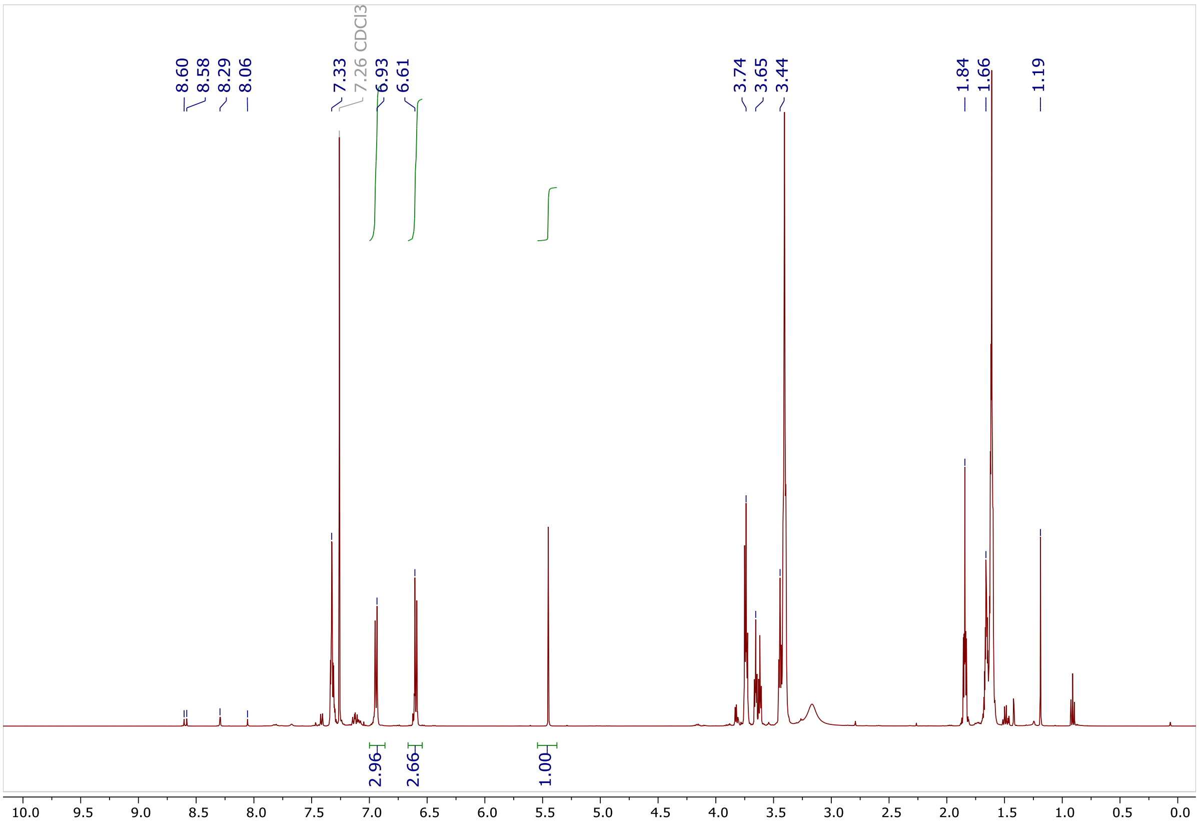
8.60
8.58
8.29
8.06
7.33
7.26 CDCl3
6.93
6.61
3.74
3.65
3.44
1.84
1.66
1.19
2.96
2.66
1.00
10.0 9.5 9.0 8.5 8.0 7.5 7.0 6.5 6.0 5.5 5.0 4.5 4.0 3.5 3.0 2.5 2.0 1.5 1.0 0.5 0.0

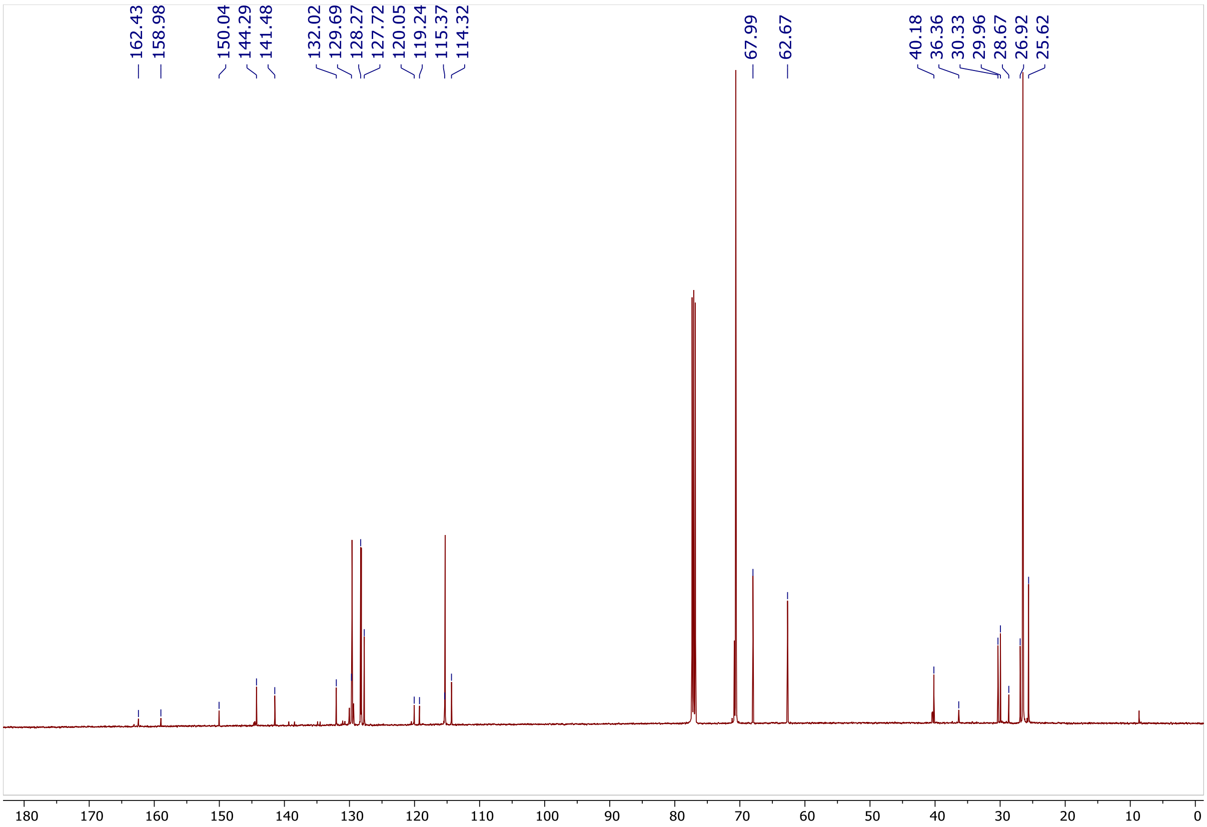
162.43
158.98
150.04
144.29
141.48
132.02
129.69
128.27
127.72
120.05
119.24
115.37
114.32
67.99
62.67
40.18
36.36
30.33
29.96
28.67
26.92
25.62
180 170 160 150 140 130 120 110 100 90 80 70 60 50 40 30 20 10 0

Cycle 5

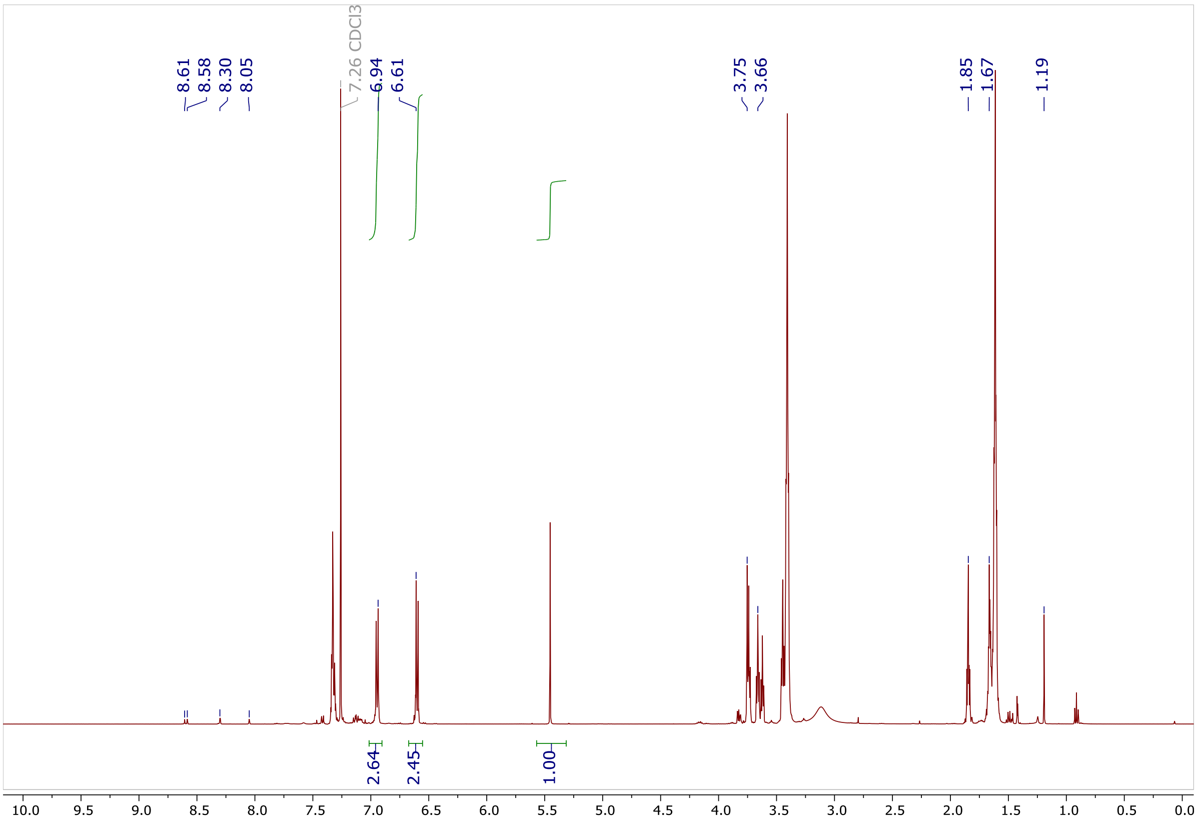
8.61
8.58
8.30
8.05
7.26 CDCl3
6.94
6.61
3.75
3.66
1.85
1.67
1.19
2.64
2.45
1.00
10.0 9.5 9.0 8.5 8.0 7.5 7.0 6.5 6.0 5.5 5.0 4.5 4.0 3.5 3.0 2.5 2.0 1.5 1.0 0.5 0.0

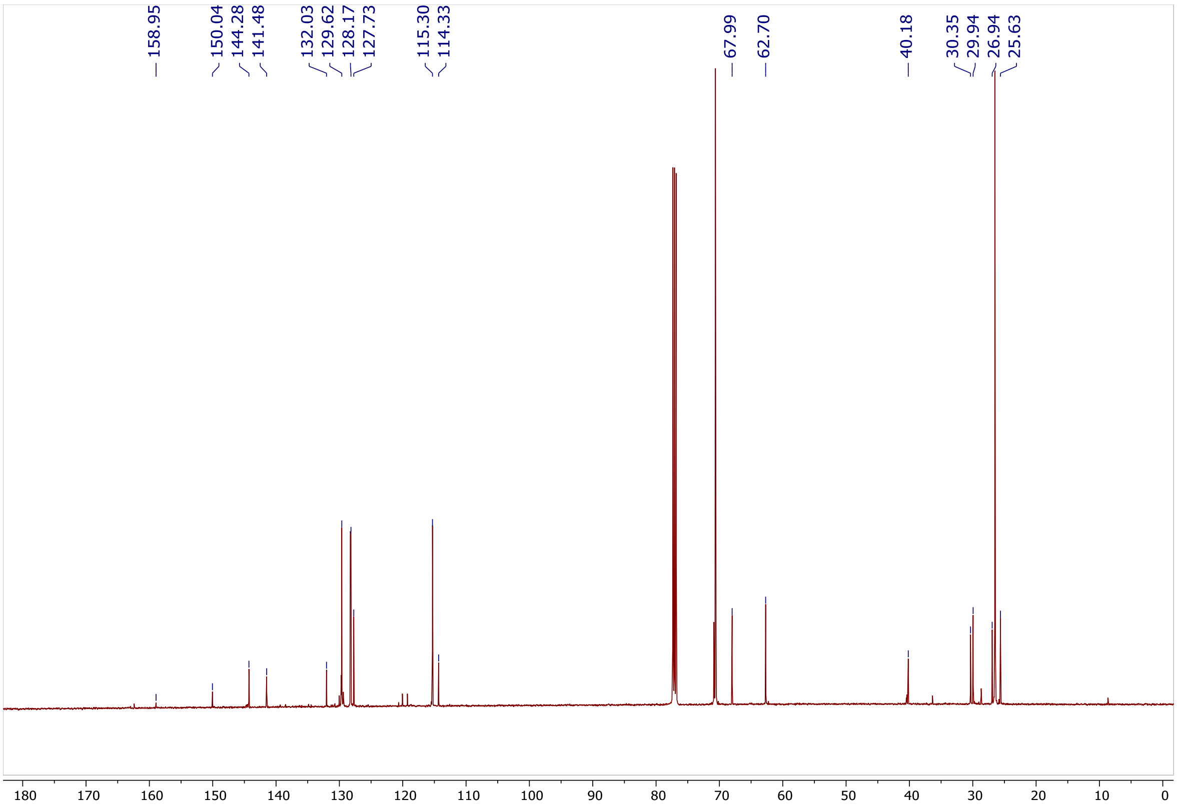
158.95
150.04
144.28
141.48
132.03
129.62
128.17
127.73
115.30
114.33
67.99
62.70
40.18
30.35
29.94
26.94
25.63
180 170 160 150 140 130 120 110 100 90 80 70 60 50 40 30 20 10 0

Cycle 6

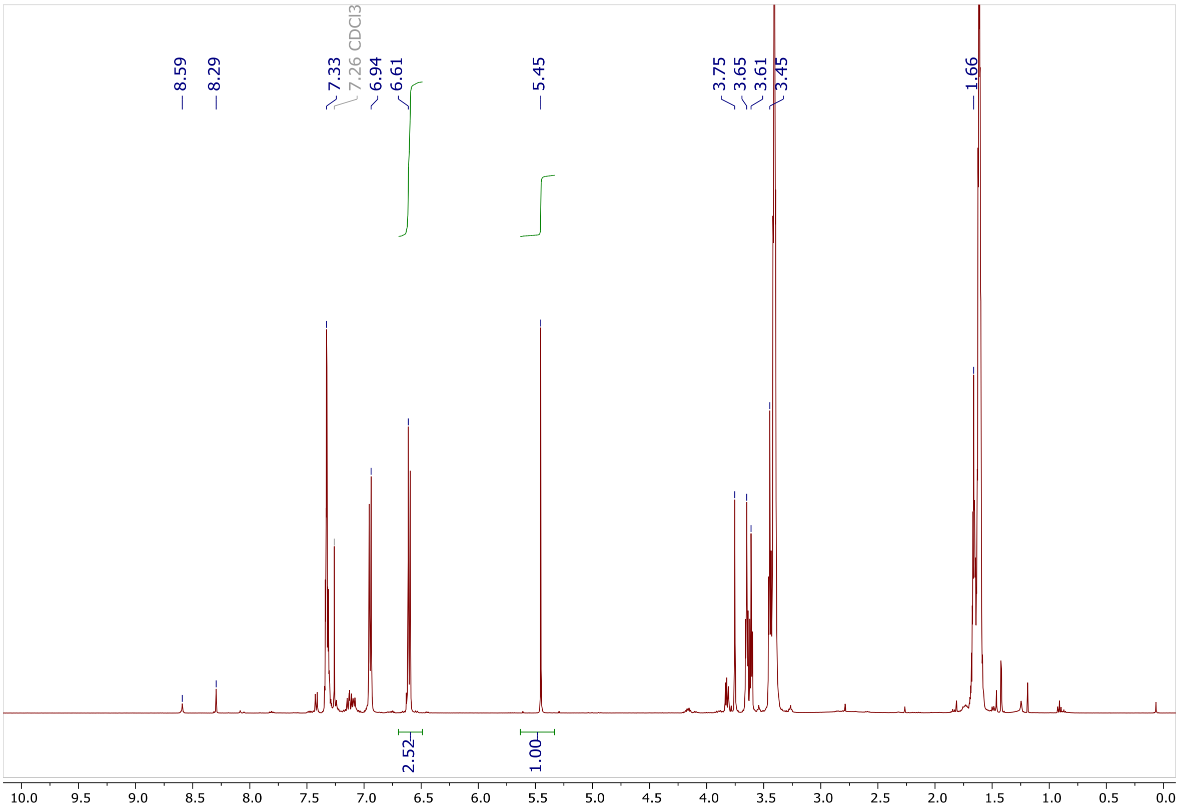

8.59
8.29
7.33
7.26 CDCl3
6.94
6.61
5.45
3.75
3.65
3.61
3.45
1.66
2.52
1.00
10.0 9.5 9.0 8.5 8.0 7.5 7.0 6.5 6.0 5.5 5.0 4.5 4.0 3.5 3.0 2.5 2.0 1.5 1.0 0.5 0.0

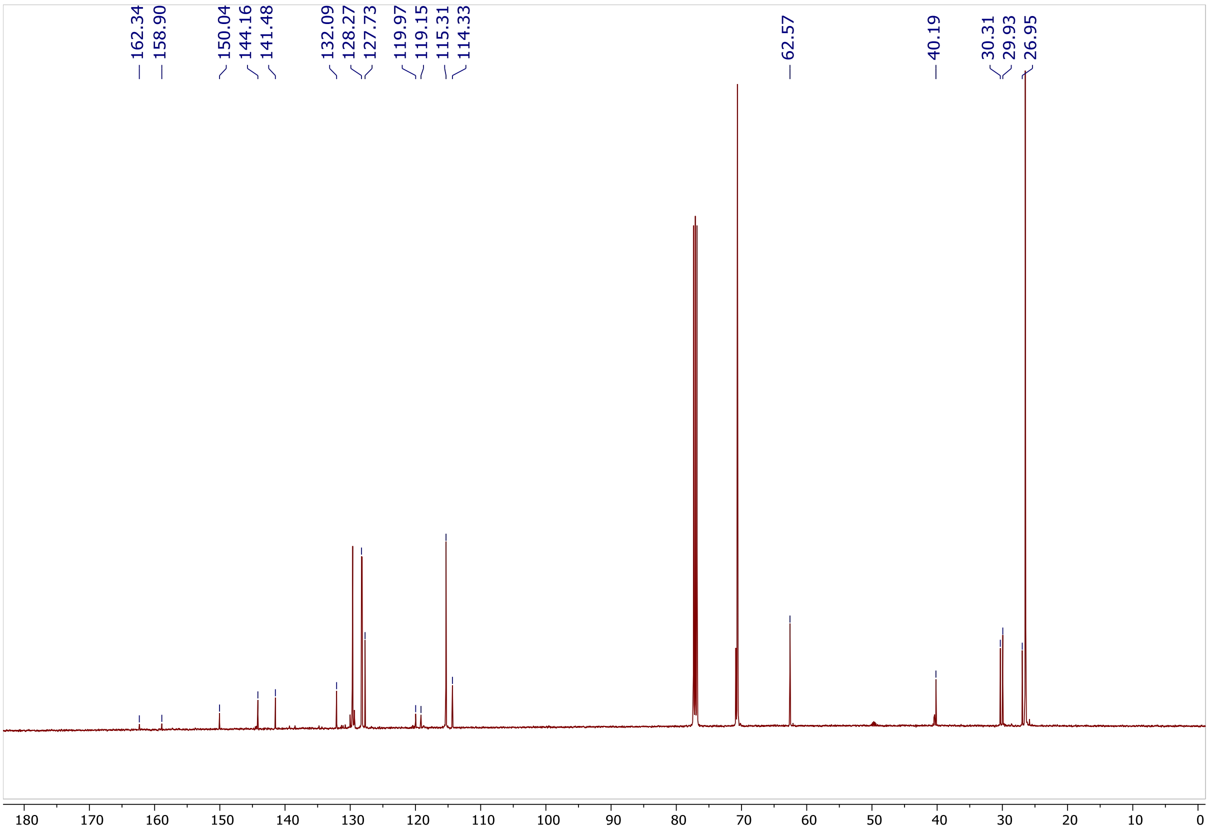

162.34
158.90
150.04
144.16
141.48
132.09
128.27
127.73
119.97
119.15
115.31
114.33
62.57
40.19
30.31
29.93
26.95
180 170 160 150 140 130 120 110 100 90 80 70 60 50 40 30 20 10 0

Cycle 7

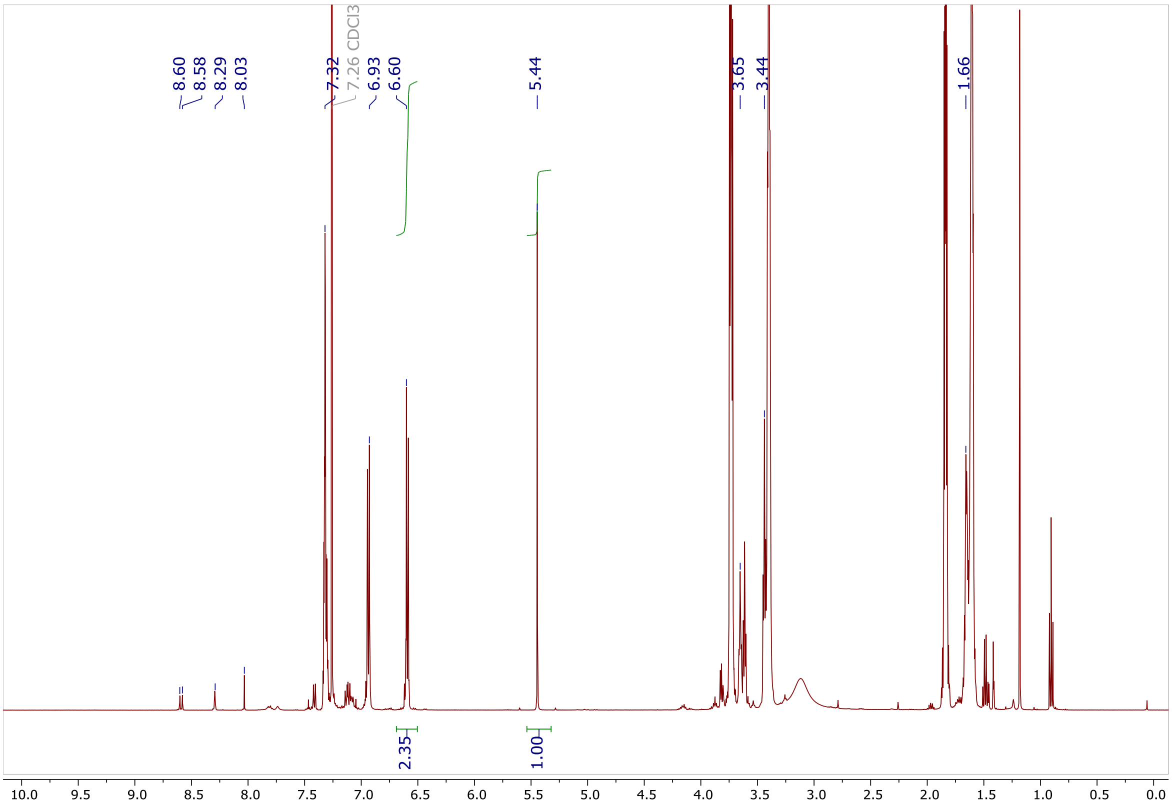
8.60
8.58
8.29
8.03
7.32
7.26 CDCl3
6.93
6.60
5.44
3.65
3.44
1.66
2.35
1.00
10.0 9.5 9.0 8.5 8.0 7.5 7.0 6.5 6.0 5.5 5.0 4.5 4.0 3.5 3.0 2.5 2.0 1.5 1.0 0.5 0.0

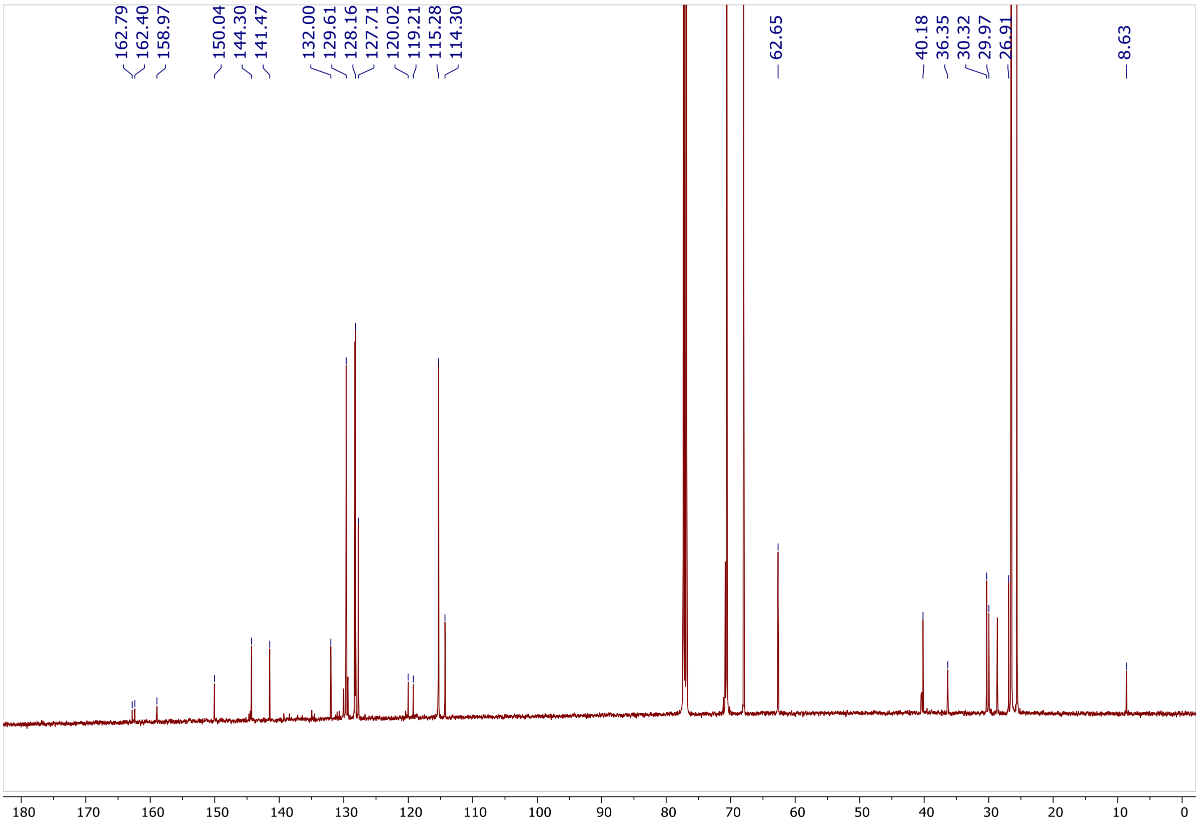
162.79
162.40
158.97
150.04
144.30
141.47
132.00
129.61
128.16
127.71
120.02
119.21
115.28
114.30
62.65
40.18
36.35
30.32
29.97
26.91
8.63
180 170 160 150 140 130 120 110 100 90 80 70 60 50 40 30 20 10 0

Cycle 8

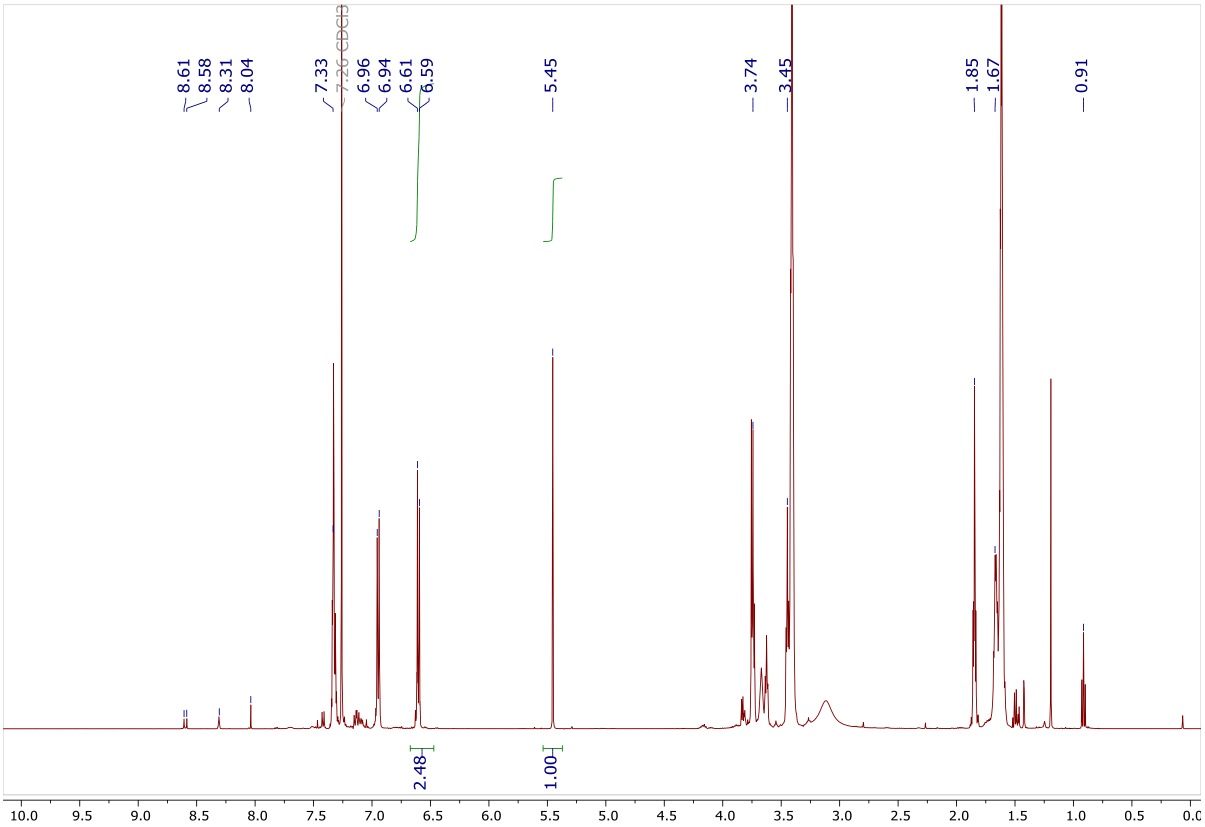
8.61
8.58
8.31
8.04
7.33
7.26 CDCl3
6.96
6.94
6.61
6.59
5.45
3.74
3.45
1.85
1.67
0.91
2.48
1.00
10.0
9.5
9.0
8.5
8.0
7.5
7.0
6.5
6.0
5.5
5.0
4.5
4.0
3.5
3.0
2.5
2.0
1.5
1.0
0.5

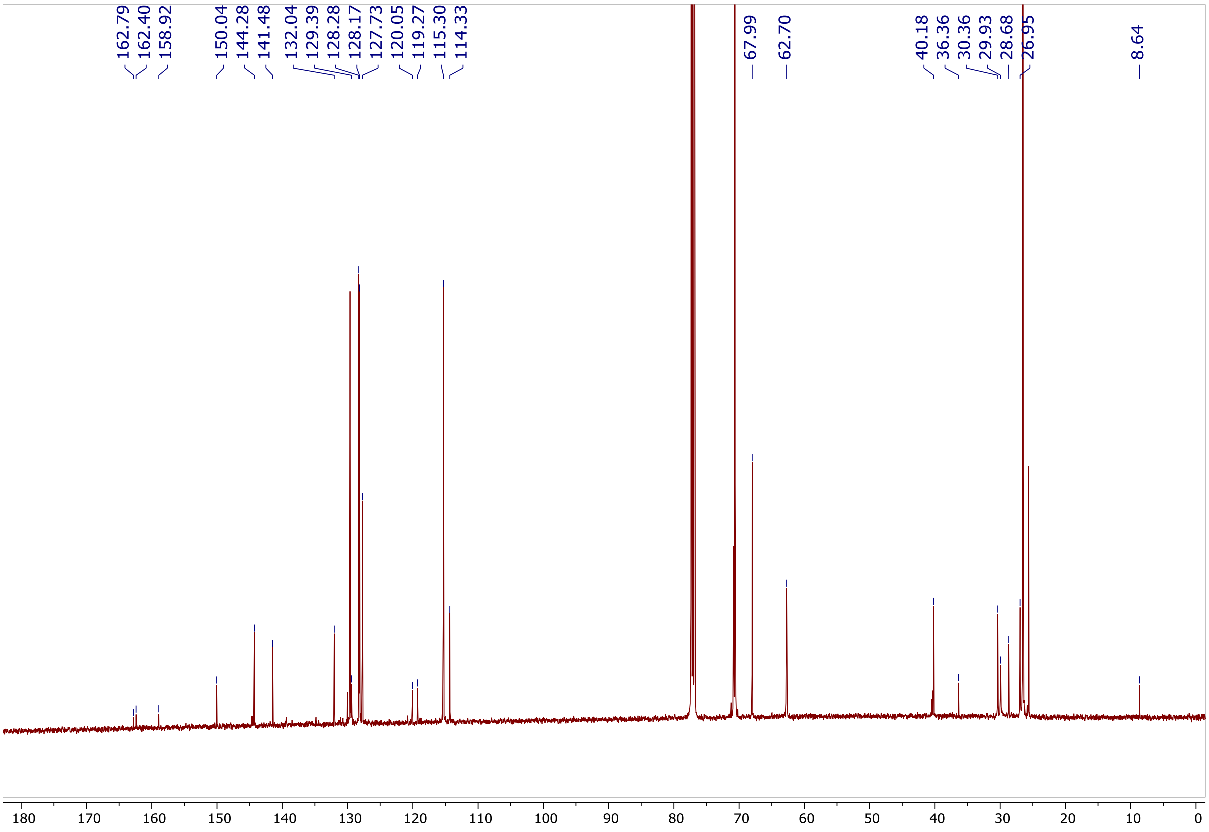
162.79
162.40
158.92
150.04
144.28
141.48
132.04
129.39
128.28
128.17
127.73
120.05
119.27
115.30
114.33
67.99
62.70
40.18
36.36
30.36
29.93
28.68
26.95
8.64
180
170
160
150
140
130
120
110
100
90
80
70
60
50
40
30
20
10
0

Cycle 9

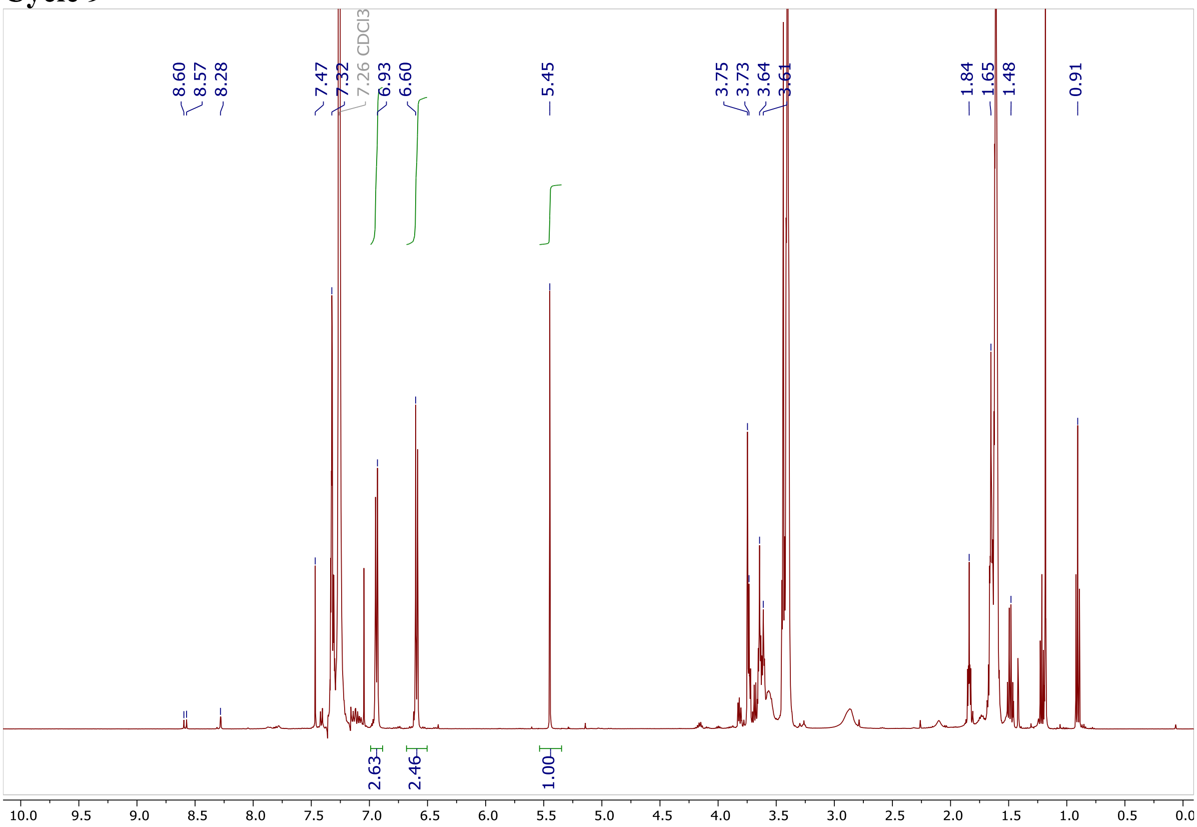
8.60
8.57
8.28
7.47
7.32
7.26 CDCl3
6.93
6.60
5.45
3.75
3.73
3.64
3.61
1.84
1.65
1.48
0.91
2.63
2.46
1.00
10.0 9.5 9.0 8.5 8.0 7.5 7.0 6.5 6.0 5.5 5.0 4.5 4.0 3.5 3.0 2.5 2.0 1.5 1.0 0.5 0.0

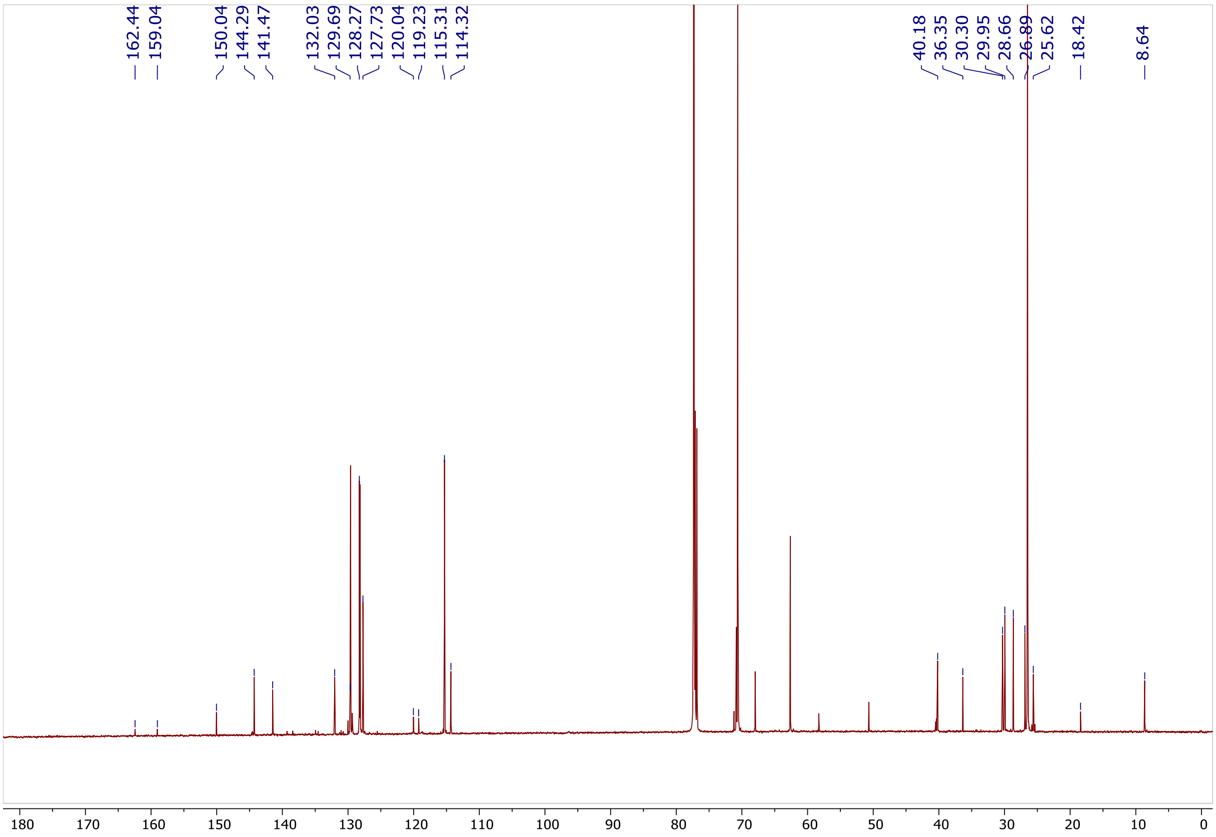
162.44
159.04
150.04
144.29
141.47
132.03
129.69
128.27
127.73
120.04
119.23
115.31
114.32
40.18
36.35
30.30
29.95
28.66
26.89
25.62
18.42
8.64
180 170 160 150 140 130 120 110 100 90 80 70 60 50 40 30 20 10 0

Cycle 10

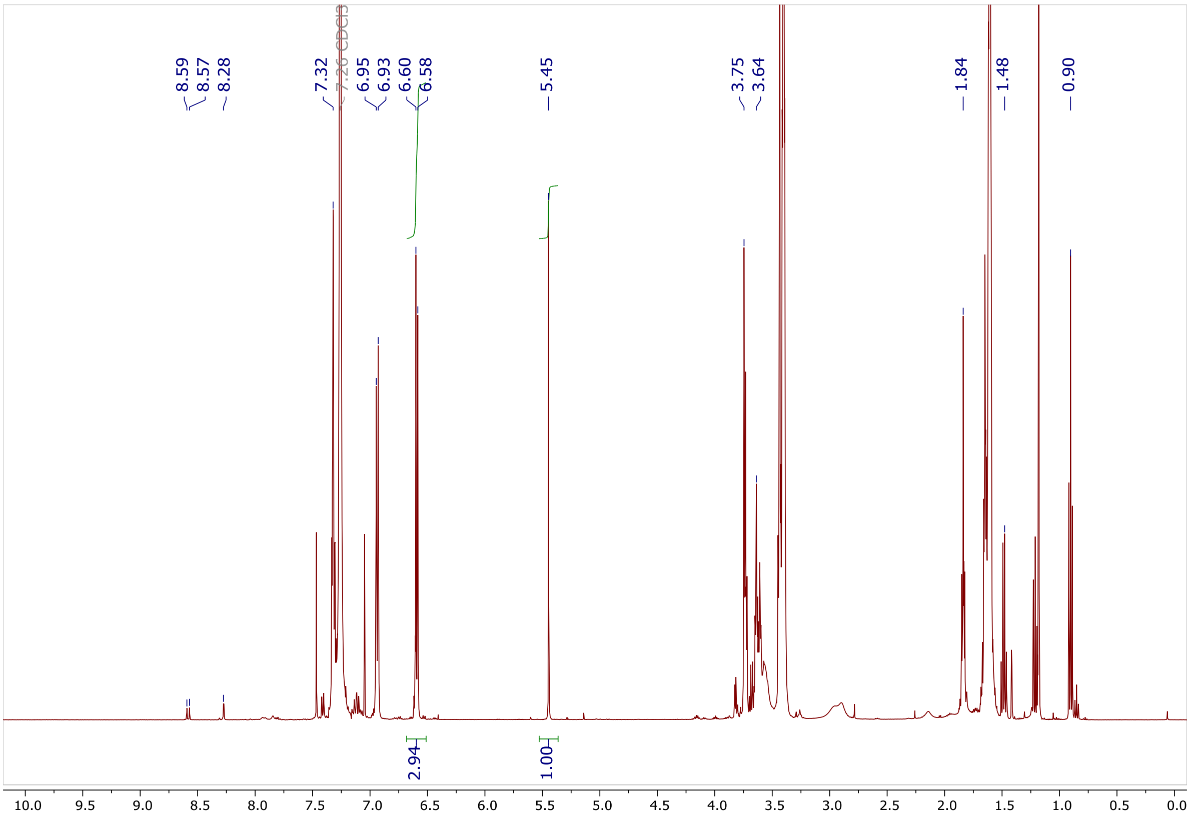
8.59
8.57
8.28
7.32
7.26 CDCl3
6.95
6.93
6.60
6.58
5.45
3.75
3.64
1.84
1.48
0.90
2.94
1.00
10.0 9.5 9.0 8.5 8.0 7.5 7.0 6.5 6.0 5.5 5.0 4.5 4.0 3.5 3.0 2.5 2.0 1.5 1.0 0.5 0.0

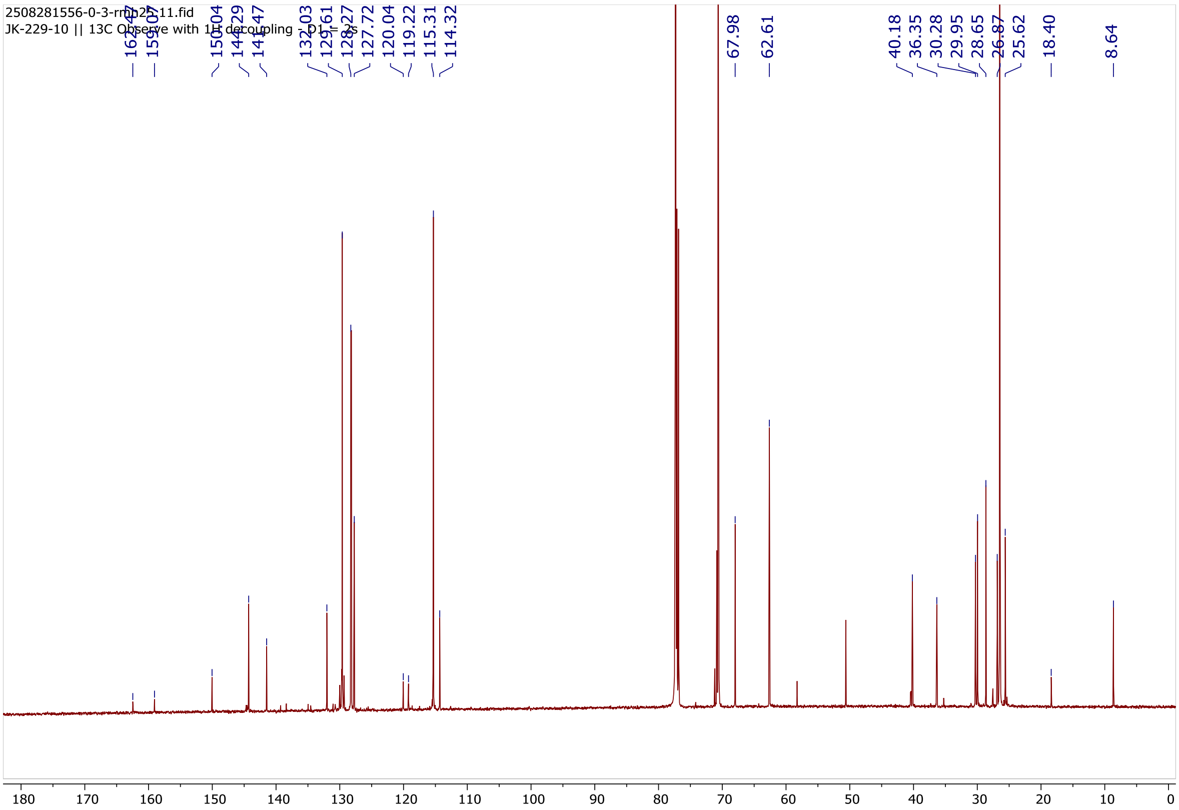
2508281556-0-3-rmb25_11.fid
JK-229-10 || 13C Observe with 1H decoupling - D1 = 2s
150.04
132.03
129.61
128.27
127.72
120.04
119.22
115.31
114.32
67.98
62.61
40.18
36.35
30.28
29.95
28.65
26.87
25.62
18.40
8.64
180 170 160 150 140 130 120 110 100 90 80 70 60 50 40 30 20 10 0

10. References:

(1) Lee, H.; Pootheri, N.; Kim, C. W.; Lee, S. A One-Step Strategy for Synthesizing N-Formanilide via Benzamide Activation and Oxalic Acid-Driven Reduction. *Org. Biomol. Chem.* **2025**, *23* (31), 7254–7259. https://doi.org/10.1039/D5OB00941C.

(2) Vlaminck, L.; Van de Voorde, B.; Du Prez, F. E. Sustainable Synthesis Routes towards Urazole Compounds. *Green Chem.* **2017**, *19* (23), 5659–5664. https://doi.org/10.1039/C7GC02027A.

(3) Liu, H.; Mei, Q.; Xu, Q.; Song, J.; Liu, H.; Han, B. Synthesis of Formamides Containing Unsaturated Groups by: N -Formylation of Amines Using CO2 with H2. *Green Chem.* **2017**, *19* (1), 196–201. https://doi.org/10.1039/c6gc02243j.

(4) Owen, A. E.; Preiss, A.; McLuskie, A.; Gao, C.; Peters, G.; Bühl, M.; Kumar, A. Manganese-Catalyzed Dehydrogenative Synthesis of Urea Derivatives and Polyureas. *ACS Catal.* **2022**, *12* (12), 6923–6933. https://doi.org/10.1021/acscatal.2c00850.

(5) Mane, M.; Balaskar, R.; Gavade, S.; Pabrekar, P.; Mane, D. An Efficient and Greener Protocol towards Synthesis of Unsymmetrical N,N′-Biphenyl Urea. *Arab. J. Chem.* **2013**, *6* (4), 423–427. https://doi.org/https://doi.org/10.1016/j.arabjc.2011.01.030.

(6) Ikawa, T.; Barder, T. E.; Biscoe, M. R.; Buchwald, S. L. Pd-Catalyzed Amidations of Aryl Chlorides Using Monodentate Biaryl Phosphine Ligands: A Kinetic, Computational, and Synthetic Investigation. *J. Am. Chem. Soc.* **2007**, *129* (43), 13001–13007. https://doi.org/10.1021/ja0717414.

(7) Futter, J.; Holzer, M.; Rieger, B. Formic Acid as Feedstock in the Phosgene-Free Dehydrogenative Coupling of Formamides and Alcohols to Polyurethanes. *Macromolecules* **2025**, *58* (4), 1817–1826. https://doi.org/10.1021/acs.macromol.4c01559.

(8) Zhang, L.; Han, Z.; Zhao, X.; Wang, Z.; Ding, K. Highly Efficient Ruthenium-Catalyzed N-Formylation of Amines with H2 and CO2. *Angew. Chem. Int. Ed.* **2015**, *54* (21), 6186–6189. https://doi.org/10.1002/anie.201500939.

(9) Das, V. K.; Devi, R. R.; Raul, P. K.; Thakur, A. J. Nano Rod-Shaped and Reusable Basic Al2O3 Catalyst for N-Formylation of Amines under Solvent-Free Conditions: A Novel, Practical and Convenient 'NOSE' Approach. *Green Chem.* **2012**, *14* (3), 847–854. https://doi.org/10.1039/C2GC16020J.

(10) Iwasaki, T.; Saito, N.; Yamada, Y.; Ajiro, S.; Nozaki, K. Hydrogenolysis of Urethanes and Ureas Catalyzed by Manganese Complex Supported by Bidentate PN Ligand. *Organometallics* **2024**, *43* (9), 924–928. https://doi.org/10.1021/acs.organomet.4c00032.

(11) Gausas, L.; Kristensen, S. K.; Sun, H.; Ahrens, A.; Donslund, B. S.; Lindhardt, A. T.; Skrydstrup, T. Catalytic Hydrogenation of Polyurethanes to Base Chemicals: From Model Systems to Commercial and End-of-Life Polyurethane Materials. *JACS Au* **2021**, *1* (4), 517–524. https://doi.org/10.1021/jacsau.1c00050.

(12) Zhou, W.; Neumann, P.; Al Batal, M.; Rominger, F.; Hashmi, A. S. K.; Schaub, T. Depolymerization of Technical-Grade Polyamide 66 and Polyurethane Materials through Hydrogenation. *ChemSusChem* **2021**, *14* (19), 4176–4180. https://doi.org/https://doi.org/10.1002/cssc.202002465.

(13) Zhou, W.; Neumann, P.; Al Batal, M.; Rominger, F.; Hashmi, A. S. K.; Schaub, T.

Depolymerization of Technical-Grade Polyamide 66 and Polyurethane Materials through Hydrogenation. *ChemSusChem* **2020**, *n/a* (n/a). https://doi.org/https://doi.org/10.1002/cssc.202002465.
(14) Gausas, L.; Donslund, B. S.; Kristensen, S. K.; Skrydstrup, T. Evaluation of Manganese Catalysts for the Hydrogenative Deconstruction of Commercial and End-of-Life Polyurethane Samples. *ChemSusChem* **2022**, *15* (1), e202101705. https://doi.org/https://doi.org/10.1002/cssc.202101705.
(15) Zubar, V.; Haedler, A. T.; Schütte, M.; Hashmi, A. S. K.; Schaub, T. Hydrogenative Depolymerization of Polyurethanes Catalyzed by a Manganese Pincer Complex. *ChemSusChem* **2022**, *15* (1), e202101606. https://doi.org/https://doi.org/10.1002/cssc.202101606.
